# Haplotype-phased genome and evolution of phytonutrient pathways of tetraploid blueberry

**DOI:** 10.1093/gigascience/giz012

**Published:** 2019-01-31

**Authors:** Marivi Colle, Courtney P Leisner, Ching Man Wai, Shujun Ou, Kevin A Bird, Jie Wang, Jennifer H Wisecaver, Alan E Yocca, Elizabeth I Alger, Haibao Tang, Zhiyong Xiong, Pete Callow, Gil Ben-Zvi, Avital Brodt, Kobi Baruch, Thomas Swale, Lily Shiue, Guo-qing Song, Kevin L Childs, Anthony Schilmiller, Nicholi Vorsa, C Robin Buell, Robert VanBuren, Ning Jiang, Patrick P Edger

**Affiliations:** 1 Department of Horticulture, Michigan State University, 1066 Bogue Street, East Lansing, MI, 48824, USA; 2 MSU AgBioResearch, Michigan State University, 446 West Circle Drive, East Lansing, MI, 48824, USA; 3 Department of Plant Biology, Michigan State University, 612 Wilson Road, East Lansing, MI, 48824 USA; 4 Ecology, Evolutionary Biology and Behavior, Michigan State University, 293 Farm Lane, East Lansing, MI, 48824, USA; 5 Department of Biochemistry, Purdue University, 175 South University Street, West Lafayette, IN, 47907, USA; 6 Purdue Center for Plant Biology, Purdue University, 610 Purdue Mall, West Lafayette, IN, 47907, USA; 7 Human Longevity Inc., 4570 Executive Drive, San Diego, CA 92121, USA; 8 Key Laboratory of Herbage and Endemic Crop Biotechnology, School of Life Sciences, Inner Mongolia University, 221 Aimin Road, Hohhot, 010070, China; 9 NRGene, 5 Golda Meir Street, Ness Ziona, 7403648, Israel; 10 Dovetail Genomics, 100 Enterprise Way, Scotts Valley, CA, 95066, USA; 11 Center for Genomics Enabled Plant Science, Michigan State University, 612 Wilson Road, East Lansing, MI, 48824, USA; 12 Mass Spectrometry & Metabolomics Core Facility, Michigan State University, 603 Wilson Road, East Lansing, MI, 48824, USA; 13 Department of Plant Biology, Rutgers University, 59 Dudley Road, New Brunswick, NJ, 08901, USA; 14 Philip E. Marucci Center for Blueberry and Cranberry Research and Extension, Rutgers University, 125A Lake Oswego Road, Chatsworth, NJ, 08019, USA; 15 Plant Resilience Institute, Michigan State University, 612 Wilson Road, East Lansing, MI, 48824 USA

**Keywords:** Blueberry, Vaccinium, Phytonutrients, Genome, Haplotype-phased, Tetraploid, Polyploid, Subgenome Dominance

## Abstract

**Background:**

Highbush blueberry (*Vaccinium corymbosum*) has long been consumed for its unique flavor and composition of health-promoting phytonutrients. However, breeding efforts to improve fruit quality in blueberry have been greatly hampered by the lack of adequate genomic resources and a limited understanding of the underlying genetics encoding key traits. The genome of highbush blueberry has been particularly challenging to assemble due, in large part, to its polyploid nature and genome size.

**Findings:**

Here, we present a chromosome-scale and haplotype-phased genome assembly of the cultivar “Draper,” which has the highest antioxidant levels among a diversity panel of 71 cultivars and 13 wild *Vaccinium* species. We leveraged this genome, combined with gene expression and metabolite data measured across fruit development, to identify candidate genes involved in the biosynthesis of important phytonutrients among other metabolites associated with superior fruit quality. Genome-wide analyses revealed that both polyploidy and tandem gene duplications modified various pathways involved in the biosynthesis of key phytonutrients. Furthermore, gene expression analyses hint at the presence of a spatial-temporal specific dominantly expressed subgenome including during fruit development.

**Conclusions:**

These findings and the reference genome will serve as a valuable resource to guide future genome-enabled breeding of important agronomic traits in highbush blueberry.

## Introduction

Since domestication efforts began in the early 1900s [1], highbush blueberry (*Vaccinium corymbosum* L.) has rapidly become a high-value fruit crop worldwide [2–4]. Highbush blueberry, compared to hundreds of closely related blueberry species (e.g., huckleberry, *Vaccinium ovatum* Pursh; bilberry, *Vaccinium myrtillus* L.; and sparkleberry, *Vaccinium arboreum* Marshall) in the Ericaceae [5, 6], is widely cultivated due to its adaptation to temperate climates, excellent fruit quality, yield, and composition of phytonutrients [7]. As a result for the demand for fresh blueberries as a "superfruit" [8], highbush blueberry production has increased 600% during the past three decades and steadily grown to a multi-billion dollar industry [9]. In addition to its short domestication history, highbush blueberry is unique in being one of only three major commercially valuable fruit crops, accompanied by cranberry (*Vaccinium macrocarpon* Ait.) [10] and the garden strawberry (*Fragaria x ananassa*) [11], with wild progenitor species native to North America.

Blueberries have a single epidermal layer that expresses a rich profile of anthocyanins during ripening that, in combination with epicuticular wax, generates its characteristic "powdery blue" color. The cuticular and epidermal layers contain nearly all of the phytonutrients in the fruit such as anthocyanins, proanthocyanidins, and flavonols [12–14]. Previous studies on blueberry have reported that these groups of compounds may have diverse health-promoting properties, including controlling diabetes, improving cognitive function, and inhibiting tumor growth [15–21]. With the growing awareness of the potential health benefits of blueberry and increasing consumer demand, a primary goal of the blueberry research community is to develop cultivars with improved antioxidant levels along with other important fruit quality traits (e.g., aroma, taste, and firmness) [22]. However, despite its economic importance and health benefit potential, breeding efforts to improve fruit quality traits in blueberry have been slow due, in large part, to the lack of genomic resources. A draft genome for a wild diploid species (2*n* = 2*x* = 24) of blueberry was previously assembled [23]. However, that draft genome consists of a large number of scaffolds (13,757 total; N50 of ∼145 kb), high percentage of gaps (∼27.35%) in a ∼393.16 Mb assembly, and, most importantly, does not reflect the genome complexity of the economically important and cultivated tetraploid (2*n* = 4*x* = 48) highbush blueberry.

Here, we present the first chromosome-scale genome assembly of tetraploid highbush blueberry. The haplotype-phased assembly consists of 48 pseudomolecules with ∼1.68 Gb of assembled sequence, ∼1.29% gaps, and an average of 32,140 protein coding genes per haplotype (128,559 total). A haplotype is the complete set of DNA within the nucleus of an individual that was inherited from one parent. We leveraged this genome to examine the origin of the polyploid event, gain insights into the underlying genetics of fruit development, and identify candidate genes involved in the biosynthesis of metabolites contributing to superior fruit quality. Furthermore, we examined gene expression patterns among the four haplotypes in highbush blueberry. This analysis uncovered the presence of spatial-temporal specific dominantly expressed subgenomes. These findings and the reference genome will serve as a powerful platform to further investigate "subgenome dominance" [24–26], facilitate the discovery and analysis of genes encoding economically important traits, and ultimately enable molecular breeding efforts in blueberry.

## Results

### Assembly and annotation of the tetraploid highbush blueberry genome

**Figure 1:**
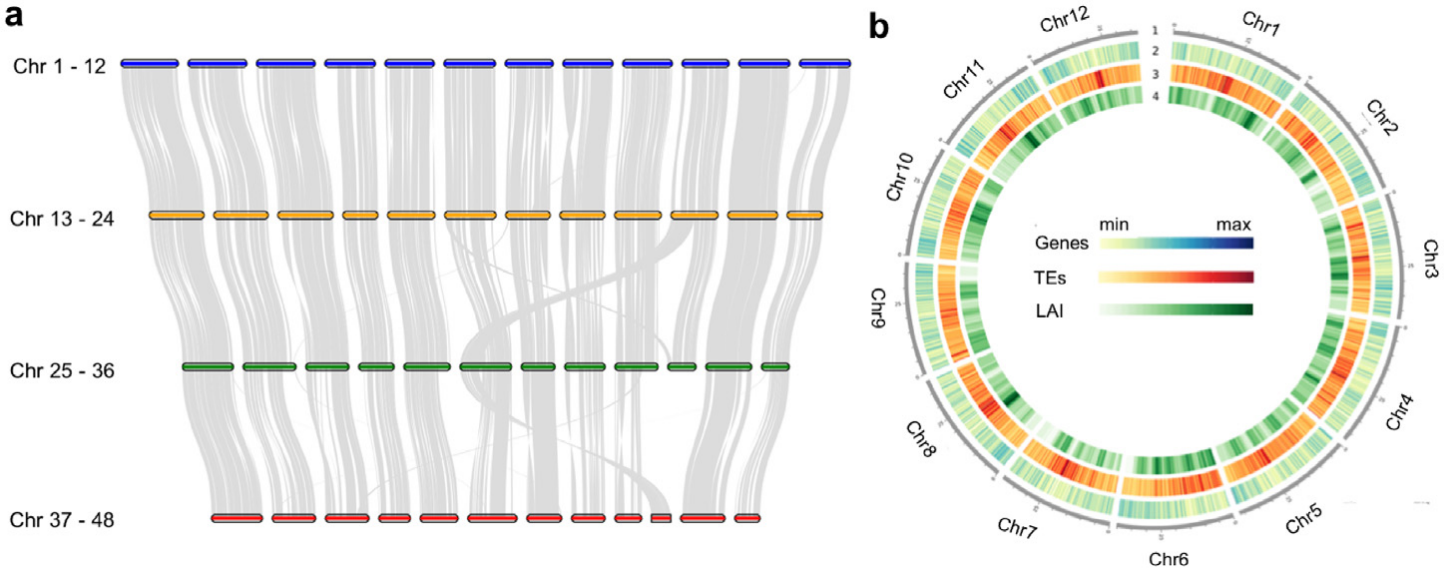
The haplotype-phased chromosome-scale highbush blueberry genome. **(a)** Collinearity among the homoeologous chromosomes. The gray lines represent conserved gene arrays between chromosomes. Chromosomes were drawn proportionally with respect to the number of genes on each chromosome. **(b)** Gene and transposable element (TE) density and LTR assembly index (LAI) in chromosomes 1–12 plotted in 300 Kb sliding window using Circos. The tracks from outside to inside are: 1 = chromosomes, 2 = gene density, 3 = TE density, and 4 = LAI score.

Our goal was to obtain a high-quality reference genome for the highbush blueberry cultivar “Draper,” which is widely grown around the world due to its excellent fruit quality. We sequenced the genome using a combination of both 10× Genomics (Pleasanton, CA) and Illumina (San Diego, CA), totaling 324X coverage of the genome (Supplementary Table S1). These data were assembled and scaffolded using the software package DenovoMAGIC3 (NRGene, Nes Ziona, Israel) (Supplementary Table S2). The genome was further scaffolded to chromosome-scale using Hi-C data (91.4X coverage) with the HiRise pipeline (Dovetail, Santa Cruz, CA) (Supplementary Figs. S1 and S2). The total length of the final assembly is 1,679,081,592 bases distributed across 48 chromosome-level pseudomolecules (Fig. 1). The final assembly size falls within the estimated genome size of "Draper" based on flow cytometry (1.63 Gb with 95% confidence interval +/− 0.06 Gb) (Extended Data Table 1).

The genome was annotated using a combination of evidence-based and *ab initio* gene prediction using the MAKER-P pipeline [27] (Supplementary Table S3). RNA sequencing (RNA-seq) data from 13 different gene expression libraries, representing unique organs, developmental stages, and treatments (Supplementary Table S4), and publicly available transcriptome and expressed sequence tags (EST) data of *V. corymbosum* in theNational Center for Biotechnology Information (NCBI) were used as transcript evidence. Protein sequences from *Arabidopsis thaliana* [28, 29], *Actinidia chinensis* [30], and UniprotKB plant database were also used as evidence for genome annotation. We predicted a total of 128,559 protein-coding genes. Benchmarking Universal Single-Copy Orthologs analysis (BUSCO, RRID:SCR_015008) v.3 [31] was performed to assess the completeness of the assembly and quality of the genome annotation. The annotated gene set contains 1,394 out of 1,440 (97%) BUSCO genes (Supplementary Table S5). Functional annotation was assigned using Basic Local Alignment Search Tool (BLAST) 2GO [32] to reference pathways in the Kyoto Encyclopedia of Genes and Genomes database [33] (Supplementary Fig. S3). Comparative genomic analyses assigned genes to 16,909 orthogroups shared by six phylogenetically diverse plant species including five eudicots (*A. chinensis* [30], *A. thaliana* [28, 29], *Fragaria vesca* [34], *Rubus occidentalis* [35], and *Vitis vinifera* [36]), each with distinct fruit types, and *Zea mays* [37] as the outgroup.

Transposable elements (TEs), both Class I and II, were identified and classified in the genome using the protocol described by Campbell et al. [27]. Overall, 44.3% of the blueberry genome is composed of TEs (Supplementary Table S6). Consistent with previous reports [38, 39], the most abundant Class I TEs were long terminal repeat retrotransposons (LTR-RTs), specifically the superfamily LTR/*Gypsy* followed by LTR/*Copia*, while for Class II transposons, the miniature inverted repeat (MITE) superfamily *hAT* was the most abundant. The quality of the genome was further assessed by examining the assembly continuity of repeat space using the LTR Assembly Index (LAI) deployed in the LTR_retriever package (v1.8) [40]. The adjusted LAI score of this blueberry genome is 14, and based on the LAI classification, this score is within the range of "reference" quality (Fig. 1). Estimation of the regional LAI in 3 Mb sliding windows also showed that assembly continuity is uniform and of high quality across the entire genome.

### Assessment of the origin of tetraploid highbush blueberry

The origin of highbush blueberry from either a single (i.e., autopolyploid) or multiple diploid progenitor species (i.e., allopolyploid) is a long-standing question [41]. Previous reports have suggested that highbush blueberry may be an autotetraploid based on the segregation ratios of certain traits [42]. However, an analysis of chromosome pairing among different cultivars revealed largely bivalent pairing during metaphase I [43], similar to patterns observed in known allopolyploids [44, 45]. To gain further insights into the polyploid history of highbush blueberry, we calculated sequence similarity and synonymous substitution (*Ks;* silent mutation) rates between genes in homoeologous regions across the genome. The average sequence similarity is ∼96.3% among syntenic homoeologous genes. The average *Ks* divergence between syntenic homoeologous genes is ∼0.036 per synonymous site. The average *Ks* divergence between homoeologous genes can be used to not only identify polyploid events [46–48] but also to estimate the divergence of the diploid progenitors from their most recent common ancestor (MRCA) [49]. The *Ks* divergence between homoeologs in highbush blueberry is six times higher than that between orthologs of two *A. thaliana* lines (Col and Ler; *Ks* of ∼0.006) that diverged roughly 200,000 years ago [50]. Based on the relatively high *Ks* rate between homoeologous regions across the genome, this suggests that tetraploid blueberry is unlikely an autopolyploid that was formed from somatic doubling or failure during meiosis involving a single individual (parent).

Furthermore, comparative genomics revealed that homoeologous regions are highly collinear, except a few notable chromosome-level translocations (Fig. 1a). These translocations were manually inspected and verified with both the raw sequence and Hi-C data. Rapid changes among homoeologous chromosomes is known to occur in newly formed allopolyploids [44, 45, 51]. We also assessed the level of similarity and content of LTR transposable elements among the four haplotypes. As the most prevalent transposable elements in plants, LTR-RTs undergo continual "bloat and purge" cycles within most plant genomes [52], resulting in a unique signature that may distinguish subgenomes in an allopolyploid. To examine the evolutionary history of LTR-RTs in the highbush blueberry genome, we calculated the mean sequence identity of LTR sequences among each of the four haplotypes (Supplementary Fig. S4). This analysis revealed that the majority of more recent LTRs (>97% similarity) are subgenome specific in highbush blueberry. In other words, the data suggest that LTRs proliferated independently in the genomes of each diploid progenitor (i.e., subgenome), following the divergence from their MRCA, but prior to polyploidy. The pair-wise LTR difference (d) of the two ancestors is 2.4%–2.6%. With Jukes-Cantor correction (K = −3/4*ln(1–4d/3)) and synonymous substitution rate of (µ = 1.3e-8) [53], the estimated time (T = K/2µ) of divergence for the diploid progenitors from their MRCA is between 0.94 to 1.02 million years ago.

These date estimates and the average speciation rate (λ = 0.59 per million years; [54]) for temperate angiosperms suggests that highbush blueberry is either an allopolyploid derived from two closely related species or an autopolyploid derived from the hybridization of two highly divergent populations of a single species. To date the most recent polyploid event in highbush blueberry, we analyzed the unique LTR insertions present in each haplotype. Based on the pair-wise LTR difference between the four haplotypes, which is of 0.81%–0.89%, the polyploid event occurred approximately 313 to 344 thousand years ago. The substitution rate of LTR sequences is likely different from that of protein coding genes. Thus, more accurate date estimates will be possible once the LTR substition rate in highbush blueberry becomes available from future studies.

After allopolyploidization, one of the parental genomes (i.e., subgenomes) often emerges with significantly greater gene content and a greater number of more highly expressed genes [55–58]. The emergence of a dominant subgenome in an allopolyploid is hypothesized to resolve genetic and epigenetic conflicts that may arise from the merger of highly divergent subgenomes into a single nucleus [26, 59, 60]. However, classic autopolyploids, formed by somatic doubling, are not expected to face these challenges or exhibit subgenome dominance since all genomic copies were contributed by a single parent [61]. This was recently supported by genome-wide analyses of a putative ancient autopolyploid (soybean; *Glycine max*) [62]. It's important to note that subgenome expression dominance could still be observed in intraspecific hybrids and autopolyploids formed by parents with highly differentiated genomes [25].

**Figure 2:**
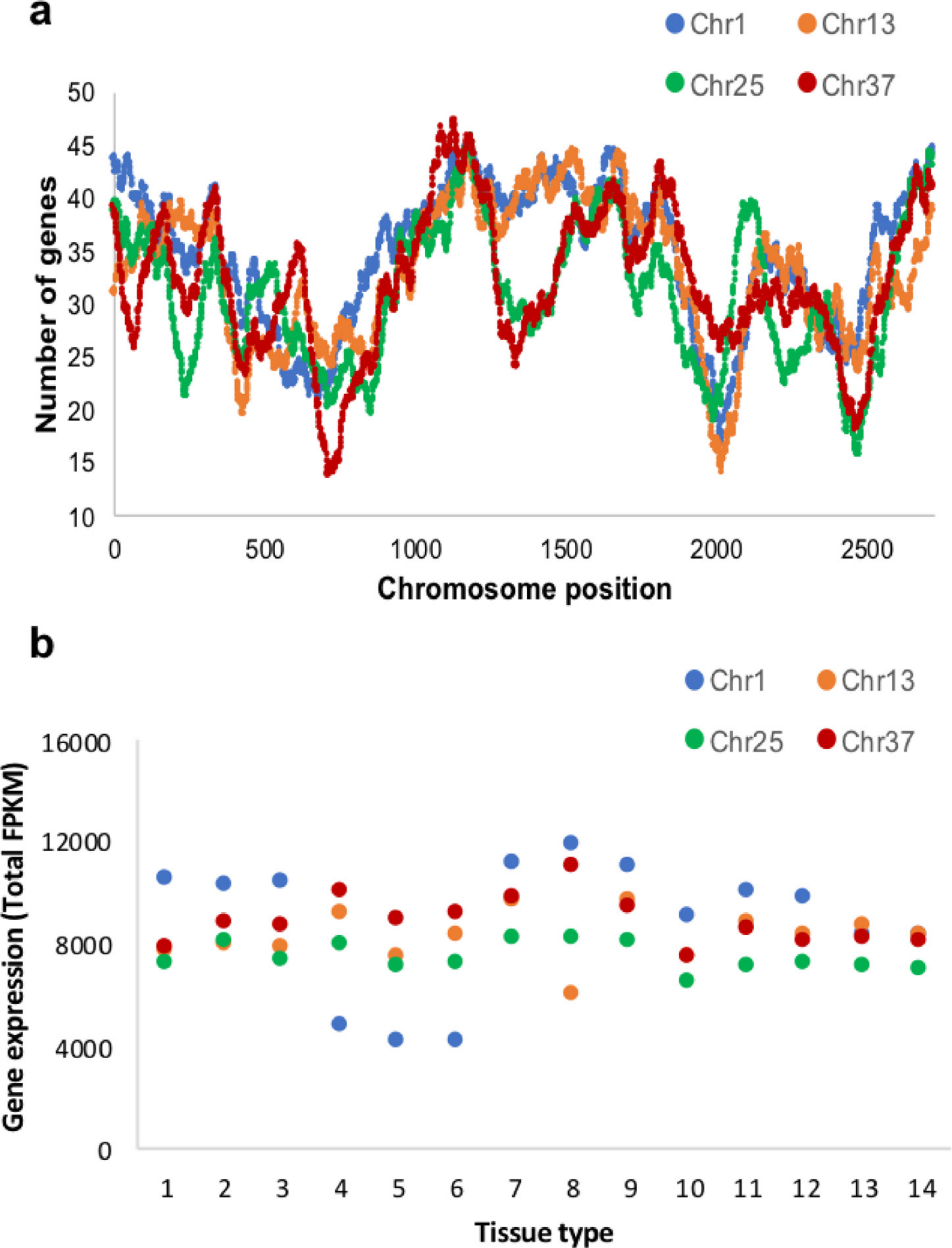
Assessment of the origin of polyploid blueberry. **(a)** Gene content comparison of homoeologous chromosomes (1, 13, 25, and 37) plotted along 2,725 collinear syntenic regions. This analysis for all 48 chromosomes can be regenerated here: [65]. **(b)** Gene expression comparison (FPKM; fragments per kilobase per million) among the same four homoeologous chromosomes across different blueberry tissues (1 = flower bud; 2 = flower at anthesis; 3 = petal fall; 4 = green fruit; 5 = pink fruit; 6 = ripe fruit; 7, 8 = leaf collected at 12 p.m. and 12 a.m., respectively; 9, 10, 11 = methyl jasmonate-treated leaf collected after 1 hour, 8 hours, and 24 hours, respectively; 12 = shoot; 13 = root; 14 = salt-treated root).

To explore this in highbush blueberry, we compared gene content and expression-level patterns between homoeologous chromosomes (Fig. 2). While gene content levels were largely similar among homoeologous chromosomes, with a few notable exceptions (Fig. 2a), gene expression levels were highest for one of the four chromosome copies in the majority (average 9.3 of 14) of gene expression libraries (112 of 168 comparisons, x2 test *P* value < 0.001)(Fig. 2b, Supplementary Fig. S5). Noteworthy, in the three fruit libraries, the most dominantly expressed often became the least expressed among the four homoeologous chromosomes (19 of 36 comparisons; x2 test *P* value <0.01) or among the two lowest expressed copies (26 of 36 comparisons; x2 test *P* value <0.01). The most dominantly expressed in other tissues remained so in developing fruit for only two of the chromosomes (6 and 10). These homoeologous chromosome sets have undergone the most structural variation, which may have modified gene expression patterns (Fig. 1a). These analyses are based on a single biological replicate from a plant grown in a growth chamber. Thus, the findings reported here should be considered as preliminary. Future studies should further explore subgenome expression dominance in highbush blueberry, including at the individual homoeolog level [63, 64], with additional biological replicates and across multiple environments.

### Changes in transcript abundance during blueberry fruit development

The progression of fruit development in blueberry is marked with visible external and internal morphological changes including in size and color (Supplementary Fig. S6a). We profiled gene expression in fruit across seven developmental stages from the earliest stage (i.e., post-fertilization) through the final stage (i.e., ripe fruit) to identify genes differentially expressed during fruit development. Distinctive transitions in gene expression were observed between early fruit growth to start of color development and complete color change to ripened fruit. We found that the majority of genes upregulated during early fruit development were involved in phenylpropanoid biosynthesis, nitrogen metabolism, as well as cutin, suberin, and wax biosynthesis (Supplementary Table S7a). In contrast, genes involved in starch and sugar metabolism were highly expressed at the onset of and during fruit ripening (Supplementary Table S7b). Moreover, principal component analysis showed the first two components accounted for 84% of the variation and separated the developmental stages into three groups: early developmental stages, petal fall and small green fruit; middle developmental stages, expanding green and pink fruit; and ,late developmental stages, complete fruit color change, unripe and ripe fruit (Supplementary Figs. S6a and S7).

Genes associated with cell division, cell wall synthesis, and transport were found to be expressed the highest during the earliest developmental stages (Extended Data Table 2), which is consistent with previous work on other fruit species [66, 67]. In addition to genes regulating cell proliferation, defense response-related genes were also highly upregulated during the earliest developmental stages. During the middle developmental stages, genes regulating cell expansion, seed development, and secondary metabolite biosynthesis were highly expressed. During late developmental stages and as the berry transitions to ripening, late embryogenesis, transmembrane transport, defense, secondary metabolite biosynthesis, and abscisic acid-related genes were highly overrepresented. Blueberry is considered a climacteric fruit; however,unlike the ethylene-driven fruit ripening in other climacteric species, abscisic acid has been demonstrated to regulate fruit ripening in blueberry [68]. In summary, global gene expression patterns mirror the morphological and physiological changes observed during blueberry development (Supplementary Fig. S6a).

### Antioxidant capacity in blueberry

The economic value of blueberry is largely determined by its fruit quality and nutritional value [7, 18, 69]. We assessed the total antioxidant capacity in mature fruit across a blueberry diversity panel and the abundance of secondary metabolites responsible for its antioxidant activity in developing fruit. A diversity panel, composed of 71 highbush blueberry cultivars and 13 wild *Vaccinium* species, was evaluated for total antioxidant capacity in mature fruit using the oxygen radical absorbance capacity (ORAC) assay [70]. Similar to previous reports [71–73], we observed a wide range in antioxidant capacity (∼5–95 nmol TE/mg FW) across cultivars, with "Draper" having the highest levels of antioxidants (Supplementary Fig. S6b). The observed variation in antioxidants among highbush blueberry, consistent with our results, were previously shown not to correlate with fruit weight or size [74]. However, in another study, a correlation between fruit size and total anthocyanin levels was identified within a few select highbush blueberry cultivars but not across other *Vaccinium* species or blackberry [75]. This inconsistency is likely due to sample size differences between studies.

To further examine the antioxidant capacity in "Draper" during fruit development, fruits from the seven aforementioned fruit developmental stages were assayed for antioxidant levels (Supplementary Fig. S6a). The highest level of antioxidants was observed at the earliest "petal fall" stage (537 nmol TE/mg FW) (Supplementary Fig. S8) after which, the level of antioxidants declined during the middle and late developmental stages. This is consistent with previous reports on the antioxidant activity in blueberry during fruit maturation [76] and similar to observations in blackberry and strawberry, wherein green fruit have the highest ORAC values [77]. The antioxidant capacity in blueberry is influenced by various metabolites including anthocyanins [12, 75, 78]. Using the same fruit development series, we quantified anthocyanin and flavonol aglycones in "Draper" using liquid chromatography-mass spectrometry (LC-MS). Overall, as the fruit changed its exocarp color from pink to dark blue during ripening, delphinidine-type anthocyanins started to accumulate and were the most abundant compound in ripe fruit (181 peak area/IS/gDW) followed by cyanidin, malvidin, and petunidin (Supplementary Fig. S6c). Flavonols were also detected in all developmental stages, with quercetin glycoside being the most abundant (88 peak area/IS/gDW), while myricetin glycoside and rutin were present at very low levels.

Blueberry also has high levels of phenolic acids; among phenolics, chlorogenic acid (CGA) was the most abundant. High levels of CGA were observed throughout fruit development, with the highest accumulation detected in young fruits (Supplementary Fig. S6d). This correlates with the pattern of antioxidant capacity across different fruit stages, suggesting that CGA is one of the major metabolites contributing to high ORAC values in young developing fruit. CGA is derived from caffeic acid and quinic acid and has vicinal hydroxyl groups that are associated with scavenging reactive oxygen species [79–81]. The antioxidant properties of CGA have been associated with preventing various chronic diseases [82–86].

### Expression of antioxidant biosynthesis-related genes

**Figure 3:**
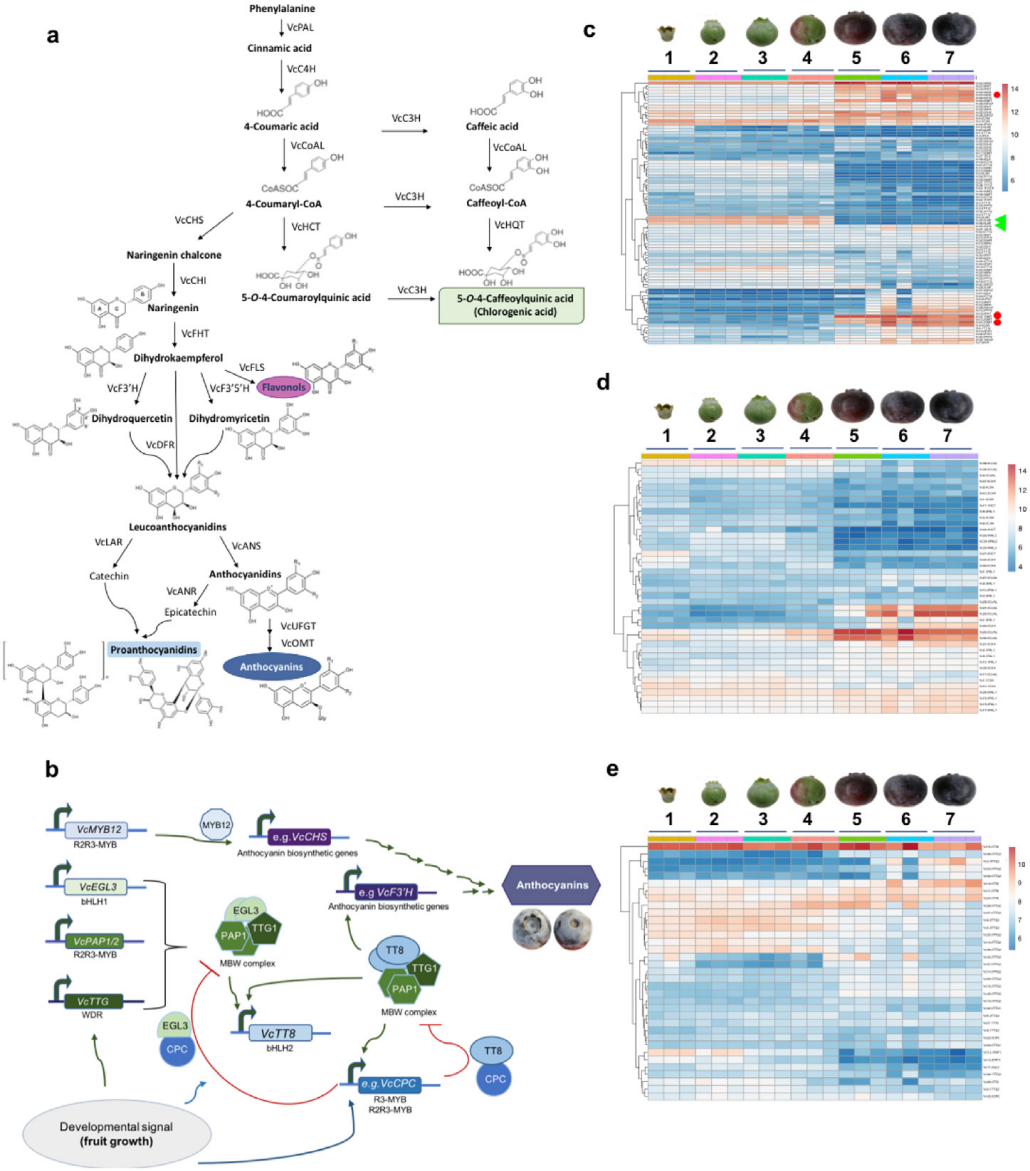
A schematic presentation of flavonoid biosynthesis in blueberry. **(a)** Predicted flavonoid biosynthetic pathway leading to production of anthocyanin. The proposed pathway is based on previously described flavonoid biosynthetic pathway in plants (Zifkin et al. [68]) and expression of predicted anthocyanin biosynthetic genes in blueberry. The core genes include phenylalanine ammonia-lyase (*PAL*), 4-hydroxycinnamoyl CoA ligase (*4CL*), trans-cinnamate 4-monooxygenase (*C4H*), cytochrome P450 98A3 (*C3H*), chalcone synthase (*CHS*), chalcone flavonone isomerase (*CHI*), flavanone-3β-hydroxylase (*FHT*), flavanone 3-hydroxylase (*F3H*), flavonoid 3′-hydroxylase (*F3′H*), flavonoid 3′,5′-hydroxylase (*F3′5′H*), dihydroflavonol reductase (*DFR*), leucoanthocyanidin reductase (*LAR*), anthocyanidin reductase (ANR), anthocyanidin synthase (*ANS*), UDP-glucose flavonoid 3-O-glucosyl transferase (*UFGT*), anthocyanin-O-methyltransferase (*OMT*), hydroxycinnamoyl-CoA shikimate/quinate hydroxycinnamoyltransferase (*HCT*), and hydroxycinnamoyl-CoA quinate hydroxycinnamoyltransferase (*HQT*). **(b)** Hypothetical regulatory pathway of anthocyanin biosynthetic genes based on the proposed model by Albert et al. [92]. **(c)** Developmental-specific expression pattern of key anthocyanin biosynthetic gene (green triangles = examples of genes upregulated during early fruit growth; red circles = examples of genes upregulated during late fruit development). **(d)** Chlorogenic acid biosynthetic genes (1 = petal fall, 2 = small green fruit, 3 = expanding green fruit, 4 = pink fruit, 5 = fruit color completely changed from pink to purple, 6 = unripe, 7 = ripe). **(e)** Expression profile of transcription factors predicted to regulate anthocyanin biosynthesis in blueberry. A high-resolution version of the heat maps is available on PURR (see Availability of Supporting Data section).

To better understand the biosynthesis of antioxidants in blueberry fruit, we identified homologs of previously characterized genes in other species involved in ascorbate, flavonols, chlorogenic acid, and anthocyanin biosynthesis (Fig. 3 and Extended Data Table 3) [68, 87–89]. The key biosynthetic genes for these compounds exhibited a distinct developmental-specific pattern of expression (Fig. 3c-3e and Supplementary Fig. S9). For example, genes involved in the conversion of leucoanthocyanidins to proanthocyanidins (e.g., *LAR* and *ANR*) are highly expressed in the earliest and middle developmental fruit stages but not in ripening fruit (Fig. 3c, green triangle, and Extended Data Table 4). Conversely, genes involved in the conversion of leucoanthocyanidins to anthocyanins (e.g., *ANS*, *UFGT*, and *OMT*) were highly expressed in mature and ripe fruit but not during early fruit developmental stages (Fig. 3c, red circle, and Extended Data Table 4). Additionally, paralogs encoding the same anthocyanin pathway enzymes (e.g., FHT, OMT) and genes involved in vacuolar localization of proanthcyanidins (e.g., glutathione S-transferase and multidrug resistance-associated protein-type) exhibited similar developmental stage-specific expression patterns. The expression of these biosynthetic genes is regulated by specific transcription factors [90]. For example, the transcription factor complex MYB-bHLH-WD regulates expression of anthocyanin biosynthetic genes in eudicots [91–94]. Using the Plant Transcription Factor Database v.4.0 [95], we identified homologs of transcription factors belonging to 55 gene families, and members of some of these gene families were predicted to be involved in the developmental regulation of flavonoid biosynthesis during blueberry fruit growth (Extended Data Table 4), including *R2-R3-MYBs*, *R3-MYBs*, *bHLHs*, and *WDRs* (Fig. 3b, 3e). These transcription factors also exhibit fruit development-specific expression patterns.

In addition, we performed a gene co-expression network analysis to identify metamodules of genes that appear co-regulated during fruit development, specifically genes that are associated with phytonutrient biosynthesis. Our analysis identified 1,988 metamodules of co-expressed genes, of which 428 metamodules contained at least one of the 57 Pfam domains that have been previously categorized as associated with specialized metabolic pathways in plants [96]. Our analysis revealed that 142 of 428 metamodules were more highly expressed in developing fruit compared to other plant tissues. Some metamodules showed clear trends of being highly expressed during either early or late fruit development. For example, METAMOD00377 is expressed early in fruit development and contains homologs to known anthocyanin genes *OMT, HCT, PAL*,and*HQT* as well as 31 homologs to known transcription factors. In contrast, METAMOD01221 is expressed late in fruit development and contains homologs of *HCT, TT19, UFGT*,and*OMT* and contains 10 homologs to known transcription factors. Moreover, we also examined metamodules for genes associated with other biosynthetic pathways that impart unique blueberry fruit characteristics. We identified two metamodules where genes appear to be co-regulated. Metamodule METAMOD00377, which contains Pfam domains associated with terpene, saccharide, and alkaloid specialized metabolism, and METAMOD01221, which contains terpene and saccharide metabolism. These metamodules contained genes that are differentially expressed during fruit development. Overall, the developmental-specific expression patterns of key biosynthetic genes and their putative transcriptional regulators emphasize the tight regulation of production, conversion, and transport of precursor compounds that lead to the accumulation of antioxidant-related metabolites in blueberry.

### Fruit aroma and the role of terpenes

The coregulation of genes involved in the biosynthesis of terpenes and saccharides during early and late fruit development described above reflects a coordinated interplay between these metabolites during fruit growth. Both terpenes and sugars contribute to the characteristic flavor of ripened fruit [97]. In blueberry, two components play a central role in flavor perception: taste, which is a balance of sweetness and acidity, and aroma. Blueberry aroma is a complex blend of volatiles that include aldehydes, esters, terpenes, ketones, and alcohols [98, 99]. Previous reports in blueberry showed that the aroma profile varies greatly across different blueberry ecotypes and cultivars [100–102]. For example, the aroma of highbush blueberry is primarily driven by terpene hydrocarbons (e.g., linalool, geraniol, hydroxycitronellol) and aldehydes (e.g., (E)-2-hexenal, (E)-2-hexenol, (Z)-3-hexenol) [98, 103]. Both linalool and geraniol are associated with sweet floral flavor. However, linalool was reported to largely impart the characteristic blueberry flavor when combined with certain aldehydes [98].

Here, we also identified and examined the expression of genes involved in the biosynthesis of linalool. Four of the linalool synthase homologs in tetraploid blueberry are highly expressed during late fruit development (Extended Data Table 5). This pattern of expression coincides with previous reports of linalool accumulation in ripened blueberry fruit [99, 103, 104]. On the other hand, one homolog of linalool synthase, although it was expressed during fruit growth, did not show a clear fruit development-specific pattern. Investigating the underlying factors regulating these enzymes will facilitate genetic manipulations that may lead to further improving blueberry flavor in the future.

### Sugar transporters

Superior fruit quality is also associated with sugar levels [105]. During fruit ripening, sugar levels of the endocarp increase by importing hexose symplastically and/or apoplastically. Sugar transporters (i.e., sugar will eventually be exported transporter [*SWEET*]), sucrose transporter, and tonoplast sugar transporter (*TST*) have been demonstrated to regulate intercellular sugar transport in phloem and fruit [106, 107]. In *A. thaliana*, all clade III *SWEET* play a role in sucrose transport, with *AtSWEET9* primarily functioning in nectary secretion [108], while *AtSWEET15* is required for seed filling by acting with *SWEET11* and *SWEET12* [109]. In blueberry, the clade III *SWEET transporters 9* and *10* were highly expressed during early fruit growth, while clade III *SWEET transporter 15* was mainly expressed in ripe fruit (Extended Data Table 5). Interestingly, one of the blueberry *SWEET15* homologs showed a distinct pattern of expression compared to the other three homologs. To the best of our knowledge, we are the first to report on the potential role of these genes during blueberry fruit development.

In addition, homologs of *A. thaliana TST1* [110] and watermelon *ClTST1* and *ClTST3* (tonoplast sugar transporters) [107] were expressed during fruit ripening in blueberry. Elevated expression of a *ClTST1* homolog was observed throughout fruit development, but the *ClTST3* homolog showed very low expression. Another gene that is highly expressed during fruit maturation is vacuolar invertase. As described in other systems [111], its upregulation during fruit ripening coincided with the breakdown of starch to sucrose or a mixture of glucose and fructose, suggesting that it may be involved in the regulation of sugar accumulation in blueberry fruit. It was previously reported that vacuolar invertase modulates the hexose to sucrose ratio in ripening fruit [112]. In addition, there are also two sugar transport protein homologs that exhibited developmental specific expression. However, their function remains largely unknown, thus, their potential role in sugar accumulation in the developing berry requires further investigation.

### Expansion of antioxidant-related gene families through tandem duplication

Tandemly duplicated genes arise as a result of unequal crossing over or template slippage during DNA repair [113, 114], exhibit high birth-death rates (i.e., predominantly young) [46], and typically are in co-regulated clusters in the genome [115]. Smaller-scale duplications [116], which include tandem duplicates, are highly biased toward certain gene families [117] including those involved in specialized metabolism [118–120]. Furthermore, tandem duplications often results in the increased dosage of gene products [121] and may improve the metabolic flux of rate-limiting steps in certain biosynthetic pathways [122].

Most genes associated with the biosynthesis of antioxidants (CGA, flavonols, anthocyanins, proanthocyanidins) have at least one tandem duplicate present in the highbush blueberry genome, with tandem array sizes ranging from 2 to 10 gene copies (Extended Data Table 6). The largest tandem arrays were found for *HQT* and *HCT* genes, which are co-regulated and involved in the CGA pathway (Fig. 3a). Differences in tandem array sizes were also observed between homoeologous chromosomes for various genes. For example, the *C3H* gene, which is involved in CGA biosynthesis (Fig. 3a), was present on all four homoeologous chromosomes but with varying tandem array sizes. One of the homoeologous chromosomes had two copies of *C3H*, while the other three homoeologous chromosomes had four copies. This suggests that copy number differences of *C3H* among subgenomes may be due to either selection for gene duplication or loss or, in the case of allopolyploidy, may be due to preexisting gene content differences among the diploid progenitor species.

Genes in the anthocyanin pathway with other unique duplication patterns include *CHS*, *CHI*, *OMT*, and *UFGT*. The gene *CHS*, involved in the conversion of 4-coumaryl-CoA to naringenin chalcone, has two copies, and both have tandem duplicates in at least three of the homoeologous chromosomes. Interestingly, the gene *CHI* has a single preserved tandem gene duplicate on only one of the homoeologous chromosomes. However, additional copies of *CHI* were also identified more distantly away from the syntenic ortholog on another homoeologous chromosome, likely involving a transposition event following tandem duplication. The *OMT* and *UFGT* genes all have tandem duplicates on all of the homoeologous chromosomes, although with varying array sizes, while the *ANR* gene involved in the conversion of anthocyanidin to proanthocyanidin is single copy on all homoeologous chromosomes. *DFR* gene, which is involved in the conversion of dihydroquercetin/dihyromyricetin to leucoanthocyanidin, has a single tandem duplicate on only one of the homoeologous chromosomes. These findings suggest that there may have been greater selective pressure to retain tandem duplicates for genes encoding enzymes involved in anthocyanin production than conversion to proanthocyanidins.

The vast majority of tandem duplicates are eventually lost (i.e., nonfunctionalization); however, in rare instances, some may undergo functional diversification (e.g., sub- and/or neo-functionalization) [46, 123]. Gene expression analysis revealed that 83.4% of the tandem duplicates were expressed in at least one transcriptome library with 73.5% expressed in at least one of the fruit developmental stages. This suggests that a subset of these duplicate genes have nonfunctionalized, subfunctionalized, or neofunctionalized. Future studies are needed to more thoroughly investigate the functions of these genes with more diverse libraries and additional transcriptome analyses.

## Discussion

Despite the economic importance of blueberry, molecular breeding approaches to produce superior cultivars have been greatly hampered by inadequate genomic resources and a limited understanding of the underlying genetics encoding important traits. This has resulted in breeders having to solely rely on traditional approaches to generate new cultivars, each with widely varying fruit quality characteristics. For example, our analysis of a diversity panel consisting of 84 cultivars and wild species revealed that "Draper" has antioxidant levels that are up to 19x higher than other cultivars. Thus, the genome of "Draper" should serve as a powerful resource to the blueberry community for guiding future breeding efforts aimed at improving antioxidant levels among other important fruit quality traits. Furthermore, to our knowledge, this is not only the first genome assembly of the cultivated highbush blueberry but is also the first chromosome-scale and haplotype-phased genome for any species in the order Ericales. Ericales includes several other high-value crops (e.g., tea, kiwifruit, and cranberry) and wild species with unique life history traits (e.g., carnivorous, American pitcher plants; parasitic, *Sarcodes* "snow flower"; and extremophiles, "Jacob cactus"). Thus, we anticipate that this reference genome, plus associated datasets, will be useful for a wide variety of evolutionary studies.

Here, we also leveraged the genome to identify candidate genes and pathways that encode superior fruit quality in blueberry, including those associated with pigmentation, sugar, and antioxidant levels. Furthermore, we found that genes encoding key biosynthetic steps in various antioxidant pathways are enriched with tandem gene duplicates. For example, tandem gene duplications have expanded gene families that are involved in the biosynthesis of anthocyanins. This suggests that, in addition to a recent whole genome duplication, tandem duplications may have greatly contributed to the metabolic diversity observed in blueberry (as previously described in *Arabidopsis* [124]). These tandem duplicates may have evolved new functions (i.e., neofunctionalized), possibly involved in the biosynthesis of novel compounds, and/or were selected to improve the metabolic flux of specific biosynthetic steps that alter the dosage of certain endpoint metabolites [122]. Future studies are needed to further investigate the possible role of tandem duplications in having modified metabolite levels and composition in wild and cultivated blueberry.

Our analyses also revealed that highbush blueberry, a tetraploid, likely arose from the hybridization of two distinct parents, possibly allopolyploidy, based on the sequence divergence, unique transposable element insertions, and subgenome expression patterns. Our analyses revealed that the subgenomes in highbush blueberry may be controlling a distinct set of genetic programs (e.g., fruit development vs mature leaves). The dominantly expressed subgenome in most surveyed tissues becomes the lowest expressed during fruit development. This observation is similar to findings in allopolyploid wheat where developmental and adaptive traits were shown to be controlled by different subgenomes [125–127]. For example, cell type- and stage-dependent subgenome expression dominance was observed in the developing wheat grain [127]. We argue that both highbush blueberry and hexaploid wheat, each now with high-quality reference genomes [128], make excellent systems to further investigate these underlying mechanisms of subgenome dominance [25]. Subgenome dominance has far-reaching implications to numerous research areas including breeding efforts [58]. For example, marker-assisted breeding needs to target the correct set of dominant homoeologs given the trait in polyploids that exhibit subgenome dominance. Thus, we anticipate that this genome, combined with improved insights into subgenome dominance, will greatly accelerate molecular breeding efforts in the cultivated highbush blueberry.

## Materials and Methods

### Plant material

*Vaccinium corymbosum* cv. Draper was selected based on having the highest antioxidant levels among a diversity panel of leading cultivars and due to its overall importance to the industry (Supplementary Fig. S6). Furthermore, cultivar Draper was selected since germplasm is widely available to the community from blueberry nurseries. The genome size (1.63 +/− 0.06 Gb) was estimated using flow cytometry with four technical replicates from Flow Cytometry Core at Benaroya Research Institute at Virginia Mason (Seattle, WA)(Extended Data Table 1).

### Genomic sequencing

High-molecular-weight genomic DNA was isolated from young leaf tissue, following a 72-hour dark treatment, using a modified nuclei preparation method [129, 130]. DNA quality was verified by pulsed-field gel electrophoresis. DNA fragments longer than 50 Kb were used to construct a 10× Gemcode library using the Chromium instrument (10× Genomics; Pleasanton, CA) and sequenced at HudsonAlpha Institute for Biotechnology (Huntsville, AL) on a HiSeqX system (lllumina; San Diego, CA) with paired-end 150 bp reads. Approximately 95 Gb (∼58-fold coverage, based on an estimated genome size of 1.63 Gb) of 10X Chromium library data was sequenced (Supplementary Table S1). To increase sequence diversity and depth, three separate mate-pair (MP) libraries were constructed with 2–5  Kb, 5–7  Kb, and 7–10  Kb jumps using the Illumina Nextera Mate-Pair Sample Preparation Kit. In addition, two additional size-selected Illumina genomic libraries, ∼470 bp and ∼800  bp, were sequenced. The ∼470  bp and ∼800 bp libraries were made using the Illumina TruSeq DNA PCR-free Sample Preparation V2 kit. The ∼470  bp library was designed to produce "overlapping libraries" after sequencing with paired-end 265  bp reads on an lllumina Hiseq2500 system, producing "stitched" reads of approximately 265  bp to 520  bp in length. The 800  bp library was sequenced on an Illumina HiSeq2500 system with paired-end 160  bp reads, while the MP libraries were sequenced on an Illumina HiSeq4000 system with paired-end 150 bp reads. A total of ∼433 Gb (∼266× fold coverage) of additional Illumina sequencing data were generated (Supplementary Table S1). Illumina library construction and sequencing was conducted at Roy J. Carver Biotechnology Center, University of Illinois at Urbana-Champaign.

### Genome assembly

The genome of "Draper" was assembled using the DeNovoMAGIC software platform (NRGene, Nes Ziona, Israel), which is a de Bruijn graph-based assembler designed for higher polyploid, heterozygous, and/or repetitive genomes [131, 132]. The Chromium 10X data were utilized to phase, elongate, and validate haplotype scaffolds. Four Dovetail Hi-C libraries were prepared as described previously [133] and sequenced on an Illumina HiSeq X system with paired-end 150 bp reads to a total of 90.7X physical coverage of the genome (Supplementary Fig. S1). The *de novo* genome assembly, raw genomic reads, and Dovetail Hi-C library reads were used as input data for HiRise, a software pipeline designed specifically for using proximity ligation data to scaffold genome assemblies [134]. Illumina genomic and Dovetail Hi-C library sequences were aligned to the draft input assembly using a modified SNAP read mapper [135]. The separations of Dovetail Hi-C read pairs mapped within draft scaffolds were analyzed by HiRise to produce a likelihood model for genomic distance between read pairs, and the model was used to identify and break putative misjoins and to make joins to close gaps between contigs.

### Collection of blueberry tissue samples, RNA library preparation, and sequencing

Plant tissue samples (flower bud, flower at anthesis, flower post-anthesis, young shoot, leaves treated with methyl jasmonate, small green fruit, expanding green fruit, pink fruit, ripe fruit, and salt-treated and untreated roots) were collected from blueberry cv. Draper grown in the growth chamber (16/8 hours photoperiod; 408mE light intensity; 23/20C day/night temperature). For the fruit developmental series, three biological replicates each of berries at seven developmental stages (petal fall/cup, small green fruit, expanding green fruit, pink fruit, purple reddish fruit, purple unripe fruit, and blue ripe fruit) were collected from cv. Draper in a field at the Horticulture Teaching and Research Center, Michigan State University, in July 2017. All plant tissues were immediately flash frozen in liquid nitrogen, and total RNA isolation was performed using the KingFisher Pure RNA Plant kit (Thermo Fisher Scientific, MA). Isolated total RNA was quantified using a Qubit 3 fluorometer (Thermo Fisher Scientific, MA). RNA libraries were prepared according to the KAPA mRNA HyperPrep kit protocol (KAPA Biosystems, Roche, USA). All samples were submitted to the Michigan State University Research Technology Support Facility Genomics core and sequenced with paired-end 150 bp reads on an Illumina HiSeq 4000 system (Illumina, San Diego, CA).

### Genome annotation

The draft genome of *V. corymbosum* cv. Draper was annotated using the MAKER annotation pipeline [27]. Transcript and protein evidence used in the annotation included protein sequences downloaded from *A. thaliana* (Araport11) and UniprotKB plant databases, *V. corymbosum* ESTs from NCBI, and transciptome data assembled with StringTie [136] from different blueberry tissues (Supplementary Table S4). A custom repeat library and Repbase [137] were used to mask repetitive regions in the genome using Repeatmasker [138]. *Ab initio* gene prediction was performed using gene predictors SNAP [139] and Augustus (Augustus: Gene Prediction, RRID:SCR_008417) [140]. The resulting MAKER Max gene set was filtered to select gene models with Pfam domain and annotation edit distance <1.0. The filtered gene set (MAKER standard) was further scanned for transposase coding regions. The amino acid sequence of predicted genes was searched (BLASTP, 1e-10) against a transposase database [27]. The alignment between the genes and the transposases was further filtered for those caused by the presence of sequences with low complexity. The total length of genes matching transposases was calculated based on the output from the search. If more than 30% of gene length aligned to the transposases, the gene was removed from the gene set. Furthermore, to assess the completeness of annotation, the *V. corymbosum* Maker standard gene set was searched against the BUSCO v.3 [31] plant dataset (embryophyta_odb9). Genes were annotated with pfam domains using InterProScan (InterProScan, RRID:SCR_005829) v5.26–65.0 [141].

### Annotation of repetitive elements

To identify and classify repetitive elements in the genome, LTR retrotransposon candidates were searched using LTRharvest [142] and LTR_finder [143] and further identified and classified (e.g., Copia and Gypsy) using LTR_retriever [40]. A non-redundant LTR library was also produced by LTR_retriever. Miniature inverted transposable elements (MITEs) were identified using MITE-Hunter [144]. MITEs were manually checked for target site duplications and terminal inverted repeats and classified into superfamilies (e.g., *Mutator*, *hAT*, *Tc1Mariner/Stowaway*, and *PIF/Harbinger*). Those with ambiguous Target Site Duplication (TSD) and Terminal Inverted Repeats (TIR) were classified as “unknowns”. Using the MITE and LTR libraries, the *V. corymbosum* genome was masked using Repeatmasker. The masked genome was further mined for repetitive elements using Repeatmodeler [145]. The repeats were then categorized into two groups: sequences with and without identities. Those without identities were searched against the transposase database; if they had a match, they were considered a transposon. The repeats were then filtered to exclude gene fragments using ProtExcluder [27] and summarized using the ‘fam_coverage.pl’ script in the LTR_retriever package. The assembly continuity of repeat space was assessed using the LLAI [146] deployed in the LTR_retriever package [40]. LAI was calculated based on either 3 Mb sliding windows or the whole assembly using LAI = (Intact LTR-RT length * 100)/Total LTR-RT length. For the sliding window estimation, a step of 300 Kb was used (-step 300,000 -window 3,000,000). To account for dynamics of LTR retrotransposons, LAI was adjusted by the mean identity of LTR sequences in the genome based on all-versus-all blastn search, which was also performed by the LAI program [146].

### Transcriptome assembly and gene-expression analysis

Illumina adapters were removed from the raw reads using Trimmomatic/0.33 (Trimmomatic, RRID:SCR_011848) [147], and trimmed reads were filtered using FASTX Toolkit [148]. After quality assessment using FastQC (FastQC, RRID:SCR_014583; [149]), the filtered reads were then aligned to the *V. corymbosum* genome using STAR [150]. For the samples that were used for annotation, transcript assembly was performed *de novo* using StringTie. Counts of uniquely mapping reads were generated through HTSeq [151] for all 35 RNA-seq datasets (plant tissue samples as well as fruit developmental series samples). Multimapping reads were excluded from the analysis except for the tandem gene expression analysis. Differential gene expression analysis was performed using the DESeq2 pipeline [152] across fruit developmental stages with three biological replicates per developmental stage (e.g., stage 1 compared to stage 2)(Fig. 3). Gene expression values were derived by calculating the fragments per kilobase per million reads mapped (FPKM) values using the standard formula for FPKM (= read count/‘per million’ scaling factor)/gene length in kilobases [Kb]).

To construct the gene co-expression network, genes that were not expressed or very weakly expressed (count <5) in 30 or more conditions were first excluded from the analysis. The count data was then transformed into variance stabilized values using the variance stabilizing transformation function in DEseq [151]. Pairwise correlations of gene expression were calculated using Pearson correlation coefficient (PCC) and mutual rank (MR) [153, 154] using scripts available for download from the project's data repository [155]. MR scores were transformed to network edge weights using geometric decay function ***e*^−^^(MR-1/^*^x^*^)^**[156]; five different co-expression networks were constructed with *x* set to 5, 10, 25, 50, and 100, respectively. Edges with PCC <0.6 or edge weight <0.01 were excluded. For each network, modules of co-expressed genes were detected using ClusterONE v1.0 using default parameters [157], and modules with *P* value > 0.1 or quality score <0.2 were excluded. The results from all co-expression networks were then combined by collapsing modules into metamodules of nonoverlapping gene sets.

### Oxygen radical absorbance capacity analysis

Total antioxidant capacity of tissues from the fruit developmental panel was analyzed using the ORAC assay [70]. Briefly, ∼20–30 mg of frozen ground fruit tissue was measured for tissue samples prior to extraction. Sample extractions were performed on ground tissue using 1.8 mL of ice cold 50% acetone. Samples were vortexed and then put on a shaker for 5 minutes at room temperature. Samples were then centrifuged at 4°C for 15 minutes (4500 *g*). The ORAC assay was performed in a 96-well black microplate (Thermo Fisher Scientific, Waltham, MA) using the FLUOstar OPTIMA microplate reader (BMG LABTECH, Offenburg, Germany). Each reaction well contained 150 μL of 0.08 μM fluorescein and 25 μL of 75 mM phosphate buffer (blank), Trolox standards (6-hydroxy-2,5,7,8-tetramethylchroman-2-carboxylic acid), or diluted sample extracts. For blueberry tissue samples, 1:80–1:20 dilutions were used. Upon loading all appropriate wells, the 96-well microplate was put into the microplate reader and incubated for 10 minutes at 37°C. Following incubation, 25 μL of 150 mM AAPH (2,2′-azobis-2-methyl-propanimidamide, dihydrochloride) was added to each well, and fluorescence measurements began immediately. Fluorescence measurements (excitation: 485 nm, emission: 520 nm) were taken for 90 seconds per cycle for 70 cycles until the fluorescent probe signal was completely quenched. The area under the fluorescence decay curve (AUC) was calculated for each well. The total antioxidant capacity of a sample was calculated by subtracting the AUC from the blank curve from the AUC of the sample curve to obtain the net AUC. Using Trolox (water-soluble analog of vitamin E) of a known concentration, a standard curve was generated (12.5 μM–100 μM), and the total antioxidant capacity of each sample was calculated as Trolox equivalents. Each sample was run twice for two technical replicates. The coefficient of variation between technical replicates was required to be less than 0.20. Biological replicates (n = 3) were run for all tissues in the fruit developmental series.

### Assay of phenolics and anthocyanin content

Berries from "Draper" were collected as described above. Approximately 100 mg (∼10:1 solvent/tissue ratio) of each frozen ground sample was resuspended in extraction solvent in a 2 mL tube (80% methanol/20% water + 0.1% formic acid, containing 0.5 nM telmisartan [internal standard]). Ground tissue was immediately mixed thoroughly to prevent thawing during extraction and to prevent metabolism of analytes by enzymes in the samples. All tubes were spun down for 10 minutes at 13,000 × *g* to pellet protein and other insoluble material. Then, 1 mL of supernatant was transferred to an autosampler vial. Anthocyanin content was evaluated by LC-MS as follows: 5 uL of sample extract were separated using a 10 minute gradient on a Waters Acquity HSS-T3 UPLC column (2.1 × 100 mm) on a Waters Acquity UPLC system interfaced with a Waters Xevo G2-XS quadrupole time-of-flight mass spectrometer (Waters Corp, Milford, MA). Column temperature was maintained at 40°C, and the flow rate was 0.3 mL/min with starting conditions of 100% solvent A (water + 0.1% formic acid) and 0% solvent B (acetonitrile). The gradient was as follows: hold at 100% A for 0.5 minutes, ramp to 50% B at 6 minutes, then ramp to 99% B at 6.5 minutes, hold at 99% B to 8.5 minutes, return to 100% A at 8.51 minutes, and hold at 100% A until 10 minutes. Mass spectra were acquired in positive ion mode electrospray ionization over m/z 50–1500 in continuum mode using a data-independent MS^E^ method that acquires data under both low and high collision energy conditions with the high collision energy setting using a ramp from 20–80 V. Capillary voltage was 3 kV, desolvation temperature was 350°C, source temperature was 100°C, cone gas flow was 25 L/hr, and desolvation gas flow was 600 L/hr. Correction for mass drift was performed using continuous infusion of the lock mass compound leucine encephalin. Anthocyanins and other related flavonoids were identified based on accurate mass and fragmentation pattern. Peak areas were determined using Quanlynx within the Masslynx software package (Waters Corp). Relative anthocyanin content was calculated for each sample using the formula: reported peak area of the compound/peak area of internal standard/weight of extracted tissue (peak area/IS/gdw).

### Genomic and gene family analyses

The genome was aligned against itself in CoGe's SynMap program using LAST (LAST, RRID:SCR_006119) and default parameters [158]. Maximum distance between two matches was set to 20 genes, with minimum number of aligned pairs set to 10 genes. Tandemly duplicated genes were identified and filtered from CoGe outputs with a maximum distance of 10 genes. Fractionation bias was calculated, setting the max query and target chromosomes to 48. These analyses can be regenerated using the CoGe platform [65]. Protein sequences of blueberry was searched against previously characterized antioxidant related genes in *Arabidopsis* and other species in UniprotKB and NCBI databases using blastp in the BLAST+ package [159] with a cut-off e-value of 1E-10.

## Supplementary Material

GIGA-D-18-00370_Original_Submission.pdf

GIGA-D-18-00370_Revision_1.pdf

Response_to_Reviewer_Comments_Original_Submission.pdf

Reviewer_1_Report_Original_Submission -- Robert Henry 10/3/2018 Reviewed

Reviewer_2_report_original_submission_revise -- Manuel Spannagl 10/18/2018 Reviewed

Reviewer_2_report_revision_1_revise -- Manuel Spannagl 1/14/2019 Reviewed

Supplemental Files

## Data Availability

The genome assembly, annotations, and other supporting data are publicly available on PURR [155] and also via the *GigaScience* database GigaDB [160] and the CyVerse CoGe platform [65,161]. The raw sequence data were deposited in the Short Read Archive under NCBI BioProject ID PRJNA494180.

## Additional files

Extended Data Table 1

Extended Data Table 2

Extended Data Table 3

Extended Data Table 4

Extended Data Table 5

Extended Data Table 6

## Abbreviations

ANR: anthocyanidin reductase; ANS: anthocyanidin synthase; BLASTP: Basic Local Alignment Search Tool (Protein); bHLH: basic helix-loop-helix; bp: base pair; BUSCO: Benchmarking Universal Single-Copy Orthologs; CGA: chlorogenic acid; 4CL: 4-hydroxycinnamoyl CoA ligase; C3H: cytochrome P450 98A3; C4H: trans-cinnamate 4-monooxygenase; CHI: chalcone flavonone isomerase; CHS: chalcone synthase; DFR: dihydroflavonol reductase; EST: expressed sequence tags; F3H: flavanone 3-hydroxylase, F3’H: flavonoid 3′-hydroxylase; F3’5’H: flavonoid 3′,5′-hydroxylase; FHT: flavanone-3β-hydroxylase; FPKM: fragments per kilobase per million; kb: kilo base; HCT: hydroxycinnamoyl-CoA shikimate/quinate hydroxycinnamoyltransferase; HQT: hydroxycinnamoyl-CoA quinate hydroxycinnamoyltransferase; LAI: LTR Assembly Index; LAR: leucoanthocyanidin reductase; LC-MS: liquid chromatography-mass spectrometry; LTR: long terminal repeats; LTR-RT: long terminal repeat retrotransposons; Mb: mega base; MRCA: most recent common ancestor; MITE: miniature inverted repeat; MP: mate-pair; MR: mutual rank; MRP: multidrug resistance-associated protein; MYB: myeloblastosis; OMT: anthocyanin-O-methyltransferase; ORAC: oxygen radical absorbance capacity; PAL: phenylalanine ammonia-lyase; PCC: Pearson's correlation coefficient; SWEET: Sugar Will Eventually be Exported Transporter; TE: transposable element; TST: tonoplast sugar transporter; UFGT: UDP-glucose flavonoid 3-O-glucosyl transferase; UPLC: Ultra Performance Liquid Chromatography; WDR: WD40 repeats.

## References

[1] Coville FV Experiments in Blueberry Culture. U.S. Government Printing Office; 1910.

[2] Ballington JR Collection, utilization, and preservation of genetic resources in Vaccinium. HortScience. American Society for Horticultural Science. 2001;36:213–20.

[3] Lewis NM, Ruud J Blueberries in the American diet. Nutr Today. 2005;40:92.

[4] Faostat F Statistical data. Rome: Food and Agriculture Organization of the United Nations; 2017.

[5] Kron KA, Judd WS, Stevens PF, et al. Phylogenetic classification of ericaceae: molecular and morphological evidence. Bot Rev The New York Botanical Garden. 2002;68:335–423.

[6] Schwery O, Onstein RE, Bouchenak-Khelladi Y, et al. As old as the mountains: the radiations of the Ericaceae. New Phytol. 2015;207:355–67. 25530223 10.1111/nph.13234

[7] Michalska A, Lysiak G Bioactive compounds of blueberries: post-harvest factors influencing the nutritional value of products. Int J Mol Sci. 2015;16:18642–63. 26266408 10.3390/ijms160818642 PMC4581264

[8] Davidson KT, Zhu Z, Balabanov D et al. Beyond conventional medicine - a look at blueberry, a cancer-fighting superfruit. Pathol Oncol Res. 2018;24:733–8. 29285736 10.1007/s12253-017-0376-2

[9] Faostat F FAOSTAT statistical database 2016.

[10] Vorsa N, Johnson-Cicalese J American Cranberry. In: Badenes ML, Byrne DH(eds). Fruit Breeding. Boston, MA: Springer US; 2012 p. 191–223.

[11] Darrow GM, Others The strawberry. History, breeding and physiology. New York: Holt, Rinehart & Winston; 1966; https://www.cabdirect.org/cabdirect/abstract/19681601719, September 15th, 2018.

[12] Prior RL, Cao G, Martin A, et al. Antioxidant capacity as influenced by total phenolic and anthocyanin content, maturity, and variety of vaccinium species. J Agric Food Chem American Chemical Society. 1998;46:2686–93.

[13] Kim H, Bartley GE, Rimando AM et al. Hepatic gene expression related to lower plasma cholesterol in hamsters fed high-fat diets supplemented with blueberry peels and peel extract. J Agric Food Chem. 2010;58:3984–91. 20143813 10.1021/jf903230s

[14] Wang SY, Camp MJ, Ehlenfeldt MK Antioxidant capacity and α-glucosidase inhibitory activity in peel and flesh of blueberry (*Vaccinium* spp.) cultivars. Food Chem. 2012;132:1759–68.

[15] Faria A, Pestana D, Teixeira D et al. Blueberry anthocyanins and pyruvic acid adducts: anticancer properties in breast cancer cell lines. Phytother Res. 2010;24:1862–9. 20564502 10.1002/ptr.3213

[16] Hurst RD, Wells RW, Hurst SM, et al. Blueberry fruit polyphenolics suppress oxidative stress-induced skeletal muscle cell damage in vitro. Mol Nutr Food Res. 2010;54:353–63. 19885847 10.1002/mnfr.200900094

[17] Krikorian R, Shidler MD, Nash TA et al. Blueberry supplementation improves memory in older adults. J Agric Food Chem. 2010;58:3996–4000. 20047325 10.1021/jf9029332 PMC2850944

[18] Norberto S, Silva S, Meireles M, et al. Blueberry anthocyanins in health promotion: a metabolic overview. J Funct Foods. 2013;5:1518–28.

[19] Wang Y, Cheng M, Zhang B, et al. Dietary supplementation of blueberry juice enhances hepatic expression of metallothionein and attenuates liver fibrosis in rats. PLoS One. 2013;8:e58659. 23554912 10.1371/journal.pone.0058659 PMC3595269

[20] Stull A, Cash K, Champagne C et al. Blueberry bioactives improve endothelial function in adults with metabolic syndrome. FASEB J. . The Federation of American Societies for Experimental Biology 2015;29:http://www.fasebj.org/content/29/1_Supplement/923.17.short. 10.3390/nu7064107 PMC4488775 26024297

[21] Bell L, Lamport DJ, Butler LT et al. A study of glycaemic effects following acute anthocyanin-rich blueberry supplementation in healthy young adults. Food Funct. 2017;8:3104–10. 28752872 10.1039/c7fo00724h

[22] Gallardo RK, Stafne ET, DeVetter LW et al. Blueberry producers’ attitudes toward harvest mechanization for fresh market. Horttechnology. 2018;28:10–6.

[23] Gupta V, Estrada AD, Blakley I, et al. RNA-seq analysis and annotation of a draft blueberry genome assembly identifies candidate genes involved in fruit ripening, biosynthesis of bioactive compounds, and stage-specific alternative splicing. GigaScience. 2015;4:5. 25830017 10.1186/s13742-015-0046-9 PMC4379747

[24] Schnable JC, Springer NM, Freeling M Differentiation of the maize subgenomes by genome dominance and both ancient and ongoing gene loss. Proc Natl Acad Sci U S A. 2011;108:4069–74. 21368132 10.1073/pnas.1101368108 PMC3053962

[25] Bird KA, VanBuren R, Puzey JR et al. The causes and consequences of subgenome dominance in hybrids and recent polyploids. New Phytol. 2018;220:87–93. 29882360 10.1111/nph.15256

[26] Bottani S, Zabet NR, Wendel JF, et al. Gene expression dominance in allopolyploids: hypotheses and models. Trends Plant Sci. 2018;23:393–402. 29433919 10.1016/j.tplants.2018.01.002

[27] Campbell MS, Law M, Holt C, et al. MAKER-P: a tool kit for the rapid creation, management, and quality control of plant genome annotations. Plant Physiol. 2014;164:513–24. 24306534 10.1104/pp.113.230144 PMC3912085

[28] Arabidopsis Genome Initiative Analysis of the genome sequence of the flowering plant *Arabidopsis thaliana*. Nature. 2000;408:796–815. 11130711 10.1038/35048692

[29] Berardini TZ, Reiser L, Li D et al. The *Arabidopsis* information resource: making and mining the ‘gold standard’ annotated reference plant genome. Genesis. 2015;53:474–85. 26201819 10.1002/dvg.22877 PMC4545719

[30] Huang S, Ding J, Deng D, et al. Draft genome of the kiwifruit *Actinidia chinensis*. Nat Commun. 2013;4:2640. 24136039 10.1038/ncomms3640 PMC4089393

[31] Simão FA, Waterhouse RM, Ioannidis P et al. BUSCO: assessing genome assembly and annotation completeness with single-copy orthologs. Bioinformatics. 2015;31:3210–2. 26059717 10.1093/bioinformatics/btv351

[32] Götz S, García-Gómez JM, Terol J et al. High-throughput functional annotation and data mining with the Blast2GO suite. Nucleic Acids Res. 2008;36:3420–35. 18445632 10.1093/nar/gkn176 PMC2425479

[33] Kanehisa M, Goto S KEGG: Kyoto encyclopedia of genes and genomes. Nucleic Acids Res. 2000;28:27–30. 10592173 10.1093/nar/28.1.27 PMC102409

[34] Edger PP, VanBuren R, Colle M, et al. Single-molecule sequencing and optical mapping yields an improved genome of woodland strawberry (*Fragaria vesca*) with chromosome-scale contiguity. GigaScience. 2018;7:1–7. 10.1093/gigascience/gix124 PMC5801600 29253147

[35] VanBuren R, Bryant D, Bushakra JM, et al. The genome of black raspberry (*Rubus occidentalis*). Plant J. 2016;87:535–47. 27228578 10.1111/tpj.13215

[36] Canaguier A, Grimplet J, Di Gaspero G, et al. A new version of the grapevine reference genome assembly (12X.v2) and of its annotation (VCost.v3). Genom Data. 2017;14:56–62. 28971018 10.1016/j.gdata.2017.09.002 PMC5612791

[37] Law M, Childs KL, Campbell MS, et al. Automated update, revision, and quality control of the maize genome annotations using MAKER-P improves the B73 RefGen_v3 gene models and identifies new genes. Plant Physiol Am Soc Plant Biol. 2015;167:25–39. 10.1104/pp.114.245027 PMC4280997 25384563

[38] Lee S-I, Kim N-S Transposable elements and genome size variations in plants. Genomics Inform. 2014;12:87–97. 25317107 10.5808/GI.2014.12.3.87 PMC4196380

[39] Vicient CM, Casacuberta JM Impact of transposable elements on polyploid plant genomes. Ann Bot. 2017;120:195–207. 28854566 10.1093/aob/mcx078 PMC5737689

[40] Ou S, Jiang N LTR_retriever: a highly accurate and sensitive program for identification of long terminal repeat retrotransposons. Plant Physiol. 2018;176:1410–22. 29233850 10.1104/pp.17.01310 PMC5813529

[41] Coville FV Blueberry chromosomes. Science. 1927;66:565–6. 17820511 10.1126/science.66.1719.565

[42] Draper AD, Scott DH Inheritance of albino seedling in tetraploid highbush blueberry. J Am Soc Hortic Sci. 1971, 96, 791–792.; http://agris.fao.org/agris-search/search.do?recordID=US201302242589.

[43] Jelenkovic G, Hough LF Chromosome associations in the first meiotic division in three tetraploid clones of vaccinium corymbosum L. Can J Genet Cytol. 1970;12:316–24.

[44] Xiong Z, Gaeta RT, Pires JC Homoeologous shuffling and chromosome compensation maintain genome balance in resynthesized allopolyploid *Brassica napus*. Proc Natl Acad Sci U S A. 2011;108:7908–13. 21512129 10.1073/pnas.1014138108 PMC3093481

[45] Chester M, Gallagher JP, Symonds VV, et al. Extensive chromosomal variation in a recently formed natural allopolyploid species, *Tragopogon miscellus* (Asteraceae). Proc Natl Acad Sci U S A. 2012;109:1176–81. 22228301 10.1073/pnas.1112041109 PMC3268322

[46] Maere S, De Bodt S, Raes J, et al. Modeling gene and genome duplications in eukaryotes. Proc Natl Acad Sci U S A. 2005;102:5454–9. 15800040 10.1073/pnas.0501102102 PMC556253

[47] Kagale S, Robinson SJ, Nixon J, et al. Polyploid evolution of the Brassicaceae during the Cenozoic era. Plant Cell. 2014;26:2777–91. 25035408 10.1105/tpc.114.126391 PMC4145113

[48] Barker MS, Vogel H, Schranz ME Paleopolyploidy in the Brassicales: analyses of the Cleome transcriptome elucidate the history of genome duplications in *Arabidopsis* and other Brassicales. Genome Biol Evol. 2009;1:391–9. 20333207 10.1093/gbe/evp040 PMC2817432

[49] Doyle JJ, Egan AN Dating the origins of polyploidy events. New Phytol. 2010;186:73–85. 20028472 10.1111/j.1469-8137.2009.03118.x

[50] Ziolkowski PA, Koczyk G, Galganski L, et al. Genome sequence comparison of Col and Ler lines reveals the dynamic nature of *Arabidopsis* chromosomes. Nucleic Acids Res. 2009;37:3189–201. 19305000 10.1093/nar/gkp183 PMC2691826

[51] Ozkan H, Levy AA, Feldman M Rapid differentiation of homeologous chromosomes in newly-formed allopolyploid wheat. Isr J Plant Sci. 2002;50:65–76.

[52] VanBuren R, Wai CM, Ou S, et al. Extreme haplotype variation in the desiccation-tolerant clubmoss *Selaginella lepidophylla*. Nat Commun. 2018;9:13. 29296019 10.1038/s41467-017-02546-5 PMC5750206

[53] Ma J, Bennetzen JL Rapid recent growth and divergence of rice nuclear genomes. Proc Natl Acad Sci U S A. 2004;101:12404–10. 15240870 10.1073/pnas.0403715101 PMC515075

[54] Lancaster LT Molecular evolutionary rates predict both extinction and speciation in temperate angiosperm lineages. BMC Evol Biol. 2010;10:162. 20515493 10.1186/1471-2148-10-162 PMC2901258

[55] Thomas B Light signals and flowering. J Exp Bot. 2006;57:3387–93. 16980594 10.1093/jxb/erl071

[56] Grover CE, Gallagher JP, Szadkowski EP, et al. Homoeolog expression bias and expression level dominance in allopolyploids. New Phytol. 2012;196:966–71. 23033870 10.1111/j.1469-8137.2012.04365.x

[57] Woodhouse MR, Cheng F, Pires JC, et al. Origin, inheritance, and gene regulatory consequences of genome dominance in polyploids. Proceedings of the National Academy of Sciences National Acad Sciences. 2014;111:5283–8. 10.1073/pnas.1402475111 PMC3986174 24706847

[58] Edger PP, McKain MR, Bird KA et al. Subgenome assignment in allopolyploids: challenges and future directions. Curr Opin Plant Biol. 2018;42:76–80. 29649616 10.1016/j.pbi.2018.03.006

[59] Freeling M, Woodhouse MR, Subramaniam S et al. Fractionation mutagenesis and similar consequences of mechanisms removing dispensable or less-expressed DNA in plants. Curr Opin Plant Biol. 2012;15:131–9. 22341793 10.1016/j.pbi.2012.01.015

[60] Cheng F, Wu J, Cai X, et al. Gene retention, fractionation and subgenome differences in polyploid plants. Nature Plants. 2018;4:258–68. 29725103 10.1038/s41477-018-0136-7

[61] Garsmeur O, Schnable JC, Almeida A, et al. Two evolutionarily distinct classes of paleopolyploidy. Mol Biol Evol. 2013;31:448–54. 24296661 10.1093/molbev/mst230

[62] Zhao M, Zhang B, Lisch D et al. Patterns and consequences of subgenome differentiation provide insights into the nature of paleopolyploidy in plants. Plant Cell. 2017;29:2974–94. 29180596 10.1105/tpc.17.00595 PMC5757279

[63] Adams KL, Cronn R, Percifield R, et al. Genes duplicated by polyploidy show unequal contributions to the transcriptome and organ-specific reciprocal silencing. Proc Natl Acad Sci U S A. 2003;100:4649–54. 12665616 10.1073/pnas.0630618100 PMC153610

[64] Boatwright JL, McIntyre LM, Morse AM, et al. A robust methodology for assessing differential homeolog contributions to the transcriptomes of allopolyploids. Genetics. 2018;210:883–94. 30213855 10.1534/genetics.118.301564 PMC6218233

[65] CoGe – a platform for comparative genomics; https://genomevolution.org/r/12w9o.; Last accessed February 18th, 2019.

[66] Bohner J, Bangerth F Cell number, cell size and hormone levels in semi-isogenic mutants of *Lycopersicon pimpinellifolium* differing in fruit size. Physiol Plant. 1988;72:316–20.

[67] Gillaspy G, Ben-David H, Gruissem W Fruits: a developmental perspective. Plant Cell. 1993;5:1439–51. 12271039 10.1105/tpc.5.10.1439 PMC160374

[68] Zifkin M, Jin A, Ozga JA, et al. Gene expression and metabolite profiling of developing highbush blueberry fruit indicates transcriptional regulation of flavonoid metabolism and activation of abscisic acid metabolism. Plant Physiol. 2012;158:200–24. 22086422 10.1104/pp.111.180950 PMC3252089

[69] Mainland CM, Tucker JW Blueberry health information - some new mostly review. Acta Hortic. 2002;39–43.12882223

[70] Gillespie KM, Chae JM, Ainsworth EA Rapid measurement of total antioxidant capacity in plants. Nat Protoc. 2007;2:867–70. 17446887 10.1038/nprot.2007.100

[71] Connor AM, Luby JJ, Tong CBS et al. Genotypic and environmental variation in antioxidant activity, total phenolic content, and anthocyanin content among blueberry cultivars. J Am Soc Hortic Sci. 2002;127:89–97.

[72] Wang H, Guo X, Hu X, et al. Comparison of phytochemical profiles, antioxidant and cellular antioxidant activities of different varieties of blueberry (*Vaccinium* spp.). Food Chem. 2017;217:773–81. 27664697 10.1016/j.foodchem.2016.09.002

[73] Wu Y, Zhou Q, Chen X-Y et al. Comparison and screening of bioactive phenolic compounds in different blueberry cultivars: evaluation of anti-oxidation and α-glucosidase inhibition effect. Food Res Int. 2017;100:312–24. 28873693 10.1016/j.foodres.2017.07.004

[74] Kalt W, Ryan DAJ, Duy JC, et al. Interspecific variation in anthocyanins, phenolics, and antioxidant capacity among genotypes of highbush and lowbush blueberries (*Vaccinium* section *cyanococcus* spp.). J Agric Food Chem. 2001;49:4761–7. 11600018 10.1021/jf010653e

[75] Moyer RA, Hummer KE, Finn CE et al. Anthocyanins, phenolics, and antioxidant capacity in diverse small fruits: vaccinium, rubus, and ribes. J Agric Food Chem. 2002;50:519–25. 11804523 10.1021/jf011062r

[76] Castrejón ADR, Eichholz I, Rohn S, et al. Phenolic profile and antioxidant activity of highbush blueberry (*Vaccinium corymbosum* L.) during fruit maturation and ripening. Food Chem. 2008;109:564–72.

[77] Wang SY, Lin HS Antioxidant activity in fruits and leaves of blackberry, raspberry, and strawberry varies with cultivar and developmental stage. J Agric Food Chem. 2000;48:140–6. 10691606 10.1021/jf9908345

[78] Zheng W, Wang SY Oxygen radical absorbing capacity of phenolics in blueberries, cranberries, chokeberries, and lingonberries. J Agric Food Chem. 2003;51:502–9. 12517117 10.1021/jf020728u

[79] Clifford MN Chlorogenic Acids. In: Clarke RJ, Macrae R(eds). Coffee: Volume 1: Chemistry. Dordrecht: Springer Netherlands; 1985 p. 153–202.

[80] Rice-Evans CA, Miller NJ, Paganga G Structure-antioxidant activity relationships of flavonoids and phenolic acids. Free Radic Biol Med. 1996;20:933–56. 8743980 10.1016/0891-5849(95)02227-9

[81] Shi H, Shi A, Dong L, et al. Chlorogenic acid protects against liver fibrosis in vivo and in vitro through inhibition of oxidative stress. Clin Nutr. 2016;35:1366–73. 27017478 10.1016/j.clnu.2016.03.002

[82] Hollman PC Evidence for health benefits of plant phenols: local or systemic effects?. J Sci Food Agric. 2001;81:842–52.

[83] Olthof MR, Hollman PC, Zock PL, et al. Consumption of high doses of chlorogenic acid, present in coffee, or of black tea increases plasma total homocysteine concentrations in humans. Am J Clin Nutr. 2001;73:532–8. 11237928 10.1093/ajcn/73.3.532

[84] Charurin P, Ames JM, del Castillo MD Antioxidant activity of coffee model systems. J Agric Food Chem. 2002;50:3751–6. 12059154 10.1021/jf011703i

[85] Yen W-J, Wang B-S, Chang L-W et al. Antioxidant properties of roasted coffee residues. J Agric Food Chem. 2005;53:2658–63. 15796608 10.1021/jf0402429

[86] Watanabe T, Arai Y, Mitsui Y, et al. The blood pressure-lowering effect and safety of chlorogenic acid from green coffee bean extract in essential hypertension. Clin Exp Hypertens. 2006;28:439–49. 16820341 10.1080/10641960600798655

[87] Falcone Ferreyra ML, Rius SP, Casati P Flavonoids: biosynthesis, biological functions, and biotechnological applications. Front Plant Sci. 2012;3:222. 23060891 10.3389/fpls.2012.00222 PMC3460232

[88] Zhang Y Regulation of ascorbate synthesis in plants. In: Zhang Y(ed). Ascorbic Acid in Plants: Biosynthesis, Regulation and Enhancement. New York, NY: Springer New York; 2013 p. 87–99.

[89] Laing W, Norling C, Brewster D, et al. Ascorbate concentration in*Arabidopsis thaliana* and expression of ascorbate related genes using rnaseq in response to light and the diurnal cycle [Internet]. bioRxiv. 2017; [cited 2018 Apr 21]. p. 138008https://www.biorxiv.org/content/early/2017/05/15/138008.abstract.

[90] Liu J, Osbourn A, Ma P MYB transcription factors as regulators of phenylpropanoid metabolism in plants. Mol Plant. 2015;8:689–708. 25840349 10.1016/j.molp.2015.03.012

[91] Petroni K, Tonelli C Recent advances on the regulation of anthocyanin synthesis in reproductive organs. Plant Sci. 2011;181:219–29. 21763532 10.1016/j.plantsci.2011.05.009

[92] Albert NW, Davies KM, Lewis DH et al. A conserved network of transcriptional activators and repressors regulates anthocyanin pigmentation in eudicots. Plant Cell. 2014;26:962–80. 24642943 10.1105/tpc.113.122069 PMC4001404

[93] Huang W, Khaldun ABM, Chen J, et al. A R2R3-MYB transcription factor regulates the flavonol biosynthetic pathway in a traditional chinese medicinal plant, epimedium sagittatum. Front Plant Sci. 2016;7:1089. 27493658 10.3389/fpls.2016.01089 PMC4954812

[94] Nguyen NH, Lee H MYB-related transcription factors function as regulators of the circadian clock and anthocyanin biosynthesis in *Arabidopsis*. Plant Signal Behav. 2016;11:e1139278. 26905954 10.1080/15592324.2016.1139278 PMC4883932

[95] Jin J, Tian F, Yang D-C et al. PlantTFDB 4.0: toward a central hub for transcription factors and regulatory interactions in plants. Nucleic Acids Res. 2017;45:D1040–5. 27924042 10.1093/nar/gkw982 PMC5210657

[96] Kautsar SA, Suarez Duran HG, Blin K et al. plantiSMASH: automated identification, annotation and expression analysis of plant biosynthetic gene clusters. Nucleic Acids Res. 2017;45:W55–63. 28453650 10.1093/nar/gkx305 PMC5570173

[97] Xi W, Zheng H, Zhang Q et al. Profiling taste and aroma compound metabolism during apricot fruit development and ripening. Int J Mol Sci. 2016;17:998. 10.3390/ijms17070998 PMC4964374 27347931

[98] Du X, Rouseff R Aroma active volatiles in four southern highbush blueberry cultivars determined by gas chromatography–olfactometry (GC-O) and gas chromatography–mass spectrometry (GC-MS). J Agric Food Chem. 2014;62:4537–43. 24758568 10.1021/jf500315t

[99] Farneti B, Khomenko I, Grisenti M, et al. Exploring blueberry aroma complexity by chromatographic and direct-injection spectrometric techniques. Front Plant Sci. 2017;8:617. 28491071 10.3389/fpls.2017.00617 PMC5405137

[100] Beaulieu JC, Stein-Chisholm RE, Boykin DL Qualitative analysis of volatiles in rabbiteye blueberry cultivars at various maturities using rapid solid-phase microextraction. J Am Soc Hortic Sci. 2014;139:167–77.

[101] Du X, Whitaker V, Rouseff R Changes in strawberry volatile sulfur compounds due to genotype, fruit maturity and sample preparation. Flavour Fragr J. 2012;27:398–404.

[102] Du X, Plotto A, Song M et al. Volatile composition of four southern highbush blueberry cultivars and effect of growing location and harvest date. J Agric Food Chem. 2011;59:8347–57. 21721574 10.1021/jf201184m

[103] Hirvi T, Honkanen E The aroma of blueberries. J Sci Food Agric. 1983;34:992–6.

[104] Horvat RJ, Senter SD Comparison of the volatile constituents from rabbiteye blueberries (*Vaccinium ashei*) during ripening. J Food Sci. 1985;50:429–31.

[105] Gilbert JL, Olmstead JW, Colquhoun TA, et al. Consumer-assisted selection of blueberry fruit quality traits. HortScience. 2014;49:864–73.

[106] Eom J-S, Chen L-Q, Sosso D et al. SWEETs, transporters for intracellular and intercellular sugar translocation. Curr Opin Plant Biol. 2015;25:53–62. 25988582 10.1016/j.pbi.2015.04.005

[107] Ren Y, Guo S, Zhang J, et al. A tonoplast sugar transporter underlies a sugar accumulation QTL in watermelon. Plant Physiol. 2018;176:836–50. 29118248 10.1104/pp.17.01290 PMC5761790

[108] Lin IW, Sosso D, Chen L-Q et al. Nectar secretion requires sucrose phosphate synthases and the sugar transporter SWEET9. Nature. 2014;508:546–9. 24670640 10.1038/nature13082

[109] Chen H-Y, Huh J-H, Yu Y-C, et al. The *Arabidopsis* vacuolar sugar transporter SWEET2 limits carbon sequestration from roots and restricts Pythium infection. Plant J. 2015;83:1046–58. 26234706 10.1111/tpj.12948

[110] Wormit A, Trentmann O, Feifer I, et al. Molecular identification and physiological characterization of a novel monosaccharide transporter from *Arabidopsis* involved in vacuolar sugar transport. Plant Cell. 2006;18:3476–90. 17158605 10.1105/tpc.106.047290 PMC1785410

[111] Sturm A, Tang GQ The sucrose-cleaving enzymes of plants are crucial for development, growth and carbon partitioning. Trends Plant Sci. 1999;4:401–7. 10498964 10.1016/s1360-1385(99)01470-3

[112] Qin G, Zhu Z, Wang W et al. A tomato vacuolar invertase inhibitor mediates sucrose metabolism and influences fruit ripening. Plant Physiol. 2016;172:1596–611. 27694342 10.1104/pp.16.01269 PMC5100769

[113] Achaz G, Coissac E, Viari A et al. Analysis of intrachromosomal duplications in yeast *Saccharomyces cerevisiae*: a possible model for their origin. Mol Biol Evol. 2000;17:1268–75. 10908647 10.1093/oxfordjournals.molbev.a026410

[114] Leister D Tandem and segmental gene duplication and recombination in the evolution of plant disease resistance genes. Trends Genet. 2004;20:116–22. 15049302 10.1016/j.tig.2004.01.007

[115] Chae L, Kim T, Nilo-Poyanco R et al. Genomic signatures of specialized metabolism in plants. Science. 2014;344:510–3. 24786077 10.1126/science.1252076

[116] Edger PP, Pires JC Gene and genome duplications: the impact of dosage-sensitivity on the fate of nuclear genes. Chromosome Res. 2009;17:699–717. 19802709 10.1007/s10577-009-9055-9

[117] Freeling M Bias in plant gene content following different sorts of duplication: tandem, whole-genome, segmental, or by transposition. Annu Rev Plant Biol. 2009;60:433–53. 19575588 10.1146/annurev.arplant.043008.092122

[118] Kliebenstein DJ, Lambrix VM, Reichelt M et al. Gene duplication in the diversification of secondary metabolism: tandem 2-oxoglutarate-dependent dioxygenases control glucosinolate biosynthesis in *Arabidopsis*. Plant Cell. 2001;13:681–93. 11251105 10.1105/tpc.13.3.681 PMC135509

[119] Ober D Seeing double: gene duplication and diversification in plant secondary metabolism. Trends Plant Sci. 2005;10:444–9. 16054418 10.1016/j.tplants.2005.07.007

[120] Hofberger JA, Lyons E, Edger PP, et al. Whole genome and tandem duplicate retention facilitated glucosinolate pathway diversification in the mustard family. Genome Biol Evol. 2013;5:2155–73. 24171911 10.1093/gbe/evt162 PMC3845643

[121] Conant GC, Wolfe KH Turning a hobby into a job: how duplicated genes find new functions. Nat Rev Genet. 2008;9:938–50. 19015656 10.1038/nrg2482

[122] Bekaert M, Edger PP, Pires JC, et al. Two-phase resolution of polyploidy in the *Arabidopsis* metabolic network gives rise to relative and absolute dosage constraints. Plant Cell. 2011;23:1719–28. 21540436 10.1105/tpc.110.081281 PMC3123947

[123] Rizzon C, Ponger L, Gaut BS Striking similarities in the genomic distribution of tandemly arrayed genes in *Arabidopsis* and rice. PLoS Comput Biol. 2006;2:e115. 16948529 10.1371/journal.pcbi.0020115 PMC1557586

[124] Kliebenstein DJ A role for gene duplication and natural variation of gene expression in the evolution of metabolism. PLoS One. 2008;3:e1838. 18350173 10.1371/journal.pone.0001838 PMC2263126

[125] Eckardt NA Genome dominance and interaction at the gene expression level in allohexaploid wheat. Plant Cell. 2014;26:1834. 24838977 10.1105/tpc.114.127183 PMC4079349

[126] Li A, Liu D, Wu J, et al. mRNA and small RNA transcriptomes reveal insights into dynamic homoeolog regulation of allopolyploid heterosis in nascent hexaploid wheat. Plant Cell. 2014;26:1878–900. 24838975 10.1105/tpc.114.124388 PMC4079356

[127] Pfeifer M, Kugler KG, Sandve SR et al. Genome interplay in the grain transcriptome of hexaploid bread wheat. Science. 2014;345:1250091. 25035498 10.1126/science.1250091

[128] International Wheat Genome Sequencing Consortium (IWGSC), IWGSC RefSeq principal investigators:, Appels R, Eversole K, Feuillet C et al. Shifting the limits in wheat research and breeding using a fully annotated reference genome. Science. 2018;361: pii: eaar7191. 10.1126/science.aar7191 30115783

[129] Zhang H-B, Zhao X, Ding X, et al. Preparation of megabase-size DNA from plant nuclei. Plant J. 1995;7:175–84.

[130] VanBuren R, Bryant D, Edger PP et al. Single-molecule sequencing of the desiccation-tolerant grass *Oropetium thomaeum*. Nature. 2015;527:508–U209. 26560029 10.1038/nature15714

[131] Avni R, Nave M, Barad O et al. Wild emmer genome architecture and diversity elucidate wheat evolution and domestication. Science. 2017;357:93–7. 28684525 10.1126/science.aan0032

[132] Luo M-C, Gu YQ, Puiu D et al. Genome sequence of the progenitor of the wheat D genome *Aegilops tauschii*. Nature. 2017;551:498–502. 29143815 10.1038/nature24486 PMC7416625

[133] Lieberman-Aiden E, van Berkum NL, Williams L et al. Comprehensive mapping of long-range interactions reveals folding principles of the human genome. Science. 2009;326:289–93. 19815776 10.1126/science.1181369 PMC2858594

[134] Putnam NH, O'Connell BL, Stites JC, et al. Chromosome-scale shotgun assembly using an in vitro method for long-range linkage. Genome Res. 2016;26:342–50. 26848124 10.1101/gr.193474.115 PMC4772016

[135] Scalable Nucleotide Alignment Program; http://snap.cs.berkeley.edu; Last accessed February 18th, 2019.

[136] Pertea M, Pertea GM, Antonescu CM et al. StringTie enables improved reconstruction of a transcriptome from RNA-seq reads. Nat Biotechnol. 2015;33:290–5. 25690850 10.1038/nbt.3122 PMC4643835

[137] Jurka J, Kapitonov VV, Pavlicek A, et al. Repbase Update, a database of eukaryotic repetitive elements. Cytogenet Genome Res. 2005;110:462–7. 16093699 10.1159/000084979

[138] Smit AFA, Hubley R, Green P RepeatMasker 1996. http://repeatmasker.org

[139] Korf I Gene finding in novel genomes. BMC Bioinformatics. 2004;5:59. 15144565 10.1186/1471-2105-5-59 PMC421630

[140] Stanke M, Waack S Gene prediction with a hidden Markov model and a new intron submodel. Bioinformatics. 2003;19(Suppl 2):ii215–25. 14534192 10.1093/bioinformatics/btg1080

[141] Jones P, Binns D, Chang H-Y, et al. InterProScan 5: genome-scale protein function classification. Bioinformatics. 2014;30:1236–40. 24451626 10.1093/bioinformatics/btu031 PMC3998142

[142] Ellinghaus D, Kurtz S, Willhoeft U LTRharvest, an efficient and flexible software for de novo detection of LTR retrotransposons. BMC Bioinformatics. 2008;9:18. 18194517 10.1186/1471-2105-9-18 PMC2253517

[143] Xu Z, Wang H LTR_FINDER: an efficient tool for the prediction of full-length LTR retrotransposons. Nucleic Acids Res. 2007;35:W265–8. 17485477 10.1093/nar/gkm286 PMC1933203

[144] Han Y, Wessler SR MITE-Hunter: a program for discovering miniature inverted-repeat transposable elements from genomic sequences. Nucleic Acids Res. 2010;38:e199. 20880995 10.1093/nar/gkq862 PMC3001096

[145] Smit A, Hubley R RepeatModeler Open-1.0. http://www.repeatmasker.org/RepeatModeler/2008; accessed February 18, 2019.

[146] Ou S LTR_retriever [Internet]. Github; https://github.com/oushujun/LTR_retriever;accessed February 18, 2019.

[147] Bolger AM, Lohse M, Usadel B Trimmomatic: a flexible trimmer for Illumina sequence data. Bioinformatics. 2014;30:2114–20. 24695404 10.1093/bioinformatics/btu170 PMC4103590

[148] FastX Toolkit; http://hannonlab.cshl.edu/fastx_toolkit/index.html; accessed February 18, 2019.

[149] FastQC; http://www.bioinformatics.bbsrc.ac.uk/projects/fastqc; accessed February 18, 2019

[150] Dobin A, Gingeras TR Mapping RNA-seq Reads with STAR. Curr Protoc Bioinformatics. 2015;51:11.14.1–19. 26334920 10.1002/0471250953.bi1114s51 PMC4631051

[151] Anders S, Pyl PT, Huber W HTSeq–a Python framework to work with high-throughput sequencing data. Bioinformatics. 2015;31:166–9. 25260700 10.1093/bioinformatics/btu638 PMC4287950

[152] Love MI, Huber W, Anders S Moderated estimation of fold change and dispersion for RNA-seq data with DESeq2. Genome Biol. 2014;15:550. 25516281 10.1186/s13059-014-0550-8 PMC4302049

[153] Obayashi T, Kinoshita K Rank of correlation coefficient as a comparable measure for biological significance of gene coexpression. DNA Res. 2009;16:249–60. 19767600 10.1093/dnares/dsp016 PMC2762411

[154] Obayashi T, Aoki Y, Tadaka S, et al. ATTED-II in 2018: a plant coexpression database based on investigation of the statistical property of the mutual rank index. Plant Cell Physiol. 2018;59:440. 29340653 10.1093/pcp/pcx209 PMC5914413

[155] The Purdue University Research Repository (PURR) https://purr.purdue.edu/projects/blueberrygenome accessed February 18, 2019.

[156] Wisecaver JH, Borowsky AT, Tzin V, et al. A global Co-expression network approach for connecting genes to specialized metabolic pathways in plants. Plant Cell. 2017;29:944–59. 28408660 10.1105/tpc.17.00009 PMC5466033

[157] Nepusz T, Yu H, Paccanaro A Detecting overlapping protein complexes in protein-protein interaction networks. Nat Methods. 2012;9:471–2. 22426491 10.1038/nmeth.1938 PMC3543700

[158] Lyons E, Pedersen B, Kane J, et al. The value of nonmodel genomes and an example using SynMap within CoGe to dissect the hexaploidy that predates the rosids. Trop Plant Biol. 2008;1:181–90.

[159] Camacho C, Coulouris G, Avagyan V et al. BLAST+: architecture and applications. BMC Bioinformatics. 2009;10:421. 20003500 10.1186/1471-2105-10-421 PMC2803857

[160] Colle M, Leisner C, Wai CM, et al. Supporting data for “Haplotype-phased genome and evolution of phytonutrient pathways of tetraploid blueberry”. GigaScience Database. 2019 10.5524/100537. PMC6423372 30715294

[161] CoGe – a platform for comparative genomics. https://genomevolution.org/coge/GenomeInfo.pl?gid=36464, accessed February 18, 2019.

