# Supplementary material for: Haplotype-phased genome and evolution of phytonutrient pathways of tetraploid blueberry
Source: Gigascience. 2019 Jan 31;8(3):giz012. doi: 10.1093/gigascience/giz012 (PMC6423372; doi:10.1093/gigascience/giz012)
Supplement: GIGA-D-18-00370_Original_Submission.pdf [file giz012_giga-d-18-00370_original_submission.pdf]

GigaScience

Subgenome dominance and evolution of phytonutrient pathways in allopolyploid blueberry

--Manuscript Draft--

**Manuscript Number:**	GIGA-D-18-00370
**Full Title:**	Subgenome dominance and evolution of phytonutrient pathways in allopolyploid blueberry
**Article Type:**	Research
**Funding Information:**	National Institute of Food and Agriculture (1015241) — Dr Patrick Edger<br>National Institute of Food and Agriculture (1009804) — Dr Patrick Edger<br>National Institute of Food and Agriculture (1016057) — Dr Jennifer Wisecaver
**Abstract:**	Highbush blueberry (Vaccinium corymbosum) has long been consumed for its unique flavor and composition of health-promoting phytonutrients, however, breeding efforts to improve fruit quality in blueberry have been greatly hampered by the lack of adequate genomic resources. The genome of highbush blueberry has been particularly challenging to assemble in large part to its polyploid nature and genome size. Here, we present a chromosome-scale and haplotype-phased genome assembly of the cultivar Draper, which has the highest antioxidant levels among a diversity panel of seventy-one cultivars and thirteen wild Vaccinium species. We leveraged this genome, combined with gene expression and metabolite data measured across fruit development, to identify genes involved in the biosynthesis of important phytonutrients among other metabolites associated with superior fruit quality. Genome-wide analyses revealed that both polyploidy and tandem gene duplications modified various pathways involved in the biosynthesis of key phytonutrients. Furthermore, analyses revealed the presence of a spatial-temporal specific dominantly expressed subgenome including during fruit development. These findings and the reference genome will serve as a valuable resource for future genome-enabled breeding of important agronomic traits in highbush blueberry.
**Corresponding Author:**	Patrick Edger<br>Michigan State University<br>UNITED STATES
**Corresponding Author Secondary Information:**	
**Corresponding Author's Institution:**	Michigan State University
**Corresponding Author's Secondary Institution:**	
**First Author:**	Marivi Colle
**First Author Secondary Information:**	
**Order of Authors:**	Marivi Colle
	Courtney Leisner
	Ching Man Wai
	Shujun Ou
	Kevin Bird
	Jie Wang
	Jennifer Wisecaver
	Alan Yocca
	Gil Ben-Zvi

Powered by Editorial Manager® and ProduXion Manager® from Aries Systems Corporation

	Elizabeth Alger
	Pete Callow
	Avital Brodt
	Kobi Baruch
	Kevin Childs
	Lily Shiue
	Guo-qing Song
	Anthony Schilmiller
	Nicholi Vorsa
	Robert VanBuren
	Robin Buell
	Ning Jiang
	Patrick Edger
	Thomas Swale
**Order of Authors Secondary Information:**	
**Additional Information:**	
**Question**	**Response**
Are you submitting this manuscript to a special series or article collection?	No
**Experimental design and statistics**<br><br>Full details of the experimental design and statistical methods used should be given in the Methods section, as detailed in our Minimum Standards Reporting Checklist. Information essential to interpreting the data presented should be made available in the figure legends.<br><br>Have you included all the information requested in your manuscript?	Yes
**Resources**<br><br>A description of all resources used, including antibodies, cell lines, animals and software tools, with enough information to allow them to be uniquely identified, should be included in the Methods section. Authors are strongly encouraged to cite Research Resource Identifiers (RRIDs) for antibodies, model organisms and tools, where possible.	Yes

Have you included the information requested as detailed in our Minimum Standards Reporting Checklist?	
**Availability of data and materials**<br><br>All datasets and code on which the conclusions of the paper rely must be either included in your submission or deposited in publicly available repositories (where available and ethically appropriate), referencing such data using a unique identifier in the references and in the “Availability of Data and Materials” section of your manuscript.<br><br>Have you have met the above requirement as detailed in our Minimum Standards Reporting Checklist?	Yes

Powered by Editorial Manager® and ProduXion Manager® from Aries Systems Corporation

Manuscript

Click here to download Manuscript BB_Genome_Manuscript_GigaScience.pdf

Subgenome dominance and evolution of phytonutrient pathways in allopolyploid blueberry

Authors: Marivi Colle[a], Courtney P. Leisner[b], Ching Man Wai[a], Shujun Ou[a,c], Kevin A. Bird[a,c], Jie Wang[b], Jennifer H. Wisecaver[d,e], Alan E. Yocca[a], Elizabeth I. Alger[a], Pete Callow[a], Gil Ben-Zvi[f], Avital Brodt[f], Kobi Baruch[f], Thomas Swale[g], Lily Shiue[g], Guo-qing Song[a], Kevin L. Childs[b,h], Anthony Schilmiller[i], Nicholi Vorsa[j,k], C. Robin Buell[e,l], Robert VanBuren[a,l], Ning Jiang[a,c], and Patrick P. Edger[a,c,1]

a. Department of Horticulture, Michigan State University, East Lansing, MI, USA
b. Department of Plant Biology, Michigan State University, East Lansing, MI, USA
c. Ecology, Evolutionary Biology and Behavior, Michigan State University, East Lansing, MI, USA
d. Department of Biochemistry, Purdue University, West Lafayette, IN, USA
e. Purdue Center for Plant Biology, Purdue University, West Lafayette, IN, USA
f. NRGene, Ness Ziona, 7403648 Israel
g. Dovetail Genomics, Santa Cruz, CA, USA
h. Center for Genomics Enabled Plant Science, Michigan State University, East Lansing, MI, USA
i. Mass Spectrometry & Metabolomics Core Facility, Michigan State University, East Lansing, MI, USA
j. Department of Plant Biology, Rutgers University, New Brunswick, NJ, USA
k. Philip E. Marucci Center for Blueberry and Cranberry Research and Extension, Rutgers University, Chatsworth, NJ, USA
l. Plant Resilience Institute, Michigan State University, East Lansing, MI, USA

1. Author for correspondence: edgerpat@msu.edu

Abstract:

Highbush blueberry (*Vaccinium corymbosum*) has long been consumed for its unique flavor and composition of health-promoting phytonutrients, however, breeding efforts to improve fruit quality in blueberry have been greatly hampered by the lack of adequate genomic resources. The genome of highbush blueberry has been particularly challenging to assemble in large part to its polyploid nature and genome size. Here, we present a chromosome-scale and haplotype-phased genome assembly of the cultivar Draper, which has the highest antioxidant levels among a diversity panel of seventy-one cultivars and thirteen wild *Vaccinium* species. We leveraged this genome, combined with gene expression and metabolite data measured across fruit development, to identify genes involved in the biosynthesis of important phytonutrients among other metabolites associated with superior fruit quality. Genome-wide analyses revealed that both polyploidy and tandem gene duplications modified various pathways involved in the biosynthesis of key phytonutrients. Furthermore, analyses revealed the presence of a spatial-temporal specific dominantly expressed subgenome including during fruit development. These findings and the reference genome will serve as a valuable resource for future genome-enabled breeding of important agronomic traits in highbush blueberry.

Introduction:

Since domestication efforts began in the early 1900s [1], highbush blueberry (*Vaccinium corymbosum* L.) has rapidly become a high value fruit crop worldwide [2–4]. Highbush blueberry, compared to hundreds of closely related blueberry species (e.g. huckleberry, *V. ovatum* Pursh; bilberry, *V. myrtillus* L.; and sparkleberry, *V. arboreum* Marshall) in the Ericaceae [5,6], is widely cultivated due to its adaptation to temperate conditions, excellent fruit quality, yield and composition of phytonutrients [7]. As a result for the demand for fresh blueberries as a "superfruit" [8], highbush blueberry production has increased 600% during the past three decades and steadily grown to a multi-billion dollar industry [9]. In addition to its short domestication history, highbush blueberry is unique in being one of only three commercially valuable fruit crops, accompanied by cranberry (*V. macrocarpon* Ait.) [10] and the garden strawberry (*Fragaria x ananassa*) [11], with wild progenitor species native to North America.

Blueberries have a single epidermal layer which expresses a rich anthocyanin profile when 'ripe', is covered with a cuticle, and in most commercial varieties is also covered with epicuticular wax, which in combination with anthocyanins, generates its characteristic 'powdery blue' color. The cuticular and epidermal layers contain nearly all of the phytonutrients in the fruit such as anthocyanins, proanthocyanidins and flavonols [12–14]. Previous studies on blueberry demonstrated that these compounds have health-promoting properties due to their role in controlling diabetes, preventing muscle damage caused by oxidative stress and cardiovascular disease, improving visual and brain functions, as well as inhibiting tumor growth and preventing the pathogenesis of other chronic diseases [15–21].

With the growing awareness of the potential health benefits of blueberry and increasing consumer demand, a primary goal of the blueberry research community is to develop cultivars with improved antioxidant levels along with other important fruit quality traits (e.g. aroma, taste, and firmness) [22]. However, in spite of its economic importance and health benefit potential, breeding efforts to improve fruit quality traits in blueberry have been slow due in large part to the lack of genetic information and genomic resources. Previous studies developed genetic and genomic resources for a wild diploid species ($2n$=$2x$=24) of blueberry [23]. However, this draft genome sequence has a large number of scaffolds (13,757 total; N50 of ~145kb), high percentage of gaps (~27.35%) in ~393.16Mb assembly, and most importantly, does not reflect the genome complexity of the economically important and cultivated tetraploid ($2n$=$4x$=48) highbush blueberry.

Here, we present the first chromosome-scale genome assembly of a tetraploid highbush blueberry. The haplotype-phased assembly consists of 48 pseudomolecules with ~1.68Gb of assembled sequence, ~1.29% gaps, and an average of 32,140 protein coding genes per haplotype (128,559 total). We leveraged this genome to examine the origin of the polyploid event and gain insights into the underlying genetics of fruit development and biosynthesis of metabolites contributing to superior fruit quality. Furthermore, we examined gene expression patterns among the four haplotypes in highbush blueberry. This analysis uncovered, unlike in other allopolyploids with a single dominantly expressed subgenome, the presence of spatial-temporal specific dominantly expressed subgenomes. These findings and the reference genome will serve as a powerful platform to further investigate the underlying mechanisms of 'subgenome dominance', further facilitate the discovery and analysis of genes encoding economically important traits and will enable molecular breeding for superior blueberry cultivars.

Results

Assembly and annotation of the tetraploid highbush blueberry genome

Our goal was to obtain a high-quality reference genome for the highbush blueberry cultivar 'Draper', which is widely grown around the world due to its excellent fruit quality. We sequenced the genome using a combination of both 10X Genomics (Pleasanton, CA) and Illumina (San Diego, CA), totalling 324X coverage of the genome (Table S1). These data were assembled and scaffolded using the software package DenovoMAGIC3 (NRGene, Nes Ziona, Israel)(Table S2), which was recently used to assemble the allotetraploid wheat (*Triticum turgidum*) genome [24]. The genome was further scaffolded to chromosome-scale using Hi-C data (91.4X coverage) in combination with the HiRise pipeline (Dovetail, Santa Cruz, CA)(Figure S1 and S2). The total length of the final assembly is 1,679,081,592 bases distributed across 48 chromosome-level pseudomolecules (**Figure 1**), and within our estimated genome size of the tetraploid (4x) based on flow cytometry (1.63Gb with 95% C.I. +/- 0.06Gb) (Extended Data Table 1).

The genome was annotated using a combination of evidence-based and *ab initio* gene prediction using MAKER-P pipeline [25] (Table S3). RNAseq datasets from thirteen different blueberry gene expression libraries, representing unique organs, developmental stages, and treatments (Table S4), and available transcriptome and expressed sequence tags (EST) data of *V. corymbosum* in NCBI were used as transcript evidence. Protein sequences from *Arabidopsis thaliana* [26,27], *Actinidia chinensis* [28] and UniprotKB plant database were used as evidence for genome annotation. We predicted a total of 128,559 protein-coding genes. Benchmarking Universal Single-Copy Orthologs analysis (BUSCO v.2) [29] was performed to assess the completeness of the assembly and quality of the genome annotation. The annotated gene set contains 1,413 out of 1,440 (98%) BUSCO genes (Table S5). Functional annotation including those associated with metabolic processes was assigned using BLAST2GO [30] and to reference pathways in the KEGG database [31] (Figure S3). Comparative genomic analyses further assigned genes to 16,909 orthogroups shared by six phylogenetically diverse plant species including five eudicots (*A. chinensis* [28], *A. thaliana* [26,27], *Fragaria vesca* [32], *Rubus occidentalis* [33] and *Vitis vinifera* [34]), each with distinct fruit types, and *Zea mays* [35] as the monocot outgroup.

Transposable elements (TEs), both Class I and II, were identified and classified in the genome using the protocol described by Campbell et al. (2014) [25]. Overall, 44.3% of the blueberry genome is composed of TEs (Table S6). Consistent with previous reports [36,37], the most abundant Class I TEs were long terminal repeat retrotransposons (LTR-RTs), specifically the superfamily LTR/*Gypsy* followed by LTR/*Copia*, while for Class II transposons, the miniature inverted repeat (MITE) superfamily *hAT* was the most prevalent. The quality of the genome was further assessed by examining the assembly continuity of repeat space using the LTR Assembly Index (LAI) deployed in the LTR_retriever package (v1.8) [38]. The adjusted LAI score of our blueberry genome is 14 and based on the LAI classification, this score is within the range of "reference" quality (**Figure 1**). Estimation of the regional LAI in 3Mb sliding windows also showed that assembly continuity is uniform and of high-quality across the entire genome.

Assessment of the origin of tetraploid highbush blueberry

The origin of highbush blueberry from either a single (i.e. autopolyploid) or multiple diploid progenitor species (i.e. allopolyploid) is a long-standing question [39]. Previous reports have suggested that highbush blueberry may be an autotetraploid based on the segregation ratios of certain traits [40]. However, an analysis of chromosomes pairing among different cultivars revealed largely bivalent pairing during metaphase I [41], similar to patterns observed in known allopolyploids [42,43]. To gain further insights into the polyploid history of highbush blueberry, we calculated sequence similarity and synonymous substitution (*Ks;* silent mutation) rates between duplicate genes in homoeologous regions across the genome. The average sequence similarity is ~96.3% among syntenic homoeologous genes. The average *Ks* divergence between syntenic homoeologous genes is ~0.036 per synonymous site. This suggests that two distinct, but closely related, species likely contributed to the polyploid origin of highbush blueberry.

Furthermore, comparative genomics revealed that homoeologous regions are highly collinear, except a few notable chromosome level translocations (**Figure 1a)**. These translocations were manually inspected and verified with both the raw sequence and Hi-C data obtained in this study. Rapid changes among homoeologous chromosomes is known to occur in newly formed and ongoing in established allopolyploids [42–44]. We also assessed the level of similarity and content of LTR transposable elements among the four haplotypes. As the most prevalent transposable elements in plants, LTR-RTs undergo continual "bloat and purge" cycles within most plant genomes [45], resulting in a unique signature that may distinguish subgenomes in an allopolyploid. To examine the evolutionary history of LTR-RTs in the highbush blueberry genome, we determined the presence and calculated the mean sequence identity of LTR sequences among each of the four haplotypes (Figure S4). The data shows that LTRs proliferated independently in the subgenomes of each diploid progenitor species, following the divergence from their most common recent ancestor, but prior to polyploidy. Thus, this analysis revealed that the majority of more recent LTRs (>96% similarity) are subgenome specific in highbush blueberry, consistent with other analyses supporting a allopolyploid origin for this species.

After allopolyploidization, one of the parental genomes (i.e. subgenomes) often emerges with significantly greater gene content and a greater number of more highly expressed genes [46–49]. The emergence of a dominant subgenome in an allopolyploid is predicted to resolve genetic and epigenetic conflicts that may arise from the merger of divergent subgenomes into a single nucleus [50–52]. However, autopolyploids are not expected to face these challenges or exhibit subgenome dominance since all genomic copies were contributed by a single progenitor species [53]. This was recently supported by genome-wide analyses of a putative ancient autopolyploid genome (soybean; *Glycine max*) [54].

To explore this in highbush blueberry, we compared gene content and expression level patterns between homoeologous chromosomes (**Figure 2)**. While gene content levels were largely similar among homoeologous chromosomes, with a few notable exceptions (**Figure 2a**), gene expression levels were highest for one of the four chromosome copies in the majority (average 9.3 of 14) of gene expression libraries (112 of 168 comparisons, x2 test p-value < 0.001)(**Figure 2b**, Figure S5). Noteworthy, in the three fruit libraries, the most dominantly expressed homoeologous chromosome often became the least expressed among the four chromosomes (19 of 36 comparisons; x2 test p-value <0.01) or among the two lowest expressed copies (26 of 36 comparisons; x2 test p-value <0.01). The most dominantly expressed

chromosome copy remained the highest expressed in developing fruit for only two of the chromosomes (6 and 10), which are also the chromosomes with the most structural variation (**Figure 1a**).

Changes in transcript abundance during blueberry fruit development

The progression of fruit development in blueberry is marked with visible external and internal morphological changes such as change in size and color (Figure S6a). We profiled gene expression in fruit using RNAseq across seven developmental stages from the earliest stage (i.e. post-fertilization) through the final stage (i.e. ripe fruit) to identify genes differentially expressed during fruit development. Distinctive transitions in gene expression were observed between early fruit growth to start of color development and complete color change to ripened fruit. We found that the majority of genes upregulated during early fruit development were involved in phenylpropanoid biosynthesis, nitrogen metabolism, as well as cutin, suberin and wax biosynthesis (Table S7a). In contrast, genes involved in starch and sugar metabolism were highly expressed at the onset of and during fruit ripening (Table S7b). Moreover, principal component analysis (PCA) showed the first two components accounted for 84% of the variation and separated the developmental stages into three groups: (early developmental stages) petal fall and small green fruit; (middle developmental stages) expanding green and pink fruit; and (late developmental stages) complete fruit color change, unripe and ripe fruit (Figure S6a & Figure S7).

Genes associated with cell division, cell wall synthesis and transport were found to be expressed the highest during the earliest developmental stages (Extended Data Table 2), which is consistent with previous work on other fruit species [55,56]. In addition to genes regulating cell proliferation, defense response related genes were also highly upregulated during the earliest developmental stages. During the middle developmental stages, genes regulating cell expansion, seed development and secondary metabolite biosynthesis were highly expressed. During late developmental stages and as the berry transitions to ripening, late embryogenesis, transmembrane transport, defense, secondary metabolite biosynthesis and abscisic acid related genes were highly overrepresented. Blueberry is considered a climacteric fruit but unlike the ethylene-driven fruit ripening in other climacteric species, abscisic acid has been demonstrated to regulate fruit ripening in blueberry [57]. In summary, global gene expression patterns mirror the morphological and physiological changes observed during blueberry development (Figure S6a).

Antioxidant capacity in blueberry

The economic significance of blueberry is largely driven by fruit quality and nutritional value [7,18,58]. We investigated the level of total antioxidant capacity across a blueberry diversity panel and the abundance of secondary metabolites responsible for its antioxidant activity in developing fruit. A diversity panel, composed of 71 highbush blueberry cultivars and 13 wild *Vaccinium* species, was evaluated for total antioxidant capacity in mature fruit using the oxygen radical absorbance capacity (ORAC) assay [59]. Similar to previous reports [60–62], we observed a wide range in antioxidant capacity (~5 -95 nmol TE/mg FW) across cultivars, with 'Draper' having the highest levels of antioxidants (Figure S6b). The observed variation in antioxidants among highbush blueberry, consistent with our results, were previously shown not to correlate with fruit weight or size [63]. In another study, a correlation between fruit size and total anthocyanin levels was identified within select highbush blueberry cultivars but not across other *Vaccinium* species or blackberry [64]. This inconsistency is likely due to sample size differences between studies.

To further examine the antioxidant capacity in 'Draper' during fruit development, fruits from the seven aforementioned fruit developmental stages were assayed for antioxidant levels (Figure S6a). The highest level of antioxidant capacity was observed at the earliest 'petal fall' stage (537 nmol TE/mg FW) (Figure S8) after which, the level of antioxidants declined during the middle and late developmental stages. This is consistent with previous reports on the antioxidant activity in blueberry during fruit maturation [65] and similar to observations in blackberry and strawberry, wherein green fruit have the highest ORAC values [66]. The antioxidant capacity in blueberry is influenced by various metabolites including anthocyanins [12,64,67]. Using the same fruit development series, we quantified anthocyanin and flavonol aglycones in 'Draper' using liquid chromatography-mass spectrometry (LC-MS). Overall, as the fruit changed its exocarp color from pink to dark blue during ripening, delphinidine-type anthocyanins started to accumulate and were the most abundant compound in ripe fruit (181 peak area/IS/gDW) followed by cyanidin, malvidin and petunidin (Figure S6c). Flavonols were also detected in all developmental stages with quercetin glycoside the most abundant (88 peak area/IS/gDW), while myricetin glycoside and rutin were present at very low levels.

Blueberry also has high levels of phenolic acids and among phenolics, chlorogenic acid (CGA) was the most abundant. High levels of CGA was observed throughout fruit development with the highest accumulation detected in young fruits (Figure S6d). This correlates with the pattern of antioxidant capacity across different fruit stages suggesting that CGA is one of the major metabolites contributing to high ORAC values in young developing fruit. CGA is derived from caffeic acid and quinic acid and has vicinal hydroxyl groups that are associated with scavenging reactive oxygen species (ROS) [68–70]. The antioxidant properties of CGA have been associated with preventing chronic diseases [71–75].

Expression of antioxidant biosynthesis related genes

To examine the developmental regulation of the biosynthesis of metabolites that contribute to antioxidant capacity in blueberry, we identified homologs of previously characterized genes in other species involved in ascorbate, flavonols, chlorogenic acid, and anthocyanin biosynthesis (**Figure 3** & Extended Data Table 3) [57,76–78]. The key biosynthetic genes for these compounds showed a distinct developmental specific pattern of expression (**Figures 3c-e** & Figure S9). For example, genes involved in the conversion of leucoanthocyanidins to proanthocyanidins (e.g. *LAR* and *ANR*) are highly expressed in the earliest and middle developmental fruit stages but not in ripening fruit (**Figure 3c**; green triangle & Extended Data Table 4). Conversely, genes involved in the conversion of leucoanthocyanidins to anthocyanins (e.g. *ANS*, *UFGT* and *OMT*) were highly expressed in mature and ripe fruit but not during early fruit developmental stages (**Figure 3c**; red circle & Extended Data Table 4). Additionally, paralogs encoding the same anthocyanin pathway enzymes (e.g. FHT, OMT) and genes involved in vacuolar localization of proanthcyanidins (e.g. glutathione S-transferase and MRP-type) exhibit similar developmental stage-specific expression patterns. The expression of these biosynthetic genes is regulated by specific transcription factors [79]. For example, the transcription factor complex, MYB-bHLH-WD (MBW) regulates expression of anthocyanin biosynthetic genes in eudicots [80–83]. Using the Plant Transcription Factor Database v.4.0 [84], we identified homologs of transcription factors (TF) belonging to 55 gene families and members of some of these gene families were predicted to be involved in the developmental regulation of flavonoid biosynthesis during blueberry fruit growth (Extended Data Table

4), including *R2-R3-MYBs*, *R3-MYBs*, *bHLHs*, and *WDRs* (**Figure 3b,e**). These transcription factors also exhibit fruit development specific expression patterns.

In addition, we performed a gene co-expression network analysis to identify metamodules of genes that appear co-regulated during fruit development in blueberry, specifically genes that are associated with phytonutrient biosynthesis. Our analysis identified 1,988 metamodules of co-expressed genes, of which, 428 metamodules contained at least one of the 57 Pfam domains that have been previously categorized as associated with specialized metabolic pathways in plants [85]. Our analysis revealed that 142/428 metamodules were more highly expressed in developing fruit compared to other plant tissues. Some metamodules showed clear trends of being highly expressed during either early or late fruit development. For example, METAMOD00377 is expressed early in fruit development and contains homologs to known anthocyanin genes *OMT, HCT, PAL, HQT* as well as 31 homologs to known transcription factors. In contrast, METAMOD01221 is expressed late in fruit development and contains homologs of *HCT, TT19, UFGT, OMT* and contains 10 homologs to known transcription factors. Moreover, we also examined metamodules for genes associated with other biosynthetic pathways which impart unique blueberry fruit characteristics. We identified two metamodules where genes appear to be co-regulated. Metamodule METAMOD00377 contains Pfam domains associated with terpene, saccharide, and alkaloid specialized metabolism and METAMOD01221 which contains terpene and saccharide specialize metabolism. These metamodules contained genes that are differentially expressed during fruit development. Overall, the developmental-specific expression patterns of key biosynthetic genes and their putative transcriptional regulators emphasizes the tight regulation of production, conversion and transport of precursor compounds that lead to the accumulation of antioxidant-related metabolites in blueberry.

Fruit aroma and the role of terpenes

The coregulation of genes involved in the biosynthesis of terpenes and saccharides during early and late fruit development described above reflects a coordinated interplay between these metabolites during fruit growth. Both terpenes and sugars contribute to the characteristic flavor of ripened fruit [86]. In blueberry, in which fruit flavor is a major determinant of fruit quality, two components play a central role in flavor perception: taste, which is a balance of sweetness and acidity, and aroma. Blueberry aroma is a complex blend of volatiles which include aldehydes, esters, terpenes, ketones and alcohols [87,88]. Previous reports in blueberry showed that aroma profile varies across different blueberry ecotypes and cultivars [89–91]. For example, the aroma of highbush blueberry is primarily driven by terpene hydrocarbons (e.g. linalool, geraniol, hydroxycitronellol) and aldehydes (e.g. (E)-2-hexenal, (E)-2-hexenol, (Z)-3-hexenol) [87,92]. Both linalool and geraniol are associated with sweet floral flavor however, linalool was reported to have high aroma intensity thus imparting the characteristic blueberry flavor when combined with aldehydes[87].

Here we identified and examined the expression of terpene synthase involved in the synthesis of linalool. Four of the linalool synthase homologs in blueberry, are highly expressed during late fruit development (Extended Data Table 5). This pattern of expression coincides with previous reports of linalool accumulation in ripened blueberry fruit[88,92,93]. On the other hand, one homolog of linalool synthase, although it is expressed during fruit growth, it did not show a clear developmental pattern of gene expression. Investigating the underlying factors regulating these enzymes will facilitate genetic manipulations that may lead to the improvement of blueberry flavor.

Sugar transporters

Blueberry fruit quality (i.e. consumer preference) is also highly influenced by relative sugar levels [94]. During fruit ripening, sugar levels of the endocarp increases by importing hexose symplastically and/or apoplastically. Sugar transporters (i.e. *SWEET*), sucrose transporter (*SUT*) and tonoplast sugar transporter (*TST*) have been demonstrated to regulate intercellular sugar transport in phloem and fruit [95,96]. In *A. thaliana*, all clade III *SWEET* transporters play a role in sucrose transport with *AtSWEET9* primarily functioning in nectary secretion [97] while *AtSWEET15* is required for seed filling by acting with *SWEET11* and *SWEET12* [98]. In blueberry, the Clade III *SWEET transporters 9* and *10* were highly expressed during early fruit growth while clade III *SWEET transporter 15* was mainly expressed in ripe fruit (Extended Data Table 5). Interestingly, one of the blueberry *SWEET15* homologs showed a distinct pattern of expression compared to the other three homologs. To the best of our knowledge, we are the first to report on the potential role of these genes during blueberry fruit development.

In addition, homologs of *A. thaliana TST1* [99] and watermelon *ClTST1* and *ClTST3* (tonoplast sugar transporters) [96] were expressed during fruit ripening in blueberry. Elevated expression of a *ClTST1* homolog was observed throughout fruit development but the *ClTST3* homolog showed very low expression. Another gene that is highly expressed during fruit maturation is vacuolar invertase. As described in other systems [100], its upregulation during fruit ripening coincided with the breakdown of starch to sucrose or a mixture of glucose and fructose suggesting that it may be involved in the regulation of sugar accumulation in blueberry fruit. It was previously reported that vacuolar invertase modulates hexose to sucrose ratio in ripening fruit [101]. In addition, there are also two sugar transport protein (*STP*) homologs that exhibited developmental specific expression. However, their function remains largely unknown, thus, their potential role in sugar accumulation in the developing berry requires further investigation.

Expansion of antioxidant-related gene families through tandem duplication

Tandemly duplicated genes arise as a result of unequal crossing-over or template slippage during DNA repair [102,103], exhibit high birth-death rates (i.e. predominantly young) [104] and typically are in co-regulated clusters in the genome [105]. Smaller-scale duplications [106], which includes tandem duplicates, are highly biased towards certain gene families [107] including those involved in specialized metabolism in plants [108–110]. Furthermore, tandem duplications often results in the increased dosage of gene products [111] and may improve the metabolic flux of rate-limiting steps in certain biosynthetic pathways [112].

Most genes associated with the biosynthesis of antioxidants (CGA, flavonols, anthocyanins, proanthocyanidins) have at least one tandem duplicate present in the highbush blueberry genome, with tandem array sizes ranging between two to ten gene copies (Extended Data Table 6). The largest tandem arrays were found for *HQT* and *HCT* genes, which are co-regulated and involved in the CGA pathway (**Figure 3a)**. Differences in tandem array sizes was also observed between homoeologous chromosomes for various genes. For example, the *C3H* gene, which is involved in CGA biosynthesis (**Figure 3a**), was present on all four homoeologous chromosomes but with varying tandem array sizes. One of the homoeologous chromosomes had two copies of *C3H*, while the other three homoeologous chromosomes had four copies. This suggests that copy number differences of *C3H* among subgenomes may be due to either selection for gene duplication or loss, or in the case of allopolyploidy, may be due to preexisting gene content differences among the diploid progenitor species.

Genes in the anthocyanin pathway with other unique duplication patterns include *CHS*, *CHI*, *OMT*, and *UFGT*. The gene *CHS*, involved in the conversion of 4-Coumaryl-CoA to naringenin chalcone, has two copies and both have tandem duplicates in at least three of the homoeologous chromosomes. Interestingly, the gene *CHI*, has a single preserved tandem gene duplicate on only one of the homoeologous chromosomes. However, additional copies of *CHI* were also identified more distantly away from the syntenic ortholog on another homoeologous chromosome, likely involving a transposition event following tandem duplication. The *OMT* and *UFGT* genes all have tandem duplicates on all of the homoeologous chromosomes, although with varying array sizes, while the *ANR* gene involved in the conversion of anthocyanidin to proanthocyanidin is single copy on all homoeologous chromosomes. *DFR* gene, which is involved in the conversion of dihydroquercetin/dihyromyricetin to leucoanthocyanidin, has a single tandem duplicate on only one of the homoeologous chromosomes. These findings suggest that there may have been greater selective pressure to retain tandem duplicates for genes encoding enzymes involved in anthocyanin production than conversion to proanthocyanidins in ‘Draper’.

Discussion

Despite the economic importance of blueberry, molecular breeding approaches to produce superior cultivars has been greatly hampered by inadequate genomic resources and a limited understanding of the underlying genetics encoding key phytonutrients. This has resulted in breeders having to rely on traditional approaches to generate new cultivars; each with widely varying fruit quality characteristics including total antioxidant levels. For example, our analysis of a diversity panel consisting of eighty-four cultivars and wild species revealed that ‘Draper’ has antioxidant levels that are up to 19x higher than other cultivars. Thus, the ‘Draper’ genome is not only, to our knowledge, the first genome assembly of the cultivated highbush blueberry, but will serve as a powerful resource to guide future breeding efforts aimed at improving various fruit quality traits. Furthermore, this is the first chromosome-scale and haplotype-phased genome for any species in the order Ericales, which includes other high-value crops (e.g. tea, kiwifruit and cranberry) and wild species with unique life history traits (e.g. carnivorous, American pitcher plants; parasitic, *Sarcodes* ‘snow flower’ and extremophiles, ‘Jacob cactus’). We anticipate that this reference genome, plus the transcriptomic and metabolomic datasets, will also serve as a robust functional genomic platform for a wide variety of evolutionary studies.

Here, we leveraged this genomic platform to identify genes and pathways that encode superior fruit quality characteristics in blueberry, including those associated with pigmentation, sugar and antioxidant levels in ripe fruit. Our analyses revealed not only a tight regulation for the biosynthesis of various metabolites (e.g. anthocyanins) based on coordinated gene expression patterns, but also that genes in the same pathway are often co-localized as gene clusters in the genome. These metabolic gene clusters, which have been described in other plant genomes [113,114], were likely selected for improved co-regulation of interacting genes in the same biosynthetic pathways [115]. Furthermore, we found that genes encoding key biosynthetic steps in antioxidant pathways are enriched with small-scale gene duplications (e.g tandem duplications) in the ‘Draper’ genome. For example, tandem gene duplications have expanded gene families in blueberry that are involved in the biosynthesis of anthocyanins. This suggests that, in addition to a recent whole genome duplication, tandem duplications have greatly contributed to the

metabolic diversity observed in blueberry (as previously described in *Arabidopsis* [116]). These tandem duplicates may have evolved new functions (i.e. neofunctionalized), now involved in the biosynthesis of novel compounds, and/or were selected to improve the metabolic flux of specific biosynthetic steps that alter the dosage of certain endpoint metabolites [112]. Future studies are needed to further investigate the role of tandem duplications in contributing towards modified metabolite levels and composition in blueberry cultivars.

Our analyses also revealed that highbush blueberry, a tetraploid, likely arose from the hybridization of two distinct species (i.e. allopolyploidy) based on the sequence divergence, unique transposable element insertions and gene expression dominance observed between parental subgenomes. Subgenome dominance is predicted to resolve various genetic and epigenetic conflicts that may arise in the nucleus of an allopolyploid [117]. Two major hypotheses have been proposed to explain the 'subgenome dominance' patterns observed in allopolyploids. The first hypothesis [50,52,118] proposes that subgenome dominance is due to differences in the densities of transposable elements (TE) near homoeologous genes. Transposable elements are known to negatively impact expression of nearby genes [119,120]. Thus, the subgenome with the lowest TE densities near genes will be more highly expressed compared to the other subgenome(s) [49,51,117,121]. An expectation of this model would be the emergence of only a single dominantly expressed subgenome in all spatial and temporal contexts. A single subgenome being dominantly expressed in all surveyed organs and developmental stages has been previously reported for *Zea mays* (maize) [118], *Brassica rapa* (Chinese cabbage) [122] and *Mimulus peregrinus* (monkeyflower) [123] among other allopolyploids. No subgenome dominance would be observed if an autopolyploid (e.g. soybean [54]) or if the subgenomes in an allopolyploid have a similar TE content [117].

The second hypothesis [51] proposes that subgenome dominance is an outcome of needing to resolve regulatory mismatches between *trans* regulators (e.g. transcription factors) and their target *cis* regulatory sequences in an allopolyploid genome. These mismatches arise, in part, because each subgenome has a suite of regulators that have independently coevolved with their binding sites [124,125], distinct gain and losses of interactions with other regulators [126,127] and may have evolved altered dosages of each regulator with varied spatial-temporal patterns [51]. Relative dosages of many regulatory interactions needs to remain properly balanced to maintain complete cellular functions [128,129]. Unlike under the previous model, one of the possible outcomes could be the emergence of spatial-temporal specific dominant subgenomes [117]. In other words, each subgenome may control a unique set of pathways, cellular functions and/or developmental programs. However, similar to the previous model, subgenome dominance would not be predicted in an autopolyploid or allopolyploid derived from species with near identical *cis*/*trans* regulators. Our analyses revealed that the subgenomes in highbush blueberry are likely controlling a different set of genetic programs (e.g. fruit development vs mature leaves). The dominantly expressed subgenome in most surveyed tissues (e.g. flower buds) becomes the lowest expressed during fruit development. This observation is similar to findings in allopolyploid wheat where developmental and adaptive traits were shown to be controlled by different subgenomes [130–132]. For example, cell type- and stage-dependent subgenome expression dominance was observed in the developing wheat grain [132].

The two proposed hypotheses are not mutually exclusive. There is strong evidence that TE density differences direct which subgenome will be dominantly expressed in interspecific hybrids and

allopolyploids. In natural and resynthesized allopolyploids with notable subgenome differences in TE densities near homoeologous genes, subgenome expression dominance has been shown to establish in the first generation interspecific hybrid and increase over successive generations in the allopolyploid [123]. This would contribute towards rapidly fixing any *cis-trans* regulatory mismatches that may be present in these allopolyploids. However, in an allopolyploid with similar TE content among subgenomes (e.g. highbush blueberry or hexaploid wheat [133]), any *cis-trans* regulatory mismatches controlling important cellular functions would need to be repaired solely via other mechanisms. These mechanisms remain poorly understood but may involve subgenome specific changes in DNA methylation and histone modifications. We argue that both highbush blueberry and hexaploid wheat, each now with high quality reference genomes [133], make excellent systems to further investigate these underlying mechanisms that rapidly resolve *cis-trans* regulatory mismatches in interspecific hybrids and allopolyploids.

Subgenome differences in TE densities permit us to predict which homoeolog in an allopolyploid will be more dominantly expressed. In the absence of TE density differences, it remains entirely unknown if the dominantly expressed subgenomes in a particular organ and/or developmental stage is a random outcome. This has serious implications in guiding crop improvement efforts in certain allopolyploids. For example, marker assisted breeding needs to target the correct set of dominant homoeologs given the trait. Thus, we anticipate that this genome, combined with our insights into subgenome dominance, will greatly accelerate molecular breeding efforts in the cultivated highbush blueberry.

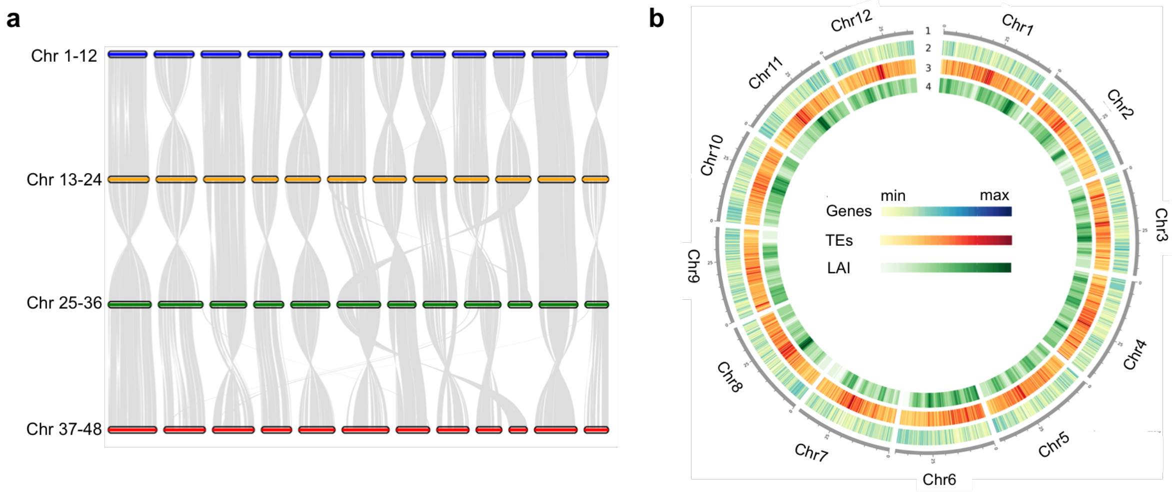

Figure 1. The haplotype-phased chromosome-scale highbush blueberry genome. (a) Collinearity among the homoeologous chromosomes. The gray lines represent conserved gene arrays between chromosomes. (b) Gene and transposable element (TE) density and LTR Assembly Index (LAI) in chromosomes 1-12 plotted in 300 Kb sliding window using Circos. The tracks from outside to inside are: 1= chromosomes, 2=gene density, 3=TE density, and 4= LAI score.

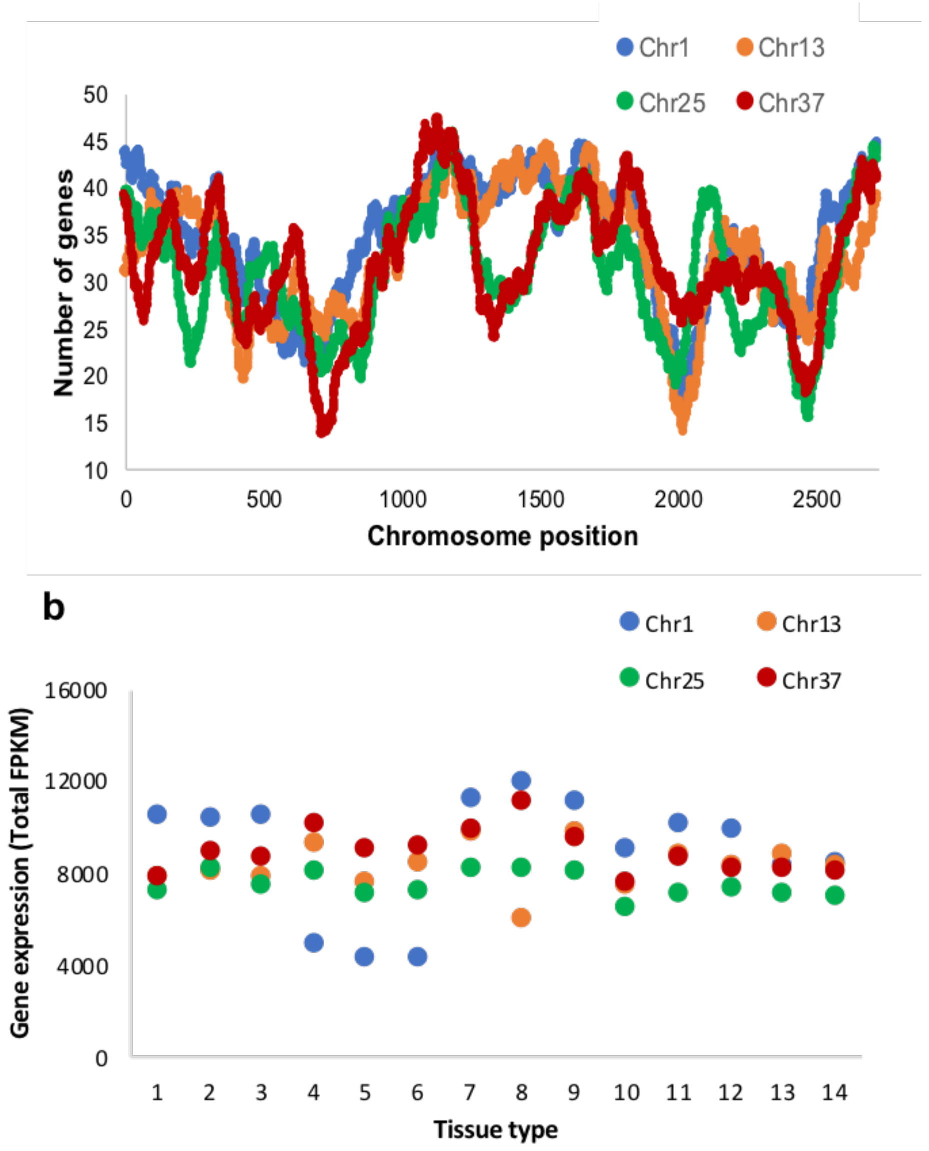

Figure 2. Assessment of the origin of polyploid blueberry. (a) Gene content comparison of homoeologous chromosomes (1, 13, 25, and 37) plotted along 2725 collinear syntenic regions. This analysis for all 48 chromosomes can be regenerated here: https://genomevolution.org/r/12w9o (b) Gene expression comparison (FPKM; fragments per kilobase per million) among the same four homoeologous chromosomes across different blueberry tissues (1=flower bud; 2=flower at anthesis; 3=petal fall; 4=green fruit; 5=pink fruit; 6=ripe fruit; 7, 8=leaf collected at 12 p.m. and 12 a.m., respectively; 9, 10, 11=methyl jasmonate treated leaf collected after one hour, eight hours and 24 hours, respectively; 12=shoot; 13=root; 14=salt-treated root).

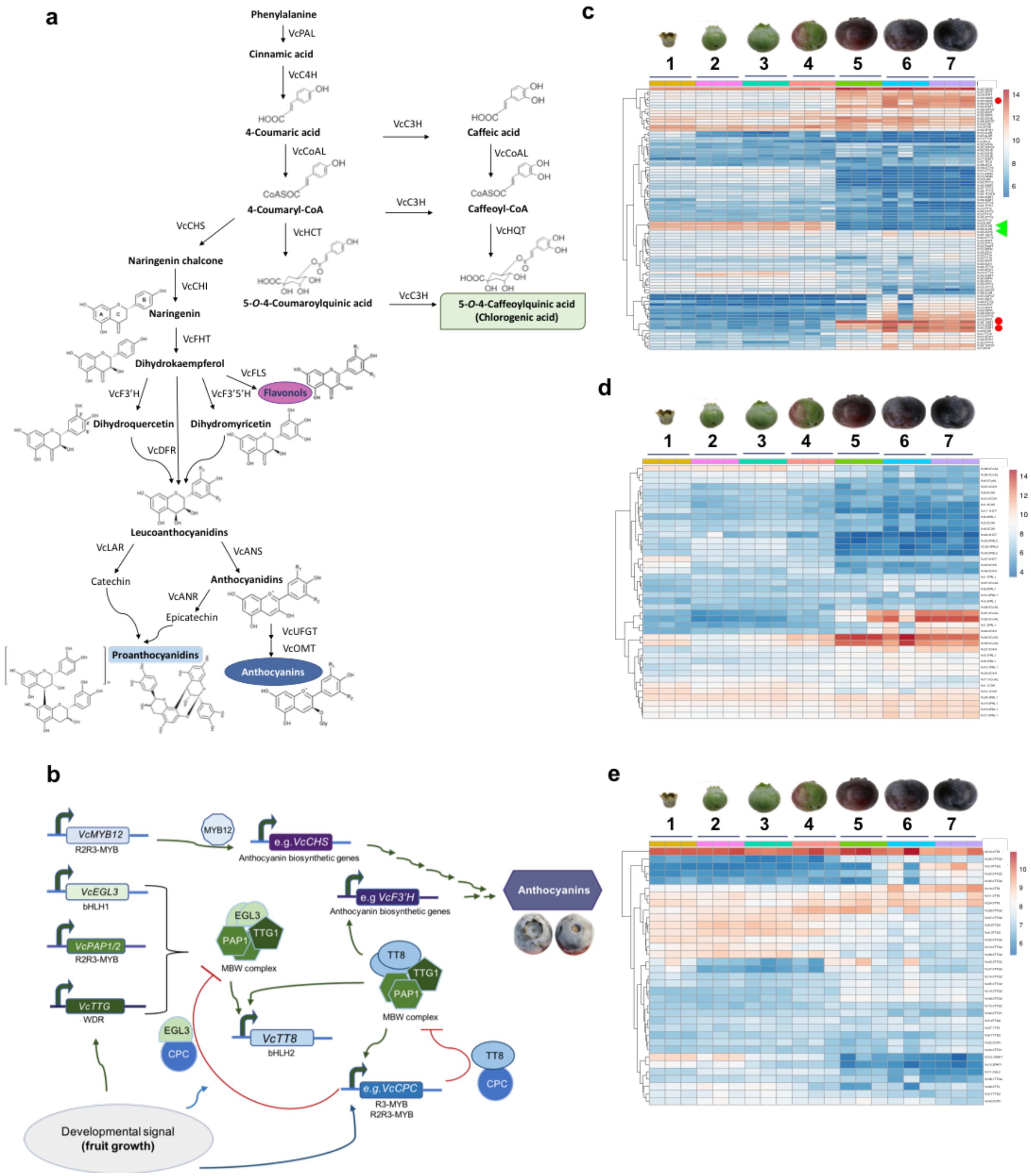

Figure 3. A schematic presentation of flavonoid biosynthesis in blueberry. (a) Predicted flavonoid biosynthetic pathway leading to production of anthocyanin. The proposed pathway is based on previously described flavonoid biosynthetic pathway in plants (Zifkin et al., 2012) and expression of predicted anthocyanin biosynthetic genes in blueberry. The core genes include phenylalanine ammonia-lyase (*PAL*), 4-hydroxycinnamoyl CoA ligase (*4CL*), trans-cinnamate 4-monooxygenase (*C4H*), cytochrome P450 98A3 (*C3H*), chalcone synthase (*CHS*), chalcone flavonone isomerase (*CHI*),

flavanone-3β-hydroxylase (*FHT*), flavanone 3-hydroxylase (*F3H*), flavonoid 3′-hydroxylase (*F3'H*), flavonoid 3′,5′-hydroxylase (*F3'5'H*), dihydroflavonol reductase (*DFR*), leucoanthocyanidin reductase (*LAR*), anthocyanidin synthase (*ANS*), UDP-glucose flavonoid 3-O-glucosyl transferase (*UFGT*), anthocyanin-O-methyltransferase (*OMT*), hydroxycinnamoyl-CoA shikimate/quinate hydroxycinnamoyltransferase (*HCT*) and hydroxycinnamoyl-CoA quinate hydroxycinnamoyltransferase (*HQT*) (b) Hypothetical regulatory pathway of anthocyanin biosynthetic genes based on the proposed model by Albert et al. (2014). (c) Developmental-specific expression pattern of key anthocyanin biosynthetic gene (green triangles = examples of genes upregulated during early fruit growth; red circles = examples of genes upregulated during late fruit development) and (d) chlorogenic acid biosynthetic genes (1=petal fall, 2= small green fruit, 3= expanding green fruit, 4=pink fruit, 5= fruit color completely changed from pink to purple, 6=unripe, 7=ripe) (e) Expression profile of transcription factors predicted to regulate anthocyanin biosynthesis in blueberry.

Materials and Methods

Plant Material

The cultivar ‘Draper’ was selected based on having the highest antioxidant levels among a diversity panel of leading cultivars and due to its overall importance to the industry (Figure S6). Furthermore, cultivar ‘Draper’ was selected since germplasm is widely available from blueberry nurseries. The genome size (1.63 +/- 0.06Gb) was estimated using flow cytometry with four technical replicates from Flow Cytometry Core at Benaroya Research Institute at Virginia Mason (Seattle, WA)(Extended Data Table 1).

Genomic Sequencing

High-molecular-weight genomic DNA was isolated from young leaf tissue, following a 72 hour dark treatment, using a modified nuclei preparation method [134,135]. DNA quality was verified by pulsed-field gel electrophoresis. DNA fragments longer than 50Kb were used to construct a 10X Gemcode library using the Chromium instrument (10X Genomics; Pleasanton, CA) and sequenced at HudsonAlpha Institute for Biotechnology (Huntsville, AL) on a HiSeqX system (lllumina; San Diego, CA) with paired-end 150bp reads. A total of ~95Gb (~58 fold coverage, based on an estimated genome size of 1.63Gb) of 10X Chromium library data was sequenced (Table S1). To increase sequence diversity and depth, three separate Mate-Pair (MP) libraries were constructed with 2-5Kb, 5-7Kb and 7-10Kb jumps using the Illumina Nextera Mate-Pair Sample Preparation Kit. In addition, two additional size-selected Illumina genomic libraries, ~470bp and ~800bp, were sequenced. The ~470bp and ~800bp libraries were made using the Illumina TruSeq DNA PCR-free Sample Preparation V2 kit. The ~470bp library was designed to produce ‘overlapping libraries’ after sequencing with paired-end 265bp reads on a lllumina Hiseq2500 system, producing ‘stitched’ reads of approximately 265bp to 520bp in length. The 800bp library was sequenced on an Illumina HiSeq2500 system with paired-end 160bp reads while the MP libraries were sequenced on an Illumina HiSeq4000 system with paired end 150bp reads. A total of ~433Gb (~266x fold coverage) of additional Illumina sequencing data was generated (Table S1). Illumina library construction and sequencing was conducted at Roy J. Carver Biotechnology Center, University of Illinois at Urbana-Champaign.

Genome Assembly

The genome of 'Draper' was assembled using the DeNovoMAGIC software platform (NRGene, Nes Ziona, Isreal); a De Bruijn graph-based assembler designed for higher polyploid, heterozygous and/or repetitive genomes [24,136]. The Chromium 10X data was utilized to phase, elongate and validate haplotype scaffolds. Four Dovetail Hi-C libraries were prepared as described previously [137] and sequenced on an Illumina HiSeq X system with paired-end 150bp reads to a total of 90.7X physical coverage of the genome (Figure S1). The *de novo* genome assembly, raw genomic reads, and Dovetail Hi-C library reads were used as input data for HiRise, a software pipeline designed specifically for using proximity ligation data to scaffold genome assemblies [138]. Illumina genomic and Dovetail Hi-C library sequences were aligned to the draft input assembly using a modified SNAP read mapper (http://snap.cs.berkeley.edu). The separations of Dovetail Hi-C read pairs mapped within draft scaffolds were analyzed by HiRise to produce a likelihood model for genomic distance between read pairs, and the model was used to identify and break putative misjoins, and to make joins to close gaps between contigs.

Collection of blueberry tissue samples, RNA library preparation and sequencing

Plant tissue samples (flower bud, flower at anthesis, flower post-anthesis, young shoot, leaves treated with methyl jasmonate, small green fruit, expanding green fruit, pink fruit, ripe fruit and salt-treated and untreated roots) were collected from blueberry cv. 'Draper' grown in the growth chamber (16/8 hours photoperiod; 408mE light intensity; 23/20C day/night temperature). For the fruit developmental series, three biological replicates each of berries at seven developmental stages (petal fall/cup, small green fruit, expanding green fruit, pink fruit, purple reddish fruit, purple unripe fruit and blue ripe fruit) were collected from cv. Draper in a field at the Horticulture Teaching and Research Center, MSU in July 2017. All plant tissues were immediately flash frozen in liquid nitrogen and total RNA isolation was performed using the KingFisher Pure RNA Plant kit (Thermo Fisher Scientific, MA). Isolated total RNA was quantified using a Qubit 3 Fluorometer (Thermo Fisher Scientific, MA). RNA libraries were prepared according to the KAPA mRNA HyperPrep kit protocol (KAPA Biosystems, Roche, USA). All samples were submitted to Michigan State University Research Technology Support Facility (RTSF) Genomics core and sequenced with paired-end 150bp reads on an Illumina HiSeq 4000 system (Illumina, San Diego, CA, USA).

Genome Annotation

The draft genome of *V. corymbosum* cv. 'Draper' was annotated using the MAKER annotation pipeline [25]. Transcript and protein evidence used in the annotation included protein sequences downloaded from *A. thaliana* (Araport11) and UniprotKB plant databases, *V. corymbosum* expressed sequence tags (EST) from NCBI, and transciptome data assembled with StringTie [139] from different blueberry tissues (Table S4). A custom repeat library and Repbase [140] were used to mask repetitive regions in the genome using Repeatmasker [141]. *Ab initio* gene prediction was performed using gene predictors SNAP [142] and Augustus [143]. The resulting MAKER Max gene set was filtered to select gene models with Pfam domain and annotation edit distance (AED) < 1.0. The filtered gene set (MAKER standard) was further scanned for transposase coding regions. The amino acid sequence of predicted genes was searched (BLASTP, 1e-10) against a transposase database [25]. The alignment between the genes and the transposases was further filtered for those caused by the presence of sequences with low complexity. The total length of genes matching transposases was calculated based on the output from the search. If more than 30% of gene

length aligned to the transposases, the gene is removed from the gene set. Furthermore, to assess the completeness of annotation, the *V. corymbosum* Maker standard gene set was searched against the Benchmarking Universal Single-Copy Orthologs (BUSCO v.2) [29] plant dataset (embryophyta_odb9). Genes were annotated with pfam domains using InterProScan v5.26-65.0 [144].

Annotation of repetitive elements

To identify and classify repetitive elements in the genome, long terminal repeat (LTR) retrotransposon candidates were searched using LTRharvest [145] and LTR_finder [146], and further identified and classified (e.g., Copia and Gypsy) using LTR_retriever [38]. A non-redundant LTR library was also produced by LTR_retriever. Miniature inverted transposable elements (MITEs) were identified using MITE-Hunter [147]. MITEs were manually checked for target site duplications and terminal inverted repeats and classified into superfamilies (e.g. *Mutator*, *hAT*, *Tc1Mariner/Stowaway* and *PIF/Harbinger*). Those with ambiguous TSD and TIR were classified as “unknowns.” Using the MITE and LTR libraries, the *V. corymbosum* genome was masked using Repeatmasker. The masked genome was further mined for repetitive elements using Repeatmodeler [148]. The repeats were then categorized into two groups: sequences with and without identities. Those without identities were searched against the transposase database and if they had a match, they were considered a transposon. The repeats were then filtered to exclude gene fragments using ProtExcluder [25] and summarized using the “fam_coverage.pl” script in the LTR_retriever package. The assembly continuity of repeat space was assessed using the LTR Assembly Index (LAI) [149] deployed in the LTR_retriever package [38]. LAI was calculated based on either 3 Mb sliding windows or the whole assembly using LAI = (Intact LTR-RT length * 100)/Total LTR-RT length. For the sliding window estimation, a step of 300 Kb was used (-step 300000 -window 3000000). To account for dynamics of LTR retrotransposons, LAI was adjusted by the mean identity of LTR sequences in the genome based on all-versus-all blastn search, which was also performed by the LAI program [149].

Transcriptome assembly and gene-expression analysis

Illumina adapters were removed from the raw reads using Trimmomatic/0.33 [150] and trimmed reads were filtered using FASTX Toolkit (http://hannonlab.cshl.edu/fastx_toolkit/index.html). After quality assessment using FastQC (http://www.bioinformatics.bbsrc.ac.uk/projects/fastqc), the filtered reads were then aligned to the *V. corymbosum* genome using STAR [151]. For the samples which were used for annotation, transcript assembly was performed *de novo* using StringTie. Counts of uniquely mapping reads were generated through HTSeq[152] for all 35 RNAseq datasets (plant tissue samples as well as fruit developmental series samples). Differential gene expression analysis was performed using the DESeq2 pipeline [153] across fruit developmental stages (e.g. stage 1 compared to stage 2)(Figure 3).

To construct the gene co-expression network, genes that were not expressed or very weakly expressed (count < 5) in 30 or more conditions were first excluded from the analysis. The count data was then transformed into variance stabilized values using the vst function in DEseq [152]. And pairwise correlations of gene expression was calculated using Pearson’s correlation coefficient (PCC) and mutual rank (MR)[154,155] using scripts available for download from the project’s data repository (https://purr.purdue.edu/projects/blueberrygenome). MR scores were transformed to network edge weights using geometric decay function $e^{-(MR-1/x)}$ [156]; five different co-expression networks were constructed with x set to 5, 10, 25, 50, and 100, respectively. Edges with PCC < 0.6 or edge weight <

0.01 was excluded. For each network, modules of co-expressed genes were detected using ClusterONE v1.0 using default parameters [157], and modules with P value > 0.1 or quality score < 0.2 were excluded. The results from all co-expression networks were then combined by collapsing modules into metamodules of nonoverlapping gene sets.

Oxygen Radical Absorbance Capacity (ORAC) Analysis

Total antioxidant capacity of tissues from the fruit developmental panel was analyzed using the oxygen radical absorbance capacity (ORAC) assay [59]. Briefly, ~20-30 mg of frozen ground fruit tissue was measured for tissue samples prior to extraction. Sample extractions were performed on ground tissue using 1.8 mL of ice cold 50% acetone. Samples were vortexed, and then put on a shaker for 5 minutes at room temperature. Samples were then centrifuged at 4 °C for 15 minutes (4,500 *g*). The ORAC assay was performed in a 96-well black microplate (Thermo Fisher Scientific, Waltham, MA) using the FLUOstar OPTIMA microplate reader (BMG LABTECH, Offenburg, Germany). Each reaction well contained 150 µl of 0.08 µM fluorescein and 25 µl of 75 mM phosphate buffer (blank), Trolox standards (6-Hydroxy-2,5,7,8-tetramethylchroman-2-carboxylic acid), or diluted sample extracts. For blueberry tissue samples 1:80-1:20 dilutions were used. Upon loading all appropriate wells, the 96-well microplate was put into the microplate reader and incubated for 10 minutes at 37 °C. Following incubation, 25 µl of 150 mM AAPH (2,2'-azobis-2-methyl-propanimidamide, dihydrochloride) was added to each well and fluorescence measurements began immediately. Fluorescence measurements (excitation: 485 nm, emission: 520 nm) were taken for 90 seconds per cycle for 70 cycles until the fluorescent probe signal was completely quenched. The area under the fluorescence decay curve (AUC) was calculated for each well. The total antioxidant capacity of a sample was calculated by subtracting the AUC from the blank curve from the AUC of the sample curve to obtain the net AUC. Using Trolox (water-soluble analog of Vitamin E) of a known concentration a standard curve was generated (12.5 µM – 100 µM) and the total antioxidant capacity of each sample was calculated as Trolox Equivalents (TE). Each sample was run twice for two technical replicates. The coefficient of variation between technical replicates was required to be less than 0.20. Biological replicates (n= 3) were run for all tissues in the fruit developmental series.

Assay of phenolics and anthocyanin content

Berries from 'Draper' were collected as described above. Approximately 100 mg (~10:1 solvent/tissue ratio) of each frozen ground sample was resuspended in extraction solvent in a 2 ml tube (80% Methanol/20% water + 0.1% formic acid, containing 0.5 M telmisartan (internal standard)). Ground tissue was immediately mixed thoroughly to prevent thawing during extraction and to prevent metabolism of analytes by enzymes in the samples. All tubes were spun down for 10 min at 13,000 x g to pellet protein and other insoluble material. Then, 1 ml of supernatant was transferred to an autosampler vial. Anthocyanin content was evaluated by liquid chromatography-mass spectrometry (LC-MS) as follows: 5 ml of sample extract were separated using a 10 min gradient on a Waters Acquity HSS-T3 UPLC column (2.1 x 100 mm) on a Waters Acquity UPLC system interfaced with a Waters Xevo G2-XS quadrupole time-of-flight mass spectrometer (Waters Corp, Milford, MA). Column temperature was maintained at 40°C and the flow rate was 0.3 ml/min with starting conditions of 100% solvent A (water + 0.1% formic acid) and 0% solvent B (acetonitrile). The gradient was as follows: hold at 100% A for 0.5 min, ramp to 50% B at 6 min, then ramp to 99% B at 6.5 min, hold at 99% B to 8.5 min, return to 100% A at 8.51 min and hold at 100% A until 10 min. Mass spectra were acquired in positive ion mode electrospray ionization over m/z 50-1500 in continuum mode using a data-

independent MS^E method which acquires data under both low and high collision energy conditions with the high collision energy setting using a ramp from 20-80 V. Capillary voltage was 3 kV, desolvation temperature was 350°C, source temperature was 100°C, cone gas flow was 25 L/hr and desolvation gas flow was 600 L/hr. Correction for mass drift was performed using continuous infusion of the lock mass compound leucine encephalin. Anthocyanins and other related flavonoids were identified based on accurate mass and fragmentation pattern. Peak areas were determined using Quanlynx within the Masslynx software package (Waters Corp). Relative anthocyanin content was calculated for each sample using the formula: reported peak area of the compound/peak area of internal standard/weight of extracted tissue (peak area/IS/gdw).

Genomic and Gene Family Analyses

The genome was aligned against itself in CoGe's SynMap program using LAST and default parameters [158]. Maximum distance between two matches was set to 20 genes, with minimum number of aligned pairs set to 10 genes. Tandemly duplicated genes were identified and filtered from CoGe outputs with a max distance of 10 genes. Fractionation bias was calculated, setting the max query and target chromosomes to 48. These analyses can be regenerated using the following link: https://genomevolution.org/r/12w9o. Protein sequences of blueberry was searched against previously characterized antioxidant related genes in Arabidopsis and other species in UniprotKB and NCBI databases using blastp in the BLAST+ package [159] with a cut-off e-value of 1E-10.

Data availability:

The genome assembly, annotations, and other supporting data are publicly available on PURR (https://purr.purdue.edu/projects/blueberrygenome). Genome assembly and annotation will also be made publicly available on Phytozome (https://phytozome.jgi.doe.gov/) and the CyVerse CoGe platform (https://genomevolution.org/coge/). The raw sequence data will be deposited in the Short Read Archive under NCBI BioProject ID PRJNAXXXXXX.

Competing interests:

The authors declare that they have no competing interests.

Acknowledgements:

This work was supported by Michigan State University AgBioResearch, USDA-NIFA HATCH 1009804 to P.P.E., USDA-NIFA AFRI 1015241 to P.P.E, and USDA-NIFA HATCH 1016057 to J.H.W.

References:

1. Coville, F. V. *Experiments in Blueberry Culture*. (U.S. Government Printing Office, 1910).

2. Ballington, J. R. Collection, utilization, and preservation of genetic resources in Vaccinium. *HortScience* **36,** 213–220 (2001).

3. Lewis, N. M. & Ruud, J. Blueberries in the American Diet. *Nutr. Today* **40,** 92 (2005).

4. Faostat, F. Statistical data. *Food and Agriculture Organization of the United Nations, Rome* (2017).

5. Kron, K. A. *et al.* Phylogenetic Classification of Ericaceae: Molecular and Morphological Evidence. *Bot. Rev.* **68,** 335–423 (2002).

6. Schwery, O. *et al.* As old as the mountains: the radiations of the Ericaceae. *New Phytol.* **207,** 355–367 (2015).

7. Michalska, A. & Lysiak, G. Bioactive Compounds of Blueberries: Post-Harvest Factors Influencing the Nutritional Value of Products. *Int. J. Mol. Sci.* **16,** 18642–18663 (2015).

8. Davidson, K. T. *et al.* Beyond Conventional Medicine - a Look at Blueberry, a Cancer-Fighting Superfruit. *Pathol. Oncol. Res.* (2017). doi:10.1007/s12253-017-0376-2

9. Faostat, F. FAOSTAT statistical database. (2016).

10. Vorsa, N. & Johnson-Cicalese, J. American Cranberry. in *Fruit Breeding* (eds. Badenes, M. L. & Byrne, D. H.) 191–223 (Springer US, 2012).

11. Darrow, G. M. & Others. The strawberry. History, breeding and physiology. *The strawberry. History, breeding and physiology.* (1966).

12. Prior, R. L. *et al.* Antioxidant Capacity As Influenced by Total Phenolic and Anthocyanin Content, Maturity, and Variety of Vaccinium Species. *J. Agric. Food Chem.* **46,** 2686–2693 (1998).

13. Kim, H., Bartley, G. E., Rimando, A. M. & Yokoyama, W. Hepatic gene expression related to lower plasma cholesterol in hamsters fed high-fat diets supplemented with blueberry peels and peel extract. *J. Agric. Food Chem.* **58,** 3984–3991 (2010).

14. Wang, S. Y., Camp, M. J. & Ehlenfeldt, M. K. Antioxidant capacity and α-glucosidase inhibitory activity in peel and flesh of blueberry (Vaccinium spp.) cultivars. *Food Chem.* **132,** 1759–1768 (2012).

15. Faria, A. *et al.* Blueberry anthocyanins and pyruvic acid adducts: anticancer properties in breast cancer cell lines. *Phytother. Res.* **24,** 1862–1869 (2010).

16. Hurst, R. D. *et al.* Blueberry fruit polyphenolics suppress oxidative stress-induced skeletal muscle cell damage in vitro. *Mol. Nutr. Food Res.* **54,** 353–363 (2010).

17. Krikorian, R. *et al.* Blueberry supplementation improves memory in older adults. *J. Agric. Food*

Chem. **58,** 3996–4000 (2010).

18. Norberto, S. *et al.* Blueberry anthocyanins in health promotion: A metabolic overview. *J. Funct. Foods* **5,** 1518–1528 (2013).
19. Wang, Y., Cheng, M., Zhang, B., Nie, F. & Jiang, H. Dietary supplementation of blueberry juice enhances hepatic expression of metallothionein and attenuates liver fibrosis in rats. *PLoS One* **8,** e58659 (2013).
20. Stull, A. *et al.* Blueberry Bioactives Improve Endothelial Function in Adults with Metabolic Syndrome. *The FASEB Journal* (2015).
21. Bell, L., Lamport, D. J., Butler, L. T. & Williams, C. M. A study of glycaemic effects following acute anthocyanin-rich blueberry supplementation in healthy young adults. *Food Funct.* **8,** 3104–3110 (2017).
22. Gallardo, R. K. *et al.* Blueberry Producers' Attitudes toward Harvest Mechanization for Fresh Market. *Horttechnology* **28,** 10–16 (2018).
23. Gupta, V. *et al.* RNA-Seq analysis and annotation of a draft blueberry genome assembly identifies candidate genes involved in fruit ripening, biosynthesis of bioactive compounds, and stage-specific alternative splicing. *Gigascience* **4,** 5 (2015).
24. Avni, R. *et al.* Wild emmer genome architecture and diversity elucidate wheat evolution and domestication. *Science* **357,** 93–97 (2017).
25. Campbell, M. S. *et al.* MAKER-P: a tool kit for the rapid creation, management, and quality control of plant genome annotations. *Plant Physiol.* **164,** 513–524 (2014).
26. Arabidopsis Genome Initiative. Analysis of the genome sequence of the flowering plant Arabidopsis thaliana. *Nature* **408,** 796–815 (2000).
27. Berardini, T. Z. *et al.* The Arabidopsis information resource: Making and mining the 'gold standard' annotated reference plant genome. *Genesis* **53,** 474–485 (2015).
28. Huang, S. *et al.* Draft genome of the kiwifruit Actinidia chinensis. *Nat. Commun.* **4,** 2640 (2013).
29. Simão, F. A., Waterhouse, R. M., Ioannidis, P., Kriventseva, E. V. & Zdobnov, E. M. BUSCO:

assessing genome assembly and annotation completeness with single-copy orthologs. *Bioinformatics* **31,** 3210–3212 (2015).

30. Götz, S. *et al.* High-throughput functional annotation and data mining with the Blast2GO suite. *Nucleic Acids Res.* **36,** 3420–3435 (2008).

31. Kanehisa, M. & Goto, S. KEGG: kyoto encyclopedia of genes and genomes. *Nucleic Acids Res.* **28,** 27–30 (2000).

32. Edger, P. P. *et al.* Single-molecule sequencing and optical mapping yields an improved genome of woodland strawberry (Fragaria vesca) with chromosome-scale contiguity. *Gigascience* **7,** 1–7 (2018).

33. VanBuren, R. *et al.* The genome of black raspberry (Rubus occidentalis). *Plant J.* **87,** 535–547 (2016).

34. Canaguier, A. *et al.* A new version of the grapevine reference genome assembly (12X.v2) and of its annotation (VCost.v3). *Genom Data* **14,** 56–62 (2017).

35. Law, M. *et al.* Automated update, revision, and quality control of the maize genome annotations using MAKER-P improves the B73 RefGen_v3 gene models and identifies new genes. *Plant Physiol.* **167,** 25–39 (2015).

36. Lee, S.-I. & Kim, N.-S. Transposable elements and genome size variations in plants. *Genomics Inform.* **12,** 87–97 (2014).

37. Vicient, C. M. & Casacuberta, J. M. Impact of transposable elements on polyploid plant genomes. *Ann. Bot.* **120,** 195–207 (2017).

38. Ou, S. & Jiang, N. LTR_retriever: A Highly Accurate and Sensitive Program for Identification of Long Terminal Repeat Retrotransposons. *Plant Physiol.* **176,** 1410–1422 (2018).

39. Coville, F. V. BLUEBERRY CHROMOSOMES. *Science* **66,** 565–566 (1927).

40. Draper, A. D. & Scott, D. H. Inheritance of albino seedling in tetraploid highbush blueberry. *J. Am. Soc. Hortic. Sci.* (1971).

41. Jelenkovic, G. & Hough, L. F. CHROMOSOME ASSOCIATIONS IN THE FIRST MEIOTIC

DIVISION IN THREE TETRAPLOID CLONES OF VACCINIUM CORYMBOSUM L. *Can. J. Genet. Cytol.* **12,** 316–324 (1970).

42. Xiong, Z., Gaeta, R. T. & Pires, J. C. Homoeologous shuffling and chromosome compensation maintain genome balance in resynthesized allopolyploid Brassica napus. *Proc. Natl. Acad. Sci. U. S. A.* **108,** 7908–7913 (2011).
43. Chester, M. *et al.* Extensive chromosomal variation in a recently formed natural allopolyploid species, Tragopogon miscellus (Asteraceae). *Proc. Natl. Acad. Sci. U. S. A.* **109,** 1176–1181 (2012).
44. Ozkan, H., Levy, A. A. & Feldman, M. Rapid differentiation of homeologous chromosomes in newly-formed allopolyploid wheat. *Isr. J. Plant Sci.* **50,** 65–76 (2002).
45. VanBuren, R. *et al.* Extreme haplotype variation in the desiccation-tolerant clubmoss Selaginella lepidophylla. *Nat. Commun.* **9,** 13 (2018).
46. Thomas, B. Light signals and flowering. *J. Exp. Bot.* **57,** 3387–3393 (2006).
47. Grover, C. E. *et al.* Homoeolog expression bias and expression level dominance in allopolyploids. *New Phytol.* **196,** 966–971 (2012).
48. Woodhouse, M. R. *et al.* Origin, inheritance, and gene regulatory consequences of genome dominance in polyploids. *Proceedings of the National Academy of Sciences* **111,** 5283–5288 (2014).
49. Edger, P. P., McKain, M. R., Bird, K. A. & VanBuren, R. Subgenome assignment in allopolyploids: challenges and future directions. *Curr. Opin. Plant Biol.* **42,** 76–80 (2018).
50. Freeling, M. *et al.* Fractionation mutagenesis and similar consequences of mechanisms removing dispensable or less-expressed DNA in plants. *Curr. Opin. Plant Biol.* **15,** 131–139 (2012).
51. Bottani, S., Zabet, N. R., Wendel, J. F. & Veitia, R. A. Gene Expression Dominance in Allopolyploids: Hypotheses and Models. *Trends Plant Sci.* (2018). doi:10.1016/j.tplants.2018.01.002
52. Cheng, F. *et al.* Gene retention, fractionation and subgenome differences in polyploid plants. *Nature Plants* **4,** 258–268 (2018).

53. Garsmeur, O. *et al.* Two evolutionarily distinct classes of paleopolyploidy. *Mol. Biol. Evol.* **31,** 448–454 (2013).

54. Zhao, M., Zhang, B., Lisch, D. & Ma, J. Patterns and Consequences of Subgenome Differentiation Provide Insights into the Nature of Paleopolyploidy in Plants. *Plant Cell* **29,** 2974–2994 (2017).

55. Bohner, J. & Bangerth, F. Cell number, cell size and hormone levels in semi-isogenic mutants of Lycopersicon pimpinellifolium differing in fruit size. *Physiol. Plant.* **72,** 316–320 (1988).

56. Gillaspy, G., Ben-David, H. & Gruissem, W. Fruits: A Developmental Perspective. *Plant Cell* **5,** 1439–1451 (1993).

57. Zifkin, M. *et al.* Gene expression and metabolite profiling of developing highbush blueberry fruit indicates transcriptional regulation of flavonoid metabolism and activation of abscisic acid metabolism. *Plant Physiol.* **158,** 200–224 (2012).

58. Mainland, C. M. & Tucker, J. W. BLUEBERRY HEALTH INFORMATION - SOME NEW MOSTLY REVIEW. *Acta Hortic.* 39–43 (2002).

59. Gillespie, K. M., Chae, J. M. & Ainsworth, E. A. Rapid measurement of total antioxidant capacity in plants. *Nat. Protoc.* **2,** 867–870 (2007).

60. Connor, A. M., Luby, J. J., Tong, C. B. S., Finn, C. E. & Hancock, J. F. Genotypic and Environmental Variation in Antioxidant Activity, Total Phenolic Content, and Anthocyanin Content among Blueberry Cultivars. *J. Am. Soc. Hortic. Sci.* **127,** 89–97 (2002).

61. Wang, H. *et al.* Comparison of phytochemical profiles, antioxidant and cellular antioxidant activities of different varieties of blueberry (Vaccinium spp.). *Food Chem.* **217,** 773–781 (2017).

62. Wu, Y. *et al.* Comparison and screening of bioactive phenolic compounds in different blueberry cultivars: Evaluation of anti-oxidation and α-glucosidase inhibition effect. *Food Res. Int.* **100,** 312–324 (2017).

63. Kalt, W. *et al.* Interspecific variation in anthocyanins, phenolics, and antioxidant capacity among genotypes of highbush and lowbush blueberries (Vaccinium section cyanococcus spp.). *J. Agric. Food Chem.* **49,** 4761–4767 (2001).

64. Moyer, R. A., Hummer, K. E., Finn, C. E., Frei, B. & Wrolstad, R. E. Anthocyanins, phenolics, and antioxidant capacity in diverse small fruits: vaccinium, rubus, and ribes. *J. Agric. Food Chem.* **50,** 519–525 (2002).

65. Castrejón, A. D. R., Eichholz, I., Rohn, S., Kroh, L. W. & Huyskens-Keil, S. Phenolic profile and antioxidant activity of highbush blueberry (Vaccinium corymbosum L.) during fruit maturation and ripening. *Food Chem.* **109,** 564–572 (2008).

66. Wang, S. Y. & Lin, H. S. Antioxidant activity in fruits and leaves of blackberry, raspberry, and strawberry varies with cultivar and developmental stage. *J. Agric. Food Chem.* **48,** 140–146 (2000).

67. Zheng, W. & Wang, S. Y. Oxygen radical absorbing capacity of phenolics in blueberries, cranberries, chokeberries, and lingonberries. *J. Agric. Food Chem.* **51,** 502–509 (2003).

68. Clifford, M. N. Chlorogenic Acids. in *Coffee: Volume 1: Chemistry* (eds. Clarke, R. J. & Macrae, R.) 153–202 (Springer Netherlands, 1985).

69. Rice-Evans, C. A., Miller, N. J. & Paganga, G. Structure-antioxidant activity relationships of flavonoids and phenolic acids. *Free Radic. Biol. Med.* **20,** 933–956 (1996).

70. Shi, H. *et al.* Chlorogenic acid protects against liver fibrosis in vivo and in vitro through inhibition of oxidative stress. *Clin. Nutr.* **35,** 1366–1373 (2016).

71. Hollman, P. C. Evidence for health benefits of plant phenols: local or systemic effects? *J. Sci. Food Agric.* **81,** 842–852 (2001).

72. Olthof, M. R., Hollman, P. C., Zock, P. L. & Katan, M. B. Consumption of high doses of chlorogenic acid, present in coffee, or of black tea increases plasma total homocysteine concentrations in humans--. *Am. J. Clin. Nutr.* **73,** 532–538 (2001).

73. Charurin, P., Ames, J. M. & del Castillo, M. D. Antioxidant activity of coffee model systems. *J. Agric. Food Chem.* **50,** 3751–3756 (2002).

74. Yen, W.-J., Wang, B.-S., Chang, L.-W. & Duh, P.-D. Antioxidant properties of roasted coffee residues. *J. Agric. Food Chem.* **53,** 2658–2663 (2005).

75. Watanabe, T. *et al.* The blood pressure-lowering effect and safety of chlorogenic acid from green

coffee bean extract in essential hypertension. *Clin. Exp. Hypertens.* **28,** 439–449 (2006).

76. Falcone Ferreyra, M. L., Rius, S. P. & Casati, P. Flavonoids: biosynthesis, biological functions, and biotechnological applications. *Front. Plant Sci.* **3,** 222 (2012).

77. Zhang, Y. Regulation of Ascorbate Synthesis in Plants. in *Ascorbic Acid in Plants: Biosynthesis, Regulation and Enhancement* (ed. Zhang, Y.) 87–99 (Springer New York, 2013).

78. Laing, W., Norling, C., Brewster, D., Wright, M. & Bulley, S. Ascorbate Concentration In Arabidopsis thaliana And Expression Of Ascorbate Related Genes Using RNAseq In Response To Light And The Diurnal Cycle. *bioRxiv* 138008 (2017). doi:10.1101/138008

79. Liu, J., Osbourn, A. & Ma, P. MYB Transcription Factors as Regulators of Phenylpropanoid Metabolism in Plants. *Mol. Plant* **8,** 689–708 (2015).

80. Petroni, K. & Tonelli, C. Recent advances on the regulation of anthocyanin synthesis in reproductive organs. *Plant Sci.* **181,** 219–229 (2011).

81. Albert, N. W. *et al.* A conserved network of transcriptional activators and repressors regulates anthocyanin pigmentation in eudicots. *Plant Cell* **26,** 962–980 (2014).

82. Huang, W. *et al.* A R2R3-MYB Transcription Factor Regulates the Flavonol Biosynthetic Pathway in a Traditional Chinese Medicinal Plant, Epimedium sagittatum. *Front. Plant Sci.* **7,** 1089 (2016).

83. Nguyen, N. H. & Lee, H. MYB-related transcription factors function as regulators of the circadian clock and anthocyanin biosynthesis in Arabidopsis. *Plant Signal. Behav.* **11,** e1139278 (2016).

84. Jin, J. *et al.* PlantTFDB 4.0: toward a central hub for transcription factors and regulatory interactions in plants. *Nucleic Acids Res.* **45,** D1040–D1045 (2017).

85. Kautsar, S. A., Suarez Duran, H. G., Blin, K., Osbourn, A. & Medema, M. H. plantiSMASH: automated identification, annotation and expression analysis of plant biosynthetic gene clusters. *Nucleic Acids Res.* **45,** W55–W63 (2017).

86. Xi, W., Zheng, H., Zhang, Q. & Li, W. Profiling Taste and Aroma Compound Metabolism during Apricot Fruit Development and Ripening. *Int. J. Mol. Sci.* **17,** (2016).

87. Du, X. & Rouseff, R. Aroma Active Volatiles in Four Southern Highbush Blueberry Cultivars

Determined by Gas Chromatography–Olfactometry (GC-O) and Gas Chromatography–Mass Spectrometry (GC-MS). *J. Agric. Food Chem.* **62,** 4537–4543 (2014).

88. Farneti, B. *et al.* Exploring Blueberry Aroma Complexity by Chromatographic and Direct-Injection Spectrometric Techniques. *Front. Plant Sci.* **8,** 617 (2017).

89. Beaulieu, J. C., Stein-Chisholm, R. E. & Boykin, D. L. Qualitative Analysis of Volatiles in Rabbiteye Blueberry Cultivars at Various Maturities Using Rapid Solid-phase Microextraction. *J. Am. Soc. Hortic. Sci.* **139,** 167–177 (2014).

90. Du, X., Whitaker, V. & Rouseff, R. Changes in strawberry volatile sulfur compounds due to genotype, fruit maturity and sample preparation. *Flavour Fragr. J.* **27,** 398–404 (2012).

91. Du, X., Plotto, A., Song, M., Olmstead, J. & Rouseff, R. Volatile composition of four southern highbush blueberry cultivars and effect of growing location and harvest date. *J. Agric. Food Chem.* **59,** 8347–8357 (2011).

92. Hirvi, T. & Honkanen, E. The aroma of blueberries. *J. Sci. Food Agric.* **34,** 992–996 (1983).

93. Horvat, R. J. & Senter, S. D. Comparison of the volatile constituents from rabbiteye blueberries (Vaccinium ashei) during ripening. *J. Food Sci.* **50,** 429–431 (1985).

94. Gilbert, J. L. *et al.* Consumer-assisted Selection of Blueberry Fruit Quality Traits. *HortScience* **49,** 864–873 (2014).

95. Eom, J.-S. *et al.* SWEETs, transporters for intracellular and intercellular sugar translocation. *Curr. Opin. Plant Biol.* **25,** 53–62 (2015).

96. Ren, Y. *et al.* A Tonoplast Sugar Transporter Underlies a Sugar Accumulation QTL in Watermelon. *Plant Physiol.* **176,** 836–850 (2018).

97. Lin, I. W. *et al.* Nectar secretion requires sucrose phosphate synthases and the sugar transporter SWEET9. *Nature* **508,** 546–549 (2014).

98. Chen, H.-Y. *et al.* The Arabidopsis vacuolar sugar transporter SWEET2 limits carbon sequestration from roots and restricts Pythium infection. *Plant J.* **83,** 1046–1058 (2015).

99. Wormit, A. *et al.* Molecular identification and physiological characterization of a novel

monosaccharide transporter from Arabidopsis involved in vacuolar sugar transport. *Plant Cell* **18,** 3476–3490 (2006).

100. Sturm, A. & Tang, G. Q. The sucrose-cleaving enzymes of plants are crucial for development, growth and carbon partitioning. *Trends Plant Sci.* **4,** 401–407 (1999).

101. Qin, G. *et al.* A Tomato Vacuolar Invertase Inhibitor Mediates Sucrose Metabolism and Influences Fruit Ripening. *Plant Physiol.* **172,** 1596–1611 (2016).

102. Achaz, G., Coissac, E., Viari, A. & Netter, P. Analysis of intrachromosomal duplications in yeast Saccharomyces cerevisiae: a possible model for their origin. *Mol. Biol. Evol.* **17,** 1268–1275 (2000).

103. Leister, D. Tandem and segmental gene duplication and recombination in the evolution of plant disease resistance genes. *Trends Genet.* **20,** 116–122 (2004).

104. Maere, S. *et al.* Modeling gene and genome duplications in eukaryotes. *Proc. Natl. Acad. Sci. U. S. A.* **102,** 5454–5459 (2005).

105. Chae, L., Kim, T., Nilo-Poyanco, R. & Rhee, S. Y. Genomic signatures of specialized metabolism in plants. *Science* **344,** 510–513 (2014).

106. Edger, P. P. & Pires, J. C. Gene and genome duplications: the impact of dosage-sensitivity on the fate of nuclear genes. *Chromosome Res.* **17,** 699–717 (2009).

107. Freeling, M. Bias in plant gene content following different sorts of duplication: tandem, whole-genome, segmental, or by transposition. *Annu. Rev. Plant Biol.* **60,** 433–453 (2009).

108. Kliebenstein, D. J., Lambrix, V. M., Reichelt, M., Gershenzon, J. & Mitchell-Olds, T. Gene duplication in the diversification of secondary metabolism: tandem 2-oxoglutarate-dependent dioxygenases control glucosinolate biosynthesis in Arabidopsis. *Plant Cell* **13,** 681–693 (2001).

109. Ober, D. Seeing double: gene duplication and diversification in plant secondary metabolism. *Trends Plant Sci.* **10,** 444–449 (2005).

110. Hofberger, J. A., Lyons, E., Edger, P. P., Pires, J. C. & Schranz, M. E. Whole Genome and Tandem Duplicate Retention Facilitated Glucosinolate Pathway Diversification in the Mustard Family. *Genome Biol. Evol.* **5,** 2155–2173 (2013).

111. Conant, G. C. & Wolfe, K. H. Turning a hobby into a job: how duplicated genes find new functions. *Nat. Rev. Genet.* **9,** 938–950 (2008).

112. Bekaert, M., Edger, P. P., Pires, J. C. & Conant, G. C. Two-phase resolution of polyploidy in the Arabidopsis metabolic network gives rise to relative and absolute dosage constraints. *Plant Cell* **23,** 1719–1728 (2011).

113. Nützmann, H.-W. & Osbourn, A. Regulation of metabolic gene clusters in Arabidopsis thaliana. *New Phytol.* **205,** 503–510 (2015).

114. Schläpfer, P. *et al.* Genome-Wide Prediction of Metabolic Enzymes, Pathways, and Gene Clusters in Plants. *Plant Physiol.* **173,** 2041–2059 (2017).

115. Nützmann, H.-W., Huang, A. & Osbourn, A. Plant metabolic clusters--from genetics to genomics. *New Phytol.* **211,** 771–789 (2016).

116. Kliebenstein, D. J. A role for gene duplication and natural variation of gene expression in the evolution of metabolism. *PLoS One* **3,** e1838 (2008).

117. Bird, K. A., VanBuren, R., Puzey, J. R. & Edger, P. P. The causes and consequences of subgenome dominance in hybrids and recent polyploids. *New Phytol.* (2018). doi:10.1111/nph.15256

118. Schnable, J. C., Springer, N. M. & Freeling, M. Differentiation of the maize subgenomes by genome dominance and both ancient and ongoing gene loss. *Proc. Natl. Acad. Sci. U. S. A.* **108,** 4069–4074 (2011).

119. McClintock, B. The significance of responses of the genome to challenge. *Science* **226,** 792–801 (1984).

120. Hollister, J. D. & Gaut, B. S. Epigenetic silencing of transposable elements: a trade-off between reduced transposition and deleterious effects on neighboring gene expression. *Genome Res.* **19,** 1419–1428 (2009).

121. Wendel, J. F., Lisch, D., Hu, G. & Mason, A. S. The long and short of doubling down: polyploidy, epigenetics, and the temporal dynamics of genome fractionation. *Curr. Opin. Genet. Dev.* **49,** 1–7 (2018).

122. Cheng, F. *et al.* Biased gene fractionation and dominant gene expression among the subgenomes of Brassica rapa. *PLoS One* **7,** e36442 (2012).

123. Edger, P. P. *et al.* Subgenome Dominance in an Interspecific Hybrid, Synthetic Allopolyploid, and a 140-Year-Old Naturally Established Neo-Allopolyploid Monkeyflower. *Plant Cell* **29,** 2150–2167 (2017).

124. Wagner, G. P. & Lynch, V. J. The gene regulatory logic of transcription factor evolution. *Trends Ecol. Evol.* **23,** 377–385 (2008).

125. Kuo, D. *et al.* Coevolution within a transcriptional network by compensatory trans and cis mutations. *Genome Res.* **20,** 1672–1678 (2010).

126. Lavoie, H. *et al.* Evolutionary tinkering with conserved components of a transcriptional regulatory network. *PLoS Biol.* **8,** e1000329 (2010).

127. Liang, Z. & Schnable, J. C. Functional Divergence between Subgenomes and Gene Pairs after Whole Genome Duplications. *Mol. Plant* **11,** 388–397 (2018).

128. Osborn, T. C. *et al.* Understanding mechanisms of novel gene expression in polyploids. *Trends Genet.* **19,** 141–147 (2003).

129. Birchler, J. A. & Veitia, R. A. Gene balance hypothesis: connecting issues of dosage sensitivity across biological disciplines. *Proc. Natl. Acad. Sci. U. S. A.* **109,** 14746–14753 (2012).

130. Eckardt, N. A. Genome Dominance and Interaction at the Gene Expression Level in Allohexaploid Wheat. *Plant Cell* **26,** 1834 (2014).

131. Li, A. *et al.* mRNA and Small RNA Transcriptomes Reveal Insights into Dynamic Homoeolog Regulation of Allopolyploid Heterosis in Nascent Hexaploid Wheat. *Plant Cell* **26,** 1878–1900 (2014).

132. Pfeifer, M. *et al.* Genome interplay in the grain transcriptome of hexaploid bread wheat. *Science* **345,** 1250091 (2014).

133. International Wheat Genome Sequencing Consortium (IWGSC) *et al.* Shifting the limits in wheat research and breeding using a fully annotated reference genome. *Science* **361,** (2018).

134. Zhang, H.-B., Zhao, X., Ding, X., Paterson, A. H. & Wing, R. A. Preparation of megabase-size DNA from plant nuclei. *Plant J.* **7,** 175–184 (1995).

135. VanBuren, R. *et al.* Single-molecule sequencing of the desiccation-tolerant grass Oropetium thomaeum. *Nature* **527,** 508–U209 (2015).

136. Luo, M.-C. *et al.* Genome sequence of the progenitor of the wheat D genome Aegilops tauschii. *Nature* **551,** 498–502 (2017).

137. Lieberman-Aiden, E. *et al.* Comprehensive mapping of long-range interactions reveals folding principles of the human genome. *Science* **326,** 289–293 (2009).

138. Putnam, N. H. *et al.* Chromosome-scale shotgun assembly using an in vitro method for long-range linkage. *Genome Res.* (2016). doi:10.1101/gr.193474.115

139. Pertea, M. *et al.* StringTie enables improved reconstruction of a transcriptome from RNA-seq reads. *Nat. Biotechnol.* **33,** 290–295 (2015).

140. Jurka, J. *et al.* Repbase Update, a database of eukaryotic repetitive elements. *Cytogenet. Genome Res.* **110,** 462–467 (2005).

141. Smit, A. F. A., Hubley, R. & Green, P. RepeatMasker. (1996).

142. Korf, I. Gene finding in novel genomes. *BMC Bioinformatics* **5,** 59 (2004).

143. Stanke, M. & Waack, S. Gene prediction with a hidden Markov model and a new intron submodel. *Bioinformatics* **19 Suppl 2,** ii215–25 (2003).

144. Jones, P. *et al.* InterProScan 5: genome-scale protein function classification. *Bioinformatics* **30,** 1236–1240 (2014).

145. Ellinghaus, D., Kurtz, S. & Willhoeft, U. LTRharvest, an efficient and flexible software for de novo detection of LTR retrotransposons. *BMC Bioinformatics* **9,** 18 (2008).

146. Xu, Z. & Wang, H. LTR_FINDER: an efficient tool for the prediction of full-length LTR retrotransposons. *Nucleic Acids Res.* **35,** W265–8 (2007).

147. Han, Y. & Wessler, S. R. MITE-Hunter: a program for discovering miniature inverted-repeat transposable elements from genomic sequences. *Nucleic Acids Res.* **38,** e199 (2010).

148. Smit, A. & Hubley, R. RepeatModeler Open-1.0. *Available fom http://www. repeatmasker. org* (2008).

149. Ou, S. *LTR_retriever*. (Github).

150. Bolger, A. M., Lohse, M. & Usadel, B. Trimmomatic: a flexible trimmer for Illumina sequence data. *Bioinformatics* **30,** 2114–2120 (2014).

151. Dobin, A. & Gingeras, T. R. Mapping RNA-seq Reads with STAR. *Curr. Protoc. Bioinformatics* **51,** 11.14.1–19 (2015).

152. Anders, S., Pyl, P. T. & Huber, W. HTSeq--a Python framework to work with high-throughput sequencing data. *Bioinformatics* **31,** 166–169 (2015).

153. Love, M. I., Huber, W. & Anders, S. Moderated estimation of fold change and dispersion for RNA-seq data with DESeq2. *Genome Biol.* **15,** 550 (2014).

154. Obayashi, T. & Kinoshita, K. Rank of correlation coefficient as a comparable measure for biological significance of gene coexpression. *DNA Res.* **16,** 249–260 (2009).

155. Obayashi, T., Aoki, Y., Tadaka, S., Kagaya, Y. & Kinoshita, K. ATTED-II in 2018: A Plant Coexpression Database Based on Investigation of the Statistical Property of the Mutual Rank Index. *Plant Cell Physiol.* **59,** 440 (2018).

156. Wisecaver, J. H. *et al.* A Global Co-expression Network Approach for Connecting Genes to Specialized Metabolic Pathways in Plants. *Plant Cell* (2017). doi:10.1105/tpc.17.00009

157. Nepusz, T., Yu, H. & Paccanaro, A. Detecting overlapping protein complexes in protein-protein interaction networks. *Nat. Methods* **9,** 471–472 (2012).

158. Lyons, E., Pedersen, B., Kane, J. & Freeling, M. The Value of Nonmodel Genomes and an Example Using SynMap Within CoGe to Dissect the Hexaploidy that Predates the Rosids. *Trop. Plant Biol.* **1,** 181–190 (2008).

159. Camacho, C. *et al.* BLAST+: architecture and applications. *BMC Bioinformatics* **10,** 421 (2009).

Click here to download Table Extended_Data_Table1.xlsx

FLOW CYTOMETRIC ESTIMATION OF NUCLEAR DNA CONTENT OF PLANT SAMPLES
Date of analysis: 03/24/16

Sample name	Sample ID	Sample G0+G1 mean	Standard(int G0+G1 mean	DNA content (pg/2C)	St. Dev ±
Draper	***10***	178.66	272.47	1.64	
V. corymbosum		189.51	290.77	1.63	
		210.66	315.85	1.67	
		230.37	340.20	1.69	
				**1.66**	**0.029**

Caluclation for Genome Size
DNA content to Mb conversion (1C = 980M

Average Estimate
1626.8

Estimate (- 1 St. Dev.)
1595.9

* Grover et al., 2004 Genome Research.
https://genome.cshlp.org/content/14/8/1474.full

Estimate (+ 1 St. Dev.)
1652.2

1.66 pg

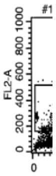

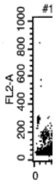

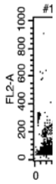

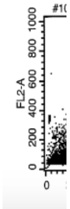

Click here to download Table Extended_Data_Table2.xlsx

Your MS Excel document "Extended_Data_Table2.xlsx" cannot be opened and processed. Please see the common list of problems, and suggested resolutions below.

External Data

If you are submitting an Excel file, please make sure that your document does not have links to external data. If it does, break the links, save the document and resend. To break the links please do the following.
-On the Edit menu, click Links.
-In the Source list, click the link you want to break.
-To select multiple linked objects, hold down CTRL and click each linked object. To select all links, press CTRL+A.
-Click Break Link.

Embedded Macros

Your submission should not contain macros. If they do, an alert box may appear when you open your document (this alert box prevents EM from automatically converting your Excel document into the PDF that Editors and Reviewers will use). You must adjust your Excel document to remove these macros.

Excel 2002/Excel XP files

At the present time, EM supports Excel files in Excel 2000 and earlier formats. If you are using a more recent version of MS Excel, try saving your Excel document in a format compatible with Excel 2000, and resubmit to EM.

Other Problems

If you are able to get your Excel document to open with no alert boxes appearing, and you have submitted it in Excel 2000 (or earlier) format, and you still see an error indication in your PDF file (where your Excel document should be appearing). please contact the journal via the 'Contact Us' button on the Navigation Bar.'

You will need to reformat your Excel document, and then re-submit it.

Click here to download Table Extended_Data_Table3.xlsx

Extended Data Table 3. Anthocyanin, chlorogenic acid and ascorbic acid biosynth

qseqid	sseqid	pident
**a. Anthocyanin and chlorogenic biosynthesis related genes**		
Phenylalanine ammonia-lyase (PAL)		
augustus_masked-VaccDscaff10-processed-gene-225.6-mRN/	tr|G3GAT3|(	86.1
maker-VaccDscaff1-augustus-gene-185.22-mRNA-1	tr|G3GAT3|(	86.1
maker-VaccDscaff8-augustus-gene-236.15-mRNA-1	tr|G3GAT3|(	85.96
maker-VaccDscaff5-snap-gene-236.31-mRNA-1	tr|G3GAT3|(	85.55
maker-VaccDscaff1-augustus-gene-187.25-mRNA-1	tr|G3GAT3|(	85.39
maker-VaccDscaff10-augustus-gene-222.29-mRNA-1	tr|G3GAT3|(	85.24
maker-VaccDscaff8-augustus-gene-235.20-mRNA-1	tr|G3GAT3|(	85.24
maker-VaccDscaff5-augustus-gene-235.33-mRNA-1	tr|G3GAT3|(	85.24
maker-VaccDscaff15-augustus-gene-283.32-mRNA-1	tr|G3GAT3|(	84.79
maker-VaccDscaff10-augustus-gene-222.30-mRNA-1	tr|G3GAT3|(	84.67
maker-VaccDscaff8-augustus-gene-235.22-mRNA-1	tr|G3GAT3|(	84.67
maker-VaccDscaff19-augustus-gene-331.32-mRNA-1	tr|G3GAT3|(	84.65
maker-VaccDscaff24-augustus-gene-101.15-mRNA-1	tr|G3GAT3|(	84.37
maker-VaccDscaff1-augustus-gene-186.19-mRNA-1	tr|G3GAT3|(	84.24
maker-VaccDscaff5-snap-gene-236.34-mRNA-1	tr|G3GAT3|(	83.38
snap_masked-VaccDscaff11-processed-gene-299.22-mRNA-1	tr|G3GAT3|(	83.26
maker-VaccDscaff21-augustus-gene-179.27-mRNA-1	tr|J7FRN4|J	85.09
maker-VaccDscaff33-augustus-gene-169.12-mRNA-1	tr|J7FRN4|J	84.8
maker-VaccDscaff26-augustus-gene-180.26-mRNA-1	tr|J7FRN4|J	84.8
maker-VaccDscaff29-augustus-gene-173.18-mRNA-1	tr|J7FRN4|J	84.8
4-hydroxycinnamoyl-CoA ligase (CoAl_4CL)		
maker-VaccDscaff6-augustus-gene-422.25-mRNA-1	tr|J7ETF7|J	82.4
maker-VaccDscaff37-augustus-gene-303.53-mRNA-1	tr|J7ETF7|J	82.21
maker-VaccDscaff38-snap-gene-101.15-mRNA-1	tr|J7ETF7|J	82.08
maker-VaccDscaff160-augustus-gene-2.34-mRNA-1	tr|J7ETF7|J	82.02
augustus_masked-VaccDscaff34-processed-gene-57.9-mRNA	tr|J7ETF7|J	76.85
augustus_masked-VaccDscaff27-processed-gene-288.0-mRN/	tr|J7ETF7|J	76.3
augustus_masked-VaccDscaff17-processed-gene-323.0-mRN/	tr|J7ETF7|J	76.3
augustus_masked-VaccDscaff50-processed-gene-21.6-mRNA	tr|J7ETF7|J	74.55
maker-VaccDscaff20-snap-gene-2.38-mRNA-1	tr|J7ETF7|J	60.96
maker-VaccDscaff28-augustus-gene-349.38-mRNA-1	tr|J7ETF7|J	58.61
maker-VaccDscaff21-augustus-gene-58.51-mRNA-1	tr|J7FPQ8|J	78.11
maker-VaccDscaff26-augustus-gene-61.51-mRNA-1	tr|J7FPQ8|J	78.11
maker-VaccDscaff29-augustus-gene-61.39-mRNA-1	tr|J7FPQ8|J	77.92
snap_masked-VaccDscaff33-processed-gene-276.28-mRNA-1	tr|J7FPQ8|J	75.65
Cinnamate 4-hydroxylase (C4H)		
maker-VaccDscaff22-augustus-gene-301.32-mRNA-1	sp|P48522|T	91.49
maker-VaccDscaff302-augustus-gene-0.32-mRNA-1	sp|P48522|T	91.29

maker-VaccDscaff47-augustus-gene-123.34-mRNA-1	sp|P48522|T	91.29
maker-VaccDscaff46-augustus-gene-145.24-mRNA-1	sp|P48522|T	91.27
maker-VaccDscaff43-augustus-gene-75.21-mRNA-1	sp|P48522|T	91.09
augustus_masked-VaccDscaff33-processed-gene-307.8-mRN	sp|P48522|T	91.09
maker-VaccDscaff21-augustus-gene-13.24-mRNA-1	sp|P48522|T	90.69
maker-VaccDscaff29-augustus-gene-7.44-mRNA-1	sp|P48522|T	90.69
maker-VaccDscaff11-augustus-gene-343.38-mRNA-1	sp|P48522|T	89.26
maker-VaccDscaff15-augustus-gene-332.34-mRNA-1	sp|P48522|T	89.13
maker-VaccDscaff190-augustus-gene-0.32-mRNA-1	sp|P48522|T	88.93
maker-VaccDscaff24-augustus-gene-58.38-mRNA-1	tr|Q9FQY6|(	86.14

Hydroxycinnamoyl-CoA shikimate/quinate hydroxycinnamoyltransferase (HCT)

snap_masked-VaccDscaff17-processed-gene-332.40-mRNA-1	sp|Q8GSM7	83
maker-VaccDscaff27-snap-gene-291.30-mRNA-1	tr|A4ZKE4|A	81.8
augustus_masked-VaccDscaff34-processed-gene-53.2-mRNA	tr|A4ZKE4|A	81.8
augustus_masked-VaccDscaff17-processed-gene-329.4-mRN	tr|A4ZKE4|A	81.34
augustus_masked-VaccDscaff15-processed-gene-101.5-mRN	tr|A4ZKI0|A	61.61
augustus_masked-VaccDscaff38-processed-gene-2.16-mRNA	tr|A4ZKI0|A	61.5
maker-VaccDscaff413-augustus-gene-0.24-mRNA-1	tr|A4ZKI0|A	61.5
maker-VaccDscaff11-augustus-gene-106.20-mRNA-1	tr|A4ZKI0|A	61.38
augustus_masked-VaccDscaff24-processed-gene-278.6-mRN	tr|A4ZKI0|A	61.38
maker-VaccDscaff24-augustus-gene-299.21-mRNA-1	tr|A4ZKI0|A	61.38
maker-VaccDscaff6-augustus-gene-402.29-mRNA-1	tr|A4ZKI0|A	61.05
augustus_masked-VaccDscaff39-processed-gene-44.7-mRNA	tr|A4ZKI0|A	60.82
maker-VaccDscaff6-augustus-gene-399.26-mRNA-1	tr|A4ZKI0|A	59.04
maker-VaccDscaff37-snap-gene-282.24-mRNA-1	tr|A4ZKI0|A	59.04
augustus_masked-VaccDscaff38-processed-gene-5.8-mRNA-1	tr|A4ZKI0|A	58.94
maker-VaccDscaff39-augustus-gene-47.29-mRNA-1	tr|A4ZKI0|A	58.58
maker-VaccDscaff38-augustus-gene-2.42-mRNA-1	tr|A4ZKI0|A	56.85
snap_masked-VaccDscaff39-processed-gene-47.15-mRNA-1	tr|A4ZKI0|A	56.79
augustus_masked-VaccDscaff6-processed-gene-403.6-mRNA	tr|A4ZKI0|A	56.62
maker-VaccDscaff37-snap-gene-285.29-mRNA-1	tr|A4ZKI0|A	56.62
augustus_masked-VaccDscaff6-processed-gene-398.6-mRNA	tr|A4ZKI0|A	56.39
augustus_masked-VaccDscaff38-processed-gene-5.5-mRNA-1	tr|A4ZKI0|A	56.31
maker-VaccDscaff8-snap-gene-353.28-mRNA-1	tr|A4ZKI0|A	56.21
maker-VaccDscaff6-snap-gene-399.34-mRNA-1	tr|A4ZKI0|A	56.14
maker-VaccDscaff1-augustus-gene-69.29-mRNA-1	tr|A4ZKI0|A	56.04
maker-VaccDscaff37-snap-gene-281.44-mRNA-1	tr|A4ZKI0|A	55.94
augustus_masked-VaccDscaff39-processed-gene-45.1-mRNA	tr|A4ZKI0|A	55.94
maker-VaccDscaff39-snap-gene-47.33-mRNA-1	tr|A4ZKI0|A	55.94
maker-VaccDscaff37-snap-gene-282.25-mRNA-1	tr|A4ZKI0|A	55.91
augustus_masked-VaccDscaff38-processed-gene-6.0-mRNA-1	tr|A4ZKI0|A	55.71
maker-VaccDscaff1-augustus-gene-70.25-mRNA-1	tr|A4ZKI0|A	55.28
maker-VaccDscaff38-snap-gene-4.32-mRNA-1	tr|A4ZKI0|A	53.78
augustus_masked-VaccDscaff39-processed-gene-46.0-mRNA	tr|A4ZKI0|A	53.78
augustus_masked-VaccDscaff6-processed-gene-400.9-mRNA	tr|A4ZKI0|A	53.55

augustus_masked-VaccDscaff38-processed-gene-5.3-mRNA-1	tr|A4ZKE4|A	52.45
maker-VaccDscaff37-augustus-gene-283.26-mRNA-1	tr|A4ZKI0|A4	51.26
maker-VaccDscaff56-snap-gene-3.55-mRNA-1	tr|A4ZKE4|A	44.65
maker-VaccDscaff38-augustus-gene-3.29-mRNA-1	tr|A4ZKE4|A	42.57
maker-VaccDscaff6-augustus-gene-402.28-mRNA-1	tr|A4ZKE4|A	42.57
maker-VaccDscaff413-augustus-gene-0.23-mRNA-1	tr|A4ZKE4|A	42.57
maker-VaccDscaff6-snap-gene-404.28-mRNA-1	tr|A4ZKE4|A	42.41
maker-VaccDscaff39-augustus-gene-43.27-mRNA-1	tr|A4ZKE4|A	42.41
augustus_masked-VaccDscaff37-processed-gene-285.4-mRNA	tr|A4ZKE4|A	42.19
maker-VaccDscaff38-augustus-gene-0.18-mRNA-1	tr|A4ZKE4|A	41.69
maker-VaccDscaff39-augustus-gene-42.23-mRNA-1	tr|A4ZKE4|A	41.52
4-coumaric acid 3`-hydroxylase (C3H)		
maker-VaccDscaff1-augustus-gene-373.37-mRNA-1	tr|J7FTV2|J7	81.14
maker-VaccDscaff1-snap-gene-373.42-mRNA-1	sp|O22203|(	71.43
maker-VaccDscaff1-snap-gene-373.43-mRNA-1	tr|J7FTV2|J7	75.96
maker-VaccDscaff10-snap-gene-65.37-mRNA-1	tr|J7FTV2|J7	80.93
maker-VaccDscaff10-snap-gene-65.38-mRNA-1	tr|J7FTV2|J7	70.88
maker-VaccDscaff19-augustus-gene-164.33-mRNA-1	tr|J7FTV2|J7	74.95
maker-VaccDscaff38-augustus-gene-5.28-mRNA-1	tr|J7FTV2|J7	75.66
maker-VaccDscaff39-augustus-gene-47.30-mRNA-1	tr|J7FTV2|J7	75.66
maker-VaccDscaff5-augustus-gene-352.30-mRNA-1	sp|O22203|(	73.49
maker-VaccDscaff5-snap-gene-352.42-mRNA-1	tr|J7FTV2|J7	80.93
maker-VaccDscaff6-snap-gene-399.30-mRNA-1	tr|J7FTV2|J7	76.06
maker-VaccDscaff8-augustus-gene-79.42-mRNA-1	tr|J7FTV2|J7	76.36
maker-VaccDscaff8-augustus-gene-79.43-mRNA-1	sp|O22203|(	75.37
maker-VaccDscaff8-snap-gene-79.46-mRNA-1	tr|J7FTV2|J7	80.93
Hydroxycinnamoyl-CoA quinate hydroxycinnamoyltransferase (HQT)		
maker-VaccDscaff39-augustus-gene-43.31-mRNA-1	tr|A4ZKM4|A	56.95
maker-VaccDscaff39-augustus-gene-44.31-mRNA-1	tr|A4ZKM4|A	54.97
maker-VaccDscaff38-augustus-gene-2.44-mRNA-1	tr|A4ZKM4|A	50.13
Chalcone synthase (CHS)		
maker-VaccDscaff13-augustus-gene-46.32-mRNA-1	NP_001267	92.53
maker-VaccDscaff32-augustus-gene-318.21-mRNA-1	NP_001267	92.53
maker-VaccDscaff30-augustus-gene-303.29-mRNA-1	NP_001267	92.27
maker-VaccDscaff42-augustus-gene-14.30-mRNA-1	NP_001267	92.27
maker-VaccDscaff25-augustus-gene-174.22-mRNA-1	AGE84303.1	91.99
maker-VaccDscaff3-augustus-gene-334.17-mRNA-1	AGE84303.1	91.99
maker-VaccDscaff25-augustus-gene-173.26-mRNA-1	AGE84303.1	91.73
maker-VaccDscaff2-augustus-gene-67.14-mRNA-1	AGE84303.1	91.47
maker-VaccDscaff36-augustus-gene-240.30-mRNA-1	AGE84303.1	91.21
augustus_masked-VaccDscaff9-processed-gene-64.0-mRNA-1	AGE84303.1	91.21
maker-VaccDscaff35-augustus-gene-1.21-mRNA-1	AGE84303.1	91.21
augustus_masked-VaccDscaff4-processed-gene-104.3-mRNA	AGE84303.1	91.21

maker-VaccDscaff2-augustus-gene-236.18-mRNA-1	AGE84303.1	86.05
augustus_masked-VaccDscaff12-processed-gene-318.4-mRN	NP_001267	62.39
maker-VaccDscaff41-snap-gene-198.34-mRNA-1	NP_001267	60.29
augustus_masked-VaccDscaff23-processed-gene-74.2-mRNA	NP_001267	60.24
maker-VaccDscaff41-snap-gene-198.39-mRNA-1	NP_001267	60.23
maker-VaccDscaff23-snap-gene-74.34-mRNA-1	NP_001267	60.05
maker-VaccDscaff12-snap-gene-319.33-mRNA-1	NP_001267	59.95
maker-VaccDscaff40-snap-gene-213.40-mRNA-1	NP_001267	57.22

Chalcone--flavonone isomerase (CHI)

augustus_masked-VaccDscaff29-processed-gene-189.2-mRN	sp|A5ANT9|	74.89
maker-VaccDscaff26-snap-gene-196.29-mRNA-1	sp|A5ANT9|	74.43
augustus_masked-VaccDscaff21-processed-gene-199.1-mRN	sp|A5ANT9|	73.97
snap_masked-VaccDscaff33-processed-gene-155.13-mRNA-1	sp|A5ANT9|	66.53
maker-VaccDscaff3-snap-gene-284.20-mRNA-1	sp|A5ANT9|	60.66
maker-VaccDscaff2-augustus-gene-128.30-mRNA-1	sp|A5ANT9|	59.32
maker-VaccDscaff25-augustus-gene-101.12-mRNA-1	sp|A5ANT9|	57.76

Flavanone 3beta-hydroxylase (FHT)

maker-VaccDscaff32-augustus-gene-323.35-mRNA-1	AAC49929.1	84.97
maker-VaccDscaff30-augustus-gene-297.38-mRNA-1	AAC49929.1	84.97
augustus_masked-VaccDscaff42-processed-gene-20.3-mRNA	AAC49929.1	84.97
maker-VaccDscaff31-augustus-gene-324.32-mRNA-1	AAP57394.1	84.91
maker-VaccDscaff13-augustus-gene-41.36-mRNA-1	AAC49929.1	84.7
maker-VaccDscaff16-augustus-gene-381.32-mRNA-1	AAP57394.1	84.32
snap_masked-VaccDscaff7-processed-gene-48.23-mRNA-1	AAP57394.1	83.02
snap_masked-VaccDscaff18-processed-gene-15.24-mRNA-1	AAP57394.1	82.76

Flavonol synthase (FLS)

maker-VaccDscaff6-snap-gene-182.24-mRNA-1	sp|Q96330|I	67.16
maker-VaccDscaff25-augustus-gene-223.22-mRNA-1	sp|Q96330|I	66.37
maker-VaccDscaff25-augustus-gene-225.24-mRNA-1	sp|Q96330|I	66.37
augustus_masked-VaccDscaff3-processed-gene-255.8-mRNA	sp|Q96330|I	66.37
maker-VaccDscaff14-augustus-gene-146.15-mRNA-1	sp|Q96330|I	66.17
maker-VaccDscaff6-augustus-gene-212.24-mRNA-1	sp|Q96330|I	62.84
maker-VaccDscaff37-augustus-gene-121.34-mRNA-1	sp|Q96330|I	62.84
maker-VaccDscaff37-snap-gene-115.28-mRNA-1	sp|Q96330|I	62.54
maker-VaccDscaff38-augustus-gene-187.30-mRNA-1	sp|Q96330|I	62.24
maker-VaccDscaff39-augustus-gene-206.36-mRNA-1	sp|Q96330|I	62.24
maker-VaccDscaff37-augustus-gene-93.33-mRNA-1	sp|Q96330|I	61.93
maker-VaccDscaff6-augustus-gene-163.26-mRNA-1	sp|Q96330|I	48.66
maker-VaccDscaff37-augustus-gene-81.25-mRNA-1	sp|Q96330|I	48.66
maker-VaccDscaff38-augustus-gene-200.23-mRNA-1	sp|Q96330|I	48.36
maker-VaccDscaff39-augustus-gene-226.23-mRNA-1	sp|Q96330|I	48.06

Flavonoid 3'-hydroxylase (F3'H)

maker-VaccDscaff13-augustus-gene-238.27-mRNA-1	BAE47006.1	80.54
maker-VaccDscaff30-augustus-gene-161.25-mRNA-1	BAE47006.1	80.35
maker-VaccDscaff42-augustus-gene-154.36-mRNA-1	BAE47006.1	80.35
maker-VaccDscaff32-augustus-gene-159.26-mRNA-1	BAE47006.1	79.58
Flavonoid 3',5'-hydroxylase (F3'5'H)		
maker-VaccDscaff46-snap-gene-18.55-mRNA-1	XP_020419	72.33
maker-VaccDscaff22-augustus-gene-138.42-mRNA-1	XP_020419	71.77
maker-VaccDscaff43-snap-gene-230.38-mRNA-1	XP_020419	67.42
snap_masked-VaccDscaff22-processed-gene-138.31-mRNA-1	XP_020419	67.2
augustus_masked-VaccDscaff43-processed-gene-231.0-mRN	XP_020419	64.15
augustus_masked-VaccDscaff22-processed-gene-138.4-mRN	XP_020419	61.38
maker-VaccDscaff46-snap-gene-18.56-mRNA-1	XP_020419	59.48
maker-VaccDscaff22-snap-gene-138.49-mRNA-1	XP_020419	55.43
maker-VaccDscaff43-augustus-gene-230.31-mRNA-1	XP_020419	55.07
augustus_masked-VaccDscaff22-processed-gene-126.2-mRN	XP_020419	50.75
maker-VaccDscaff43-augustus-gene-231.35-mRNA-1	XP_020419	50
maker-VaccDscaff22-augustus-gene-138.38-mRNA-1	XP_020419	48.33
Dihydroflavonol reductase (DFR)		
maker-VaccDscaff30-augustus-gene-208.37-mRNA-1	AFF60412.1	79.34
augustus_masked-VaccDscaff13-processed-gene-166.8-mRN	CAA53578.1	77.88
augustus_masked-VaccDscaff32-processed-gene-199.0-mRN	CAA53578.1	73.85
augustus_masked-VaccDscaff42-processed-gene-96.9-mRNA	CAA53578.1	73.85
maker-VaccDscaff12-snap-gene-66.28-mRNA-1	CAA53578.1	66.87
augustus_masked-VaccDscaff1613-processed-gene-0.0-mRN	CAA53578.1	65.96
Leucoanthocyanidin reductase (LAR)		
maker-VaccDscaff14-augustus-gene-252.26-mRNA-1	BAM42675.	99.72
maker-VaccDscaff25-augustus-gene-293.25-mRNA-1	BAM42675.	99.43
maker-VaccDscaff2-augustus-gene-298.20-mRNA-1	BAM42675.	99.43
maker-VaccDscaff3-augustus-gene-115.16-mRNA-1	BAM42675.	99.43
maker-VaccDscaff28-augustus-gene-227.23-mRNA-1	BAM42675.	77.45
maker-VaccDscaff20-augustus-gene-119.25-mRNA-1	BAM42675.	77.15
maker-VaccDscaff19-augustus-gene-121.20-mRNA-1	BAM42675.	77.15
maker-VaccDscaff28-augustus-gene-232.26-mRNA-1	BAM42675.	76.85
maker-VaccDscaff4-augustus-gene-340.14-mRNA-1	XP_00722	70.88
maker-VaccDscaff9-augustus-gene-315.13-mRNA-1	AAZ82411.1	67.63
maker-VaccDscaff36-augustus-gene-103.16-mRNA-1	AAZ82411.1	66.38
maker-VaccDscaff35-augustus-gene-247.32-mRNA-1	AAZ82411.1	65.61
Anthocyanidin synthase (ANS)		
maker-VaccDscaff22-augustus-gene-131.24-mRNA-1	ABV82967.1	81.13
maker-VaccDscaff46-augustus-gene-11.23-mRNA-1	AQP31154.1	78.41
maker-VaccDscaff43-augustus-gene-236.29-mRNA-1	AQP31154.1	78.12

Anthocyanidin reductase (ANR)		
maker-VaccDscaff15-augustus-gene-178.21-mRNA-1	Q9SEV0	BAN_ARAT
maker-VaccDscaff19-augustus-gene-256.19-mRNA-1	Q9SEV0	BAN_ARAT
maker-VaccDscaff133-augustus-gene-0.14-mRNA-1	Q9SEV0	BAN_ARAT
maker-VaccDscaff24-augustus-gene-201.24-mRNA-1	Q9SEV0	BAN_ARAT
UDP glucose:flavonoid 3-O-glucosyl transferase (UFGT)		
maker-VaccDscaff48-snap-gene-55.35-mRNA-1	AHL45018.1	52.83
maker-VaccDscaff28-augustus-gene-290.32-mRNA-1	AHL45018.1	52.48
maker-VaccDscaff6-augustus-gene-420.36-mRNA-1	AHL45018.1	51.75
maker-VaccDscaff37-augustus-gene-300.34-mRNA-1	AHL45018.1	51.75
augustus_masked-VaccDscaff39-processed-gene-2.0-mRNA-1	AHL45018.1	51.75
maker-VaccDscaff20-augustus-gene-68.36-mRNA-1	AAS00612.2	50.75
maker-VaccDscaff20-augustus-gene-68.33-mRNA-1	AHL45018.1	50.53
maker-VaccDscaff28-augustus-gene-290.34-mRNA-1	AHL45018.1	50.53
maker-VaccDscaff38-augustus-gene-9.29-mRNA-1	AAS00612.2	50.32
maker-VaccDscaff34-augustus-gene-294.28-mRNA-1	AHL45018.1	50.21
maker-VaccDscaff34-augustus-gene-294.29-mRNA-1	AHL45018.1	50
maker-VaccDscaff20-augustus-gene-68.35-mRNA-1	AHL45018.1	49.25
snap_masked-VaccDscaff20-processed-gene-68.12-mRNA-1	AHL45018.1	48.63
maker-VaccDscaff6-augustus-gene-420.31-mRNA-1	AHL45018.1	48.16
maker-VaccDscaff39-augustus-gene-2.34-mRNA-1	AHL45018.1	48.16
augustus_masked-VaccDscaff17-processed-gene-66.1-mRNA	AAS00612.2	48.09
maker-VaccDscaff37-augustus-gene-300.29-mRNA-1	AHL45018.1	47.95
Anthocyanin O-methyltransferase (OMT)		
maker-VaccDscaff13-snap-gene-341.30-mRNA-1	ACO52469.	59.31
augustus_masked-VaccDscaff32-processed-gene-21.11-mRN	ACO52469.	59.31
augustus_masked-VaccDscaff30-processed-gene-58.9-mRNA	ACO52469.	59.31
maker-VaccDscaff32-snap-gene-23.20-mRNA-1	AIS23587.1	59.17
maker-VaccDscaff32-augustus-gene-21.27-mRNA-1	ACO52469.	58.87
maker-VaccDscaff30-augustus-gene-59.19-mRNA-1	ACO52469.	58.87
augustus_masked-VaccDscaff42-processed-gene-262.6-mRN	ACO52469.	58.87
maker-VaccDscaff42-augustus-gene-261.27-mRNA-1	AIS23587.1	57.34
snap_masked-VaccDscaff13-processed-gene-341.19-mRNA-1	AIS23587.1	56.58
maker-VaccDscaff45-snap-gene-216.26-mRNA-1	ACO52469.	55.02
maker-VaccDscaff17-augustus-gene-235.23-mRNA-1	ACO52469.	55.02
maker-VaccDscaff374-snap-gene-0.33-mRNA-1	ACO52469.	55.02
maker-VaccDscaff34-snap-gene-139.31-mRNA-1	ACO52469.	54.59
maker-VaccDscaff30-snap-gene-57.21-mRNA-1	ACO52469.	53.07
Transparent testa 12 (TT12)		
maker-VaccDscaff2-augustus-gene-227.19-mRNA-1	E3SHE0_M	76.69
maker-VaccDscaff3-snap-gene-209.28-mRNA-1	E3SHE0_M	80.43
maker-VaccDscaff14-augustus-gene-186.29-mRNA-1	E3SHE0_M	80.24
maker-VaccDscaff72-snap-gene-2.46-mRNA-1	E3SHE0_M	80.24

maker-VaccDscaff15-augustus-gene-107.18-mRNA-1	E3SHE2_M.	67.72
maker-VaccDscaff24-augustus-gene-293.28-mRNA-1	E3SHE2_M.	73.31
maker-VaccDscaff24-augustus-gene-293.29-mRNA-1	E3SHE2_M.	72.71
maker-VaccDscaff11-snap-gene-112.24-mRNA-1	E3SHE2_M.	67.53
maker-VaccDscaff19-snap-gene-200.32-mRNA-1	E3SHE2_M.	61.75
maker-VaccDscaff11-augustus-gene-112.19-mRNA-1	E3SHE2_M.	72.65
maker-VaccDscaff19-snap-gene-200.30-mRNA-1	E3SHE2_M.	66.67
maker-VaccDscaff37-snap-gene-24.28-mRNA-1	E3SHE2_M.	49.08
maker-VaccDscaff37-snap-gene-25.36-mRNA-1	E3SHE2_M.	49.08
maker-VaccDscaff39-augustus-gene-272.23-mRNA-1	E3SHE2_M.	49.08
maker-VaccDscaff6-snap-gene-102.18-mRNA-1	E3SHE2_M.	49.38
maker-VaccDscaff39-snap-gene-272.29-mRNA-1	E3SHE2_M.	49.38
maker-VaccDscaff4-augustus-gene-170.22-mRNA-1	E3SHE2_M.	49.18
maker-VaccDscaff37-snap-gene-24.29-mRNA-1	E3SHE2_M.	48.97
maker-VaccDscaff12-augustus-gene-77.23-mRNA-1	A0A1U7VQ'	52.67
maker-VaccDscaff4-snap-gene-322.31-mRNA-1	A0A1U7VQ'	59.62
maker-VaccDscaff36-snap-gene-121.31-mRNA-1	A0A1U7VQ'	59.38
maker-VaccDscaff20-augustus-gene-17.30-mRNA-1	A0A1U7VQ'	50
maker-VaccDscaff28-augustus-gene-334.35-mRNA-1	A0A1U7VQ'	50
maker-VaccDscaff11-augustus-gene-14.31-mRNA-1	A0A1U7VQ'	59.23
maker-VaccDscaff40-augustus-gene-9.29-mRNA-1	A0A1U7VQ'	57.55
maker-VaccDscaff9-augustus-gene-258.23-mRNA-1	A0A1U7VQ'	60.48
maker-VaccDscaff35-snap-gene-193.37-mRNA-1	A0A1U7VQ'	59.95
maker-VaccDscaff36-snap-gene-161.33-mRNA-1	A0A1U7VQ'	59.71
maker-VaccDscaff35-augustus-gene-228.24-mRNA-1	A0A1U7VQ'	55.72
maker-VaccDscaff35-snap-gene-193.37-mRNA-1	A0A1U7VQ'	61.27
maker-VaccDscaff28-snap-gene-334.43-mRNA-1	A0A1U7VQ'	52.35
maker-VaccDscaff20-snap-gene-17.42-mRNA-1	A0A1U7VQ'	52.1
maker-VaccDscaff19-snap-gene-16.34-mRNA-1	A0A1U7VQ'	51.36
maker-VaccDscaff4-snap-gene-292.40-mRNA-1	A0A1U7VQ'	57.96
maker-VaccDscaff15-augustus-gene-108.25-mRNA-1	E3SHE2_M.	75.81
maker-VaccDscaff44-snap-gene-234.34-mRNA-1	A0A1U7VQ'	62
maker-VaccDscaff23-augustus-gene-333.30-mRNA-1	A0A1U7VQ'	57.5
maker-VaccDscaff36-snap-gene-161.33-mRNA-1	A0A1U7VQ'	55
maker-VaccDscaff15-augustus-gene-107.19-mRNA-1	E3SHE2_M.	75.69
maker-VaccDscaff4-snap-gene-292.40-mRNA-1	A0A1U7VQ'	58.94
maker-VaccDscaff36-snap-gene-159.21-mRNA-1	A0A1U7VQ'	57.18
maker-VaccDscaff36-snap-gene-161.34-mRNA-1	A0A1U7VQ'	55.16
maker-VaccDscaff35-snap-gene-193.35-mRNA-1	A0A1U7VQ'	54.16
maker-VaccDscaff9-snap-gene-257.32-mRNA-1	A0A1U7VQ'	50.88
maker-VaccDscaff4-snap-gene-292.41-mRNA-1	A0A1U7VQ'	61.52
maker-VaccDscaff2-augustus-gene-421.33-mRNA-1	A0A1U7VQ'	55.08
maker-VaccDscaff28-snap-gene-334.43-mRNA-1	A0A1U7VQ'	52.81
maker-VaccDscaff39-snap-gene-272.27-mRNA-1	E3SHE2_M.	52.66
maker-VaccDscaff4-augustus-gene-291.30-mRNA-1	A0A1U7VQ'	66.77
maker-VaccDscaff15-augustus-gene-26.39-mRNA-1	A0A1U7VQ'	64.78

maker-VaccDscaff48-augustus-gene-94.41-mRNA-1	A0A1U7VQ\	50.17
maker-VaccDscaff3-snap-gene-209.28-mRNA-1	E3SHE0_M/	62.06
maker-VaccDscaff48-snap-gene-94.51-mRNA-1	A0A1U7VQ\	52.13
maker-VaccDscaff2-snap-gene-227.26-mRNA-1	E3SHE2_M/	74.06
maker-VaccDscaff24-snap-gene-183.28-mRNA-1	A0A1I9LM1	53.01
snap_masked-VaccDscaff9-processed-gene-261.8-mRNA-1	A0A1U7VQ\	72.83

Transparent testa 9 (TT19)

maker-VaccDscaff13-augustus-gene-26.16-mRNA-1	GSTFC_AR	60.19
maker-VaccDscaff30-augustus-gene-282.25-mRNA-1	GSTFC_AR	59.72
maker-VaccDscaff42-augustus-gene-34.23-mRNA-1	GSTFC_AR	47.91
maker-VaccDscaff2-augustus-gene-225.25-mRNA-1	GSTFC_AR	47.37
maker-VaccDscaff72-augustus-gene-0.26-mRNA-1	GSTFC_AR	47.37
maker-VaccDscaff3-augustus-gene-210.28-mRNA-1	GSTFC_AR	47.37
maker-VaccDscaff14-augustus-gene-185.29-mRNA-1	GSTFC_AR	46.89
maker-VaccDscaff7-augustus-gene-2.31-mRNA-1	GSTFC_AR	44.71
maker-VaccDscaff18-augustus-gene-12.28-mRNA-1	GSTFC_AR	44.23
maker-VaccDscaff16-augustus-gene-385.29-mRNA-1	GSTFC_AR	43.75
maker-VaccDscaff31-augustus-gene-329.24-mRNA-1	GSTFC_AR	43.75
maker-VaccDscaff15-snap-gene-378.50-mRNA-1	GSTFC_AR	41.82
maker-VaccDscaff11-snap-gene-386.61-mRNA-1	GSTFC_AR	41.31

b. Ascorbic acid biosynthesis related genes

l-galactose-1-phosphate phosphatase (GPP) or inositol monophosphatase

maker-VaccDscaff11-augustus-gene-253.33-mRNA-1	AAV49506.1	90
maker-VaccDscaff176-snap-gene-1.80-mRNA-1	AAV49506.1	90
maker-VaccDscaff9-snap-gene-116.36-mRNA-1	AAV49506.1	89.85
maker-VaccDscaff35-augustus-gene-72.36-mRNA-1	AAV49506.1	89.63
maker-VaccDscaff4-snap-gene-162.42-mRNA-1	AAV49506.1	89.62
maker-VaccDscaff30-snap-gene-118.40-mRNA-1	G8GJD2_BI	68.57
maker-VaccDscaff42-snap-gene-193.40-mRNA-1	G8GJD2_BI	68
maker-VaccDscaff32-snap-gene-92.47-mRNA-1	G8GJD2_BI	67.94
maker-VaccDscaff30-snap-gene-120.42-mRNA-1	G8GJD2_BI	66.86
maker-VaccDscaff42-snap-gene-193.39-mRNA-1	G8GJD2_BI	66.5
maker-VaccDscaff13-augustus-gene-280.32-mRNA-1	G8GJD2_BI	61.2
augustus_masked-VaccDscaff30-processed-gene-118.1-mRN/	G8GJD2_BI	60.88
augustus_masked-VaccDscaff42-processed-gene-194.3-mRN/	G8GJD2_BI	60.88

GDP-l-galactose phosphorylase (GGP)

maker-VaccDscaff93-augustus-gene-2.31-mRNA-1	D3JYW8_A(	84.84
maker-VaccDscaff6-augustus-gene-38.29-mRNA-1	D3JYW8_A(	84.4
maker-VaccDscaff80-augustus-gene-5.36-mRNA-1	D3JYW8_A(	84.18
maker-VaccDscaff16-augustus-gene-117.32-mRNA-1	A0A1S3BIN	65.68
maker-VaccDscaff31-snap-gene-26.35-mRNA-1	A0A1S3BIN	64.76

GDP-d-mannose-3',5'-epimerase (GME)

maker-VaccDscaff1397-augustus-gene-0.2-mRNA-1	B6ZL92_PR	95.48
maker-VaccDscaff9-augustus-gene-34.28-mRNA-1	B6ZL92_PR	95.09
augustus_masked-VaccDscaff36-processed-gene-277.9-mRN/	B6ZL92_PR	94.43
maker-VaccDscaff9-augustus-gene-34.26-mRNA-1	B6ZL92_PR	94.43
maker-VaccDscaff35-augustus-gene-30.30-mRNA-1	B6ZL92_PR	94.43
maker-VaccDscaff4-augustus-gene-58.31-mRNA-1	B6ZL92_PR	94.43

GDP-d-mannose pyrophosphorylase (GMP) or mannose-1-phosphate guanylyltran

maker-VaccDscaff29-snap-gene-140.35-mRNA-1	C0KWD9_9	96.38
maker-VaccDscaff21-augustus-gene-140.35-mRNA-1	C0KWD9_9	95.01
maker-VaccDscaff26-snap-gene-143.37-mRNA-1	C0KWD9_9	95.01
maker-VaccDscaff19-snap-gene-49.37-mRNA-1	C0KWD9_9	95
maker-VaccDscaff29-snap-gene-140.35-mRNA-1	C0KWD9_9	95
maker-VaccDscaff48-snap-gene-77.28-mRNA-1	C0KWD9_9	95
maker-VaccDscaff28-augustus-gene-305.39-mRNA-1	C0KWD9_9	94.74
maker-VaccDscaff20-snap-gene-48.42-mRNA-1	C0KWD9_9	94.17
snap_masked-VaccDscaff44-processed-gene-39.18-mRNA-1	C0KWD9_9	87.61
snap_masked-VaccDscaff28-processed-gene-49.17-mRNA-1	C0KWD9_9	87.61
maker-VaccDscaff20-augustus-gene-320.37-mRNA-1	C0KWD9_9	66.54
maker-VaccDscaff49-snap-gene-43.39-mRNA-1	C0KWD9_9	61.09

l-galactose dehydrogenase (GDH)

maker-VaccDscaff16-snap-gene-57.31-mRNA-1	GALDH_AR	84.36
maker-VaccDscaff18-augustus-gene-342.40-mRNA-1	GALDH_AR	83.7
maker-VaccDscaff3-augustus-gene-23.19-mRNA-1	GALDH_AR	83.58
maker-VaccDscaff2-augustus-gene-392.33-mRNA-1	GALDH_AR	81.65
maker-VaccDscaff7-augustus-gene-369.28-mRNA-1	GALDH_AR	81.38
maker-VaccDscaff14-augustus-gene-339.32-mRNA-1	GALDH_AR	81.33
maker-VaccDscaff7-augustus-gene-315.29-mRNA-1	V9NF86_AC	63.7
maker-VaccDscaff18-augustus-gene-277.37-mRNA-1	V9NF86_AC	63.7
maker-VaccDscaff34-snap-gene-271.46-mRNA-1	GALDH_AR	59.46
maker-VaccDscaff45-augustus-gene-47.42-mRNA-1	GALDH_AR	58.7
maker-VaccDscaff27-augustus-gene-21.36-mRNA-1	D7MGH1_A	56.31
maker-VaccDscaff17-augustus-gene-77.38-mRNA-1	D7MGH1_A	56.31

l-galactono-1,4-lactone dehydrogenase (GLDH)

maker-VaccDscaff21-snap-gene-333.63-mRNA-1	GLDH_ARA	82.18
maker-VaccDscaff29-augustus-gene-311.44-mRNA-1	GLDH_ARA	79.72
maker-VaccDscaff26-augustus-gene-325.17-mRNA-1	GLDH_ARA	79.54

nesis-related genes in blueberry.

length	mismatch	gapopen	qstart	qend	sstart	send
698	96	1	17	713	14	711
698	96	1	17	713	14	711
698	97	1	17	713	14	711
678	97	1	17	693	14	691
698	101	1	17	713	14	711
698	102	1	17	713	14	711
698	102	1	17	713	14	711
698	102	1	17	713	14	711
710	108	0	6	715	2	711
698	106	1	17	713	14	711
698	106	1	17	713	14	711
710	109	0	6	715	2	711
710	111	0	6	715	2	711
698	109	1	17	713	14	711
716	100	2	17	731	14	711
723	108	1	6	728	2	711
704	98	2	15	711	14	717
704	100	2	15	711	14	717
704	100	2	15	711	14	717
704	100	2	15	711	14	717
534	94	0	16	549	23	556
534	95	0	16	549	23	556
530	95	0	16	545	23	552
534	96	0	16	549	23	556
540	121	1	15	554	21	556
540	124	1	15	554	21	556
540	124	1	14	553	21	556
558	131	2	4	554	3	556
543	190	2	59	590	25	556
563	197	3	56	613	25	556
530	110	2	53	577	1	529
530	110	2	1	525	1	529
530	111	2	1	525	1	529
423	97	2	1	418	1	422
505	43	0	1	505	1	505
505	44	0	1	505	1	505

505	44	0	1	505	1	505
504	44	0	1	504	1	504
505	45	0	1	505	1	505
505	45	0	1	505	1	505
505	47	0	1	505	1	505
505	47	0	1	505	1	505
503	54	0	1	503	1	503
506	54	1	1	506	1	505
506	55	1	1	506	1	505
505	70	0	1	505	1	505

300	50	1	201	499	136	435
434	79	0	48	481	1	434
434	79	0	1	434	1	434
434	81	0	1	434	1	434
435	165	2	1	435	1	433
439	163	2	1	439	1	433
439	163	2	1	439	1	433
435	166	2	1	435	1	433
435	166	2	1	435	1	433
435	166	2	1	435	1	433
439	165	2	1	439	1	433
439	166	2	1	439	1	433
437	176	3	1	437	1	434
437	176	3	1	437	1	434
436	176	3	1	436	1	433
437	178	3	1	437	1	434
438	184	2	29	466	1	433
442	180	5	1	442	1	431
438	185	2	1	438	1	433
438	185	2	5	442	1	433
438	186	4	1	438	1	433
444	183	4	1	444	1	433
443	180	6	1	442	1	430
440	182	4	1	440	1	429
439	186	5	1	438	1	433
438	188	4	1	438	1	433
438	188	3	1	438	1	433
438	188	4	1	438	1	433
440	183	4	1	440	1	429
438	189	4	1	438	1	433
445	187	4	1	445	1	433
437	198	3	1	437	1	433
437	198	3	1	437	1	433
437	199	3	1	437	1	433

265	124	2	1	265	171	433
437	193	4	5	425	1	433
318	161	8	76	384	4	315
444	238	7	4	445	5	433
444	238	7	4	445	5	433
444	238	7	4	445	5	433
448	239	7	2	447	3	433
448	239	7	2	447	3	433
448	240	6	2	447	3	433
439	237	6	101	537	3	424
448	243	6	2	447	3	433
493	92	1	19	511	17	508
504	118	2	23	526	19	496
495	116	1	611	1105	17	508
493	93	1	19	511	17	508
498	118	2	540	1037	30	500
495	121	2	19	513	17	508
493	119	1	19	511	17	508
493	119	1	19	511	17	508
479	118	2	23	493	19	496
493	93	1	19	511	17	508
493	115	2	19	509	17	508
495	114	1	21	515	17	508
479	117	1	23	501	19	496
493	93	1	19	511	17	508
439	180	2	29	467	1	430
322	136	2	29	350	1	313
377	163	4	29	405	1	352
388	28	1	6	392	2	389
388	28	1	6	392	2	389
388	29	1	6	392	2	389
388	29	1	6	392	2	389
387	31	0	1	387	1	387
387	31	0	1	387	1	387
387	32	0	1	387	1	387
387	33	0	1	387	1	387
387	34	0	1	387	1	387
387	34	0	1	387	1	387
387	34	0	1	387	1	387
387	34	0	1	387	1	387

387	54	0	178	564	1	387
351	108	7	121	471	61	387
408	129	8	1	399	4	387
410	135	8	1	406	4	389
342	121	7	41	382	61	387
408	130	8	1	399	4	387
392	133	8	9	391	11	387
395	127	9	9	379	11	387
219	54	1	1	218	1	219
219	55	1	1	218	1	219
219	56	1	1	218	1	219
245	55	2	1	244	1	219
211	82	1	7	217	6	215
177	72	0	4	180	39	215
116	49	0	2	117	100	215
366	48	1	1	359	1	366
366	48	1	1	359	1	366
366	48	1	1	359	1	366
371	48	4	1	366	1	368
366	49	1	1	359	1	366
370	52	3	1	366	1	368
377	51	4	1	373	1	368
377	52	4	1	373	1	368
134	44	0	1	134	201	334
333	111	1	1	332	1	333
333	111	1	1	332	1	333
333	111	1	1	332	1	333
334	112	1	1	333	1	334
261	95	1	6	264	4	264
261	95	1	6	264	4	264
331	122	1	6	334	4	334
331	123	1	6	334	4	334
331	123	1	6	334	4	334
331	124	1	6	334	4	334
335	166	4	1	330	1	334
335	166	4	1	330	1	334
335	167	4	1	330	1	334
335	168	4	1	330	1	334

514	93	3	18	531	1	507
514	94	3	1	514	1	507
514	94	3	1	514	1	507
519	93	4	18	536	1	506

206	57	0	25	230	62	267
209	59	0	65	273	59	267
267	84	2	81	344	1	267
189	62	0	1	189	79	267
106	36	1	1	106	163	266
189	71	1	416	604	62	248
269	104	3	471	736	1	267
276	114	3	640	915	1	267
276	115	3	268	543	1	267
134	63	1	206	339	129	259
274	126	3	100	372	3	266
180	88	3	89	267	23	198

213	44	0	11	223	7	219
330	73	0	11	340	7	336
348	73	1	11	358	7	336
348	73	1	11	358	7	336
329	103	2	30	357	2	325
329	106	2	7	334	2	325

351	1	0	1	351	1	351
351	2	0	1	351	1	351
351	2	0	1	351	1	351
352	1	1	1	352	1	351
337	72	2	14	350	19	351
337	73	2	14	350	19	351
337	70	3	14	347	19	351
337	74	2	14	350	19	351
182	53	0	5	186	14	195
346	109	2	2	346	19	362
351	110	3	2	351	19	362
346	111	3	2	341	19	362

355	61	3	1	355	1	349
352	73	1	4	355	3	351
352	74	1	4	355	3	351

65.15	330	115	0	3	332	9
64.85	330	116	0	3	332	9
64.85	330	116	0	3	332	9
61.08	352	115	1	3	354	9
460	204	8	202	652	24	479
463	207	8	329	782	24	482
458	209	6	7	453	27	483
458	209	6	7	453	27	483
458	209	6	7	453	27	483
266	118	6	1	253	206	471
469	214	8	4	458	19	483
469	214	8	4	458	19	483
310	140	7	363	658	164	473
466	209	10	14	469	29	481
466	210	11	14	469	29	481
469	222	8	4	460	19	483
475	212	9	20	470	17	483
463	221	7	2	447	23	483
463	219	8	50	493	23	483
472	227	10	1	456	1	470
463	222	7	2	447	23	483
231	91	2	63	290	4	234
231	91	2	10	237	4	234
231	91	2	10	237	4	234
218	85	3	29	243	18	234
231	92	2	9	236	4	234
231	92	2	9	236	4	234
231	92	2	10	237	4	234
218	89	3	3	217	18	234
228	93	3	15	239	10	234
229	101	2	89	315	6	234
229	101	2	70	296	6	234
229	101	2	103	329	6	234
229	102	2	63	289	6	234
228	99	3	13	232	7	234
532	97	2	1	532	1	505
506	96	2	323	826	1	505
506	99	1	1	506	1	505
506	97	2	270	773	1	505

505	131	2	1	473	1	505
502	131	1	3	501	4	505
502	134	1	3	501	4	505
502	131	2	3	472	4	505
502	125	3	3	437	4	505
501	134	1	4	501	5	505
498	124	3	3	458	4	501
489	247	2	6	493	5	492
489	247	2	6	493	5	492
489	247	2	6	493	5	492
486	245	1	13	498	8	492
486	245	1	13	498	8	492
486	244	2	44	529	25	507
486	247	1	13	498	8	492
450	178	3	15	462	18	434
421	144	1	31	451	38	432
421	145	1	31	451	38	432
420	200	3	10	428	24	434
420	200	3	10	428	24	434
417	149	2	108	524	38	433
417	175	1	15	429	18	434
415	144	2	50	464	38	432
412	149	3	531	942	38	433
412	150	3	48	459	38	433
411	116	2	31	391	38	432
408	145	1	38	445	38	432
405	188	2	18	421	34	434
405	189	2	18	421	34	434
405	187	3	339	737	34	434
402	163	3	47	443	37	437
401	97	0	1	401	105	505
400	148	2	54	453	38	433
400	170	0	20	419	25	424
400	114	4	546	882	38	434
399	97	0	1	399	107	505
397	110	1	518	861	38	434
397	110	4	55	391	38	434
397	118	3	49	385	38	434
397	122	3	49	385	38	434
397	127	4	49	377	38	434
395	137	1	38	417	38	432
394	175	2	38	430	42	434
392	179	2	505	890	43	434
338	160	0	122	459	155	492
334	111	0	1	334	101	434
301	103	2	54	352	38	337

293	136	3	10	301	24	307
282	66	3	42	282	133	414
282	130	2	18	298	34	311
266	66	2	44	307	236	500
266	125	0	32	297	51	316
254	69	0	5	258	95	348
211	84	0	1	211	1	211
211	85	0	1	211	1	211
263	85	2	1	263	1	211
209	109	1	1	208	1	209
209	109	1	1	208	1	209
209	109	1	1	208	1	209
209	110	1	1	208	1	209
208	114	1	5	212	3	209
208	115	1	5	212	3	209
208	116	1	5	212	3	209
208	116	1	5	212	3	209
165	90	3	211	371	49	211
213	117	4	3	209	2	212
270	27	0	1	270	1	270
270	27	0	50	319	1	270
266	27	0	372	637	5	270
270	28	0	1	270	1	270
106	11	0	407	512	130	235
175	55	0	1	175	1	175
175	56	0	1	175	1	175
287	90	1	1	287	1	285
175	57	1	1	174	1	175
197	66	0	1	197	1	197
317	91	2	1	317	1	285
317	92	2	1	317	1	285
317	92	2	1	317	1	285
455	61	5	1	452	1	450
455	63	5	1	452	1	450
455	64	5	1	452	1	450
437	122	8	1	431	1	415
437	119	9	1	424	1	415

376	17	0	1	376	1	376
326	15	1	12	337	1	325
377	20	1	1	377	1	376
377	20	1	1	377	1	376
377	20	1	1	377	1	376
377	20	1	1	377	1	376
sferase						
138	5	0	1	138	1	138
361	18	0	1	361	1	361
361	18	0	1	361	1	361
360	18	0	174	533	2	361
360	18	0	138	497	2	361
360	18	0	142	501	2	361
361	19	0	101	461	1	361
360	21	0	122	481	2	361
113	14	0	1	113	121	233
113	14	0	1	113	121	233
254	26	4	1	195	1	254
257	30	5	30	242	47	277
179	28	0	30	208	98	276
184	30	0	25	208	94	277
274	45	0	5	278	4	277
316	58	0	5	320	4	319
145	27	0	6	150	133	277
316	59	0	5	320	4	319
460	144	6	1	447	1	450
460	144	6	1	447	1	450
222	31	2	30	192	98	319
247	34	3	87	290	98	319
222	45	3	8	177	98	319
222	45	3	8	177	98	319
522	93	0	65	586	59	580
567	99	1	63	629	59	609
567	100	1	61	627	59	609

evalue	bitscore
0	1261
0	1261
0	1261
0	1211
0	1254
0	1253
0	1253
0	1252
0	1269
0	1237
0	1245
0	1265
0	1260
0	1236
0	1242
0	1258
0	1232
0	1229
0	1229
0	1230
0	894
0	892
0	883
0	889
0	841
0	835
0	833
0	834
0	678
0	664
0	865
0	865
0	867
0	665
0	942
0	936

0	943
0	941
0	942
0	935
0	933
0	933
0	919
0	922
0	919
0	912

0	533
0	762
0	758
0	754
0	585
0	580
0	580
0	583
0	581
0	582
0	579
0	577
0	537
0	537
0	536
0	534
0	542
0	521
0	537
0	538
5.00E-179	503
0	515
7.00E-178	516
0	512
1.00E-177	499
2.00E-177	499
0	526
7.00E-179	503
2.00E-180	510
8.00E-176	495
0	513
9.00E-179	503
3.00E-179	504
2.00E-178	502

2.00E-101	298
3.00E-166	470
4.00E-82	256
3.00E-123	361
2.00E-123	362
2.00E-123	362
7.00E-125	365
1.00E-125	367
1.00E-119	352
1.00E-110	333
1.00E-118	349
0	861
0	770
0	809
0	859
0	743
0	780
0	804
0	805
0	761
0	859
0	801
0	816
0	785
0	860
0	536
1.00E-129	373
3.00E-128	372
0	746
0	746
0	743
0	744
0	751
0	751
0	749
0	747
0	747
0	747
0	747
0	747

0	708
6.00E-144	414
5.00E-168	473
4.00E-167	476
9.00E-134	385
1.00E-167	472
3.00E-157	445
7.00E-149	423
9.00E-120	336
2.00E-119	335
3.00E-119	335
4.00E-113	321
3.00E-90	261
4.00E-75	221
1.00E-44	140
0	655
0	655
0	655
0	659
0	654
0	657
0	654
0	652
2.00E-65	197
2.00E-163	456
2.00E-163	456
2.00E-163	456
1.00E-163	457
5.00E-122	354
5.00E-122	354
9.00E-157	439
1.00E-155	436
7.00E-156	437
2.00E-155	436
1.00E-115	334
2.00E-115	333
1.00E-114	332
8.00E-114	330

0	826
0	827
0	827
0	816
2.00E-117	332
1.00E-117	335
2.00E-139	392
8.00E-99	283
1.00E-47	148
1.00E-83	259
3.00E-108	338
8.00E-110	335
4.00E-113	333
2.00E-43	145
5.00E-101	296
2.00E-60	188
2.00E-130	369
0	550
0	540
0	540
2.00E-158	457
2.00E-162	454
0	723
0	723
0	722
0	719
0	555
0	552
0	545
0	552
4.00E-99	287
2.00E-172	481
5.00E-170	474
5.00E-165	461
0	585
0	588
0	588

338	2.00E-156
338	1.00E-155
338	1.00E-155
338	7.00E-151
8.00E-163	473
6.00E-162	475
7.00E-166	473
7.00E-166	473
2.00E-165	472
2.00E-89	268
6.00E-162	463
1.00E-163	468
1.00E-101	314
2.00E-146	424
7.00E-146	422
1.00E-159	457
9.00E-153	440
7.00E-152	437
7.00E-150	434
4.00E-158	453
3.00E-151	436
1.00E-96	280
2.00E-97	280
8.00E-97	279
1.00E-90	263
2.00E-96	278
2.00E-96	278
2.00E-96	278
1.00E-87	254
1.00E-91	266
1.00E-91	269
8.00E-93	271
6.00E-92	270
3.00E-92	269
7.00E-84	246
0	791
0	800
0	803
0	799

0	673
0	743
0	737
0	668
0	588
0	734
0	653
1.00E-161	464
3.00E-163	468
9.00E-162	464
1.00E-164	472
6.00E-166	475
9.00E-161	463
7.00E-163	468
3.00E-143	417
4.00E-173	489
3.00E-171	486
9.00E-141	408
1.00E-140	407
1.00E-167	479
1.00E-158	455
9.00E-173	490
7.00E-165	486
4.00E-165	485
1.00E-149	427
2.00E-168	496
8.00E-135	407
5.00E-133	402
6.00E-126	390
4.00E-152	451
0	628
2.00E-173	490
1.00E-152	437
4.00E-131	396
0	623
3.00E-149	443
4.00E-143	412
6.00E-139	401
9.00E-137	396
5.00E-123	360
2.00E-171	485
3.00E-142	412
2.00E-129	393
5.00E-121	358
2.00E-156	444
7.00E-127	370

2.00E-92	276
2.00E-101	318
4.00E-92	275
1.00E-136	394
3.00E-94	280
7.00E-124	357
1.00E-95	273
9.00E-95	271
2.00E-81	239
8.00E-70	207
8.00E-70	207
1.00E-69	206
9.00E-69	204
7.00E-64	192
5.00E-63	190
5.00E-62	187
3.00E-62	188
2.00E-39	135
1.00E-50	165
0	504
0	504
0	511
0	509
4.00E-62	201
4.00E-87	253
4.00E-87	253
1.00E-146	409
1.00E-82	242
7.00E-94	272
7.00E-139	391
9.00E-137	385
9.00E-137	385
0	770
0	765
0	763
0	573
0	557

0	755
0	655
0	746
0	746
0	749
0	747
5.00E-89	272
0	708
0	708
0	708
0	707
0	706
0	709
0	703
1.00E-68	210
1.00E-68	211
6.00E-105	305
3.00E-92	271
2.00E-110	315
8.00E-114	324
5.00E-174	481
0	541
3.00E-84	246
0	537
0	575
0	576
2.00E-81	240
6.00E-89	263
1.00E-77	230
1.00E-77	230
0	907
0	942
0	941

Click here to download Table Extended_Data_Table4.xlsx

Extended Data Table 4.Transcription factors in blueberry.

Transcription facto	*A. thaliana* Gene ID	*V. corymbosum* Gene ID
ERF	AT1G01250.1	augustus_masked-VaccDscaff22-processed-gene
bHLH	AT1G01260.3	augustus_masked-VaccDscaff22-processed-gene
C3H	AT1G01350.1	maker-VaccDscaff22-augustus-gene-6.32-mRNA-
C3H	AT1G01350.1	maker-VaccDscaff20-augustus-gene-338.26-mRN
C3H	AT1G01350.1	maker-VaccDscaff40-augustus-gene-196.23-mRN
C3H	AT1G01350.1	maker-VaccDscaff41-snap-gene-184.34-mRNA-1
C3H	AT1G01350.1	maker-VaccDscaff44-snap-gene-27.25-mRNA-1
C3H	AT1G01350.1	maker-VaccDscaff23-augustus-gene-89.29-mRNA
C3H	AT1G01350.1	maker-VaccDscaff28-snap-gene-35.32-mRNA-1
MYB_related	AT1G01380.1	maker-VaccDscaff1069-augustus-gene-0.8-mRNA
NAC	AT1G01720.1	maker-VaccDscaff38-augustus-gene-136.22-mRN
NAC	AT1G01720.1	maker-VaccDscaff25-augustus-gene-212.27-mRN
NAC	AT1G01720.1	maker-VaccDscaff6-augustus-gene-284.24-mRNA
NAC	AT1G01720.1	maker-VaccDscaff2-augustus-gene-198.27-mRNA
NAC	AT1G01720.1	maker-VaccDscaff14-augustus-gene-155.31-mRN
NAC	AT1G01720.1	maker-VaccDscaff37-augustus-gene-172.29-mRN
NAC	AT1G01720.1	maker-VaccDscaff39-augustus-gene-142.20-mRN
NAC	AT1G01720.1	maker-VaccDscaff3-augustus-gene-244.22-mRNA
SBP	AT1G02065.1	maker-VaccDscaff22-augustus-gene-46.36-mRNA
C3H	AT1G03790.1	augustus_masked-VaccDscaff10-processed-gene
C3H	AT1G03790.1	augustus_masked-VaccDscaff8-processed-gene-
C3H	AT1G03790.1	augustus_masked-VaccDscaff5-processed-gene-
C3H	AT1G03790.1	augustus_masked-VaccDscaff1-processed-gene-
bZIP	AT1G03970.1	maker-VaccDscaff14-snap-gene-321.47-mRNA-1
bZIP	AT1G03970.1	maker-VaccDscaff3-snap-gene-37.43-mRNA-1
HD-ZIP	AT1G05230.3	maker-VaccDscaff8-augustus-gene-92.25-mRNA-
HD-ZIP	AT1G05230.3	maker-VaccDscaff8-snap-gene-89.29-mRNA-1
HD-ZIP	AT1G05230.3	augustus_masked-VaccDscaff15-processed-gene
HD-ZIP	AT1G05230.3	augustus_masked-VaccDscaff44-processed-gene
bHLH	AT1G05710.5	maker-VaccDscaff9-snap-gene-103.23-mRNA-1
bHLH	AT1G05710.5	maker-VaccDscaff35-snap-gene-59.22-mRNA-1
bHLH	AT1G05710.5	maker-VaccDscaff4-snap-gene-150.29-mRNA-1
DBB	AT1G06040.1	maker-VaccDscaff25-augustus-gene-108.26-mRN
DBB	AT1G06040.1	maker-VaccDscaff36-augustus-gene-211.32-mRN
DBB	AT1G06040.1	maker-VaccDscaff2-augustus-gene-122.33-mRNA
DBB	AT1G06040.1	maker-VaccDscaff9-augustus-gene-96.36-mRNA-
DBB	AT1G06040.1	maker-VaccDscaff35-augustus-gene-50.32-mRNA
DBB	AT1G06040.1	maker-VaccDscaff3-augustus-gene-277.27-mRNA
bZIP	AT1G06070.1	maker-VaccDscaff25-augustus-gene-100.21-mRN
bZIP	AT1G06070.1	maker-VaccDscaff2-augustus-gene-128.34-mRNA
bZIP	AT1G06070.1	maker-VaccDscaff3-augustus-gene-285.16-mRNA
bZIP	AT1G06850.1	snap_masked-VaccDscaff25-processed-gene-11[illegible]

bZIP	AT1G06850.1	maker-VaccDscaff3-augustus-gene-273.27-mRN/
GRAS	AT1G07530.1	augustus_masked-VaccDscaff22-processed-gene
GRAS	AT1G07530.1	maker-VaccDscaff43-snap-gene-104.43-mRNA-1
GRAS	AT1G07530.1	maker-VaccDscaff43-snap-gene-104.43-mRNA-1
GRAS	AT1G07530.1	augustus_masked-VaccDscaff43-processed-gene
GRAS	AT1G07530.1	augustus_masked-VaccDscaff21-processed-gene
GRAS	AT1G07530.1	augustus_masked-VaccDscaff33-processed-gene
GRAS	AT1G07530.1	augustus_masked-VaccDscaff11-processed-gene
GRAS	AT1G07530.1	augustus_masked-VaccDscaff11-processed-gene
GRAS	AT1G07530.1	augustus_masked-VaccDscaff11-processed-gene
GRAS	AT1G07530.1	augustus_masked-VaccDscaff11-processed-gene
GRAS	AT1G07530.1	augustus_masked-VaccDscaff11-processed-gene
GRAS	AT1G07530.1	augustus_masked-VaccDscaff26-processed-gene
GRAS	AT1G07530.1	snap_masked-VaccDscaff46-processed-gene-135
GRAS	AT1G07530.1	snap_masked-VaccDscaff46-processed-gene-135
GRAS	AT1G07530.1	maker-VaccDscaff19-snap-gene-352.29-mRNA-1
GRAS	AT1G07530.1	maker-VaccDscaff19-snap-gene-353.23-mRNA-1
GRAS	AT1G07530.1	maker-VaccDscaff19-snap-gene-353.27-mRNA-1
GRAS	AT1G07530.1	maker-VaccDscaff19-snap-gene-353.27-mRNA-1
GRAS	AT1G07530.1	maker-VaccDscaff19-snap-gene-353.27-mRNA-1
GRAS	AT1G07530.1	maker-VaccDscaff19-snap-gene-353.25-mRNA-1
GRAS	AT1G07530.1	maker-VaccDscaff19-snap-gene-353.25-mRNA-1
GRAS	AT1G07530.1	augustus_masked-VaccDscaff15-processed-gene
GRAS	AT1G07530.1	maker-VaccDscaff15-snap-gene-325.50-mRNA-1
GRAS	AT1G07530.1	maker-VaccDscaff15-snap-gene-325.50-mRNA-1
GRAS	AT1G07530.1	maker-VaccDscaff15-snap-gene-325.50-mRNA-1
GRAS	AT1G07530.1	snap_masked-VaccDscaff15-processed-gene-325
GRAS	AT1G07530.1	maker-VaccDscaff15-snap-gene-325.54-mRNA-1
GRAS	AT1G07530.1	maker-VaccDscaff15-snap-gene-325.54-mRNA-1
GRAS	AT1G07530.1	maker-VaccDscaff24-snap-gene-80.42-mRNA-1
GRAS	AT1G07530.1	maker-VaccDscaff24-snap-gene-80.39-mRNA-1
GRAS	AT1G07530.1	maker-VaccDscaff24-snap-gene-80.39-mRNA-1
GRAS	AT1G07530.1	maker-VaccDscaff24-snap-gene-80.39-mRNA-1
GRAS	AT1G07530.1	augustus_masked-VaccDscaff24-processed-gene
GRAS	AT1G07530.1	augustus_masked-VaccDscaff29-processed-gene
GRAS	AT1G07530.1	maker-VaccDscaff47-snap-gene-98.35-mRNA-1
MYB_related	AT1G07540.1	maker-VaccDscaff11-augustus-gene-334.31-mRN
MYB_related	AT1G07540.1	augustus_masked-VaccDscaff19-processed-gene
MYB_related	AT1G07540.1	augustus_masked-VaccDscaff19-processed-gene
MYB_related	AT1G07540.1	augustus_masked-VaccDscaff15-processed-gene
MYB_related	AT1G07540.1	augustus_masked-VaccDscaff15-processed-gene
MYB_related	AT1G07540.1	augustus_masked-VaccDscaff24-processed-gene
LBD	AT1G07900.1	maker-VaccDscaff22-augustus-gene-266.27-mRN
LBD	AT1G07900.1	maker-VaccDscaff43-augustus-gene-120.26-mRN
LBD	AT1G07900.1	maker-VaccDscaff11-augustus-gene-307.38-mRN
LBD	AT1G07900.1	snap_masked-VaccDscaff46-processed-gene-99.

LBD	AT1G07900.1	maker-VaccDscaff19-snap-gene-337.60-mRNA-1
LBD	AT1G07900.1	maker-VaccDscaff15-augustus-gene-291.38-mRN
LBD	AT1G07900.1	maker-VaccDscaff24-augustus-gene-95.27-mRNA
LBD	AT1G07900.1	snap_masked-VaccDscaff47-processed-gene-81.
NF-YC	AT1G07980.1	maker-VaccDscaff22-snap-gene-263.45-mRNA-1
NF-YC	AT1G07980.1	maker-VaccDscaff46-augustus-gene-96.25-mRNA
GATA	AT1G08000.2	maker-VaccDscaff22-augustus-gene-261.25-mRN
GATA	AT1G08000.2	maker-VaccDscaff22-augustus-gene-261.25-mRN
GATA	AT1G08000.2	maker-VaccDscaff46-augustus-gene-93.25-mRNA
GATA	AT1G08000.2	maker-VaccDscaff46-augustus-gene-93.25-mRNA
C2H2	AT1G08290.1	maker-VaccDscaff22-augustus-gene-240.23-mRN
C2H2	AT1G08290.1	maker-VaccDscaff46-augustus-gene-104.25-mRN
C2H2	AT1G08290.1	maker-VaccDscaff47-augustus-gene-53.19-mRNA
YABBY	AT1G08465.1	maker-VaccDscaff22-snap-gene-229.17-mRNA-1
YABBY	AT1G08465.1	maker-VaccDscaff43-augustus-gene-148.12-mRN
YABBY	AT1G08465.1	maker-VaccDscaff46-augustus-gene-117.21-mRN
YABBY	AT1G08465.1	maker-VaccDscaff47-augustus-gene-39.21-mRNA
MYB	AT1G08810.1	maker-VaccDscaff21-augustus-gene-309.26-mRN
MYB	AT1G08810.1	maker-VaccDscaff26-augustus-gene-302.35-mRN
MYB	AT1G08810.1	augustus_masked-VaccDscaff44-processed-gene
MYB	AT1G08810.1	maker-VaccDscaff29-augustus-gene-290.30-mRN
MYB	AT1G08810.1	maker-VaccDscaff3-snap-gene-141.27-mRNA-1
NF-YC	AT1G08970.4	maker-VaccDscaff27-snap-gene-149.23-mRNA-1
NF-YC	AT1G08970.4	maker-VaccDscaff45-snap-gene-169.15-mRNA-1
NF-YC	AT1G08970.4	maker-VaccDscaff17-snap-gene-186.26-mRNA-1
NF-YC	AT1G08970.4	maker-VaccDscaff9-snap-gene-24.45-mRNA-1
NF-YC	AT1G08970.4	maker-VaccDscaff35-snap-gene-44.41-mRNA-1
NF-YC	AT1G08970.4	maker-VaccDscaff34-snap-gene-162.22-mRNA-1
NF-YC	AT1G08970.4	snap_masked-VaccDscaff4-processed-gene-47.3
NF-YC	AT1G08970.4	snap_masked-VaccDscaff4-processed-gene-101.
NF-YB	AT1G09030.1	augustus_masked-VaccDscaff7-processed-gene-
NF-YB	AT1G09030.1	augustus_masked-VaccDscaff31-processed-gene
NF-YB	AT1G09030.1	augustus_masked-VaccDscaff18-processed-gene
MYB	AT1G09770.1	snap_masked-VaccDscaff27-processed-gene-65.
MYB	AT1G09770.1	snap_masked-VaccDscaff27-processed-gene-65.
MYB	AT1G09770.1	augustus_masked-VaccDscaff25-processed-gene
MYB	AT1G09770.1	maker-VaccDscaff45-snap-gene-14.41-mRNA-1
MYB	AT1G09770.1	maker-VaccDscaff17-snap-gene-103.35-mRNA-1
MYB	AT1G09770.1	snap_masked-VaccDscaff14-processed-gene-25.
MYB	AT1G09770.1	maker-VaccDscaff34-snap-gene-293.23-mRNA-1
MYB	AT1G09770.1	snap_masked-VaccDscaff3-processed-gene-404.
NAC	AT1G12260.1	maker-VaccDscaff22-augustus-gene-141.31-mRN
NAC	AT1G12260.1	maker-VaccDscaff43-augustus-gene-226.23-mRN
NAC	AT1G12260.1	augustus_masked-VaccDscaff21-processed-gene
NAC	AT1G12260.1	maker-VaccDscaff33-augustus-gene-255.32-mRN
NAC	AT1G12260.1	maker-VaccDscaff26-augustus-gene-81.34-mRNA

NAC	AT1G12260.1	maker-VaccDscaff46-augustus-gene-20.26-mRN
NAC	AT1G12260.1	augustus_masked-VaccDscaff29-processed-gene
RAV	AT1G13260.1	maker-VaccDscaff20-augustus-gene-121.18-mRN
RAV	AT1G13260.1	augustus_masked-VaccDscaff12-processed-gene
RAV	AT1G13260.1	augustus_masked-VaccDscaff25-processed-gene
RAV	AT1G13260.1	augustus_masked-VaccDscaff25-processed-gene
RAV	AT1G13260.1	augustus_masked-VaccDscaff2-processed-gene-
RAV	AT1G13260.1	augustus_masked-VaccDscaff40-processed-gene
RAV	AT1G13260.1	augustus_masked-VaccDscaff41-processed-gene
RAV	AT1G13260.1	augustus_masked-VaccDscaff14-processed-gene
RAV	AT1G13260.1	augustus_masked-VaccDscaff19-processed-gene
RAV	AT1G13260.1	augustus_masked-VaccDscaff30-processed-gene
RAV	AT1G13260.1	augustus_masked-VaccDscaff4-processed-gene-
RAV	AT1G13260.1	augustus_masked-VaccDscaff23-processed-gene
RAV	AT1G13260.1	augustus_masked-VaccDscaff3-processed-gene-
RAV	AT1G13260.1	augustus_masked-VaccDscaff28-processed-gene
RAV	AT1G13260.1	snap_masked-VaccDscaff28-processed-gene-23
C2H2	AT1G13290.1	maker-VaccDscaff20-augustus-gene-121.22-mRN
C2H2	AT1G13290.1	maker-VaccDscaff19-augustus-gene-124.26-mRN
C2H2	AT1G13290.1	maker-VaccDscaff28-augustus-gene-225.15-mRN
Trihelix	AT1G13450.3	maker-VaccDscaff30-augustus-gene-313.27-mRN
WRKY	AT1G13960.1	maker-VaccDscaff25-augustus-gene-288.22-mRN
WRKY	AT1G13960.1	maker-VaccDscaff2-augustus-gene-293.35-mRN
WRKY	AT1G13960.1	maker-VaccDscaff2-augustus-gene-293.35-mRN
WRKY	AT1G13960.1	maker-VaccDscaff14-augustus-gene-249.28-mRN
WRKY	AT1G13960.1	maker-VaccDscaff3-snap-gene-120.33-mRNA-1
WRKY	AT1G13960.1	maker-VaccDscaff3-snap-gene-120.33-mRNA-1
WRKY	AT1G13960.1	maker-VaccDscaff3-snap-gene-120.33-mRNA-1
MYB	AT1G14350.2	maker-VaccDscaff36-augustus-gene-127.21-mRN
MYB	AT1G14350.2	maker-VaccDscaff9-augustus-gene-292.18-mRN
MYB	AT1G14350.2	maker-VaccDscaff35-augustus-gene-223.35-mRN
MYB	AT1G14350.2	maker-VaccDscaff4-augustus-gene-317.25-mRN
Whirly	AT1G14410.1	maker-VaccDscaff9-augustus-gene-291.24-mRN
Whirly	AT1G14410.1	maker-VaccDscaff4-augustus-gene-316.27-mRN
G2-like	AT1G14600.1	maker-VaccDscaff20-snap-gene-168.21-mRNA-1
G2-like	AT1G14600.1	maker-VaccDscaff28-snap-gene-158.34-mRNA-1
GRAS	AT1G14920.1	augustus_masked-VaccDscaff680-processed-gen
GRAS	AT1G14920.1	augustus_masked-VaccDscaff13-processed-gene
GRAS	AT1G14920.1	augustus_masked-VaccDscaff41-processed-gene
GRAS	AT1G14920.1	snap_masked-VaccDscaff19-processed-gene-112
GRAS	AT1G14920.1	augustus_masked-VaccDscaff61-processed-gene
GRAS	AT1G14920.1	augustus_masked-VaccDscaff42-processed-gene
GRAS	AT1G14920.1	augustus_masked-VaccDscaff23-processed-gene
MYB_related	AT1G15720.1	maker-VaccDscaff28-snap-gene-201.45-mRNA-1
MYB_related	AT1G15720.1	maker-VaccDscaff28-snap-gene-201.45-mRNA-1
AP2	AT1G16060.1	maker-VaccDscaff17-augustus-gene-330.30-mRN

AP2	AT1G16060.1	maker-VaccDscaff17-augustus-gene-332.45-mRN
AP2	AT1G16060.1	maker-VaccDscaff27-snap-gene-290.40-mRNA-1
AP2	AT1G16060.1	maker-VaccDscaff34-augustus-gene-54.42-mRN/
AP2	AT1G16060.1	maker-VaccDscaff35-augustus-gene-291.30-mRN
AP2	AT1G16060.1	maker-VaccDscaff36-augustus-gene-35.33-mRN/
AP2	AT1G16060.1	maker-VaccDscaff4-augustus-gene-396.19-mRN/
AP2	AT1G16060.1	maker-VaccDscaff9-augustus-gene-375.42-mRN/
MYB	AT1G16490.1	maker-VaccDscaff58-snap-gene-1.25-mRNA-1
STAT	AT1G17040.1	maker-VaccDscaff1048-augustus-gene-0.2-mRN/
MYB	AT1G17950.1	maker-VaccDscaff25-augustus-gene-200.22-mRN
MYB	AT1G17950.1	maker-VaccDscaff2-augustus-gene-208.18-mRN/
MYB	AT1G17950.1	maker-VaccDscaff14-augustus-gene-162.19-mRN
MYB	AT1G17950.1	maker-VaccDscaff3-augustus-gene-234.13-mRN/
Nin-like	AT1G18790.1	maker-VaccDscaff13-augustus-gene-255.16-mRN
Nin-like	AT1G18790.1	maker-VaccDscaff32-augustus-gene-145.21-mRN
Nin-like	AT1G18790.1	maker-VaccDscaff30-augustus-gene-147.19-mRN
Nin-like	AT1G18790.1	maker-VaccDscaff42-snap-gene-167.31-mRNA-1
ZF-HD	AT1G18835.1	augustus_masked-VaccDscaff14-processed-gene
ZF-HD	AT1G18835.1	augustus_masked-VaccDscaff3-processed-gene-
MYB_related	AT1G19000.2	maker-VaccDscaff34-augustus-gene-118.19-mRN
ERF	AT1G19210.1	snap_masked-VaccDscaff71-processed-gene-3.1
ERF	AT1G19210.1	snap_masked-VaccDscaff159-processed-gene-2.
ARF	AT1G19220.1	maker-VaccDscaff10-snap-gene-330.33-mRNA-1
ARF	AT1G19220.1	maker-VaccDscaff12-augustus-gene-319.28-mRN
ARF	AT1G19220.1	maker-VaccDscaff12-snap-gene-319.35-mRNA-1
ARF	AT1G19220.1	augustus_masked-VaccDscaff12-processed-gene
ARF	AT1G19220.1	snap_masked-VaccDscaff13-processed-gene-39
ARF	AT1G19220.1	maker-VaccDscaff40-snap-gene-213.41-mRNA-1
ARF	AT1G19220.1	snap_masked-VaccDscaff40-processed-gene-21
ARF	AT1G19220.1	augustus_masked-VaccDscaff40-processed-gene
ARF	AT1G19220.1	snap_masked-VaccDscaff41-processed-gene-19
ARF	AT1G19220.1	snap_masked-VaccDscaff41-processed-gene-19
ARF	AT1G19220.1	snap_masked-VaccDscaff41-processed-gene-19
ARF	AT1G19220.1	maker-VaccDscaff30-snap-gene-3.34-mRNA-1
ARF	AT1G19220.1	snap_masked-VaccDscaff71-processed-gene-4.1
ARF	AT1G19220.1	maker-VaccDscaff23-augustus-gene-73.28-mRN/
ARF	AT1G19220.1	maker-VaccDscaff23-augustus-gene-73.28-mRN/
ARF	AT1G19220.1	snap_masked-VaccDscaff159-processed-gene-2.
ARF	AT1G19850.1	maker-VaccDscaff12-snap-gene-56.27-mRNA-1
ARF	AT1G19850.1	maker-VaccDscaff23-augustus-gene-349.16-mRN
ARF	AT1G19850.1	maker-VaccDscaff23-snap-gene-355.33-mRNA-1
SBP	AT1G20980.1	maker-VaccDscaff12-snap-gene-18.52-mRNA-1
SBP	AT1G20980.1	maker-VaccDscaff12-snap-gene-28.41-mRNA-1
SBP	AT1G20980.1	maker-VaccDscaff23-snap-gene-366.37-mRNA-1
GRAS	AT1G21450.1	augustus_masked-VaccDscaff2-processed-gene-
GRAS	AT1G21450.1	augustus_masked-VaccDscaff14-processed-gene

GRAS	AT1G21450.1	augustus_masked-VaccDscaff3-processed-gene-:
ERF	AT1G21910.1	snap_masked-VaccDscaff25-processed-gene-221
ERF	AT1G21910.1	augustus_masked-VaccDscaff2-processed-gene-:
ERF	AT1G21910.1	augustus_masked-VaccDscaff14-processed-gene
ERF	AT1G21910.1	augustus_masked-VaccDscaff3-processed-gene-:
MIKC_MADS	AT1G22130.1	maker-VaccDscaff18-augustus-gene-37.32-mRN/
MIKC_MADS	AT1G22130.1	augustus_masked-VaccDscaff34-processed-gene
MYB	AT1G22640.1	maker-VaccDscaff20-snap-gene-232.35-mRNA-1
MYB	AT1G22640.1	maker-VaccDscaff32-augustus-gene-55.29-mRN/
ERF	AT1G22810.1	augustus_masked-VaccDscaff200-processed-gen
ERF	AT1G22810.1	maker-VaccDscaff200-augustus-gene-0.35-mRN/
ERF	AT1G22810.1	augustus_masked-VaccDscaff3-processed-gene-:
ERF	AT1G22810.1	augustus_masked-VaccDscaff3-processed-gene-:
TALE	AT1G23380.1	maker-VaccDscaff27-snap-gene-130.21-mRNA-1
TALE	AT1G23380.1	maker-VaccDscaff27-augustus-gene-255.29-mRN
TALE	AT1G23380.1	maker-VaccDscaff36-augustus-gene-157.10-mRN
TALE	AT1G23380.1	maker-VaccDscaff45-snap-gene-132.24-mRNA-1
TALE	AT1G23380.1	maker-VaccDscaff17-snap-gene-167.20-mRNA-1
TALE	AT1G23380.1	maker-VaccDscaff17-augustus-gene-282.15-mRN
TALE	AT1G23380.1	maker-VaccDscaff17-augustus-gene-282.15-mRN
TALE	AT1G23380.1	maker-VaccDscaff9-snap-gene-265.26-mRNA-1
TALE	AT1G23380.1	maker-VaccDscaff35-snap-gene-200.18-mRNA-1
TALE	AT1G23380.1	maker-VaccDscaff34-augustus-gene-97.28-mRN/
TALE	AT1G23380.1	maker-VaccDscaff4-snap-gene-296.28-mRNA-1
TALE	AT1G23380.2	maker-VaccDscaff36-snap-gene-157.15-mRNA-1
TALE	AT1G23380.2	maker-VaccDscaff9-augustus-gene-265.21-mRN/
TALE	AT1G23380.2	maker-VaccDscaff35-snap-gene-200.17-mRNA-1
TALE	AT1G23380.2	maker-VaccDscaff4-augustus-gene-296.24-mRN/
YABBY	AT1G23420.1	maker-VaccDscaff36-augustus-gene-155.28-mRN
YABBY	AT1G23420.1	maker-VaccDscaff4-augustus-gene-297.22-mRN/
MIKC_MADS	AT1G24260.1	maker-VaccDscaff102-augustus-gene-1.33-mRN/
MIKC_MADS	AT1G24260.1	maker-VaccDscaff2-augustus-gene-240.26-mRN/
MIKC_MADS	AT1G24260.1	maker-VaccDscaff14-augustus-gene-201.25-mRN
MIKC_MADS	AT1G24260.1	maker-VaccDscaff9-augustus-gene-224.37-mRN/
MIKC_MADS	AT1G24260.1	maker-VaccDscaff3-augustus-gene-198.30-mRN/
MIKC_MADS	AT1G24260.3	augustus_masked-VaccDscaff3-processed-gene-:
bHLH	AT1G25330.1	maker-VaccDscaff28-augustus-gene-214.18-mRN
MYB	AT1G25340.1	maker-VaccDscaff42-augustus-gene-127.23-mRN
G2-like	AT1G25550.1	maker-VaccDscaff20-augustus-gene-121.21-mRN
G2-like	AT1G25550.1	maker-VaccDscaff19-augustus-gene-124.22-mRN
G2-like	AT1G25550.1	maker-VaccDscaff28-augustus-gene-225.19-mRN
NAC	AT1G25580.1	maker-VaccDscaff20-augustus-gene-120.26-mRN
NAC	AT1G25580.1	maker-VaccDscaff25-augustus-gene-294.20-mRN
NAC	AT1G25580.1	maker-VaccDscaff2-augustus-gene-298.19-mRN/
NAC	AT1G25580.1	maker-VaccDscaff14-snap-gene-253.20-mRNA-1
NAC	AT1G25580.1	maker-VaccDscaff19-augustus-gene-123.23-mRN

NAC	AT1G25580.1	augustus_masked-VaccDscaff3-processed-gene-[illegible]
NAC	AT1G25580.1	maker-VaccDscaff28-augustus-gene-226.21-mRN[illegible]
NAC	AT1G25580.1	maker-VaccDscaff28-augustus-gene-233.12-mRN[illegible]
C2H2	AT1G26610.1	augustus_masked-VaccDscaff13-processed-gene
C2H2	AT1G26610.1	augustus_masked-VaccDscaff13-processed-gene
C2H2	AT1G26610.1	augustus_masked-VaccDscaff32-processed-gene
C2H2	AT1G26610.1	augustus_masked-VaccDscaff32-processed-gene
C2H2	AT1G26610.1	snap_masked-VaccDscaff30-processed-gene-32[illegible]
C2H2	AT1G26610.1	snap_masked-VaccDscaff30-processed-gene-32[illegible]
NAC	AT1G26870.1	maker-VaccDscaff36-snap-gene-126.37-mRNA-1
NAC	AT1G26870.1	maker-VaccDscaff13-augustus-gene-78.34-mRN[illegible]
NAC	AT1G26870.1	maker-VaccDscaff7-augustus-gene-364.34-mRN[illegible]
NAC	AT1G26870.1	maker-VaccDscaff9-augustus-gene-293.20-mRN[illegible]
NAC	AT1G26870.1	maker-VaccDscaff32-snap-gene-286.42-mRNA-1
NAC	AT1G26870.1	maker-VaccDscaff35-augustus-gene-224.15-mRN[illegible]
NAC	AT1G26870.1	maker-VaccDscaff30-snap-gene-327.41-mRNA-1
NAC	AT1G26870.1	maker-VaccDscaff4-snap-gene-318.24-mRNA-1
bHLH	AT1G26945.1	maker-VaccDscaff2-augustus-gene-274.21-mRN[illegible]
bHLH	AT1G26945.1	maker-VaccDscaff2-augustus-gene-275.29-mRN[illegible]
bHLH	AT1G26945.1	maker-VaccDscaff94-augustus-gene-5.35-mRNA-[illegible]
bHLH	AT1G26945.1	maker-VaccDscaff3-augustus-gene-163.12-mRN[illegible]
C2H2	AT1G27730.1	augustus_masked-VaccDscaff12-processed-gene
C2H2	AT1G27730.1	augustus_masked-VaccDscaff16-processed-gene
C2H2	AT1G27730.1	augustus_masked-VaccDscaff7-processed-gene-[illegible]
C2H2	AT1G27730.1	augustus_masked-VaccDscaff31-processed-gene
C2H2	AT1G27730.1	augustus_masked-VaccDscaff2-processed-gene-[illegible]
C2H2	AT1G27730.1	augustus_masked-VaccDscaff18-processed-gene
C2H2	AT1G27730.1	augustus_masked-VaccDscaff40-processed-gene
C2H2	AT1G27730.1	augustus_masked-VaccDscaff14-processed-gene
C2H2	AT1G27730.1	augustus_masked-VaccDscaff257-processed-gen[illegible]
C2H2	AT1G27730.1	augustus_masked-VaccDscaff23-processed-gene
C2H2	AT1G27730.1	augustus_masked-VaccDscaff3-processed-gene-[illegible]
CO-like	AT1G28050.1	snap_masked-VaccDscaff37-processed-gene-241
ERF	AT1G28360.1	augustus_masked-VaccDscaff9-processed-gene-[illegible]
ERF	AT1G28360.1	augustus_masked-VaccDscaff35-processed-gene
Dof	AT1G29160.1	augustus_masked-VaccDscaff9-processed-gene-[illegible]
Dof	AT1G29160.1	augustus_masked-VaccDscaff35-processed-gene
Dof	AT1G29160.1	augustus_masked-VaccDscaff4-processed-gene-[illegible]
WRKY	AT1G29280.1	maker-VaccDscaff11-snap-gene-132.20-mRNA-1
WRKY	AT1G29280.1	maker-VaccDscaff11-snap-gene-132.20-mRNA-1
WRKY	AT1G29280.1	maker-VaccDscaff19-snap-gene-214.17-mRNA-1
WRKY	AT1G29280.1	maker-VaccDscaff19-snap-gene-214.17-mRNA-1
WRKY	AT1G29280.1	maker-VaccDscaff15-augustus-gene-123.18-mRN[illegible]
WRKY	AT1G29280.1	maker-VaccDscaff15-augustus-gene-385.29-mRN[illegible]
WRKY	AT1G29280.1	maker-VaccDscaff9-augustus-gene-187.26-mRN[illegible]
WRKY	AT1G29280.1	maker-VaccDscaff35-augustus-gene-135.31-mRN[illegible]

WRKY	AT1G29280.1	maker-VaccDscaff24-snap-gene-263.22-mRNA-1
WRKY	AT1G29280.1	maker-VaccDscaff24-snap-gene-263.22-mRNA-1
WRKY	AT1G29280.1	maker-VaccDscaff4-augustus-gene-209.28-mRNA
WRKY	AT1G29280.1	maker-VaccDscaff51-snap-gene-5.52-mRNA-1
WRKY	AT1G29860.1	maker-VaccDscaff330-augustus-gene-0.25-mRNA
WRKY	AT1G29860.1	augustus_masked-VaccDscaff2-processed-gene-
WRKY	AT1G29860.1	augustus_masked-VaccDscaff14-processed-gene
WRKY	AT1G29860.1	maker-VaccDscaff3-augustus-gene-215.25-mRNA
HD-ZIP	AT1G30490.1	maker-VaccDscaff20-augustus-gene-213.21-mRN
HD-ZIP	AT1G30490.1	maker-VaccDscaff44-augustus-gene-142.17-mRN
NF-YA	AT1G30500.2	maker-VaccDscaff20-snap-gene-214.21-mRNA-1
NF-YA	AT1G30500.2	maker-VaccDscaff11-snap-gene-259.55-mRNA-1
NF-YA	AT1G30500.2	maker-VaccDscaff19-snap-gene-295.45-mRNA-1
NF-YA	AT1G30500.2	maker-VaccDscaff15-augustus-gene-249.30-mRN
NF-YA	AT1G30500.2	maker-VaccDscaff44-snap-gene-141.20-mRNA-1
NF-YA	AT1G30500.2	maker-VaccDscaff24-snap-gene-136.64-mRNA-1
bHLH	AT1G30670.1	maker-VaccDscaff38-augustus-gene-27.38-mRNA
bHLH	AT1G30670.1	maker-VaccDscaff12-augustus-gene-110.26-mRN
bHLH	AT1G30670.1	maker-VaccDscaff6-augustus-gene-388.28-mRNA
bHLH	AT1G30670.1	maker-VaccDscaff37-augustus-gene-269.34-mRN
bHLH	AT1G30670.1	maker-VaccDscaff23-augustus-gene-302.29-mRN
bHLH	AT1G30670.1	maker-VaccDscaff39-augustus-gene-26.24-mRNA
C2H2	AT1G30970.1	maker-VaccDscaff11-augustus-gene-224.43-mRN
C2H2	AT1G30970.1	maker-VaccDscaff15-snap-gene-213.36-mRNA-1
C2H2	AT1G30970.1	maker-VaccDscaff15-augustus-gene-220.44-mRN
C2H2	AT1G30970.1	maker-VaccDscaff24-augustus-gene-167.35-mRN
LBD	AT1G31320.1	maker-VaccDscaff701-augustus-gene-0.11-mRNA
LBD	AT1G31320.1	maker-VaccDscaff62-augustus-gene-5.25-mRNA-
LBD	AT1G31320.1	maker-VaccDscaff298-augustus-gene-0.34-mRNA
LBD	AT1G31320.1	maker-VaccDscaff11-augustus-gene-345.33-mRN
LBD	AT1G31320.1	maker-VaccDscaff58-augustus-gene-2.27-mRNA-
LBD	AT1G31320.1	maker-VaccDscaff6-augustus-gene-7.37-mRNA-1
LBD	AT1G31320.1	maker-VaccDscaff166-augustus-gene-2.45-mRNA
LBD	AT1G31320.1	maker-VaccDscaff24-snap-gene-56.35-mRNA-1
LBD	AT1G31320.1	maker-VaccDscaff24-snap-gene-56.35-mRNA-1
LBD	AT1G31320.1	maker-VaccDscaff78-augustus-gene-4.25-mRNA-
G2-like	AT1G32240.1	maker-VaccDscaff22-augustus-gene-182.17-mRN
G2-like	AT1G32240.1	maker-VaccDscaff43-augustus-gene-182.20-mRN
G2-like	AT1G32240.1	maker-VaccDscaff25-augustus-gene-40.21-mRNA
G2-like	AT1G32240.1	maker-VaccDscaff2-augustus-gene-57.23-mRNA-
G2-like	AT1G32240.1	maker-VaccDscaff46-augustus-gene-63.19-mRNA
G2-like	AT1G32240.1	maker-VaccDscaff14-augustus-gene-42.21-mRNA
G2-like	AT1G32240.1	maker-VaccDscaff3-augustus-gene-385.18-mRNA
HSF	AT1G32330.1	maker-VaccDscaff14-augustus-gene-44.30-mRNA
C3H	AT1G32360.1	maker-VaccDscaff22-augustus-gene-178.23-mRN
C3H	AT1G32360.1	maker-VaccDscaff43-augustus-gene-185.31-mRN

C3H	AT1G32360.1	maker-VaccDscaff46-augustus-gene-59.30-mRN/
LSD	AT1G32540.1	maker-VaccDscaff2-snap-gene-13.28-mRNA-1
LSD	AT1G32540.3	maker-VaccDscaff22-augustus-gene-171.36-mRN
LSD	AT1G32540.3	maker-VaccDscaff25-snap-gene-50.41-mRNA-1
bHLH	AT1G32640.1	augustus_masked-VaccDscaff22-processed-gene
bHLH	AT1G32640.1	augustus_masked-VaccDscaff38-processed-gene
bHLH	AT1G32640.1	snap_masked-VaccDscaff43-processed-gene-196
bHLH	AT1G32640.1	maker-VaccDscaff27-augustus-gene-121.34-mRN
bHLH	AT1G32640.1	augustus_masked-VaccDscaff20-processed-gene
bHLH	AT1G32640.1	augustus_masked-VaccDscaff12-processed-gene
bHLH	AT1G32640.1	augustus_masked-VaccDscaff25-processed-gene
bHLH	AT1G32640.1	augustus_masked-VaccDscaff16-processed-gene
bHLH	AT1G32640.1	snap_masked-VaccDscaff16-processed-gene-301
bHLH	AT1G32640.1	augustus_masked-VaccDscaff46-processed-gene
bHLH	AT1G32640.1	augustus_masked-VaccDscaff30-processed-gene
SRS	AT1G32730.1	maker-VaccDscaff22-augustus-gene-167.24-mRN
SRS	AT1G32730.1	maker-VaccDscaff43-snap-gene-203.26-mRNA-1
SRS	AT1G32730.1	maker-VaccDscaff46-snap-gene-47.38-mRNA-1
NAC	AT1G32770.1	maker-VaccDscaff21-snap-gene-165.44-mRNA-1
NAC	AT1G32770.1	maker-VaccDscaff26-snap-gene-167.34-mRNA-1
C2H2	AT1G34370.2	maker-VaccDscaff38-augustus-gene-176.32-mRN
C2H2	AT1G34370.2	maker-VaccDscaff6-augustus-gene-193.34-mRN/
C2H2	AT1G34370.2	maker-VaccDscaff37-augustus-gene-104.16-mRN
C2H2	AT1G34370.3	maker-VaccDscaff13-augustus-gene-13.36-mRN/
C2H2	AT1G34370.3	maker-VaccDscaff30-augustus-gene-269.43-mRN
C2H2	AT1G34370.3	maker-VaccDscaff42-augustus-gene-47.29-mRN/
MYB	AT1G34670.1	maker-VaccDscaff38-augustus-gene-80.26-mRN/
MYB	AT1G34670.1	maker-VaccDscaff12-augustus-gene-233.16-mRN
MYB	AT1G34670.1	maker-VaccDscaff6-snap-gene-337.23-mRNA-1
MYB	AT1G34670.1	maker-VaccDscaff40-augustus-gene-83.21-mRN/
MYB	AT1G34670.1	maker-VaccDscaff41-augustus-gene-73.16-mRN/
MYB	AT1G34670.1	maker-VaccDscaff37-augustus-gene-230.19-mRN
MYB	AT1G34670.1	maker-VaccDscaff23-augustus-gene-244.11-mRN
MYB	AT1G34670.1	maker-VaccDscaff39-augustus-gene-86.16-mRN/
C2H2	AT1G34790.1	maker-VaccDscaff12-snap-gene-235.31-mRNA-1
C2H2	AT1G34790.1	augustus_masked-VaccDscaff40-processed-gene
C2H2	AT1G34790.1	augustus_masked-VaccDscaff40-processed-gene
C2H2	AT1G34790.1	maker-VaccDscaff41-augustus-gene-70.24-mRN/
C2H2	AT1G34790.1	maker-VaccDscaff23-augustus-gene-247.21-mRN
C2H2	AT1G34790.1	maker-VaccDscaff23-augustus-gene-247.21-mRN
bHLH	AT1G35460.1	maker-VaccDscaff2-snap-gene-220.32-mRNA-1
bHLH	AT1G35460.1	maker-VaccDscaff14-snap-gene-179.40-mRNA-1
bHLH	AT1G35460.1	maker-VaccDscaff3-snap-gene-216.36-mRNA-1
bHLH	AT1G35460.1	maker-VaccDscaff3-snap-gene-217.37-mRNA-1
ERF	AT1G36060.1	snap_masked-VaccDscaff36-processed-gene-256
ERF	AT1G36060.1	maker-VaccDscaff35-augustus-gene-20.23-mRN/

bZIP	AT1G45249.1	maker-VaccDscaff12-augustus-gene-94.20-mRNA
bZIP	AT1G45249.1	maker-VaccDscaff7-snap-gene-24.27-mRNA-1
bZIP	AT1G45249.1	augustus_masked-VaccDscaff31-processed-gene
bZIP	AT1G45249.1	maker-VaccDscaff18-augustus-gene-2.21-mRNA-
bZIP	AT1G45249.1	maker-VaccDscaff40-augustus-gene-24.16-mRNA
bZIP	AT1G45249.1	maker-VaccDscaff41-augustus-gene-47.25-mRNA
bZIP	AT1G45249.1	maker-VaccDscaff982-snap-gene-0.6-mRNA-1
bZIP	AT1G45249.1	maker-VaccDscaff23-snap-gene-318.41-mRNA-1
bZIP	AT1G45249.1	maker-VaccDscaff169-augustus-gene-0.20-mRNA
HSF	AT1G46264.1	maker-VaccDscaff12-augustus-gene-96.24-mRNA
HSF	AT1G46264.1	maker-VaccDscaff7-augustus-gene-23.34-mRNA-
HSF	AT1G46264.1	maker-VaccDscaff31-snap-gene-337.34-mRNA-1
HSF	AT1G46264.1	maker-VaccDscaff18-snap-gene-3.42-mRNA-1
HSF	AT1G46264.1	maker-VaccDscaff40-augustus-gene-26.25-mRNA
HSF	AT1G46264.1	maker-VaccDscaff41-augustus-gene-45.26-mRNA
HSF	AT1G46264.1	maker-VaccDscaff23-augustus-gene-315.33-mRN
HSF	AT1G46264.1	maker-VaccDscaff169-augustus-gene-0.18-mRNA
WOX	AT1G46480.1	maker-VaccDscaff12-augustus-gene-98.14-mRNA
WOX	AT1G46480.1	maker-VaccDscaff7-augustus-gene-22.26-mRNA-
WOX	AT1G46480.1	maker-VaccDscaff31-augustus-gene-335.17-mRN
WOX	AT1G46480.1	maker-VaccDscaff18-snap-gene-5.52-mRNA-1
WOX	AT1G46480.1	maker-VaccDscaff40-augustus-gene-28.21-mRNA
WOX	AT1G46480.1	maker-VaccDscaff41-augustus-gene-43.22-mRNA
WOX	AT1G46480.1	maker-VaccDscaff222-augustus-gene-0.19-mRNA
WOX	AT1G46480.1	maker-VaccDscaff23-augustus-gene-313.32-mRN
E2F/DP	AT1G47870.1	snap_masked-VaccDscaff12-processed-gene-25.
E2F/DP	AT1G47870.1	snap_masked-VaccDscaff12-processed-gene-54.
E2F/DP	AT1G47870.2	augustus_masked-VaccDscaff23-processed-gene
MYB	AT1G48000.1	maker-VaccDscaff11-augustus-gene-396.29-mRN
MYB	AT1G48000.1	maker-VaccDscaff15-augustus-gene-387.25-mRN
MYB	AT1G48000.1	maker-VaccDscaff24-augustus-gene-2.25-mRNA-
MYB	AT1G48000.1	maker-VaccDscaff51-augustus-gene-3.19-mRNA-
MYB	AT1G49010.1	maker-VaccDscaff13-augustus-gene-208.43-mRN
MYB	AT1G49010.1	maker-VaccDscaff279-augustus-gene-0.38-mRNA
MYB	AT1G49010.1	maker-VaccDscaff32-augustus-gene-172.23-mRN
MYB	AT1G49010.1	maker-VaccDscaff42-augustus-gene-135.18-mRN
MYB_related	AT1G49950.3	maker-VaccDscaff2-snap-gene-401.25-mRNA-1
MYB_related	AT1G49950.3	maker-VaccDscaff14-snap-gene-347.33-mRNA-1
GRAS	AT1G50420.1	maker-VaccDscaff11-augustus-gene-60.23-mRNA
GRAS	AT1G50420.1	maker-VaccDscaff19-augustus-gene-161.20-mRN
GRAS	AT1G50420.1	maker-VaccDscaff15-augustus-gene-67.32-mRNA
GRAS	AT1G50420.1	maker-VaccDscaff24-snap-gene-335.41-mRNA-1
GRAS	AT1G50420.1	maker-VaccDscaff760-snap-gene-0.17-mRNA-1
ERF	AT1G50640.1	augustus_masked-VaccDscaff10-processed-gene
ERF	AT1G50640.1	augustus_masked-VaccDscaff8-processed-gene-[illegible]
ERF	AT1G50640.1	augustus_masked-VaccDscaff22-processed-gene

ERF	AT1G50640.1	augustus_masked-VaccDscaff43-processed-gene
ERF	AT1G50640.1	augustus_masked-VaccDscaff5-processed-gene-
ERF	AT1G50640.1	augustus_masked-VaccDscaff1-processed-gene-
ERF	AT1G50640.1	augustus_masked-VaccDscaff47-processed-gene
RAV	AT1G51120.1	augustus_masked-VaccDscaff27-processed-gene
RAV	AT1G51120.1	augustus_masked-VaccDscaff12-processed-gene
RAV	AT1G51120.1	augustus_masked-VaccDscaff45-processed-gene
RAV	AT1G51120.1	augustus_masked-VaccDscaff17-processed-gene
RAV	AT1G51120.1	augustus_masked-VaccDscaff40-processed-gene
RAV	AT1G51120.1	augustus_masked-VaccDscaff41-processed-gene
RAV	AT1G51120.1	augustus_masked-VaccDscaff34-processed-gene
RAV	AT1G51120.1	augustus_masked-VaccDscaff23-processed-gene
AP2	AT1G51190.1	augustus_masked-VaccDscaff44-processed-gene
AP2	AT1G51190.1	maker-VaccDscaff11-snap-gene-36.17-mRNA-1
AP2	AT1G51190.1	maker-VaccDscaff15-snap-gene-44.26-mRNA-1
GATA	AT1G51600.2	maker-VaccDscaff18-augustus-gene-54.31-mRN
GATA	AT1G51600.2	maker-VaccDscaff18-augustus-gene-54.31-mRN
Dof	AT1G51700.1	maker-VaccDscaff27-snap-gene-20.34-mRNA-1
Dof	AT1G51700.1	augustus_masked-VaccDscaff27-processed-gene
Dof	AT1G51700.1	augustus_masked-VaccDscaff17-processed-gene
Dof	AT1G51700.1	augustus_masked-VaccDscaff496-processed-gen
HD-ZIP	AT1G52150.1	maker-VaccDscaff27-snap-gene-320.33-mRNA-1
HD-ZIP	AT1G52150.1	maker-VaccDscaff27-snap-gene-320.33-mRNA-1
HD-ZIP	AT1G52150.1	maker-VaccDscaff17-snap-gene-356.34-mRNA-1
HD-ZIP	AT1G52150.1	maker-VaccDscaff17-snap-gene-356.34-mRNA-1
HD-ZIP	AT1G52150.2	snap_masked-VaccDscaff34-processed-gene-26.
HD-ZIP	AT1G52150.2	snap_masked-VaccDscaff34-processed-gene-26.
HD-ZIP	AT1G52150.2	snap_masked-VaccDscaff34-processed-gene-26.
HD-ZIP	AT1G52150.3	maker-VaccDscaff34-augustus-gene-26.36-mRN
NAC	AT1G52890.1	snap_masked-VaccDscaff17-processed-gene-25
NAC	AT1G52890.1	snap_masked-VaccDscaff19-processed-gene-15
ERF	AT1G53910.3	maker-VaccDscaff27-snap-gene-332.36-mRNA-1
NF-YC	AT1G54830.3	augustus_masked-VaccDscaff20-processed-gene
NF-YC	AT1G54830.3	augustus_masked-VaccDscaff44-processed-gene
NF-YC	AT1G54830.3	augustus_masked-VaccDscaff44-processed-gene
GRAS	AT1G55580.1	augustus_masked-VaccDscaff6-processed-gene-
GRAS	AT1G55580.1	augustus_masked-VaccDscaff6-processed-gene-
GRAS	AT1G55580.1	augustus_masked-VaccDscaff90-processed-gene
NAC	AT1G56010.1	maker-VaccDscaff205-snap-gene-0.29-mRNA-1
NAC	AT1G56010.2	maker-VaccDscaff62-augustus-gene-7.28-mRNA-
NAC	AT1G56010.2	maker-VaccDscaff6-augustus-gene-10.17-mRNA-
NAC	AT1G56010.2	maker-VaccDscaff6-augustus-gene-30.24-mRNA-
NAC	AT1G56010.2	maker-VaccDscaff90-augustus-gene-1.17-mRNA-
NAC	AT1G56010.2	maker-VaccDscaff1-snap-gene-461.86-mRNA-1
NAC	AT1G56010.2	snap_masked-VaccDscaff78-processed-gene-6.2
NF-YC	AT1G56170.2	maker-VaccDscaff381-augustus-gene-0.26-mRN

NF-YC	AT1G56170.2	snap_masked-VaccDscaff9-processed-gene-356.
NF-YC	AT1G56170.2	snap_masked-VaccDscaff9-processed-gene-356.
NF-YC	AT1G56170.2	snap_masked-VaccDscaff35-processed-gene-273
NF-YC	AT1G56170.2	maker-VaccDscaff268-augustus-gene-0.56-mRNA
NF-YC	AT1G56170.2	augustus_masked-VaccDscaff4-processed-gene-
NF-YC	AT1G56170.2	snap_masked-VaccDscaff4-processed-gene-376.
TCP	AT1G58100.1	snap_masked-VaccDscaff11-processed-gene-113
TCP	AT1G58100.1	snap_masked-VaccDscaff11-processed-gene-236
TCP	AT1G58100.1	snap_masked-VaccDscaff106-processed-gene-0.
bZIP	AT1G58110.2	maker-VaccDscaff27-snap-gene-92.37-mRNA-1
bZIP	AT1G58110.2	maker-VaccDscaff31-augustus-gene-239.23-mRN
bZIP	AT1G58110.2	maker-VaccDscaff34-snap-gene-222.30-mRNA-1
bHLH	AT1G59640.1	maker-VaccDscaff36-augustus-gene-131.23-mRN
bHLH	AT1G59640.1	maker-VaccDscaff9-augustus-gene-287.31-mRNA
bHLH	AT1G59640.1	maker-VaccDscaff35-augustus-gene-219.31-mRN
bHLH	AT1G59640.1	maker-VaccDscaff4-augustus-gene-312.22-mRNA
ARF	AT1G59750.2	maker-VaccDscaff10-snap-gene-296.46-mRNA-1
ARF	AT1G59750.3	maker-VaccDscaff2-augustus-gene-355.18-mRNA
ARF	AT1G59750.4	augustus_masked-VaccDscaff8-processed-gene-
ARF	AT1G59750.4	augustus_masked-VaccDscaff25-processed-gene
ARF	AT1G59750.4	maker-VaccDscaff5-snap-gene-80.53-mRNA-1
ARF	AT1G59750.4	augustus_masked-VaccDscaff14-processed-gene
ARF	AT1G59750.4	augustus_masked-VaccDscaff3-processed-gene-
NAC	AT1G61110.1	snap_masked-VaccDscaff10-processed-gene-138
NAC	AT1G61110.1	maker-VaccDscaff17-snap-gene-86.28-mRNA-1
NAC	AT1G61110.1	maker-VaccDscaff1-snap-gene-291.34-mRNA-1
NAC	AT1G61110.1	maker-VaccDscaff34-augustus-gene-304.20-mRN
WRKY	AT1G62300.1	maker-VaccDscaff11-augustus-gene-207.26-mRN
WRKY	AT1G62300.1	maker-VaccDscaff6-augustus-gene-98.16-mRNA-
WRKY	AT1G62300.1	maker-VaccDscaff19-augustus-gene-273.26-mRN
WRKY	AT1G62300.1	maker-VaccDscaff15-augustus-gene-200.28-mRN
WRKY	AT1G62300.1	maker-VaccDscaff37-augustus-gene-20.21-mRNA
WRKY	AT1G62300.1	maker-VaccDscaff1-augustus-gene-368.31-mRNA
WRKY	AT1G62300.1	maker-VaccDscaff24-augustus-gene-191.23-mRN
WRKY	AT1G62300.1	maker-VaccDscaff39-augustus-gene-278.17-mRN
TALE	AT1G62360.1	maker-VaccDscaff11-snap-gene-210.26-mRNA-1
TALE	AT1G62360.1	maker-VaccDscaff19-augustus-gene-275.17-mRN
TALE	AT1G62360.1	maker-VaccDscaff15-augustus-gene-203.18-mRN
TALE	AT1G62360.1	maker-VaccDscaff24-augustus-gene-188.19-mRN
TALE	AT1G62360.1	maker-VaccDscaff34-snap-gene-185.28-mRNA-1
TALE	AT1G62990.1	maker-VaccDscaff22-snap-gene-130.28-mRNA-1
TALE	AT1G62990.1	maker-VaccDscaff43-snap-gene-237.33-mRNA-1
TALE	AT1G62990.1	maker-VaccDscaff46-augustus-gene-10.21-mRNA
ERF	AT1G63030.2	augustus_masked-VaccDscaff922-processed-gen
ERF	AT1G63030.2	augustus_masked-VaccDscaff12-processed-gene
ERF	AT1G63030.2	augustus_masked-VaccDscaff12-processed-gene

GRAS	AT1G63100.1	snap_masked-VaccDscaff43-processed-gene-241
MYB	AT1G63910.1	maker-VaccDscaff20-augustus-gene-335.23-mRN
MYB	AT1G63910.1	augustus_masked-VaccDscaff28-processed-gene
WRKY	AT1G64000.1	maker-VaccDscaff1742-snap-gene-0.8-mRNA-1
WRKY	AT1G64000.1	maker-VaccDscaff1593-snap-gene-0.8-mRNA-1
Nin-like	AT1G64530.1	maker-VaccDscaff22-augustus-gene-107.23-mRN
Nin-like	AT1G64530.1	maker-VaccDscaff43-augustus-gene-262.23-mRN
C2H2	AT1G66140.1	maker-VaccDscaff20-augustus-gene-156.15-mRN
C2H2	AT1G66140.1	maker-VaccDscaff44-augustus-gene-170.19-mRN
C2H2	AT1G66140.1	maker-VaccDscaff28-augustus-gene-167.32-mRN
GRAS	AT1G66350.1	augustus_masked-VaccDscaff25-processed-gene
GRAS	AT1G66350.1	snap_masked-VaccDscaff30-processed-gene-28.
GRAS	AT1G66350.1	augustus_masked-VaccDscaff3-processed-gene-
MYB	AT1G66370.1	maker-VaccDscaff13-snap-gene-106.35-mRNA-1
MYB	AT1G66370.1	maker-VaccDscaff13-snap-gene-106.36-mRNA-1
MYB	AT1G66370.1	maker-VaccDscaff1486-snap-gene-0.3-mRNA-1
bHLH	AT1G66470.1	maker-VaccDscaff16-augustus-gene-117.30-mRN
bHLH	AT1G66470.1	maker-VaccDscaff7-augustus-gene-316.24-mRNA
bHLH	AT1G66470.1	maker-VaccDscaff31-augustus-gene-25.24-mRNA
bHLH	AT1G66470.1	maker-VaccDscaff18-augustus-gene-278.21-mRN
CAMTA	AT1G67310.1	maker-VaccDscaff102-augustus-gene-1.31-mRNA
CAMTA	AT1G67310.1	maker-VaccDscaff102-augustus-gene-1.31-mRNA
CAMTA	AT1G67310.1	augustus_masked-VaccDscaff36-processed-gene
CAMTA	AT1G67310.1	maker-VaccDscaff16-augustus-gene-57.27-mRNA
CAMTA	AT1G67310.1	maker-VaccDscaff16-augustus-gene-57.27-mRNA
CAMTA	AT1G67310.1	maker-VaccDscaff7-snap-gene-368.33-mRNA-1
CAMTA	AT1G67310.1	maker-VaccDscaff2-augustus-gene-240.28-mRNA
CAMTA	AT1G67310.1	maker-VaccDscaff2-augustus-gene-240.28-mRNA
CAMTA	AT1G67310.1	maker-VaccDscaff14-augustus-gene-201.27-mRN
CAMTA	AT1G67310.1	maker-VaccDscaff14-augustus-gene-201.27-mRN
CAMTA	AT1G67310.1	maker-VaccDscaff9-snap-gene-225.54-mRNA-1
CAMTA	AT1G67310.1	snap_masked-VaccDscaff35-processed-gene-164
CAMTA	AT1G67310.1	snap_masked-VaccDscaff4-processed-gene-263.
CAMTA	AT1G67310.1	maker-VaccDscaff3-augustus-gene-197.29-mRNA
CAMTA	AT1G67310.1	maker-VaccDscaff3-augustus-gene-197.29-mRNA
ARR-B	AT1G67710.1	augustus_masked-VaccDscaff36-processed-gene
ARR-B	AT1G67710.1	maker-VaccDscaff9-augustus-gene-244.22-mRNA
ARR-B	AT1G67710.1	maker-VaccDscaff35-snap-gene-180.33-mRNA-1
ARR-B	AT1G67710.1	snap_masked-VaccDscaff4-processed-gene-279.
WRKY	AT1G68150.1	maker-VaccDscaff2-augustus-gene-371.29-mRNA
WRKY	AT1G68150.1	maker-VaccDscaff14-augustus-gene-320.38-mRN
MYB	AT1G68320.1	maker-VaccDscaff20-augustus-gene-132.28-mRN
MYB	AT1G68320.1	maker-VaccDscaff19-augustus-gene-134.20-mRN
MYB	AT1G68320.1	maker-VaccDscaff28-augustus-gene-214.19-mRN
bZIP	AT1G68640.1	maker-VaccDscaff20-augustus-gene-123.23-mRN
bZIP	AT1G68640.1	maker-VaccDscaff19-augustus-gene-125.17-mRN

bZIP	AT1G68640.1	maker-VaccDscaff28-augustus-gene-224.15-mRN
TCP	AT1G68800.1	snap_masked-VaccDscaff20-processed-gene-11[illegible]
TCP	AT1G68800.1	maker-VaccDscaff36-augustus-gene-102.19-mRN
TCP	AT1G68800.1	snap_masked-VaccDscaff19-processed-gene-121
TCP	AT1G68800.1	maker-VaccDscaff35-augustus-gene-247.34-mRN
TCP	AT1G68800.1	augustus_masked-VaccDscaff28-processed-gene
TCP	AT1G68800.1	snap_masked-VaccDscaff28-processed-gene-231
bHLH	AT1G68810.1	maker-VaccDscaff20-augustus-gene-119.22-mRN
bHLH	AT1G68810.1	maker-VaccDscaff25-augustus-gene-293.23-mRN
bHLH	AT1G68810.1	maker-VaccDscaff2-augustus-gene-297.28-mRN[illegible]
bHLH	AT1G68810.1	maker-VaccDscaff14-augustus-gene-251.30-mRN
bHLH	AT1G68810.1	maker-VaccDscaff19-augustus-gene-121.17-mRN
bHLH	AT1G68810.1	maker-VaccDscaff3-augustus-gene-115.18-mRN[illegible]
bHLH	AT1G68810.1	maker-VaccDscaff28-augustus-gene-228.19-mRN
bHLH	AT1G68810.1	maker-VaccDscaff28-augustus-gene-231.17-mRN
RAV	AT1G68840.2	augustus_masked-VaccDscaff2-processed-gene-[illegible]
RAV	AT1G68840.2	augustus_masked-VaccDscaff14-processed-gene
RAV	AT1G68840.2	augustus_masked-VaccDscaff3-processed-gene-[illegible]
bHLH	AT1G69010.1	maker-VaccDscaff25-snap-gene-282.30-mRNA-1
bHLH	AT1G69010.1	maker-VaccDscaff2-snap-gene-287.35-mRNA-1
bHLH	AT1G69010.1	maker-VaccDscaff14-snap-gene-243.22-mRNA-1
bHLH	AT1G69010.1	maker-VaccDscaff3-snap-gene-127.12-mRNA-1
MIKC_MADS	AT1G69120.1	maker-VaccDscaff36-snap-gene-96.32-mRNA-1
MIKC_MADS	AT1G69120.1	maker-VaccDscaff9-snap-gene-322.25-mRNA-1
MIKC_MADS	AT1G69120.1	maker-VaccDscaff4-snap-gene-346.40-mRNA-1
YABBY	AT1G69180.1	maker-VaccDscaff25-augustus-gene-285.25-mRN
YABBY	AT1G69180.1	maker-VaccDscaff2-augustus-gene-289.28-mRN[illegible]
YABBY	AT1G69180.1	maker-VaccDscaff14-augustus-gene-246.34-mRN
YABBY	AT1G69180.1	maker-VaccDscaff3-augustus-gene-123.20-mRN[illegible]
NAC	AT1G69490.1	maker-VaccDscaff10-augustus-gene-138.28-mRN
NAC	AT1G69490.1	maker-VaccDscaff25-augustus-gene-239.19-mRN
NAC	AT1G69490.1	maker-VaccDscaff2-augustus-gene-281.20-mRN[illegible]
NAC	AT1G69490.1	maker-VaccDscaff14-augustus-gene-236.21-mRN
NAC	AT1G69490.1	maker-VaccDscaff3-augustus-gene-158.19-mRN[illegible]
MYB	AT1G69560.1	maker-VaccDscaff16-augustus-gene-64.40-mRN[illegible]
MYB	AT1G69560.1	maker-VaccDscaff7-augustus-gene-359.22-mRN[illegible]
MYB	AT1G69560.1	maker-VaccDscaff18-augustus-gene-331.23-mRN
G2-like	AT1G69580.1	maker-VaccDscaff14-augustus-gene-238.13-mRN
G2-like	AT1G69580.1	maker-VaccDscaff3-snap-gene-160.14-mRNA-1
HD-ZIP	AT1G69780.1	maker-VaccDscaff36-augustus-gene-126.28-mRN
HD-ZIP	AT1G69780.1	maker-VaccDscaff2-augustus-gene-274.20-mRN[illegible]
HD-ZIP	AT1G69780.1	maker-VaccDscaff2-augustus-gene-275.28-mRN[illegible]
HD-ZIP	AT1G69780.1	maker-VaccDscaff9-augustus-gene-292.19-mRN[illegible]
HD-ZIP	AT1G69780.1	maker-VaccDscaff35-augustus-gene-224.16-mRN
HD-ZIP	AT1G69780.1	maker-VaccDscaff4-augustus-gene-317.26-mRN[illegible]
HD-ZIP	AT1G69780.1	maker-VaccDscaff94-augustus-gene-5.34-mRNA-

HD-ZIP	AT1G69780.1	maker-VaccDscaff3-augustus-gene-163.13-mRNA
MYB_related	AT1G70000.2	maker-VaccDscaff9-augustus-gene-286.18-mRNA
MYB_related	AT1G70000.2	maker-VaccDscaff35-augustus-gene-218.16-mRN
MYB_related	AT1G70000.2	snap_masked-VaccDscaff4-processed-gene-312.
TALE	AT1G70510.1	maker-VaccDscaff27-snap-gene-130.20-mRNA-1
TALE	AT1G70510.1	maker-VaccDscaff45-snap-gene-132.23-mRNA-1
TALE	AT1G70510.1	maker-VaccDscaff17-augustus-gene-167.14-mRN
TALE	AT1G70510.1	maker-VaccDscaff34-augustus-gene-185.23-mRN
Whirly	AT1G71260.1	maker-VaccDscaff20-snap-gene-237.24-mRNA-1
Whirly	AT1G71260.1	maker-VaccDscaff44-snap-gene-120.35-mRNA-1
Whirly	AT1G71260.1	maker-VaccDscaff28-augustus-gene-117.27-mRN
ERF	AT1G71450.1	augustus_masked-VaccDscaff8-processed-gene-
ERF	AT1G71450.1	augustus_masked-VaccDscaff20-processed-gene
ERF	AT1G71450.1	augustus_masked-VaccDscaff20-processed-gene
ERF	AT1G71450.1	augustus_masked-VaccDscaff20-processed-gene
ERF	AT1G71450.1	augustus_masked-VaccDscaff1-processed-gene-
ERF	AT1G71450.1	snap_masked-VaccDscaff44-processed-gene-11
ERF	AT1G71450.1	augustus_masked-VaccDscaff44-processed-gene
ERF	AT1G71450.1	augustus_masked-VaccDscaff44-processed-gene
ERF	AT1G71450.1	maker-VaccDscaff28-snap-gene-115.36-mRNA-1
ERF	AT1G71450.1	maker-VaccDscaff28-augustus-gene-116.31-mRN
MIKC_MADS	AT1G71692.1	maker-VaccDscaff14-augustus-gene-173.24-mRN
MIKC_MADS	AT1G71692.1	maker-VaccDscaff3-augustus-gene-223.26-mRNA
C2H2	AT1G72050.1	maker-VaccDscaff12-snap-gene-67.65-mRNA-1
bHLH	AT1G72210.1	maker-VaccDscaff22-augustus-gene-177.38-mRN
bHLH	AT1G72210.1	maker-VaccDscaff43-augustus-gene-187.14-mRN
bHLH	AT1G72210.1	maker-VaccDscaff12-augustus-gene-150.21-mRN
bHLH	AT1G72210.1	maker-VaccDscaff25-augustus-gene-44.27-mRNA
bHLH	AT1G72210.1	maker-VaccDscaff16-snap-gene-268.34-mRNA-1
bHLH	AT1G72210.1	maker-VaccDscaff7-augustus-gene-185.19-mRNA
bHLH	AT1G72210.1	maker-VaccDscaff31-snap-gene-213.31-mRNA-1
bHLH	AT1G72210.1	maker-VaccDscaff2-augustus-gene-63.21-mRNA-
bHLH	AT1G72210.1	maker-VaccDscaff46-augustus-gene-58.26-mRNA
bHLH	AT1G72210.1	maker-VaccDscaff110-augustus-gene-1.26-mRNA
bHLH	AT1G72210.1	maker-VaccDscaff18-augustus-gene-106.24-mRN
bHLH	AT1G72210.1	maker-VaccDscaff40-augustus-gene-72.19-mRNA
bHLH	AT1G72210.1	maker-VaccDscaff14-augustus-gene-47.25-mRNA
bHLH	AT1G72210.1	maker-VaccDscaff23-augustus-gene-261.25-mRN
AP2	AT1G72570.1	maker-VaccDscaff17-augustus-gene-386.27-mRN
NF-YA	AT1G72830.1	maker-VaccDscaff21-augustus-gene-11.32-mRNA
NF-YA	AT1G72830.1	maker-VaccDscaff26-augustus-gene-9.27-mRNA-
NF-YA	AT1G72830.1	maker-VaccDscaff29-augustus-gene-6.15-mRNA-
NF-YA	AT1G72830.2	snap_masked-VaccDscaff64-processed-gene-8.3
NF-YA	AT1G72830.2	maker-VaccDscaff30-snap-gene-10.17-mRNA-1
NF-YA	AT1G72830.2	snap_masked-VaccDscaff269-processed-gene-0.
HD-ZIP	AT1G73360.1	maker-VaccDscaff421-augustus-gene-0.22-mRNA

HD-ZIP	AT1G73360.1	maker-VaccDscaff12-snap-gene-361.41-mRNA-1
HD-ZIP	AT1G73360.1	maker-VaccDscaff13-augustus-gene-182.28-mRN
HD-ZIP	AT1G73360.1	augustus_masked-VaccDscaff31-processed-gene
HD-ZIP	AT1G73360.1	maker-VaccDscaff32-augustus-gene-188.18-mRN
HD-ZIP	AT1G73360.1	maker-VaccDscaff30-augustus-gene-204.41-mRN
EIL	AT1G73730.1	augustus_masked-VaccDscaff38-processed-gene
C2H2	AT1G74250.1	maker-VaccDscaff13-augustus-gene-236.24-mRN
C2H2	AT1G74250.1	maker-VaccDscaff32-augustus-gene-157.16-mRN
bHLH	AT1G74500.1	maker-VaccDscaff13-augustus-gene-261.12-mRN
bHLH	AT1G74500.1	maker-VaccDscaff32-augustus-gene-115.17-mRN
bHLH	AT1G74500.1	maker-VaccDscaff30-augustus-gene-141.17-mRN
bHLH	AT1G74500.1	maker-VaccDscaff30-snap-gene-141.19-mRNA-1
bHLH	AT1G74500.1	maker-VaccDscaff42-augustus-gene-174.21-mRN
MYB	AT1G74650.1	maker-VaccDscaff64-augustus-gene-8.43-mRNA-
ZF-HD	AT1G74660.1	augustus_masked-VaccDscaff45-processed-gene
ZF-HD	AT1G74660.1	augustus_masked-VaccDscaff17-processed-gene
BES1	AT1G75080.2	maker-VaccDscaff16-augustus-gene-366.27-mRN
BES1	AT1G75080.2	maker-VaccDscaff13-augustus-gene-152.16-mRN
BES1	AT1G75080.2	maker-VaccDscaff7-augustus-gene-33.28-mRNA-
BES1	AT1G75080.2	maker-VaccDscaff31-snap-gene-311.34-mRNA-1
BES1	AT1G75080.2	maker-VaccDscaff18-augustus-gene-31.39-mRNA
BES1	AT1G75080.2	maker-VaccDscaff32-augustus-gene-212.35-mRN
BES1	AT1G75080.2	maker-VaccDscaff30-augustus-gene-220.32-mRN
BES1	AT1G75080.2	maker-VaccDscaff30-augustus-gene-222.17-mRN
MYB_related	AT1G75250.1	augustus_masked-VaccDscaff12-processed-gene
MYB_related	AT1G75250.1	snap_masked-VaccDscaff16-processed-gene-370
MYB_related	AT1G75250.1	maker-VaccDscaff6-snap-gene-154.32-mRNA-1
MYB_related	AT1G75250.1	augustus_masked-VaccDscaff31-processed-gene
MYB_related	AT1G75250.1	augustus_masked-VaccDscaff18-processed-gene
MYB_related	AT1G75250.1	augustus_masked-VaccDscaff40-processed-gene
MYB_related	AT1G75250.1	snap_masked-VaccDscaff14-processed-gene-373
MYB_related	AT1G75250.1	maker-VaccDscaff30-snap-gene-127.34-mRNA-1
MYB_related	AT1G75250.1	augustus_masked-VaccDscaff23-processed-gene
bZIP	AT1G75390.1	augustus_masked-VaccDscaff126-processed-gen
bZIP	AT1G75390.1	augustus_masked-VaccDscaff12-processed-gene
bZIP	AT1G75390.1	snap_masked-VaccDscaff2-processed-gene-430.
bZIP	AT1G75390.1	snap_masked-VaccDscaff14-processed-gene-375
bZIP	AT1G75390.1	augustus_masked-VaccDscaff1259-processed-ge
TALE	AT1G75430.1	maker-VaccDscaff16-augustus-gene-373.28-mRN
TALE	AT1G75430.1	maker-VaccDscaff16-snap-gene-373.38-mRNA-1
TALE	AT1G75430.1	maker-VaccDscaff16-augustus-gene-374.40-mRN
TALE	AT1G75430.1	maker-VaccDscaff16-augustus-gene-374.39-mRN
TALE	AT1G75430.1	snap_masked-VaccDscaff16-processed-gene-374
TALE	AT1G75430.1	maker-VaccDscaff7-augustus-gene-42.42-mRNA-
TALE	AT1G75430.1	maker-VaccDscaff31-augustus-gene-319.36-mRN
TALE	AT1G75430.1	maker-VaccDscaff18-augustus-gene-21.26-mRNA

TALE	AT1G75430.1	maker-VaccDscaff18-augustus-gene-22.24-mRN/
ERF	AT1G75490.1	augustus_masked-VaccDscaff1047-processed-ge
ERF	AT1G75490.1	augustus_masked-VaccDscaff12-processed-gene
ERF	AT1G75490.1	maker-VaccDscaff16-snap-gene-375.36-mRNA-1
ERF	AT1G75490.1	snap_masked-VaccDscaff7-processed-gene-43.8
ERF	AT1G75490.1	maker-VaccDscaff31-snap-gene-319.46-mRNA-1
ERF	AT1G75490.1	snap_masked-VaccDscaff17-processed-gene-111
ERF	AT1G75490.1	snap_masked-VaccDscaff18-processed-gene-20.
SRS	AT1G75520.1	maker-VaccDscaff16-augustus-gene-376.14-mRN
SRS	AT1G75520.1	maker-VaccDscaff7-augustus-gene-43.29-mRNA-
SRS	AT1G75520.1	maker-VaccDscaff31-augustus-gene-320.14-mRN
SRS	AT1G75520.1	maker-VaccDscaff18-augustus-gene-20.18-mRN/
SRS	AT1G75520.1	snap_masked-VaccDscaff32-processed-gene-94.
DBB	AT1G75540.1	maker-VaccDscaff16-augustus-gene-376.13-mRN
DBB	AT1G75540.1	augustus_masked-VaccDscaff13-processed-gene
DBB	AT1G75540.1	maker-VaccDscaff32-augustus-gene-95.27-mRN/
DBB	AT1G75540.1	maker-VaccDscaff30-augustus-gene-115.42-mRN
DBB	AT1G75540.1	maker-VaccDscaff42-snap-gene-196.36-mRNA-1
C2H2	AT1G75710.1	maker-VaccDscaff16-augustus-gene-380.25-mRN
C2H2	AT1G75710.1	maker-VaccDscaff7-augustus-gene-47.34-mRNA-
C2H2	AT1G75710.1	maker-VaccDscaff31-augustus-gene-324.29-mRN
C2H2	AT1G75710.1	maker-VaccDscaff18-augustus-gene-16.30-mRN/
NAC	AT1G76420.1	maker-VaccDscaff12-snap-gene-14.33-mRNA-1
NAC	AT1G76420.1	maker-VaccDscaff12-snap-gene-32.51-mRNA-1
NAC	AT1G76420.1	maker-VaccDscaff16-augustus-gene-389.35-mRN
NAC	AT1G76420.1	maker-VaccDscaff23-augustus-gene-363.28-mRN
Trihelix	AT1G76880.1	maker-VaccDscaff2-augustus-gene-312.31-mRN/
Trihelix	AT1G76880.1	maker-VaccDscaff3-augustus-gene-99.26-mRNA-
NAC	AT1G77450.1	maker-VaccDscaff11-snap-gene-48.29-mRNA-1
NAC	AT1G77450.1	maker-VaccDscaff11-snap-gene-48.29-mRNA-1
NAC	AT1G77450.1	maker-VaccDscaff19-augustus-gene-150.22-mRN
NAC	AT1G77450.1	maker-VaccDscaff15-snap-gene-56.32-mRNA-1
NAC	AT1G77450.1	maker-VaccDscaff15-snap-gene-56.32-mRNA-1
NAC	AT1G77450.1	maker-VaccDscaff24-snap-gene-324.28-mRNA-1
NAC	AT1G77450.1	maker-VaccDscaff24-snap-gene-324.28-mRNA-1
ERF	AT1G78080.1	augustus_masked-VaccDscaff12-processed-gene
ERF	AT1G78080.1	snap_masked-VaccDscaff40-processed-gene-91.
ERF	AT1G78080.1	augustus_masked-VaccDscaff41-processed-gene
ERF	AT1G78080.1	maker-VaccDscaff4-snap-gene-79.32-mRNA-1
ERF	AT1G78080.1	augustus_masked-VaccDscaff23-processed-gene
DBB	AT1G78600.1	maker-VaccDscaff515-snap-gene-0.28-mRNA-1
DBB	AT1G78600.1	maker-VaccDscaff553-augustus-gene-0.13-mRN/
BES1	AT1G78700.1	maker-VaccDscaff9-augustus-gene-181.33-mRN/
BES1	AT1G78700.1	augustus_masked-VaccDscaff35-processed-gene
BES1	AT1G78700.1	maker-VaccDscaff4-augustus-gene-215.27-mRN/
MYB	AT1G79180.1	maker-VaccDscaff151-augustus-gene-2.41-mRN/

MYB	AT1G79180.1	maker-VaccDscaff6-augustus-gene-4.41-mRNA-1
G2-like	AT1G79430.2	maker-VaccDscaff6-augustus-gene-1.18-mRNA-1
HD-ZIP	AT1G79840.1	maker-VaccDscaff36-snap-gene-2.36-mRNA-1
HD-ZIP	AT1G79840.1	maker-VaccDscaff9-snap-gene-393.36-mRNA-1
HD-ZIP	AT1G79840.1	maker-VaccDscaff35-snap-gene-304.40-mRNA-1
HD-ZIP	AT1G79840.1	maker-VaccDscaff4-augustus-gene-423.32-mRN/
ERF	AT1G80580.1	augustus_masked-VaccDscaff27-processed-gene
ERF	AT1G80580.1	snap_masked-VaccDscaff17-processed-gene-336
ERF	AT1G80580.1	snap_masked-VaccDscaff50-processed-gene-25.
WRKY	AT1G80840.1	maker-VaccDscaff27-augustus-gene-292.21-mRN
WRKY	AT1G80840.1	maker-VaccDscaff36-augustus-gene-33.17-mRN/
WRKY	AT1G80840.1	snap_masked-VaccDscaff17-processed-gene-328
WRKY	AT1G80840.1	snap_masked-VaccDscaff9-processed-gene-377.
WRKY	AT1G80840.1	maker-VaccDscaff35-augustus-gene-292.32-mRN
WRKY	AT1G80840.1	maker-VaccDscaff34-augustus-gene-51.32-mRN/
WRKY	AT1G80840.1	maker-VaccDscaff4-augustus-gene-398.15-mRN/
G2-like	AT2G01060.1	maker-VaccDscaff36-augustus-gene-294.23-mRN
G2-like	AT2G01060.1	snap_masked-VaccDscaff11-processed-gene-220
G2-like	AT2G01060.1	snap_masked-VaccDscaff11-processed-gene-220
G2-like	AT2G01060.1	augustus_masked-VaccDscaff26-processed-gene
G2-like	AT2G01060.1	maker-VaccDscaff5-augustus-gene-314.25-mRN/
G2-like	AT2G01060.1	maker-VaccDscaff15-augustus-gene-218.26-mRN
G2-like	AT2G01060.1	maker-VaccDscaff9-augustus-gene-107.34-mRN/
G2-like	AT2G01060.1	maker-VaccDscaff35-augustus-gene-62.30-mRN/
G2-like	AT2G01060.1	maker-VaccDscaff24-augustus-gene-172.24-mRN
G2-like	AT2G01060.1	maker-VaccDscaff82-augustus-gene-3.28-mRNA-
G2-like	AT2G01060.1	maker-VaccDscaff4-augustus-gene-153.22-mRN/
G2-like	AT2G01060.1	maker-VaccDscaff29-augustus-gene-296.28-mRN
HD-ZIP	AT2G01430.1	maker-VaccDscaff44-augustus-gene-193.29-mRN
HD-ZIP	AT2G01430.1	maker-VaccDscaff28-augustus-gene-194.33-mRN
GRAS	AT2G01570.1	snap_masked-VaccDscaff21-processed-gene-187
GRAS	AT2G01570.1	snap_masked-VaccDscaff21-processed-gene-187
GRAS	AT2G01570.1	maker-VaccDscaff25-snap-gene-86.23-mRNA-1
GRAS	AT2G01570.1	augustus_masked-VaccDscaff30-processed-gene
GRAS	AT2G01570.1	snap_masked-VaccDscaff300-processed-gene-0.
GRAS	AT2G01570.1	maker-VaccDscaff3-augustus-gene-357.27-mRN/
GRAS	AT2G01570.1	augustus_masked-VaccDscaff3-processed-gene-:
BBR-BPC	AT2G01930.2	augustus_masked-VaccDscaff20-processed-gene
BBR-BPC	AT2G01930.2	maker-VaccDscaff28-augustus-gene-162.20-mRN
C2H2	AT2G01940.1	maker-VaccDscaff20-augustus-gene-163.20-mRN
C2H2	AT2G01940.1	maker-VaccDscaff44-augustus-gene-162.31-mRN
C2H2	AT2G01940.1	augustus_masked-VaccDscaff876-processed-gen
C2H2	AT2G01940.1	maker-VaccDscaff28-augustus-gene-161.35-mRN
NAC	AT2G02450.1	maker-VaccDscaff25-augustus-gene-349.28-mRN
NAC	AT2G02450.1	maker-VaccDscaff2-augustus-gene-352.14-mRN/
NAC	AT2G02450.1	maker-VaccDscaff14-augustus-gene-299.20-mRN

NAC	AT2G02450.1	maker-VaccDscaff3-augustus-gene-54.33-mRNA-
NAC	AT2G02450.2	maker-VaccDscaff27-augustus-gene-112.26-mRN
NAC	AT2G02450.2	maker-VaccDscaff45-augustus-gene-109.21-mRN
NAC	AT2G02450.2	maker-VaccDscaff17-augustus-gene-148.26-mRN
NAC	AT2G02450.2	maker-VaccDscaff34-augustus-gene-203.16-mRN
G2-like	AT2G03500.1	maker-VaccDscaff41-augustus-gene-219.27-mRN
MIKC_MADS	AT2G03710.2	maker-VaccDscaff9-snap-gene-322.27-mRNA-1
NF-YB	AT2G13570.1	augustus_masked-VaccDscaff38-processed-gene
NF-YB	AT2G13570.1	snap_masked-VaccDscaff6-processed-gene-293.
NF-YB	AT2G13570.1	augustus_masked-VaccDscaff37-processed-gene
NF-YB	AT2G13570.1	maker-VaccDscaff37-augustus-gene-179.37-mRN
NF-YB	AT2G13570.1	augustus_masked-VaccDscaff39-processed-gene
MIKC_MADS	AT2G14210.1	snap_masked-VaccDscaff31-processed-gene-28.
MIKC_MADS	AT2G14210.1	snap_masked-VaccDscaff41-processed-gene-10.
MIKC_MADS	AT2G14210.1	maker-VaccDscaff23-augustus-gene-280.35-mRN
bHLH	AT2G14760.1	maker-VaccDscaff39-snap-gene-255.40-mRNA-1
bHLH	AT2G14760.3	maker-VaccDscaff6-snap-gene-135.38-mRNA-1
C3H	AT2G16485.1	maker-VaccDscaff126-augustus-gene-1.33-mRNA
C3H	AT2G16485.1	maker-VaccDscaff126-augustus-gene-1.33-mRNA
C3H	AT2G16485.1	maker-VaccDscaff14-augustus-gene-380.29-mRN
C3H	AT2G16485.1	maker-VaccDscaff14-augustus-gene-380.29-mRN
MYB	AT2G16720.1	maker-VaccDscaff21-snap-gene-13.33-mRNA-1
MYB	AT2G16720.1	maker-VaccDscaff33-snap-gene-307.38-mRNA-1
NAC	AT2G17040.1	maker-VaccDscaff13-augustus-gene-314.23-mRN
NAC	AT2G17040.1	maker-VaccDscaff2-augustus-gene-443.32-mRNA
NAC	AT2G17040.1	maker-VaccDscaff41-augustus-gene-275.38-mRN
NAC	AT2G17040.1	maker-VaccDscaff14-augustus-gene-390.33-mRN
NAC	AT2G17040.1	maker-VaccDscaff32-augustus-gene-66.25-mRNA
NAC	AT2G17040.1	maker-VaccDscaff30-augustus-gene-82.49-mRNA
NAC	AT2G17040.1	maker-VaccDscaff42-augustus-gene-212.26-mRN
ZF-HD	AT2G18350.1	augustus_masked-VaccDscaff27-processed-gene
ZF-HD	AT2G18350.1	augustus_masked-VaccDscaff45-processed-gene
ZF-HD	AT2G18350.1	augustus_masked-VaccDscaff40-processed-gene
ZF-HD	AT2G18350.1	augustus_masked-VaccDscaff34-processed-gene
ZF-HD	AT2G18350.1	maker-VaccDscaff34-snap-gene-221.26-mRNA-1
HD-ZIP	AT2G18550.1	maker-VaccDscaff13-augustus-gene-153.25-mRN
HD-ZIP	AT2G18550.1	maker-VaccDscaff32-augustus-gene-211.24-mRN
HD-ZIP	AT2G18550.1	maker-VaccDscaff30-augustus-gene-219.29-mRN
HD-ZIP	AT2G18550.1	maker-VaccDscaff42-augustus-gene-84.31-mRNA
C3H	AT2G19810.1	maker-VaccDscaff13-augustus-gene-337.26-mRN
C3H	AT2G19810.1	maker-VaccDscaff32-augustus-gene-18.24-mRNA
C3H	AT2G19810.1	maker-VaccDscaff30-augustus-gene-62.27-mRNA
C3H	AT2G19810.1	maker-VaccDscaff42-augustus-gene-265.20-mRN
C3H	AT2G20280.1	maker-VaccDscaff21-augustus-gene-167.26-mRN
C3H	AT2G20280.1	maker-VaccDscaff194-augustus-gene-0.25-mRNA
C3H	AT2G20280.1	maker-VaccDscaff33-augustus-gene-178.24-mRN

C3H	AT2G20280.1	maker-VaccDscaff26-augustus-gene-171.36-mRN
ERF	AT2G20880.1	maker-VaccDscaff46-snap-gene-88.33-mRNA-1
BBR-BPC	AT2G21240.2	maker-VaccDscaff126-augustus-gene-0.25-mRNA
BBR-BPC	AT2G21240.2	maker-VaccDscaff13-augustus-gene-288.27-mRN
BBR-BPC	AT2G21240.2	maker-VaccDscaff2-snap-gene-434.24-mRNA-1
BBR-BPC	AT2G21240.2	maker-VaccDscaff14-augustus-gene-381.19-mRN
BBR-BPC	AT2G21240.2	maker-VaccDscaff32-augustus-gene-99.34-mRNA
BBR-BPC	AT2G21240.2	maker-VaccDscaff30-augustus-gene-108.25-mRN
BBR-BPC	AT2G21240.2	maker-VaccDscaff30-augustus-gene-111.28-mRN
CAMTA	AT2G22300.2	maker-VaccDscaff38-augustus-gene-81.24-mRNA
CAMTA	AT2G22300.2	maker-VaccDscaff6-augustus-gene-337.17-mRNA
CAMTA	AT2G22300.2	augustus_masked-VaccDscaff37-processed-gene
CAMTA	AT2G22300.2	maker-VaccDscaff39-augustus-gene-86.17-mRNA
HD-ZIP	AT2G22430.1	maker-VaccDscaff38-augustus-gene-74.25-mRNA
HD-ZIP	AT2G22430.1	maker-VaccDscaff6-augustus-gene-344.23-mRNA
HD-ZIP	AT2G22430.1	maker-VaccDscaff39-augustus-gene-82.12-mRNA
GRF	AT2G22840.1	maker-VaccDscaff41-augustus-gene-161.25-mRN
GRF	AT2G22840.1	maker-VaccDscaff41-augustus-gene-161.25-mRN
MYB	AT2G23290.1	augustus_masked-VaccDscaff30-processed-gene
ERF	AT2G23340.1	augustus_masked-VaccDscaff12-processed-gene
ERF	AT2G23340.1	augustus_masked-VaccDscaff16-processed-gene
ERF	AT2G23340.1	augustus_masked-VaccDscaff13-processed-gene
ERF	AT2G23340.1	augustus_masked-VaccDscaff7-processed-gene-
ERF	AT2G23340.1	augustus_masked-VaccDscaff31-processed-gene
ERF	AT2G23340.1	augustus_masked-VaccDscaff40-processed-gene
ERF	AT2G23340.1	augustus_masked-VaccDscaff41-processed-gene
ERF	AT2G23340.1	augustus_masked-VaccDscaff260-processed-gen
ERF	AT2G23340.1	augustus_masked-VaccDscaff32-processed-gene
ERF	AT2G23340.1	augustus_masked-VaccDscaff30-processed-gene
ERF	AT2G23340.1	augustus_masked-VaccDscaff23-processed-gene
NAC	AT2G24430.2	maker-VaccDscaff25-augustus-gene-131.19-mRN
NAC	AT2G24430.2	maker-VaccDscaff2-augustus-gene-109.35-mRNA
NAC	AT2G24430.2	maker-VaccDscaff3-augustus-gene-263.20-mRNA
C3H	AT2G24830.1	snap_masked-VaccDscaff20-processed-gene-331
C3H	AT2G24830.1	maker-VaccDscaff44-snap-gene-52.50-mRNA-1
C3H	AT2G24830.1	snap_masked-VaccDscaff28-processed-gene-53.
M-type_MADS	AT2G24840.1	augustus_masked-VaccDscaff1-processed-gene-
M-type_MADS	AT2G24840.1	augustus_masked-VaccDscaff28-processed-gene
ARR-B	AT2G25180.1	maker-VaccDscaff153-augustus-gene-1.40-mRNA
ARR-B	AT2G25180.1	maker-VaccDscaff16-snap-gene-227.30-mRNA-1
ARR-B	AT2G25180.1	maker-VaccDscaff7-snap-gene-176.22-mRNA-1
ARR-B	AT2G25180.1	maker-VaccDscaff31-snap-gene-168.18-mRNA-1
ARR-B	AT2G25180.1	maker-VaccDscaff18-augustus-gene-176.29-mRN
YABBY	AT2G26580.2	maker-VaccDscaff12-snap-gene-352.28-mRNA-1
YABBY	AT2G26580.2	maker-VaccDscaff40-augustus-gene-250.29-mRN
YABBY	AT2G26580.2	maker-VaccDscaff41-augustus-gene-233.31-mRN

YABBY	AT2G26580.2	maker-VaccDscaff23-snap-gene-34.32-mRNA-1
NAC	AT2G27300.1	maker-VaccDscaff22-augustus-gene-235.30-mRN
NAC	AT2G27300.1	maker-VaccDscaff11-snap-gene-114.33-mRNA-1
NAC	AT2G27300.1	maker-VaccDscaff46-augustus-gene-109.23-mRN
NAC	AT2G27300.1	maker-VaccDscaff19-snap-gene-199.27-mRNA-1
NAC	AT2G27300.1	maker-VaccDscaff15-snap-gene-109.37-mRNA-1
NAC	AT2G27300.1	maker-VaccDscaff24-augustus-gene-291.27-mRN
NAC	AT2G27300.1	maker-VaccDscaff47-augustus-gene-45.30-mRNA
NAC	AT2G27300.1	maker-VaccDscaff47-augustus-gene-48.21-mRNA
NF-YB	AT2G27470.1	augustus_masked-VaccDscaff11-processed-gene
NF-YB	AT2G27470.1	maker-VaccDscaff19-augustus-gene-312.35-mRN
NF-YB	AT2G27470.1	maker-VaccDscaff15-augustus-gene-265.32-mRN
NF-YB	AT2G27470.1	maker-VaccDscaff24-augustus-gene-119.35-mRN
ARF	AT2G28350.1	maker-VaccDscaff11-augustus-gene-302.17-mRN
ARF	AT2G28350.1	maker-VaccDscaff19-augustus-gene-333.25-mRN
ARF	AT2G28350.1	maker-VaccDscaff15-augustus-gene-286.20-mRN
ARF	AT2G28350.1	maker-VaccDscaff24-augustus-gene-99.15-mRNA
C3H	AT2G28450.1	maker-VaccDscaff11-augustus-gene-304.27-mRN
C3H	AT2G28450.1	maker-VaccDscaff15-snap-gene-288.25-mRNA-1
C3H	AT2G28450.1	maker-VaccDscaff24-augustus-gene-98.19-mRNA
C3H	AT2G28450.2	maker-VaccDscaff11-augustus-gene-304.28-mRN
C3H	AT2G28450.2	maker-VaccDscaff19-augustus-gene-335.38-mRN
AP2	AT2G28550.1	maker-VaccDscaff19-snap-gene-59.43-mRNA-1
AP2	AT2G28550.1	maker-VaccDscaff20-snap-gene-63.39-mRNA-1
AP2	AT2G28550.3	augustus_masked-VaccDscaff1-processed-gene-
AP2	AT2G28550.3	maker-VaccDscaff11-augustus-gene-310.23-mRN
AP2	AT2G28550.3	maker-VaccDscaff19-snap-gene-340.36-mRNA-1
AP2	AT2G28550.3	maker-VaccDscaff22-augustus-gene-269.24-mRN
AP2	AT2G28550.3	maker-VaccDscaff28-augustus-gene-296.28-mRN
AP2	AT2G28550.3	maker-VaccDscaff43-augustus-gene-119.38-mRN
AP2	AT2G28550.3	maker-VaccDscaff47-augustus-gene-85.17-mRNA
AP2	AT2G28550.3	maker-VaccDscaff48-augustus-gene-60.37-mRNA
AP2	AT2G28550.3	maker-VaccDscaff8-augustus-gene-249.8-mRNA-
AP2	AT2G28550.3	snap_masked-VaccDscaff15-processed-gene-29
WOX	AT2G28610.1	maker-VaccDscaff46-augustus-gene-124.33-mRN
WOX	AT2G28610.1	maker-VaccDscaff28-augustus-gene-192.27-mRN
C2H2	AT2G28710.1	augustus_masked-VaccDscaff20-processed-gene
C2H2	AT2G28710.1	augustus_masked-VaccDscaff19-processed-gene
C2H2	AT2G28710.1	augustus_masked-VaccDscaff19-processed-gene
C2H2	AT2G28710.1	snap_masked-VaccDscaff37-processed-gene-28
C2H2	AT2G28710.1	augustus_masked-VaccDscaff28-processed-gene
C2H2	AT2G28710.1	augustus_masked-VaccDscaff48-processed-gene
C2H2	AT2G29660.1	augustus_masked-VaccDscaff19-processed-gene
C2H2	AT2G29660.1	augustus_masked-VaccDscaff15-processed-gene
C2H2	AT2G29660.1	augustus_masked-VaccDscaff24-processed-gene
C2H2	AT2G29660.1	augustus_masked-VaccDscaff221-processed-gen

LBD	AT2G30130.1	maker-VaccDscaff21-augustus-gene-119.38-mRN
LBD	AT2G30130.1	maker-VaccDscaff20-snap-gene-36.38-mRNA-1
LBD	AT2G30130.1	maker-VaccDscaff33-snap-gene-217.28-mRNA-1
LBD	AT2G30130.1	maker-VaccDscaff33-snap-gene-217.28-mRNA-1
LBD	AT2G30130.1	maker-VaccDscaff11-augustus-gene-319.21-mRN
LBD	AT2G30130.1	maker-VaccDscaff26-augustus-gene-123.41-mRN
LBD	AT2G30130.1	maker-VaccDscaff19-augustus-gene-37.43-mRNA
LBD	AT2G30130.1	maker-VaccDscaff19-augustus-gene-366.31-mRN
LBD	AT2G30130.1	maker-VaccDscaff15-augustus-gene-307.19-mRN
LBD	AT2G30130.1	maker-VaccDscaff15-augustus-gene-334.23-mRN
LBD	AT2G30130.1	maker-VaccDscaff24-augustus-gene-66.43-mRNA
LBD	AT2G30130.1	maker-VaccDscaff214-augustus-gene-0.26-mRNA
LBD	AT2G30130.1	maker-VaccDscaff29-augustus-gene-120.26-mRN
LBD	AT2G30130.1	maker-VaccDscaff28-augustus-gene-319.25-mRN
B3	AT2G30470.1	maker-VaccDscaff43-augustus-gene-76.26-mRNA
B3	AT2G30470.1	maker-VaccDscaff31-augustus-gene-121.34-mRN
WRKY	AT2G30590.1	augustus_masked-VaccDscaff22-processed-gene
WRKY	AT2G30590.1	snap_masked-VaccDscaff22-processed-gene-34(
WRKY	AT2G30590.1	maker-VaccDscaff11-augustus-gene-315.26-mRN
WRKY	AT2G30590.1	maker-VaccDscaff46-snap-gene-175.41-mRNA-1
WRKY	AT2G30590.1	maker-VaccDscaff19-augustus-gene-370.30-mRN
WRKY	AT2G30590.1	maker-VaccDscaff15-augustus-gene-303.35-mRN
WRKY	AT2G30590.1	augustus_masked-VaccDscaff24-processed-gene
WRKY	AT2G30590.1	maker-VaccDscaff47-snap-gene-167.28-mRNA-1
MYB	AT2G31180.1	maker-VaccDscaff22-augustus-gene-332.54-mRN
MYB	AT2G31180.1	maker-VaccDscaff38-augustus-gene-244.13-mRN
MYB	AT2G31180.1	maker-VaccDscaff43-augustus-gene-40.46-mRNA
MYB	AT2G31180.1	maker-VaccDscaff11-augustus-gene-368.37-mRN
MYB	AT2G31180.1	maker-VaccDscaff6-augustus-gene-53.22-mRNA-
MYB	AT2G31180.1	augustus_masked-VaccDscaff6-processed-gene-
MYB	AT2G31180.1	augustus_masked-VaccDscaff46-processed-gene
MYB	AT2G31180.1	maker-VaccDscaff15-augustus-gene-360.46-mRN
MYB	AT2G31180.1	maker-VaccDscaff37-augustus-gene-38.13-mRNA
MYB	AT2G31180.1	maker-VaccDscaff24-augustus-gene-34.41-mRNA
MYB	AT2G31180.1	maker-VaccDscaff52-augustus-gene-13.23-mRNA
MYB	AT2G31180.1	maker-VaccDscaff47-augustus-gene-159.52-mRN
bHLH	AT2G31220.1	maker-VaccDscaff12-snap-gene-246.38-mRNA-1
bHLH	AT2G31220.1	maker-VaccDscaff41-snap-gene-118.25-mRNA-1
bHLH	AT2G31220.1	maker-VaccDscaff23-augustus-gene-170.31-mRN
MYB	AT2G32460.1	maker-VaccDscaff10-augustus-gene-60.45-mRNA
MYB	AT2G32460.1	maker-VaccDscaff8-snap-gene-10.35-mRNA-1
MYB	AT2G32460.1	maker-VaccDscaff8-snap-gene-10.34-mRNA-1
MYB	AT2G32460.1	maker-VaccDscaff8-snap-gene-11.49-mRNA-1
MYB	AT2G32460.1	maker-VaccDscaff8-snap-gene-11.48-mRNA-1
MYB	AT2G32460.1	maker-VaccDscaff8-augustus-gene-87.22-mRNA-
MYB	AT2G32460.1	maker-VaccDscaff83-snap-gene-5.40-mRNA-1

MYB	AT2G32460.1	maker-VaccDscaff6-augustus-gene-101.19-mRNA
MYB	AT2G32460.1	maker-VaccDscaff5-augustus-gene-346.37-mRNA
MYB	AT2G32460.1	maker-VaccDscaff5-snap-gene-414.45-mRNA-1
MYB	AT2G32460.1	maker-VaccDscaff5-snap-gene-414.44-mRNA-1
MYB	AT2G32460.1	maker-VaccDscaff9-snap-gene-119.37-mRNA-1
MYB	AT2G32460.1	maker-VaccDscaff1-augustus-gene-367.20-mRNA
MYB	AT2G32460.1	snap_masked-VaccDscaff1-processed-gene-449.
MYB	AT2G32460.1	maker-VaccDscaff4-snap-gene-166.41-mRNA-1
MYB	AT2G32460.2	maker-VaccDscaff39-augustus-gene-273.22-mRN
Trihelix	AT2G33550.1	maker-VaccDscaff623-augustus-gene-0.5-mRNA-
Trihelix	AT2G33550.1	maker-VaccDscaff124-augustus-gene-3.45-mRNA
Trihelix	AT2G33550.1	maker-VaccDscaff2-augustus-gene-233.18-mRNA
Trihelix	AT2G33550.1	maker-VaccDscaff14-augustus-gene-193.15-mRN
ERF	AT2G33710.1	maker-VaccDscaff25-augustus-gene-316.25-mRN
ERF	AT2G33710.1	maker-VaccDscaff2-augustus-gene-319.14-mRNA
ERF	AT2G33710.1	maker-VaccDscaff14-augustus-gene-273.30-mRN
SBP	AT2G33810.1	maker-VaccDscaff11-snap-gene-157.27-mRNA-1
SBP	AT2G33810.1	maker-VaccDscaff19-snap-gene-223.19-mRNA-1
SBP	AT2G33810.1	augustus_masked-VaccDscaff15-processed-gene
ARF	AT2G33860.1	maker-VaccDscaff9-augustus-gene-167.14-mRNA
ARF	AT2G33860.1	augustus_masked-VaccDscaff35-processed-gene
ARF	AT2G33860.1	maker-VaccDscaff4-augustus-gene-230.27-mRNA
Dof	AT2G34140.1	augustus_masked-VaccDscaff11-processed-gene
Dof	AT2G34140.1	augustus_masked-VaccDscaff19-processed-gene
Dof	AT2G34140.1	snap_masked-VaccDscaff24-processed-gene-25
Dof	AT2G34140.1	snap_masked-VaccDscaff248-processed-gene-0.
HD-ZIP	AT2G34710.1	maker-VaccDscaff28-augustus-gene-136.10-mRN
WRKY	AT2G34830.1	maker-VaccDscaff24-augustus-gene-4.31-mRNA-
bZIP	AT2G35530.1	maker-VaccDscaff22-augustus-gene-184.18-mRN
bZIP	AT2G35530.1	maker-VaccDscaff2-augustus-gene-53.18-mRNA-
bZIP	AT2G35530.1	maker-VaccDscaff46-augustus-gene-66.21-mRNA
bZIP	AT2G35530.1	maker-VaccDscaff46-augustus-gene-68.28-mRNA
bZIP	AT2G35530.1	maker-VaccDscaff14-augustus-gene-38.22-mRNA
bZIP	AT2G35530.1	maker-VaccDscaff3-snap-gene-390.33-mRNA-1
bZIP	AT2G35530.1	augustus_masked-VaccDscaff3-processed-gene-
bZIP	AT2G35530.1	maker-VaccDscaff47-augustus-gene-6.27-mRNA-
BBR-BPC	AT2G35550.1	snap_masked-VaccDscaff16-processed-gene-35
BBR-BPC	AT2G35550.4	augustus_masked-VaccDscaff7-processed-gene-
ERF	AT2G35700.1	augustus_masked-VaccDscaff218-processed-gen
ERF	AT2G35700.1	augustus_masked-VaccDscaff3-processed-gene-
ERF	AT2G35700.1	augustus_masked-VaccDscaff91-processed-gene
ERF	AT2G35700.1	augustus_masked-VaccDscaff197-processed-gen
TALE	AT2G35940.3	maker-VaccDscaff10-augustus-gene-199.19-mRN
TALE	AT2G35940.3	maker-VaccDscaff8-augustus-gene-212.24-mRNA
TALE	AT2G35940.3	maker-VaccDscaff5-augustus-gene-215.20-mRNA
TALE	AT2G35940.3	maker-VaccDscaff1-augustus-gene-209.13-mRNA

E2F/DP	AT2G36010.1	maker-VaccDscaff21-augustus-gene-278.22-mRN
E2F/DP	AT2G36010.1	maker-VaccDscaff33-augustus-gene-68.23-mRN/
E2F/DP	AT2G36010.1	maker-VaccDscaff29-augustus-gene-254.22-mRN
E2F/DP	AT2G36010.3	maker-VaccDscaff26-augustus-gene-265.23-mRN
B3	AT2G36080.1	maker-VaccDscaff29-augustus-gene-250.19-mRN
ERF	AT2G36450.1	snap_masked-VaccDscaff21-processed-gene-251
ERF	AT2G36450.1	augustus_masked-VaccDscaff33-processed-gene
ERF	AT2G36450.1	augustus_masked-VaccDscaff26-processed-gene
ERF	AT2G36450.1	augustus_masked-VaccDscaff29-processed-gene
MYB	AT2G36890.1	maker-VaccDscaff13-snap-gene-337.33-mRNA-1
MYB	AT2G36890.1	maker-VaccDscaff30-snap-gene-62.32-mRNA-1
MYB	AT2G36890.1	maker-VaccDscaff42-augustus-gene-265.19-mRN
MYB	AT2G36890.1	maker-VaccDscaff3-snap-gene-423.28-mRNA-1
MYB_related	AT2G36960.3	maker-VaccDscaff21-augustus-gene-228.19-mRN
MYB_related	AT2G36960.3	maker-VaccDscaff33-augustus-gene-127.28-mRN
MYB_related	AT2G36960.3	maker-VaccDscaff26-augustus-gene-225.20-mRN
MYB_related	AT2G36960.3	maker-VaccDscaff29-augustus-gene-216.25-mRN
NF-YB	AT2G37060.3	maker-VaccDscaff10-snap-gene-231.33-mRNA-1
NF-YB	AT2G37060.3	maker-VaccDscaff10-snap-gene-239.35-mRNA-1
NF-YB	AT2G37060.3	maker-VaccDscaff8-augustus-gene-248.18-mRN/
NF-YB	AT2G37060.3	maker-VaccDscaff20-augustus-gene-63.32-mRN/
NF-YB	AT2G37060.3	maker-VaccDscaff5-snap-gene-166.17-mRNA-1
NF-YB	AT2G37060.3	maker-VaccDscaff19-augustus-gene-59.36-mRN/
NF-YB	AT2G37060.3	maker-VaccDscaff1-snap-gene-176.39-mRNA-1
NF-YB	AT2G37060.3	maker-VaccDscaff28-snap-gene-296.33-mRNA-1
NF-YB	AT2G37060.3	maker-VaccDscaff48-snap-gene-60.42-mRNA-1
S1Fa-like	AT2G37120.1	maker-VaccDscaff10-snap-gene-228.28-mRNA-1
S1Fa-like	AT2G37120.1	maker-VaccDscaff8-snap-gene-244.22-mRNA-1
S1Fa-like	AT2G37120.1	maker-VaccDscaff5-snap-gene-169.23-mRNA-1
S1Fa-like	AT2G37120.1	maker-VaccDscaff1-snap-gene-180.21-mRNA-1
MYB	AT2G37630.1	snap_masked-VaccDscaff27-processed-gene-32.
MYB	AT2G37630.1	maker-VaccDscaff17-augustus-gene-52.12-mRN/
MYB	AT2G37630.1	maker-VaccDscaff34-augustus-gene-309.24-mRN
GRAS	AT2G37650.1	augustus_masked-VaccDscaff10-processed-gene
GRAS	AT2G37650.1	augustus_masked-VaccDscaff8-processed-gene-
GRAS	AT2G37650.1	augustus_masked-VaccDscaff5-processed-gene-
GRAS	AT2G37650.1	augustus_masked-VaccDscaff1-processed-gene-
C2H2	AT2G37740.1	augustus_masked-VaccDscaff10-processed-gene
C2H2	AT2G37740.1	augustus_masked-VaccDscaff8-processed-gene-
C2H2	AT2G37740.1	augustus_masked-VaccDscaff21-processed-gene
C2H2	AT2G37740.1	augustus_masked-VaccDscaff33-processed-gene
C2H2	AT2G37740.1	augustus_masked-VaccDscaff26-processed-gene
C2H2	AT2G37740.1	augustus_masked-VaccDscaff5-processed-gene-
C2H2	AT2G37740.1	augustus_masked-VaccDscaff1-processed-gene-
MYB	AT2G38090.1	maker-VaccDscaff21-snap-gene-131.29-mRNA-1
MYB	AT2G38090.1	maker-VaccDscaff27-augustus-gene-31.26-mRN/

MYB	AT2G38090.1	maker-VaccDscaff20-augustus-gene-44.44-mRN/
MYB	AT2G38090.1	maker-VaccDscaff33-augustus-gene-204.30-mRN
MYB	AT2G38090.1	maker-VaccDscaff45-augustus-gene-33.23-mRN/
MYB	AT2G38090.1	maker-VaccDscaff26-augustus-gene-135.23-mRN
MYB	AT2G38090.1	maker-VaccDscaff17-augustus-gene-60.28-mRN/
MYB	AT2G38090.1	augustus_masked-VaccDscaff19-processed-gene
MYB	AT2G38090.1	maker-VaccDscaff15-augustus-gene-315.33-mRN
MYB	AT2G38090.1	maker-VaccDscaff34-augustus-gene-259.28-mRN
MYB	AT2G38090.1	maker-VaccDscaff29-snap-gene-132.47-mRNA-1
MYB	AT2G38090.1	maker-VaccDscaff28-augustus-gene-311.40-mRN
MYB	AT2G38090.1	augustus_masked-VaccDscaff48-processed-gene
G2-like	AT2G38300.1	maker-VaccDscaff43-augustus-gene-87.26-mRN/
G2-like	AT2G38300.1	maker-VaccDscaff9-augustus-gene-179.19-mRN/
G2-like	AT2G38300.1	maker-VaccDscaff35-augustus-gene-126.21-mRN
WRKY	AT2G38470.1	maker-VaccDscaff43-augustus-gene-91.27-mRN/
WRKY	AT2G38470.1	maker-VaccDscaff27-augustus-gene-12.38-mRN/
WRKY	AT2G38470.1	maker-VaccDscaff45-augustus-gene-55.32-mRN/
WRKY	AT2G38470.1	maker-VaccDscaff17-augustus-gene-14.29-mRN/
WRKY	AT2G38470.1	maker-VaccDscaff34-augustus-gene-280.25-mRN
WRKY	AT2G38470.1	augustus_masked-VaccDscaff47-processed-gene
WRKY	AT2G38470.1	augustus_masked-VaccDscaff47-processed-gene
C3H	AT2G40140.2	maker-VaccDscaff20-snap-gene-40.47-mRNA-1
C3H	AT2G40140.2	maker-VaccDscaff19-augustus-gene-40.36-mRN/
C3H	AT2G40140.2	maker-VaccDscaff28-augustus-gene-315.46-mRN
C3H	AT2G40140.2	maker-VaccDscaff48-snap-gene-67.40-mRNA-1
G2-like	AT2G40260.1	maker-VaccDscaff4-snap-gene-217.22-mRNA-1
ERF	AT2G40350.1	augustus_masked-VaccDscaff8-processed-gene-:
ERF	AT2G40350.1	augustus_masked-VaccDscaff5-processed-gene-:
ERF	AT2G40350.1	augustus_masked-VaccDscaff1-processed-gene-:
LBD	AT2G40470.1	maker-VaccDscaff22-snap-gene-299.44-mRNA-1
LBD	AT2G40470.1	maker-VaccDscaff43-snap-gene-78.27-mRNA-1
LBD	AT2G40470.1	maker-VaccDscaff43-snap-gene-90.31-mRNA-1
LBD	AT2G40470.1	maker-VaccDscaff20-snap-gene-35.50-mRNA-1
LBD	AT2G40470.1	maker-VaccDscaff11-augustus-gene-315.23-mRN
LBD	AT2G40470.1	maker-VaccDscaff19-augustus-gene-33.32-mRN/
LBD	AT2G40470.1	maker-VaccDscaff19-augustus-gene-371.24-mRN
LBD	AT2G40470.1	maker-VaccDscaff15-snap-gene-302.37-mRNA-1
LBD	AT2G40470.1	maker-VaccDscaff24-augustus-gene-62.35-mRN/
LBD	AT2G40470.1	maker-VaccDscaff28-augustus-gene-322.39-mRN
LBD	AT2G40470.1	maker-VaccDscaff47-snap-gene-122.46-mRNA-1
LBD	AT2G40470.1	maker-VaccDscaff48-augustus-gene-81.38-mRN/
bZIP	AT2G40620.1	maker-VaccDscaff21-augustus-gene-108.32-mRN
bZIP	AT2G40620.1	maker-VaccDscaff20-augustus-gene-32.30-mRN/
bZIP	AT2G40620.1	maker-VaccDscaff33-augustus-gene-230.31-mRN
bZIP	AT2G40620.1	maker-VaccDscaff19-augustus-gene-31.29-mRN/
bZIP	AT2G40620.1	maker-VaccDscaff29-augustus-gene-108.22-mRN

bZIP	AT2G40620.1	maker-VaccDscaff28-augustus-gene-324.51-mRN
bZIP	AT2G40620.1	maker-VaccDscaff48-augustus-gene-83.44-mRNA
WRKY	AT2G40740.2	maker-VaccDscaff21-snap-gene-105.34-mRNA-1
HSF	AT2G41690.1	maker-VaccDscaff21-augustus-gene-340.20-mRN
HSF	AT2G41690.1	maker-VaccDscaff33-augustus-gene-35.24-mRNA
HSF	AT2G41690.1	maker-VaccDscaff26-augustus-gene-332.27-mRN
HSF	AT2G41690.1	maker-VaccDscaff29-augustus-gene-319.17-mRN
AP2	AT2G41710.1	maker-VaccDscaff1268-augustus-gene-0.4-mRNA
AP2	AT2G41710.1	maker-VaccDscaff21-augustus-gene-342.29-mRN
AP2	AT2G41710.1	maker-VaccDscaff26-augustus-gene-334.27-mRN
C2H2	AT2G41835.1	maker-VaccDscaff5-augustus-gene-271.26-mRNA
C2H2	AT2G41835.1	maker-VaccDscaff1-augustus-gene-292.31-mRNA
C3H	AT2G41900.1	augustus_masked-VaccDscaff22-processed-gene
C3H	AT2G41900.1	maker-VaccDscaff22-snap-gene-84.20-mRNA-1
C3H	AT2G41900.1	augustus_masked-VaccDscaff21-processed-gene
C3H	AT2G41900.1	augustus_masked-VaccDscaff33-processed-gene
C3H	AT2G41900.1	maker-VaccDscaff1501-snap-gene-0.3-mRNA-1
C3H	AT2G41900.1	augustus_masked-VaccDscaff26-processed-gene
C3H	AT2G41900.1	maker-VaccDscaff507-snap-gene-0.11-mRNA-1
C3H	AT2G41900.1	augustus_masked-VaccDscaff29-processed-gene
SBP	AT2G42200.1	augustus_masked-VaccDscaff29-processed-gene
SBP	AT2G42200.1	augustus_masked-VaccDscaff29-processed-gene
bHLH	AT2G42280.1	augustus_masked-VaccDscaff22-processed-gene
bHLH	AT2G42280.1	maker-VaccDscaff21-augustus-gene-356.22-mRN
bHLH	AT2G42280.1	maker-VaccDscaff33-augustus-gene-18.35-mRNA
bHLH	AT2G42280.1	maker-VaccDscaff26-augustus-gene-348.18-mRN
bHLH	AT2G42280.1	maker-VaccDscaff1751-augustus-gene-0.6-mRNA
bHLH	AT2G42280.1	maker-VaccDscaff29-augustus-gene-336.19-mRN
LBD	AT2G42430.1	augustus_masked-VaccDscaff11-processed-gene
LBD	AT2G42430.1	maker-VaccDscaff46-augustus-gene-173.21-mRN
LBD	AT2G42430.1	augustus_masked-VaccDscaff15-processed-gene
LBD	AT2G42430.1	augustus_masked-VaccDscaff24-processed-gene
NAC	AT2G43000.1	augustus_masked-VaccDscaff6-processed-gene-
FAR1	AT2G43280.1	maker-VaccDscaff9-snap-gene-96.39-mRNA-1
Trihelix	AT2G44730.1	maker-VaccDscaff45-augustus-gene-160.12-mRN
Trihelix	AT2G44730.1	maker-VaccDscaff45-augustus-gene-160.12-mRN
Trihelix	AT2G44730.1	maker-VaccDscaff17-augustus-gene-193.28-mRN
Trihelix	AT2G44730.1	maker-VaccDscaff17-augustus-gene-193.28-mRN
WRKY	AT2G44745.1	maker-VaccDscaff22-snap-gene-53.54-mRNA-1
WRKY	AT2G44745.1	maker-VaccDscaff1090-augustus-gene-0.4-mRNA
ERF	AT2G44940.1	augustus_masked-VaccDscaff22-processed-gene
YABBY	AT2G45190.1	maker-VaccDscaff22-augustus-gene-41.25-mRNA
YABBY	AT2G45190.1	maker-VaccDscaff22-augustus-gene-142.21-mRN
YABBY	AT2G45190.1	maker-VaccDscaff43-snap-gene-226.27-mRNA-1
YABBY	AT2G45190.1	maker-VaccDscaff46-snap-gene-21.25-mRNA-1
LBD	AT2G45420.1	maker-VaccDscaff22-augustus-gene-36.36-mRNA

LBD	AT2G45420.1	augustus_masked-VaccDscaff842-processed-gen
LBD	AT2G45420.1	maker-VaccDscaff684-augustus-gene-0.6-mRNA-
MIKC_MADS	AT2G45650.1	maker-VaccDscaff22-snap-gene-30.36-mRNA-1
MIKC_MADS	AT2G45650.1	maker-VaccDscaff21-augustus-gene-73.27-mRN/
MIKC_MADS	AT2G45650.1	maker-VaccDscaff36-snap-gene-44.40-mRNA-1
MIKC_MADS	AT2G45650.1	maker-VaccDscaff33-augustus-gene-263.32-mRN
MIKC_MADS	AT2G45650.1	maker-VaccDscaff1265-augustus-gene-0.3-mRN/
MIKC_MADS	AT2G45650.1	maker-VaccDscaff26-augustus-gene-72.29-mRN/
MIKC_MADS	AT2G45650.1	maker-VaccDscaff29-augustus-gene-74.26-mRN/
MIKC_MADS	AT2G45660.1	maker-VaccDscaff22-augustus-gene-30.25-mRN/
MIKC_MADS	AT2G45660.1	maker-VaccDscaff22-augustus-gene-130.22-mRN
MIKC_MADS	AT2G45660.1	snap_masked-VaccDscaff12-processed-gene-281
MIKC_MADS	AT2G45660.1	maker-VaccDscaff12-augustus-gene-282.22-mRN
MIKC_MADS	AT2G45660.1	maker-VaccDscaff691-augustus-gene-0.7-mRNA-
MIKC_MADS	AT2G45660.1	snap_masked-VaccDscaff40-processed-gene-174
MIKC_MADS	AT2G45660.1	maker-VaccDscaff40-snap-gene-173.29-mRNA-1
MIKC_MADS	AT2G45660.1	maker-VaccDscaff40-snap-gene-173.30-mRNA-1
MIKC_MADS	AT2G45660.1	maker-VaccDscaff40-augustus-gene-175.28-mRN
MIKC_MADS	AT2G45660.1	maker-VaccDscaff40-snap-gene-175.33-mRNA-1
MIKC_MADS	AT2G45660.1	snap_masked-VaccDscaff41-processed-gene-162
MIKC_MADS	AT2G45660.1	maker-VaccDscaff41-augustus-gene-163.25-mRN
MIKC_MADS	AT2G45660.1	maker-VaccDscaff41-snap-gene-165.36-mRNA-1
MIKC_MADS	AT2G45660.1	maker-VaccDscaff41-snap-gene-165.37-mRNA-1
MIKC_MADS	AT2G45660.1	maker-VaccDscaff23-snap-gene-111.21-mRNA-1
MIKC_MADS	AT2G45660.1	augustus_masked-VaccDscaff23-processed-gene
TCP	AT2G45680.1	augustus_masked-VaccDscaff22-processed-gene
BES1	AT2G45880.1	augustus_masked-VaccDscaff19-processed-gene
BES1	AT2G45880.1	augustus_masked-VaccDscaff15-processed-gene
BES1	AT2G45880.1	augustus_masked-VaccDscaff24-processed-gene
BES1	AT2G45880.1	augustus_masked-VaccDscaff29-processed-gene
WRKY	AT2G46130.1	maker-VaccDscaff22-augustus-gene-14.28-mRN/
MYB_related	AT2G46410.1	maker-VaccDscaff22-snap-gene-109.19-mRNA-1
MYB_related	AT2G46410.1	maker-VaccDscaff43-augustus-gene-258.26-mRN
MYB_related	AT2G46410.1	snap_masked-VaccDscaff9-processed-gene-121.
MYB_related	AT2G46410.1	maker-VaccDscaff37-augustus-gene-29.37-mRN/
MYB_related	AT2G46410.1	snap_masked-VaccDscaff4-processed-gene-169.
MYB_related	AT2G46410.1	maker-VaccDscaff39-augustus-gene-265.22-mRN
ARF	AT2G46530.1	maker-VaccDscaff209-augustus-gene-1.37-mRN/
ARF	AT2G46530.1	maker-VaccDscaff20-augustus-gene-363.21-mRN
ARF	AT2G46530.1	maker-VaccDscaff49-snap-gene-18.32-mRNA-1
ARF	AT2G46530.3	maker-VaccDscaff20-snap-gene-363.29-mRNA-1
ARF	AT2G46530.3	maker-VaccDscaff28-snap-gene-13.24-mRNA-1
HD-ZIP	AT2G46680.1	augustus_masked-VaccDscaff1475-processed-ge
NAC	AT2G46770.1	augustus_masked-VaccDscaff175-processed-gen
NAC	AT2G46770.1	maker-VaccDscaff175-snap-gene-1.25-mRNA-1
NAC	AT2G46770.1	maker-VaccDscaff20-augustus-gene-375.23-mRN

NAC	AT2G46770.1	maker-VaccDscaff20-augustus-gene-375.23-mRN
NAC	AT2G46770.1	maker-VaccDscaff6-augustus-gene-249.26-mRN
NAC	AT2G46770.1	snap_masked-VaccDscaff49-processed-gene-5.1
NAC	AT2G46770.1	snap_masked-VaccDscaff49-processed-gene-5.1
NAC	AT2G46770.1	augustus_masked-VaccDscaff67-processed-gene
NAC	AT2G46770.1	maker-VaccDscaff67-snap-gene-6.25-mRNA-1
NAC	AT2G46770.1	maker-VaccDscaff66-snap-gene-3.24-mRNA-1
NAC	AT2G46770.1	maker-VaccDscaff66-snap-gene-4.17-mRNA-1
NAC	AT2G46770.1	maker-VaccDscaff28-augustus-gene-2.24-mRNA-
B3	AT2G46870.1	augustus_masked-VaccDscaff20-processed-gene
B3	AT2G46870.1	augustus_masked-VaccDscaff49-processed-gene
B3	AT2G46870.1	augustus_masked-VaccDscaff49-processed-gene
SBP	AT2G47070.1	maker-VaccDscaff11-augustus-gene-398.29-mRN
SBP	AT2G47070.1	maker-VaccDscaff11-augustus-gene-400.42-mRN
SBP	AT2G47070.1	maker-VaccDscaff15-augustus-gene-389.30-mRN
SBP	AT2G47070.1	maker-VaccDscaff51-augustus-gene-1.26-mRNA-
WRKY	AT2G47260.1	maker-VaccDscaff11-augustus-gene-395.29-mRN
WRKY	AT2G47260.1	maker-VaccDscaff15-snap-gene-386.36-mRNA-1
WRKY	AT2G47260.1	maker-VaccDscaff24-augustus-gene-3.26-mRNA-
WRKY	AT2G47260.1	maker-VaccDscaff51-augustus-gene-4.26-mRNA-
MYB	AT2G47460.1	maker-VaccDscaff13-snap-gene-381.52-mRNA-1
ERF	AT2G47520.1	augustus_masked-VaccDscaff22-processed-gene
ERF	AT2G47520.1	maker-VaccDscaff43-augustus-gene-7.31-mRNA-
ERF	AT2G47520.1	maker-VaccDscaff46-augustus-gene-202.30-mRN
ERF	AT2G47520.1	maker-VaccDscaff47-augustus-gene-190.29-mRN
NF-YB	AT2G47810.1	augustus_masked-VaccDscaff10-processed-gene
NF-YB	AT2G47810.1	augustus_masked-VaccDscaff8-processed-gene-
NF-YB	AT2G47810.1	augustus_masked-VaccDscaff22-processed-gene
NF-YB	AT2G47810.1	snap_masked-VaccDscaff43-processed-gene-13.
NF-YB	AT2G47810.1	snap_masked-VaccDscaff16-processed-gene-29
NF-YB	AT2G47810.1	augustus_masked-VaccDscaff11-processed-gene
NF-YB	AT2G47810.1	augustus_masked-VaccDscaff46-processed-gene
NF-YB	AT2G47810.1	augustus_masked-VaccDscaff5-processed-gene-
NF-YB	AT2G47810.1	augustus_masked-VaccDscaff15-processed-gene
NF-YB	AT2G47810.1	augustus_masked-VaccDscaff1-processed-gene-
NF-YB	AT2G47810.1	augustus_masked-VaccDscaff24-processed-gene
NF-YB	AT2G47810.1	augustus_masked-VaccDscaff51-processed-gene
NF-YB	AT2G47810.1	augustus_masked-VaccDscaff47-processed-gene
C3H	AT2G47850.3	maker-VaccDscaff10-augustus-gene-366.31-mRN
C3H	AT2G47850.3	maker-VaccDscaff8-augustus-gene-382.35-mRN
C3H	AT2G47850.3	maker-VaccDscaff118-snap-gene-3.81-mRNA-1
C3H	AT2G47850.3	maker-VaccDscaff5-augustus-gene-47.25-mRNA-
C3H	AT2G47850.3	maker-VaccDscaff15-augustus-gene-376.33-mRN
C3H	AT2G47850.3	maker-VaccDscaff9-augustus-gene-10.41-mRNA-
C3H	AT2G47850.3	maker-VaccDscaff1-augustus-gene-39.37-mRNA-
C3H	AT2G47850.3	maker-VaccDscaff24-augustus-gene-14.27-mRN

C3H	AT2G47850.3	maker-VaccDscaff138-augustus-gene-2.29-mRN/
C3H	AT2G47850.3	maker-VaccDscaff4-snap-gene-20.31-mRNA-1
C3H	AT2G47850.3	maker-VaccDscaff4-augustus-gene-35.43-mRNA-
C3H	AT2G47850.3	maker-VaccDscaff51-augustus-gene-16.29-mRN/
E2F/DP	AT3G01330.1	maker-VaccDscaff11-augustus-gene-32.28-mRN/
E2F/DP	AT3G01330.1	maker-VaccDscaff18-snap-gene-355.32-mRNA-1
HD-ZIP	AT3G01470.1	maker-VaccDscaff16-snap-gene-13.42-mRNA-1
HD-ZIP	AT3G01470.1	maker-VaccDscaff13-snap-gene-70.42-mRNA-1
HD-ZIP	AT3G01470.1	maker-VaccDscaff7-snap-gene-387.39-mRNA-1
HD-ZIP	AT3G01470.1	snap_masked-VaccDscaff18-processed-gene-36(
HD-ZIP	AT3G01470.1	maker-VaccDscaff32-snap-gene-295.59-mRNA-1
HD-ZIP	AT3G01470.1	maker-VaccDscaff30-snap-gene-336.46-mRNA-1
NAC	AT3G01600.1	maker-VaccDscaff13-augustus-gene-247.29-mRN
NAC	AT3G01600.1	snap_masked-VaccDscaff32-processed-gene-12⁄
WRKY	AT3G01970.1	maker-VaccDscaff16-augustus-gene-7.27-mRNA-
WRKY	AT3G01970.1	maker-VaccDscaff16-snap-gene-7.34-mRNA-1
WRKY	AT3G01970.1	maker-VaccDscaff16-snap-gene-7.36-mRNA-1
WRKY	AT3G01970.1	maker-VaccDscaff7-snap-gene-411.31-mRNA-1
TCP	AT3G02150.1	augustus_masked-VaccDscaff16-processed-gene
TCP	AT3G02150.1	augustus_masked-VaccDscaff13-processed-gene
TCP	AT3G02150.1	augustus_masked-VaccDscaff7-processed-gene-:
TCP	AT3G02150.1	augustus_masked-VaccDscaff18-processed-gene
TCP	AT3G02150.1	augustus_masked-VaccDscaff30-processed-gene
MIKC_MADS	AT3G02310.1	maker-VaccDscaff13-augustus-gene-88.12-mRN/
MIKC_MADS	AT3G02310.1	maker-VaccDscaff13-augustus-gene-230.13-mRN
MIKC_MADS	AT3G02310.1	maker-VaccDscaff32-augustus-gene-149.22-mRN
MIKC_MADS	AT3G02310.1	maker-VaccDscaff32-augustus-gene-276.19-mRN
MIKC_MADS	AT3G02310.1	maker-VaccDscaff30-augustus-gene-171.24-mRN
MIKC_MADS	AT3G02310.1	maker-VaccDscaff30-augustus-gene-319.18-mRN
MIKC_MADS	AT3G02310.1	maker-VaccDscaff42-snap-gene-146.24-mRNA-1
CO-like	AT3G02380.1	maker-VaccDscaff13-augustus-gene-93.32-mRN/
CO-like	AT3G02380.1	maker-VaccDscaff32-augustus-gene-272.20-mRN
LBD	AT3G02550.1	maker-VaccDscaff36-augustus-gene-108.31-mRN
LBD	AT3G02550.1	maker-VaccDscaff13-augustus-gene-98.29-mRN/
LBD	AT3G02550.1	maker-VaccDscaff9-augustus-gene-311.38-mRN/
LBD	AT3G02550.1	maker-VaccDscaff35-augustus-gene-242.35-mRN
C3H	AT3G02830.1	maker-VaccDscaff132-augustus-gene-3.28-mRN/
C3H	AT3G02830.1	maker-VaccDscaff19-augustus-gene-96.25-mRN/
C3H	AT3G02830.1	maker-VaccDscaff28-augustus-gene-261.30-mRN
C3H	AT3G02830.1	maker-VaccDscaff48-snap-gene-20.48-mRNA-1
WOX	AT3G03660.1	maker-VaccDscaff10-augustus-gene-231.21-mRN
WOX	AT3G03660.1	maker-VaccDscaff5-augustus-gene-166.15-mRN/
LBD	AT3G03760.1	maker-VaccDscaff21-augustus-gene-303.23-mRN
LBD	AT3G03760.1	maker-VaccDscaff26-augustus-gene-295.37-mRN
LBD	AT3G03760.1	maker-VaccDscaff29-augustus-gene-282.21-mRN
G2-like	AT3G04030.3	augustus_masked-VaccDscaff10-processed-gene

G2-like	AT3G04030.3	maker-VaccDscaff8-augustus-gene-139.17-mRNA
G2-like	AT3G04030.3	maker-VaccDscaff5-augustus-gene-291.29-mRNA
G2-like	AT3G04030.3	maker-VaccDscaff1-augustus-gene-312.32-mRNA
M-type_MADS	AT3G05860.2	snap_masked-VaccDscaff22-processed-gene-65.
M-type_MADS	AT3G05860.2	augustus_masked-VaccDscaff25-processed-gene
bHLH	AT3G06120.1	maker-VaccDscaff10-snap-gene-344.19-mRNA-1
bHLH	AT3G06120.1	maker-VaccDscaff8-snap-gene-355.25-mRNA-1
bHLH	AT3G06120.1	maker-VaccDscaff5-snap-gene-53.28-mRNA-1
bHLH	AT3G06120.1	maker-VaccDscaff1-snap-gene-65.32-mRNA-1
bHLH	AT3G06120.1	maker-VaccDscaff1-snap-gene-66.43-mRNA-1
C3H	AT3G06410.1	maker-VaccDscaff9-augustus-gene-10.42-mRNA-
MYB	AT3G06490.1	maker-VaccDscaff8-augustus-gene-406.20-mRNA
MYB	AT3G06490.1	maker-VaccDscaff9-augustus-gene-1.20-mRNA-1
MYB	AT3G06490.1	maker-VaccDscaff4-augustus-gene-1.22-mRNA-1
MYB	AT3G06490.1	maker-VaccDscaff4-augustus-gene-13.23-mRNA-
MYB	AT3G06490.1	maker-VaccDscaff4-augustus-gene-13.22-mRNA-
GATA	AT3G06740.1	maker-VaccDscaff10-augustus-gene-383.24-mRN
GATA	AT3G06740.1	maker-VaccDscaff8-snap-gene-394.22-mRNA-1
GATA	AT3G06740.1	maker-VaccDscaff337-snap-gene-0.36-mRNA-1
GATA	AT3G06740.1	maker-VaccDscaff9-augustus-gene-5.35-mRNA-1
GATA	AT3G06740.1	maker-VaccDscaff1-augustus-gene-21.20-mRNA-
GATA	AT3G06740.1	maker-VaccDscaff4-augustus-gene-6.19-mRNA-1
GATA	AT3G06740.1	maker-VaccDscaff4-augustus-gene-9.39-mRNA-1
GATA	AT3G06740.1	maker-VaccDscaff4-augustus-gene-30.25-mRNA-
CO-like	AT3G07650.4	maker-VaccDscaff10-augustus-gene-39.36-mRNA
CO-like	AT3G07650.4	maker-VaccDscaff8-augustus-gene-46.29-mRNA-
CO-like	AT3G07650.4	maker-VaccDscaff1236-augustus-gene-0.3-mRNA
CO-like	AT3G07650.4	maker-VaccDscaff5-augustus-gene-381.33-mRNA
CO-like	AT3G07650.4	maker-VaccDscaff1-augustus-gene-404.27-mRNA
MYB	AT3G09230.1	maker-VaccDscaff45-augustus-gene-25.28-mRNA
MYB	AT3G09230.1	augustus_masked-VaccDscaff178-processed-gen
MYB	AT3G09230.1	maker-VaccDscaff17-augustus-gene-20.19-mRNA
MYB	AT3G09230.1	maker-VaccDscaff34-augustus-gene-253.30-mRN
Trihelix	AT3G10030.1	maker-VaccDscaff21-augustus-gene-205.40-mRN
Trihelix	AT3G10030.1	maker-VaccDscaff21-snap-gene-244.29-mRNA-1
Trihelix	AT3G10030.1	maker-VaccDscaff33-snap-gene-111.42-mRNA-1
Trihelix	AT3G10030.1	maker-VaccDscaff33-augustus-gene-149.22-mRN
Trihelix	AT3G10030.1	maker-VaccDscaff26-augustus-gene-201.36-mRN
Trihelix	AT3G10030.1	maker-VaccDscaff26-snap-gene-239.46-mRNA-1
Trihelix	AT3G10030.1	augustus_masked-VaccDscaff29-processed-gene
Trihelix	AT3G10030.1	maker-VaccDscaff29-augustus-gene-231.26-mRN
Trihelix	AT3G10040.1	maker-VaccDscaff10-snap-gene-210.28-mRNA-1
Trihelix	AT3G10040.1	maker-VaccDscaff8-snap-gene-222.34-mRNA-1
Trihelix	AT3G10040.1	maker-VaccDscaff5-snap-gene-224.41-mRNA-1
Trihelix	AT3G10040.1	augustus_masked-VaccDscaff1-processed-gene-
NAC	AT3G10490.1	maker-VaccDscaff26-augustus-gene-194.26-mRN

NAC	AT3G10490.1	maker-VaccDscaff29-augustus-gene-188.19-mRN
G2-like	AT3G10760.1	augustus_masked-VaccDscaff21-processed-gene
G2-like	AT3G10760.1	augustus_masked-VaccDscaff26-processed-gene
G2-like	AT3G10760.1	augustus_masked-VaccDscaff29-processed-gene
LBD	AT3G11090.1	augustus_masked-VaccDscaff20-processed-gene
LBD	AT3G11090.1	augustus_masked-VaccDscaff19-processed-gene
LBD	AT3G11090.1	augustus_masked-VaccDscaff28-processed-gene
LBD	AT3G11090.1	snap_masked-VaccDscaff48-processed-gene-66.
WOX	AT3G11260.1	maker-VaccDscaff21-augustus-gene-130.27-mRN
WOX	AT3G11260.1	maker-VaccDscaff33-augustus-gene-206.32-mRN
WOX	AT3G11260.1	maker-VaccDscaff26-augustus-gene-133.21-mRN
WOX	AT3G11260.1	maker-VaccDscaff29-augustus-gene-130.25-mRN
MYB	AT3G11450.1	maker-VaccDscaff5-augustus-gene-366.35-mRNA
MYB	AT3G11450.1	snap_masked-VaccDscaff1-processed-gene-388.
C3H	AT3G12130.1	snap_masked-VaccDscaff703-processed-gene-0.
C3H	AT3G12130.1	maker-VaccDscaff23-snap-gene-365.40-mRNA-1
C3H	AT3G12130.1	maker-VaccDscaff23-snap-gene-365.40-mRNA-1
C3H	AT3G12680.1	augustus_masked-VaccDscaff62-processed-gene
C3H	AT3G12680.1	augustus_masked-VaccDscaff62-processed-gene
C3H	AT3G12680.1	augustus_masked-VaccDscaff62-processed-gene
C3H	AT3G12680.1	maker-VaccDscaff317-snap-gene-0.48-mRNA-1
C3H	AT3G12680.1	maker-VaccDscaff317-snap-gene-0.48-mRNA-1
C3H	AT3G12680.1	maker-VaccDscaff317-snap-gene-0.48-mRNA-1
C3H	AT3G12680.1	augustus_masked-VaccDscaff11-processed-gene
C3H	AT3G12680.1	snap_masked-VaccDscaff6-processed-gene-404.
C3H	AT3G12680.1	augustus_masked-VaccDscaff166-processed-gen
C3H	AT3G12680.1	augustus_masked-VaccDscaff166-processed-gen
C3H	AT3G12680.1	augustus_masked-VaccDscaff166-processed-gen
C3H	AT3G12680.1	augustus_masked-VaccDscaff78-processed-gene
C3H	AT3G12680.1	augustus_masked-VaccDscaff78-processed-gene
C3H	AT3G12680.1	augustus_masked-VaccDscaff78-processed-gene
MYB	AT3G12720.1	maker-VaccDscaff11-augustus-gene-223.26-mRN
MYB	AT3G12720.1	maker-VaccDscaff15-augustus-gene-214.26-mRN
MYB	AT3G12720.1	maker-VaccDscaff24-augustus-gene-169.31-mRN
MYB	AT3G12720.1	maker-VaccDscaff82-augustus-gene-6.35-mRNA-
MYB	AT3G13540.1	maker-VaccDscaff22-augustus-gene-3.22-mRNA-
MYB	AT3G13540.1	maker-VaccDscaff38-snap-gene-163.31-mRNA-1
MYB	AT3G13540.1	maker-VaccDscaff11-augustus-gene-9.17-mRNA-
MYB	AT3G13540.1	maker-VaccDscaff6-augustus-gene-237.10-mRNA
MYB	AT3G13540.1	maker-VaccDscaff6-snap-gene-261.32-mRNA-1
MYB	AT3G13540.1	maker-VaccDscaff15-snap-gene-21.24-mRNA-1
MYB	AT3G13540.1	maker-VaccDscaff37-augustus-gene-140.14-mRN
MYB	AT3G13540.1	maker-VaccDscaff37-snap-gene-143.27-mRNA-1
MYB	AT3G13540.1	maker-VaccDscaff44-snap-gene-239.19-mRNA-1
MYB	AT3G13540.1	maker-VaccDscaff92-augustus-gene-4.28-mRNA-
MYB	AT3G13540.1	maker-VaccDscaff39-snap-gene-168.25-mRNA-1

MYB	AT3G13540.1	maker-VaccDscaff39-augustus-gene-189.28-mRN
GRAS	AT3G13840.1	augustus_masked-VaccDscaff17-processed-gene
LBD	AT3G13850.1	snap_masked-VaccDscaff22-processed-gene-202
LBD	AT3G13850.1	maker-VaccDscaff36-augustus-gene-12.30-mRNA
LBD	AT3G13850.1	augustus_masked-VaccDscaff182-processed-gen
LBD	AT3G13850.1	maker-VaccDscaff9-augustus-gene-402.30-mRNA
LBD	AT3G13850.1	maker-VaccDscaff35-augustus-gene-312.40-mRN
LBD	AT3G13850.1	augustus_masked-VaccDscaff34-processed-gene
LBD	AT3G13850.1	maker-VaccDscaff4-augustus-gene-415.29-mRNA
LBD	AT3G13850.1	augustus_masked-VaccDscaff47-processed-gene
MYB	AT3G13890.1	snap_masked-VaccDscaff39-processed-gene-111
MYB	AT3G13890.2	maker-VaccDscaff27-augustus-gene-348.13-mRN
MYB	AT3G13890.2	maker-VaccDscaff27-augustus-gene-348.14-mRN
MYB	AT3G13890.2	maker-VaccDscaff27-snap-gene-349.8-mRNA-1
MYB	AT3G13890.2	maker-VaccDscaff27-snap-gene-350.30-mRNA-1
MYB	AT3G13890.2	maker-VaccDscaff27-snap-gene-350.29-mRNA-1
MYB	AT3G13890.2	maker-VaccDscaff36-augustus-gene-11.20-mRNA
MYB	AT3G13890.2	maker-VaccDscaff36-augustus-gene-11.19-mRNA
MYB	AT3G13890.2	maker-VaccDscaff9-augustus-gene-400.23-mRNA
MYB	AT3G13890.2	maker-VaccDscaff9-augustus-gene-400.24-mRNA
MYB	AT3G13890.2	maker-VaccDscaff35-augustus-gene-313.33-mRN
MYB	AT3G13890.2	maker-VaccDscaff35-snap-gene-313.41-mRNA-1
MYB	AT3G13890.2	maker-VaccDscaff34-augustus-gene-15.17-mRNA
MYB	AT3G13890.2	augustus_masked-VaccDscaff34-processed-gene
MYB	AT3G13890.2	maker-VaccDscaff34-augustus-gene-16.17-mRNA
MYB	AT3G13890.2	maker-VaccDscaff34-snap-gene-17.23-mRNA-1
MYB	AT3G13890.2	maker-VaccDscaff4-augustus-gene-413.24-mRNA
MYB	AT3G13890.2	maker-VaccDscaff4-augustus-gene-414.22-mRNA
GRF	AT3G13960.1	maker-VaccDscaff22-snap-gene-192.24-mRNA-1
GRF	AT3G13960.1	maker-VaccDscaff43-snap-gene-168.35-mRNA-1
GRF	AT3G13960.1	maker-VaccDscaff21-augustus-gene-13.30-mRNA
GRF	AT3G13960.1	maker-VaccDscaff13-augustus-gene-381.41-mRN
GRF	AT3G13960.1	snap_masked-VaccDscaff13-processed-gene-381
GRF	AT3G13960.1	maker-VaccDscaff33-augustus-gene-307.20-mRN
GRF	AT3G13960.1	maker-VaccDscaff302-augustus-gene-0.41-mRNA
GRF	AT3G13960.1	maker-VaccDscaff46-snap-gene-79.28-mRNA-1
GRF	AT3G13960.1	maker-VaccDscaff30-augustus-gene-17.40-mRNA
GRF	AT3G13960.1	maker-VaccDscaff29-augustus-gene-9.39-mRNA-
GRF	AT3G13960.1	maker-VaccDscaff47-snap-gene-11.26-mRNA-1
ERF	AT3G15210.1	augustus_masked-VaccDscaff10-processed-gene
ERF	AT3G15210.1	augustus_masked-VaccDscaff8-processed-gene-
ERF	AT3G15210.1	augustus_masked-VaccDscaff696-processed-gen
ERF	AT3G15210.1	augustus_masked-VaccDscaff5-processed-gene-
ERF	AT3G15210.1	augustus_masked-VaccDscaff1-processed-gene-
SBP	AT3G15270.1	maker-VaccDscaff883-augustus-gene-0.6-mRNA-
NAC	AT3G15510.1	maker-VaccDscaff8-snap-gene-30.29-mRNA-1

NAC	AT3G15510.1	maker-VaccDscaff6-augustus-gene-64.35-mRNA-
NAC	AT3G15510.1	maker-VaccDscaff5-augustus-gene-399.21-mRNA
NAC	AT3G15510.1	maker-VaccDscaff1-augustus-gene-421.18-mRNA
NAC	AT3G15510.1	maker-VaccDscaff665-augustus-gene-0.12-mRNA
MYB_related	AT3G16350.1	maker-VaccDscaff27-augustus-gene-321.25-mRN
MYB_related	AT3G16350.1	maker-VaccDscaff17-augustus-gene-357.20-mRN
MYB_related	AT3G16350.1	maker-VaccDscaff34-augustus-gene-24.37-mRNA
NAC	AT3G17730.1	augustus_masked-VaccDscaff9-processed-gene-
NAC	AT3G17730.1	maker-VaccDscaff35-augustus-gene-97.21-mRNA
NAC	AT3G17730.1	maker-VaccDscaff4-augustus-gene-188.17-mRNA
NAC	AT3G17730.1	maker-VaccDscaff4-augustus-gene-191.13-mRNA
MYB	AT3G18100.2	maker-VaccDscaff30-augustus-gene-291.34-mRN
HB-other	AT3G18380.1	snap_masked-VaccDscaff10-processed-gene-27.
HB-other	AT3G18380.1	maker-VaccDscaff8-augustus-gene-36.39-mRNA-
HB-other	AT3G18380.1	maker-VaccDscaff1388-snap-gene-0.6-mRNA-1
HB-other	AT3G18380.1	maker-VaccDscaff5-augustus-gene-394.43-mRNA
HB-other	AT3G18380.1	maker-VaccDscaff1-augustus-gene-415.37-mRNA
HB-other	AT3G18380.1	maker-VaccDscaff1438-snap-gene-0.6-mRNA-1
NAC	AT3G18400.1	augustus_masked-VaccDscaff36-processed-gene
NAC	AT3G18400.1	maker-VaccDscaff9-augustus-gene-311.41-mRNA
NAC	AT3G18400.1	snap_masked-VaccDscaff35-processed-gene-242
NAC	AT3G18400.1	maker-VaccDscaff4-augustus-gene-334.27-mRNA
B3	AT3G18990.1	maker-VaccDscaff13-augustus-gene-141.29-mRN
B3	AT3G19184.1	maker-VaccDscaff27-augustus-gene-223.46-mRN
B3	AT3G19184.1	maker-VaccDscaff45-snap-gene-218.38-mRNA-1
B3	AT3G19184.1	maker-VaccDscaff45-snap-gene-218.38-mRNA-1
B3	AT3G19184.1	maker-VaccDscaff17-augustus-gene-239.31-mRN
B3	AT3G19184.1	maker-VaccDscaff3-snap-gene-9.46-mRNA-1
B3	AT3G19184.1	maker-VaccDscaff3-snap-gene-9.46-mRNA-1
bHLH	AT3G19860.1	maker-VaccDscaff12-augustus-gene-391.21-mRN
bHLH	AT3G19860.1	snap_masked-VaccDscaff13-processed-gene-296
bHLH	AT3G19860.1	maker-VaccDscaff53-augustus-gene-5.31-mRNA-
bHLH	AT3G19860.1	snap_masked-VaccDscaff32-processed-gene-105
bHLH	AT3G19860.1	maker-VaccDscaff30-snap-gene-98.38-mRNA-1
bHLH	AT3G19860.1	maker-VaccDscaff30-snap-gene-102.30-mRNA-1
bHLH	AT3G19860.1	snap_masked-VaccDscaff23-processed-gene-5.1
ERF	AT3G20310.1	snap_masked-VaccDscaff6-processed-gene-90.2
EIL	AT3G20770.1	augustus_masked-VaccDscaff43-processed-gene
EIL	AT3G20770.1	augustus_masked-VaccDscaff536-processed-gen
EIL	AT3G20770.1	augustus_masked-VaccDscaff47-processed-gene
GATA	AT3G21175.2	maker-VaccDscaff27-snap-gene-77.25-mRNA-1
GATA	AT3G21175.2	maker-VaccDscaff20-snap-gene-143.35-mRNA-1
GATA	AT3G21175.2	maker-VaccDscaff16-augustus-gene-343.13-mRN
GATA	AT3G21175.2	maker-VaccDscaff45-snap-gene-73.34-mRNA-1
GATA	AT3G21175.2	maker-VaccDscaff7-augustus-gene-35.32-mRNA-
GATA	AT3G21175.2	maker-VaccDscaff7-augustus-gene-64.18-mRNA-

GATA	AT3G21175.2	maker-VaccDscaff31-augustus-gene-291.23-mRN
GATA	AT3G21175.2	maker-VaccDscaff2-snap-gene-384.33-mRNA-1
GATA	AT3G21175.2	augustus_masked-VaccDscaff2-processed-gene-
GATA	AT3G21175.2	maker-VaccDscaff17-snap-gene-114.50-mRNA-1
GATA	AT3G21175.2	maker-VaccDscaff14-augustus-gene-331.39-mRN
GATA	AT3G21175.2	maker-VaccDscaff34-snap-gene-238.34-mRNA-1
GATA	AT3G21175.2	maker-VaccDscaff23-snap-gene-278.20-mRNA-1
GATA	AT3G21175.2	maker-VaccDscaff3-augustus-gene-27.25-mRNA-
GATA	AT3G21175.2	maker-VaccDscaff3-snap-gene-386.44-mRNA-1
GATA	AT3G21175.2	maker-VaccDscaff234-snap-gene-0.51-mRNA-1
bHLH	AT3G21330.1	augustus_masked-VaccDscaff17-processed-gene
bHLH	AT3G22100.1	augustus_masked-VaccDscaff10-processed-gene
bHLH	AT3G22100.1	augustus_masked-VaccDscaff8-processed-gene-
bHLH	AT3G22100.1	augustus_masked-VaccDscaff5-processed-gene-
bHLH	AT3G22100.1	augustus_masked-VaccDscaff1-processed-gene-
HSF	AT3G22830.1	maker-VaccDscaff10-snap-gene-327.42-mRNA-1
HSF	AT3G22830.1	maker-VaccDscaff22-augustus-gene-344.19-mRN
HSF	AT3G22830.1	maker-VaccDscaff43-snap-gene-28.26-mRNA-1
HSF	AT3G22830.1	maker-VaccDscaff46-augustus-gene-180.27-mRN
HSF	AT3G22830.1	maker-VaccDscaff5-augustus-gene-69.29-mRNA-
HSF	AT3G22830.1	snap_masked-VaccDscaff1-processed-gene-84.1
HSF	AT3G22830.1	maker-VaccDscaff47-snap-gene-171.22-mRNA-1
ERF	AT3G23230.1	augustus_masked-VaccDscaff10-processed-gene
ERF	AT3G23230.1	augustus_masked-VaccDscaff8-processed-gene-
ERF	AT3G23230.1	maker-VaccDscaff22-snap-gene-333.33-mRNA-1
ERF	AT3G23230.1	maker-VaccDscaff22-snap-gene-333.33-mRNA-1
ERF	AT3G23230.1	snap_masked-VaccDscaff43-processed-gene-39.
ERF	AT3G23230.1	augustus_masked-VaccDscaff43-processed-gene
ERF	AT3G23230.1	augustus_masked-VaccDscaff36-processed-gene
ERF	AT3G23230.1	augustus_masked-VaccDscaff36-processed-gene
ERF	AT3G23230.1	augustus_masked-VaccDscaff11-processed-gene
ERF	AT3G23230.1	augustus_masked-VaccDscaff11-processed-gene
ERF	AT3G23230.1	augustus_masked-VaccDscaff46-processed-gene
ERF	AT3G23230.1	augustus_masked-VaccDscaff46-processed-gene
ERF	AT3G23230.1	augustus_masked-VaccDscaff5-processed-gene-
ERF	AT3G23230.1	augustus_masked-VaccDscaff15-processed-gene
ERF	AT3G23230.1	augustus_masked-VaccDscaff15-processed-gene
ERF	AT3G23230.1	augustus_masked-VaccDscaff1-processed-gene-
ERF	AT3G23230.1	augustus_masked-VaccDscaff35-processed-gene
ERF	AT3G23230.1	augustus_masked-VaccDscaff24-processed-gene
ERF	AT3G23230.1	maker-VaccDscaff24-snap-gene-34.50-mRNA-1
ERF	AT3G23230.1	augustus_masked-VaccDscaff4-processed-gene-
ERF	AT3G23230.1	maker-VaccDscaff47-snap-gene-160.37-mRNA-1
ERF	AT3G23230.1	maker-VaccDscaff47-snap-gene-160.37-mRNA-1
ERF	AT3G23240.1	maker-VaccDscaff10-snap-gene-292.18-mRNA-1
ERF	AT3G23240.1	maker-VaccDscaff10-snap-gene-292.18-mRNA-1

ERF	AT3G23240.1	augustus_masked-VaccDscaff8-processed-gene-:
ERF	AT3G23240.1	augustus_masked-VaccDscaff8-processed-gene-:
ERF	AT3G23240.1	augustus_masked-VaccDscaff22-processed-gene
ERF	AT3G23240.1	augustus_masked-VaccDscaff43-processed-gene
ERF	AT3G23240.1	augustus_masked-VaccDscaff20-processed-gene
ERF	AT3G23240.1	snap_masked-VaccDscaff11-processed-gene-368
ERF	AT3G23240.1	augustus_masked-VaccDscaff46-processed-gene
ERF	AT3G23240.1	maker-VaccDscaff5-snap-gene-77.26-mRNA-1
ERF	AT3G23240.1	maker-VaccDscaff5-snap-gene-77.26-mRNA-1
ERF	AT3G23240.1	snap_masked-VaccDscaff40-processed-gene-255
ERF	AT3G23240.1	augustus_masked-VaccDscaff19-processed-gene
ERF	AT3G23240.1	augustus_masked-VaccDscaff15-processed-gene
ERF	AT3G23240.1	maker-VaccDscaff1-snap-gene-111.21-mRNA-1
ERF	AT3G23240.1	maker-VaccDscaff1-snap-gene-111.21-mRNA-1
ERF	AT3G23240.1	maker-VaccDscaff24-augustus-gene-34.39-mRNA
ERF	AT3G23240.1	snap_masked-VaccDscaff23-processed-gene-39.
ERF	AT3G23240.1	augustus_masked-VaccDscaff28-processed-gene
ERF	AT3G23240.1	augustus_masked-VaccDscaff47-processed-gene
ERF	AT3G23240.1	augustus_masked-VaccDscaff48-processed-gene
MYB	AT3G23250.1	maker-VaccDscaff8-augustus-gene-300.20-mRNA
MYB	AT3G23250.1	maker-VaccDscaff20-augustus-gene-92.16-mRNA
MYB	AT3G23250.1	maker-VaccDscaff6-augustus-gene-115.13-mRNA
MYB	AT3G23250.1	maker-VaccDscaff19-augustus-gene-89.19-mRNA
MYB	AT3G23250.1	maker-VaccDscaff1-augustus-gene-115.28-mRNA
MYB	AT3G23250.1	maker-VaccDscaff28-snap-gene-269.38-mRNA-1
MYB	AT3G23250.1	maker-VaccDscaff48-augustus-gene-27.25-mRNA
G2-like	AT3G24120.1	maker-VaccDscaff36-snap-gene-237.31-mRNA-1
G2-like	AT3G24120.1	maker-VaccDscaff14-augustus-gene-37.37-mRNA
G2-like	AT3G24120.1	maker-VaccDscaff9-snap-gene-67.39-mRNA-1
G2-like	AT3G24120.1	maker-VaccDscaff4-augustus-gene-109.27-mRNA
G2-like	AT3G24120.2	snap_masked-VaccDscaff10-processed-gene-40.
bHLH	AT3G24140.1	maker-VaccDscaff2-augustus-gene-73.20-mRNA-
MYB	AT3G24310.1	maker-VaccDscaff2-augustus-gene-90.32-mRNA-
Trihelix	AT3G24490.1	augustus_masked-VaccDscaff12-processed-gene
Trihelix	AT3G24490.1	augustus_masked-VaccDscaff41-processed-gene
HSF	AT3G24520.1	maker-VaccDscaff40-augustus-gene-229.22-mRN
HSF	AT3G24520.1	maker-VaccDscaff41-augustus-gene-213.33-mRN
B3	AT3G24650.1	maker-VaccDscaff45-snap-gene-172.41-mRNA-1
Trihelix	AT3G25990.1	maker-VaccDscaff13-augustus-gene-58.37-mRNA
Trihelix	AT3G25990.1	maker-VaccDscaff32-augustus-gene-306.26-mRN
Trihelix	AT3G25990.1	maker-VaccDscaff42-augustus-gene-5.27-mRNA-
LBD	AT3G26620.1	maker-VaccDscaff19-snap-gene-148.30-mRNA-1
LBD	AT3G26660.1	maker-VaccDscaff38-snap-gene-210.28-mRNA-1
LBD	AT3G26660.1	maker-VaccDscaff15-snap-gene-31.32-mRNA-1
LBD	AT3G26660.1	maker-VaccDscaff15-snap-gene-54.23-mRNA-1
LBD	AT3G26660.1	maker-VaccDscaff37-augustus-gene-69.35-mRNA

LBD	AT3G26660.1	maker-VaccDscaff44-snap-gene-228.39-mRNA-1
LBD	AT3G26660.1	maker-VaccDscaff24-snap-gene-322.24-mRNA-1
LBD	AT3G26660.1	maker-VaccDscaff39-snap-gene-237.29-mRNA-1
bHLH	AT3G26744.4	snap_masked-VaccDscaff13-processed-gene-48.
bHLH	AT3G26744.4	augustus_masked-VaccDscaff50-processed-gene
bHLH	AT3G26744.4	maker-VaccDscaff32-augustus-gene-316.27-mRN
bHLH	AT3G26744.4	maker-VaccDscaff30-augustus-gene-304.40-mRN
bHLH	AT3G26744.4	maker-VaccDscaff42-augustus-gene-12.30-mRN
LBD	AT3G27650.1	augustus_masked-VaccDscaff13-processed-gene
LBD	AT3G27650.1	maker-VaccDscaff32-snap-gene-301.41-mRNA-1
LBD	AT3G27650.1	maker-VaccDscaff30-snap-gene-343.78-mRNA-1
MYB	AT3G27810.1	maker-VaccDscaff16-augustus-gene-16.37-mRN
MYB	AT3G27810.1	maker-VaccDscaff16-augustus-gene-33.28-mRN
MYB	AT3G27810.1	maker-VaccDscaff13-augustus-gene-67.33-mRN
MYB	AT3G27810.1	maker-VaccDscaff18-augustus-gene-364.34-mRN
MYB	AT3G27810.1	maker-VaccDscaff32-augustus-gene-298.24-mRN
MYB	AT3G27810.1	maker-VaccDscaff30-augustus-gene-338.25-mRN
bHLH	AT3G28857.1	maker-VaccDscaff36-snap-gene-81.41-mRNA-1
bHLH	AT3G28857.1	maker-VaccDscaff13-augustus-gene-77.41-mRN
bHLH	AT3G28857.1	maker-VaccDscaff9-augustus-gene-336.36-mRN
bHLH	AT3G28857.1	maker-VaccDscaff32-augustus-gene-287.19-mRN
bHLH	AT3G28857.1	maker-VaccDscaff35-augustus-gene-265.35-mRN
bHLH	AT3G28857.1	maker-VaccDscaff30-augustus-gene-327.30-mRN
bHLH	AT3G28857.1	maker-VaccDscaff4-augustus-gene-359.33-mRN
ZF-HD	AT3G28917.1	augustus_masked-VaccDscaff21-processed-gene
ZF-HD	AT3G28917.1	snap_masked-VaccDscaff36-processed-gene-83.
ZF-HD	AT3G28917.1	snap_masked-VaccDscaff16-processed-gene-62.
ZF-HD	AT3G28917.1	augustus_masked-VaccDscaff13-processed-gene
ZF-HD	AT3G28917.1	augustus_masked-VaccDscaff7-processed-gene-
ZF-HD	AT3G28917.1	augustus_masked-VaccDscaff26-processed-gene
ZF-HD	AT3G28917.1	snap_masked-VaccDscaff18-processed-gene-33
ZF-HD	AT3G28917.1	snap_masked-VaccDscaff9-processed-gene-334.
ZF-HD	AT3G28917.1	augustus_masked-VaccDscaff32-processed-gene
ZF-HD	AT3G28917.1	snap_masked-VaccDscaff35-processed-gene-26
ZF-HD	AT3G28917.1	augustus_masked-VaccDscaff30-processed-gene
ZF-HD	AT3G28917.1	augustus_masked-VaccDscaff4-processed-gene-
ZF-HD	AT3G28917.1	augustus_masked-VaccDscaff29-processed-gene
MYB	AT3G30210.1	maker-VaccDscaff16-snap-gene-84.41-mRNA-1
MYB	AT3G30210.1	maker-VaccDscaff7-augustus-gene-342.27-mRN
MYB	AT3G30210.1	snap_masked-VaccDscaff18-processed-gene-31
bZIP	AT3G30530.1	augustus_masked-VaccDscaff20-processed-gene
bZIP	AT3G30530.1	augustus_masked-VaccDscaff16-processed-gene
bZIP	AT3G30530.1	augustus_masked-VaccDscaff7-processed-gene-
bZIP	AT3G30530.1	augustus_masked-VaccDscaff31-processed-gene
bZIP	AT3G30530.1	augustus_masked-VaccDscaff18-processed-gene
bZIP	AT3G30530.1	augustus_masked-VaccDscaff19-processed-gene

bZIP	AT3G30530.1	augustus_masked-VaccDscaff28-processed-gene
bZIP	AT3G30530.1	augustus_masked-VaccDscaff48-processed-gene
C2H2	AT3G44750.2	maker-VaccDscaff21-snap-gene-247.39-mRNA-1
C2H2	AT3G44750.2	maker-VaccDscaff33-augustus-gene-108.21-mRN
MYB	AT3G46130.1	maker-VaccDscaff22-augustus-gene-273.29-mRN
MYB	AT3G46130.1	maker-VaccDscaff43-augustus-gene-115.32-mRN
MYB	AT3G46130.1	maker-VaccDscaff43-augustus-gene-114.39-mRN
MYB	AT3G46130.1	maker-VaccDscaff11-augustus-gene-326.34-mRN
MYB	AT3G46130.1	maker-VaccDscaff19-augustus-gene-345.33-mRN
MYB	AT3G46130.1	maker-VaccDscaff15-augustus-gene-317.35-mRN
MYB	AT3G46130.1	maker-VaccDscaff24-augustus-gene-87.27-mRNA
MYB	AT3G46130.1	maker-VaccDscaff47-augustus-gene-90.26-mRNA
GRAS	AT3G46600.2	maker-VaccDscaff50-snap-gene-8.32-mRNA-1
GRAS	AT3G46600.2	maker-VaccDscaff37-augustus-gene-266.27-mRN
GRAS	AT3G46600.2	maker-VaccDscaff844-augustus-gene-0.9-mRNA-
G2-like	AT3G46640.2	augustus_masked-VaccDscaff22-processed-gene
G2-like	AT3G46640.2	augustus_masked-VaccDscaff43-processed-gene
G2-like	AT3G46640.2	maker-VaccDscaff46-snap-gene-136.49-mRNA-1
G2-like	AT3G46640.2	maker-VaccDscaff24-augustus-gene-78.36-mRNA
G2-like	AT3G46640.2	maker-VaccDscaff47-augustus-gene-100.19-mRN
MYB	AT3G47600.1	maker-VaccDscaff25-snap-gene-247.28-mRNA-1
MYB	AT3G47600.1	maker-VaccDscaff16-augustus-gene-61.39-mRNA
MYB	AT3G47600.1	maker-VaccDscaff64-augustus-gene-3.30-mRNA-
MYB	AT3G47600.1	maker-VaccDscaff64-augustus-gene-4.30-mRNA-
MYB	AT3G47600.1	maker-VaccDscaff7-augustus-gene-363.26-mRNA
MYB	AT3G47600.1	maker-VaccDscaff18-augustus-gene-336.27-mRN
bHLH	AT3G47640.3	maker-VaccDscaff30-snap-gene-143.31-mRNA-1
LBD	AT3G47870.1	maker-VaccDscaff16-augustus-gene-47.39-mRNA
LBD	AT3G47870.1	maker-VaccDscaff16-augustus-gene-48.42-mRNA
LBD	AT3G47870.1	maker-VaccDscaff7-augustus-gene-376.31-mRNA
LBD	AT3G47870.1	maker-VaccDscaff7-snap-gene-376.45-mRNA-1
LBD	AT3G47870.1	maker-VaccDscaff18-snap-gene-351.45-mRNA-1
E2F/DP	AT3G48160.2	maker-VaccDscaff27-augustus-gene-242.25-mRN
E2F/DP	AT3G48160.2	maker-VaccDscaff17-augustus-gene-265.26-mRN
E2F/DP	AT3G48160.2	maker-VaccDscaff34-augustus-gene-111.28-mRN
C2H2	AT3G48430.1	maker-VaccDscaff36-augustus-gene-51.36-mRNA
C2H2	AT3G48430.1	maker-VaccDscaff36-augustus-gene-51.36-mRNA
C2H2	AT3G48430.1	maker-VaccDscaff9-snap-gene-358.40-mRNA-1
C2H2	AT3G48430.1	maker-VaccDscaff9-snap-gene-358.40-mRNA-1
C2H2	AT3G48430.1	maker-VaccDscaff35-augustus-gene-275.27-mRN
C2H2	AT3G48430.1	maker-VaccDscaff35-augustus-gene-275.27-mRN
C2H2	AT3G48430.1	maker-VaccDscaff4-augustus-gene-378.32-mRNA
C2H2	AT3G48430.1	maker-VaccDscaff4-augustus-gene-378.32-mRNA
C3H	AT3G48440.1	maker-VaccDscaff27-snap-gene-262.22-mRNA-1
C3H	AT3G48440.1	maker-VaccDscaff36-augustus-gene-51.35-mRNA
C3H	AT3G48440.1	maker-VaccDscaff33-augustus-gene-105.20-mRN

C3H	AT3G48440.1	snap_masked-VaccDscaff33-processed-gene-10[illegible]
C3H	AT3G48440.1	maker-VaccDscaff17-snap-gene-295.25-mRNA-1
C3H	AT3G48440.1	maker-VaccDscaff9-snap-gene-358.39-mRNA-1
C3H	AT3G48440.1	snap_masked-VaccDscaff34-processed-gene-84.[illegible]
NF-YC	AT3G48590.1	snap_masked-VaccDscaff27-processed-gene-27[illegible]
NF-YC	AT3G48590.1	maker-VaccDscaff36-augustus-gene-45.33-mRN[illegible]
NF-YC	AT3G48590.1	snap_masked-VaccDscaff17-processed-gene-30[illegible]
NF-YC	AT3G48590.1	snap_masked-VaccDscaff9-processed-gene-364.[illegible]
NF-YC	AT3G48590.1	snap_masked-VaccDscaff50-processed-gene-4.1[illegible]
NF-YC	AT3G48590.1	snap_masked-VaccDscaff35-processed-gene-28[illegible]
NF-YC	AT3G48590.1	maker-VaccDscaff34-augustus-gene-73.38-mRN[illegible]
NF-YC	AT3G48590.1	snap_masked-VaccDscaff4-processed-gene-384.[illegible]
MYB	AT3G49690.1	maker-VaccDscaff13-augustus-gene-110.26-mR[illegible]
MYB	AT3G49690.1	maker-VaccDscaff40-snap-gene-49.36-mRNA-1
MYB	AT3G49690.1	maker-VaccDscaff32-augustus-gene-265.16-mR[illegible]
LBD	AT3G49940.1	maker-VaccDscaff27-augustus-gene-208.18-mR[illegible]
LBD	AT3G49940.1	augustus_masked-VaccDscaff12-processed-gene[illegible]
LBD	AT3G49940.1	maker-VaccDscaff12-augustus-gene-111.25-mR[illegible]
LBD	AT3G49940.1	maker-VaccDscaff45-augustus-gene-209.29-mR[illegible]
LBD	AT3G49940.1	maker-VaccDscaff40-snap-gene-39.47-mRNA-1
LBD	AT3G49940.1	maker-VaccDscaff40-augustus-gene-39.37-mRN[illegible]
LBD	AT3G49940.1	maker-VaccDscaff41-augustus-gene-29.36-mRN[illegible]
LBD	AT3G49940.1	maker-VaccDscaff41-augustus-gene-29.37-mRN[illegible]
LBD	AT3G49940.1	maker-VaccDscaff32-augustus-gene-258.34-mR[illegible]
LBD	AT3G49940.1	maker-VaccDscaff30-augustus-gene-254.23-mR[illegible]
LBD	AT3G49940.1	maker-VaccDscaff34-augustus-gene-147.23-mR[illegible]
LBD	AT3G49940.1	maker-VaccDscaff23-augustus-gene-301.30-mR[illegible]
GRAS	AT3G49950.1	maker-VaccDscaff16-snap-gene-146.22-mRNA-1
GRAS	AT3G49950.1	maker-VaccDscaff7-snap-gene-285.34-mRNA-1
GRAS	AT3G49950.1	maker-VaccDscaff31-snap-gene-60.19-mRNA-1
GRAS	AT3G49950.1	maker-VaccDscaff18-snap-gene-256.18-mRNA-1
MYB	AT3G50060.1	augustus_masked-VaccDscaff21-processed-gene[illegible]
MYB	AT3G50060.1	augustus_masked-VaccDscaff33-processed-gene[illegible]
MYB	AT3G50060.1	augustus_masked-VaccDscaff26-processed-gene[illegible]
MYB	AT3G50060.1	snap_masked-VaccDscaff29-processed-gene-12[illegible]
ERF	AT3G50260.1	augustus_masked-VaccDscaff7-processed-gene-[illegible]
ERF	AT3G50260.1	augustus_masked-VaccDscaff31-processed-gene[illegible]
ERF	AT3G50260.1	augustus_masked-VaccDscaff18-processed-gene[illegible]
ERF	AT3G50260.1	augustus_masked-VaccDscaff222-processed-gen[illegible]
bHLH	AT3G50330.1	augustus_masked-VaccDscaff16-processed-gene[illegible]
C2H2	AT3G50700.1	maker-VaccDscaff13-augustus-gene-154.17-mR[illegible]
C2H2	AT3G50700.1	maker-VaccDscaff11-augustus-gene-385.23-mR[illegible]
C2H2	AT3G50700.1	maker-VaccDscaff15-augustus-gene-377.29-mR[illegible]
C2H2	AT3G50700.1	maker-VaccDscaff32-augustus-gene-210.36-mR[illegible]
C2H2	AT3G50700.1	maker-VaccDscaff30-augustus-gene-218.22-mR[illegible]
C2H2	AT3G50700.1	maker-VaccDscaff24-augustus-gene-13.23-mRN[illegible]

C2H2	AT3G50700.1	maker-VaccDscaff42-augustus-gene-85.21-mRN/
BES1	AT3G50750.1	augustus_masked-VaccDscaff27-processed-gene
BES1	AT3G50750.1	augustus_masked-VaccDscaff45-processed-gene
BES1	AT3G50750.1	augustus_masked-VaccDscaff17-processed-gene
BES1	AT3G50750.1	augustus_masked-VaccDscaff34-processed-gene
C3H	AT3G51120.1	maker-VaccDscaff13-snap-gene-43.56-mRNA-1
C3H	AT3G51120.1	maker-VaccDscaff13-snap-gene-43.56-mRNA-1
C3H	AT3G51120.1	maker-VaccDscaff123-snap-gene-2.38-mRNA-1
Dof	AT3G52440.1	augustus_masked-VaccDscaff21-processed-gene
Dof	AT3G52440.1	augustus_masked-VaccDscaff33-processed-gene
Dof	AT3G52440.1	augustus_masked-VaccDscaff26-processed-gene
Dof	AT3G52440.1	augustus_masked-VaccDscaff24-processed-gene
Dof	AT3G52440.1	augustus_masked-VaccDscaff29-processed-gene
GRF	AT3G52910.1	maker-VaccDscaff21-augustus-gene-252.25-mRN
GRF	AT3G52910.1	maker-VaccDscaff33-augustus-gene-49.24-mRN/
GRF	AT3G52910.1	maker-VaccDscaff26-augustus-gene-244.23-mRN
GRF	AT3G52910.1	maker-VaccDscaff29-augustus-gene-238.19-mRN
MYB	AT3G53200.1	maker-VaccDscaff33-augustus-gene-120.22-mRN
GRAS	AT3G54220.1	maker-VaccDscaff10-augustus-gene-175.17-mRN
GRAS	AT3G54220.1	maker-VaccDscaff8-augustus-gene-188.34-mRN/
GRAS	AT3G54220.1	maker-VaccDscaff5-snap-gene-174.30-mRNA-1
GRAS	AT3G54220.1	maker-VaccDscaff5-snap-gene-174.30-mRNA-1
GRAS	AT3G54220.1	maker-VaccDscaff1-snap-gene-258.37-mRNA-1
AP2	AT3G54320.3	maker-VaccDscaff1413-snap-gene-0.8-mRNA-1
MIKC_MADS	AT3G54340.1	maker-VaccDscaff12-augustus-gene-60.42-mRN/
MIKC_MADS	AT3G54340.1	maker-VaccDscaff13-augustus-gene-45.48-mRN/
MIKC_MADS	AT3G54340.1	maker-VaccDscaff631-snap-gene-0.18-mRNA-1
MIKC_MADS	AT3G54340.1	maker-VaccDscaff32-augustus-gene-320.28-mRN
MIKC_MADS	AT3G54340.1	maker-VaccDscaff30-augustus-gene-300.28-mRN
MIKC_MADS	AT3G54340.1	maker-VaccDscaff42-augustus-gene-17.36-mRN/
MIKC_MADS	AT3G54340.1	maker-VaccDscaff801-snap-gene-0.11-mRNA-1
Dof	AT3G55370.1	augustus_masked-VaccDscaff22-processed-gene
Dof	AT3G55370.1	maker-VaccDscaff46-snap-gene-129.47-mRNA-1
MYB	AT3G55730.1	maker-VaccDscaff20-augustus-gene-45.19-mRN/
MYB	AT3G55730.1	maker-VaccDscaff28-augustus-gene-310.39-mRN
WRKY	AT3G56400.1	maker-VaccDscaff10-snap-gene-1.30-mRNA-1
WRKY	AT3G56400.1	augustus_masked-VaccDscaff13-processed-gene
WRKY	AT3G56400.1	maker-VaccDscaff42-augustus-gene-227.32-mRN
bZIP	AT3G56850.1	maker-VaccDscaff21-snap-gene-316.33-mRNA-1
bZIP	AT3G56850.1	augustus_masked-VaccDscaff26-processed-gene
bZIP	AT3G56850.1	augustus_masked-VaccDscaff29-processed-gene
MIKC_MADS	AT3G57230.2	maker-VaccDscaff12-augustus-gene-132.21-mRN
MIKC_MADS	AT3G57230.2	snap_masked-VaccDscaff16-processed-gene-34
MIKC_MADS	AT3G57230.2	snap_masked-VaccDscaff7-processed-gene-62.2
MIKC_MADS	AT3G57230.2	snap_masked-VaccDscaff31-processed-gene-29
MIKC_MADS	AT3G57230.2	snap_masked-VaccDscaff18-processed-gene-51.

MIKC_MADS	AT3G57230.2	augustus_masked-VaccDscaff23-processed-gene
MIKC_MADS	AT3G57230.2	snap_masked-VaccDscaff3-processed-gene-24.1
ERF	AT3G57600.1	augustus_masked-VaccDscaff27-processed-gene
ERF	AT3G57600.1	augustus_masked-VaccDscaff45-processed-gene
ERF	AT3G57600.1	augustus_masked-VaccDscaff17-processed-gene
C2H2	AT3G57670.1	maker-VaccDscaff22-augustus-gene-83.33-mRNA
C2H2	AT3G57670.1	maker-VaccDscaff21-augustus-gene-354.25-mRN
C2H2	AT3G57670.1	augustus_masked-VaccDscaff1532-processed-ge
C2H2	AT3G57670.1	maker-VaccDscaff33-augustus-gene-20.34-mRNA
C2H2	AT3G57670.1	maker-VaccDscaff26-augustus-gene-346.28-mRN
C2H2	AT3G57670.1	augustus_masked-VaccDscaff1490-processed-ge
C2H2	AT3G57670.1	maker-VaccDscaff44-augustus-gene-208.24-mRN
C2H2	AT3G57670.1	maker-VaccDscaff29-augustus-gene-334.27-mRN
bZIP	AT3G58120.1	maker-VaccDscaff38-augustus-gene-218.40-mRN
bZIP	AT3G58120.1	maker-VaccDscaff6-augustus-gene-147.30-mRNA
bZIP	AT3G58120.1	augustus_masked-VaccDscaff37-processed-gene
bZIP	AT3G58120.1	maker-VaccDscaff1278-augustus-gene-0.4-mRNA
bZIP	AT3G58120.1	maker-VaccDscaff39-augustus-gene-244.33-mRN
LBD	AT3G58190.1	augustus_masked-VaccDscaff22-processed-gene
LBD	AT3G58190.1	augustus_masked-VaccDscaff22-processed-gene
LBD	AT3G58190.1	maker-VaccDscaff43-augustus-gene-35.18-mRNA
LBD	AT3G58190.1	augustus_masked-VaccDscaff43-processed-gene
LBD	AT3G58190.1	maker-VaccDscaff46-augustus-gene-173.19-mRN
LBD	AT3G58190.1	augustus_masked-VaccDscaff46-processed-gene
LBD	AT3G58190.1	augustus_masked-VaccDscaff47-processed-gene
LBD	AT3G58190.1	augustus_masked-VaccDscaff47-processed-gene
MIKC_MADS	AT3G58780.1	maker-VaccDscaff6-snap-gene-173.22-mRNA-1
MIKC_MADS	AT3G58780.3	maker-VaccDscaff39-augustus-gene-214.16-mRN
SBP	AT3G60030.1	maker-VaccDscaff10-augustus-gene-398.28-mRN
SBP	AT3G60030.1	maker-VaccDscaff8-augustus-gene-410.30-mRNA
SBP	AT3G60030.1	maker-VaccDscaff5-augustus-gene-3.28-mRNA-1
SBP	AT3G60030.1	maker-VaccDscaff1-augustus-gene-3.18-mRNA-1
MYB_related	AT3G60110.1	snap_masked-VaccDscaff23-processed-gene-365
HD-ZIP	AT3G60390.1	maker-VaccDscaff22-augustus-gene-50.31-mRNA
MYB	AT3G60460.1	maker-VaccDscaff22-augustus-gene-154.19-mRN
MYB	AT3G60460.1	augustus_masked-VaccDscaff43-processed-gene
MYB	AT3G60460.1	augustus_masked-VaccDscaff12-processed-gene
MYB	AT3G60460.1	maker-VaccDscaff46-snap-gene-35.33-mRNA-1
MYB	AT3G60460.1	augustus_masked-VaccDscaff40-processed-gene
MYB	AT3G60460.1	augustus_masked-VaccDscaff41-processed-gene
MYB	AT3G60460.1	maker-VaccDscaff23-augustus-gene-204.45-mRN
GATA	AT3G60530.1	maker-VaccDscaff12-augustus-gene-271.25-mRN
GATA	AT3G60530.1	maker-VaccDscaff40-augustus-gene-155.34-mRN
GATA	AT3G60530.1	maker-VaccDscaff41-augustus-gene-144.15-mRN
GATA	AT3G60530.1	maker-VaccDscaff23-augustus-gene-138.22-mRN
HD-ZIP	AT3G61150.1	maker-VaccDscaff21-snap-gene-65.59-mRNA-1

HD-ZIP	AT3G61150.1	maker-VaccDscaff33-augustus-gene-271.34-mRN
HD-ZIP	AT3G61150.1	maker-VaccDscaff29-augustus-gene-66.49-mRN/
MYB	AT3G61250.1	maker-VaccDscaff22-snap-gene-24.47-mRNA-1
MYB	AT3G61250.1	maker-VaccDscaff22-snap-gene-24.47-mRNA-1
MYB	AT3G61250.1	maker-VaccDscaff22-snap-gene-24.46-mRNA-1
MYB	AT3G61250.1	maker-VaccDscaff21-augustus-gene-63.28-mRN/
MYB	AT3G61250.1	maker-VaccDscaff753-snap-gene-0.17-mRNA-1
MYB	AT3G61250.1	maker-VaccDscaff12-snap-gene-288.13-mRNA-1
MYB	AT3G61250.1	maker-VaccDscaff33-augustus-gene-273.23-mRN
MYB	AT3G61250.1	augustus_masked-VaccDscaff562-processed-gen
MYB	AT3G61250.1	maker-VaccDscaff26-augustus-gene-63.26-mRN/
MYB	AT3G61250.1	maker-VaccDscaff40-snap-gene-180.28-mRNA-1
MYB	AT3G61250.1	maker-VaccDscaff41-snap-gene-170.21-mRNA-1
MYB	AT3G61250.1	maker-VaccDscaff569-snap-gene-0.19-mRNA-1
MYB	AT3G61250.1	maker-VaccDscaff569-snap-gene-0.18-mRNA-1
MYB	AT3G61250.1	snap_masked-VaccDscaff30-processed-gene-17.
MYB	AT3G61250.1	maker-VaccDscaff574-snap-gene-0.18-mRNA-1
MYB	AT3G61250.1	maker-VaccDscaff575-snap-gene-0.19-mRNA-1
MYB	AT3G61250.1	maker-VaccDscaff23-augustus-gene-105.26-mRN
MYB	AT3G61250.1	maker-VaccDscaff29-augustus-gene-64.35-mRN/
ARF	AT3G61830.1	maker-VaccDscaff209-augustus-gene-0.17-mRN/
ARF	AT3G61830.1	maker-VaccDscaff20-augustus-gene-363.23-mRN
ARF	AT3G61830.1	maker-VaccDscaff49-augustus-gene-18.25-mRN/
ARF	AT3G61830.1	maker-VaccDscaff28-snap-gene-12.29-mRNA-1
HD-ZIP	AT3G61890.1	maker-VaccDscaff108-augustus-gene-3.29-mRN/
HD-ZIP	AT3G61890.1	maker-VaccDscaff6-augustus-gene-255.14-mRN/
HD-ZIP	AT3G61890.1	maker-VaccDscaff39-augustus-gene-174.30-mRN
bZIP	AT3G62420.1	augustus_masked-VaccDscaff22-processed-gene
bZIP	AT3G62420.1	augustus_masked-VaccDscaff43-processed-gene
bZIP	AT3G62420.1	snap_masked-VaccDscaff25-processed-gene-327
bZIP	AT3G62420.1	augustus_masked-VaccDscaff2-processed-gene-:
bZIP	AT3G62420.1	augustus_masked-VaccDscaff46-processed-gene
bZIP	AT3G62420.1	augustus_masked-VaccDscaff14-processed-gene
bZIP	AT3G62420.1	snap_masked-VaccDscaff3-processed-gene-74.2
bZIP	AT3G62420.1	augustus_masked-VaccDscaff47-processed-gene
bHLH	AT4G00050.1	maker-VaccDscaff22-augustus-gene-46.34-mRN/
bHLH	AT4G00120.1	augustus_masked-VaccDscaff13-processed-gene
bHLH	AT4G00120.1	augustus_masked-VaccDscaff30-processed-gene
bHLH	AT4G00120.1	augustus_masked-VaccDscaff42-processed-gene
GRAS	AT4G00150.1	augustus_masked-VaccDscaff22-processed-gene
LBD	AT4G00210.1	maker-VaccDscaff40-snap-gene-170.39-mRNA-1
LBD	AT4G00210.1	maker-VaccDscaff41-augustus-gene-159.30-mRN
LBD	AT4G00210.1	maker-VaccDscaff23-augustus-gene-122.27-mRN
LBD	AT4G00210.1	maker-VaccDscaff23-augustus-gene-125.22-mRN
HD-ZIP	AT4G00730.1	maker-VaccDscaff22-augustus-gene-27.16-mRN/
HD-ZIP	AT4G00730.1	maker-VaccDscaff20-snap-gene-20.39-mRNA-1

HD-ZIP	AT4G00730.1	maker-VaccDscaff26-augustus-gene-65.47-mRNA
HD-ZIP	AT4G00730.1	maker-VaccDscaff19-snap-gene-19.41-mRNA-1
HD-ZIP	AT4G00730.1	maker-VaccDscaff28-snap-gene-352.46-mRNA-1
HD-ZIP	AT4G00730.1	maker-VaccDscaff48-snap-gene-79.39-mRNA-1
HD-ZIP	AT4G00730.2	maker-VaccDscaff46-snap-gene-50.39-mRNA-1
HB-other	AT4G02560.2	maker-VaccDscaff11-snap-gene-384.42-mRNA-1
HB-other	AT4G02560.2	maker-VaccDscaff11-snap-gene-384.42-mRNA-1
HB-other	AT4G02560.2	maker-VaccDscaff24-augustus-gene-13.26-mRNA
HB-other	AT4G02560.2	maker-VaccDscaff24-augustus-gene-13.26-mRNA
HB-other	AT4G02560.2	maker-VaccDscaff51-snap-gene-15.31-mRNA-1
HB-other	AT4G02560.2	maker-VaccDscaff51-snap-gene-15.31-mRNA-1
bHLH	AT4G02590.2	maker-VaccDscaff22-augustus-gene-342.30-mRN
bHLH	AT4G02590.2	maker-VaccDscaff43-snap-gene-30.40-mRNA-1
bHLH	AT4G02590.2	maker-VaccDscaff36-augustus-gene-278.29-mRN
bHLH	AT4G02590.2	maker-VaccDscaff46-augustus-gene-177.36-mRN
bHLH	AT4G02590.2	maker-VaccDscaff5-augustus-gene-106.33-mRNA
bHLH	AT4G02590.2	snap_masked-VaccDscaff9-processed-gene-36.9
bHLH	AT4G02590.2	maker-VaccDscaff1-augustus-gene-91.23-mRNA-
bHLH	AT4G02590.2	maker-VaccDscaff35-augustus-gene-29.28-mRNA
bHLH	AT4G02590.2	maker-VaccDscaff4-augustus-gene-58.29-mRNA-
bHLH	AT4G02590.2	maker-VaccDscaff47-augustus-gene-169.33-mRN
bHLH	AT4G02590.3	augustus_masked-VaccDscaff4-processed-gene-
C2H2	AT4G02670.1	maker-VaccDscaff43-augustus-gene-28.20-mRNA
HB-other	AT4G03250.1	maker-VaccDscaff11-augustus-gene-68.24-mRNA
HB-other	AT4G03250.1	maker-VaccDscaff19-augustus-gene-169.22-mRN
HB-other	AT4G03250.1	maker-VaccDscaff15-augustus-gene-75.25-mRNA
HB-other	AT4G03250.1	maker-VaccDscaff24-augustus-gene-343.33-mRN
C2H2	AT4G06634.1	maker-VaccDscaff2-augustus-gene-364.31-mRNA
TALE	AT4G08150.1	maker-VaccDscaff12-augustus-gene-123.27-mRN
TALE	AT4G08150.1	maker-VaccDscaff40-augustus-gene-51.18-mRNA
TALE	AT4G08150.1	maker-VaccDscaff41-augustus-gene-18.14-mRNA
TALE	AT4G08150.1	maker-VaccDscaff23-augustus-gene-291.17-mRN
GRAS	AT4G08250.1	augustus_masked-VaccDscaff6-processed-gene-
GRAS	AT4G08250.1	augustus_masked-VaccDscaff37-processed-gene
GRAS	AT4G08250.1	augustus_masked-VaccDscaff37-processed-gene
GRAS	AT4G08250.1	augustus_masked-VaccDscaff702-processed-gen
MYB	AT4G09460.1	maker-VaccDscaff36-augustus-gene-8.19-mRNA-
MYB	AT4G09460.1	maker-VaccDscaff36-augustus-gene-113.22-mRN
MYB	AT4G09460.1	maker-VaccDscaff13-augustus-gene-102.20-mRN
MYB	AT4G09460.1	maker-VaccDscaff9-augustus-gene-307.24-mRNA
MYB	AT4G09460.1	maker-VaccDscaff9-augustus-gene-407.23-mRNA
MYB	AT4G09460.1	maker-VaccDscaff35-augustus-gene-237.26-mRN
MYB	AT4G09460.1	maker-VaccDscaff35-augustus-gene-317.22-mRN
MYB	AT4G09460.1	maker-VaccDscaff4-augustus-gene-330.20-mRNA
MYB	AT4G09460.1	maker-VaccDscaff4-augustus-gene-410.23-mRNA
bHLH	AT4G09820.1	augustus_masked-VaccDscaff11-processed-gene

bHLH	AT4G09820.1	augustus_masked-VaccDscaff11-processed-gene
bHLH	AT4G09820.1	augustus_masked-VaccDscaff11-processed-gene
bHLH	AT4G09820.1	maker-VaccDscaff19-augustus-gene-381.30-mRN
bHLH	AT4G09820.1	maker-VaccDscaff19-augustus-gene-381.30-mRN
bHLH	AT4G09820.1	maker-VaccDscaff19-augustus-gene-381.30-mRN
bHLH	AT4G09820.1	maker-VaccDscaff15-augustus-gene-371.25-mRN
bHLH	AT4G09820.1	maker-VaccDscaff15-augustus-gene-371.25-mRN
bHLH	AT4G09820.1	maker-VaccDscaff15-augustus-gene-371.25-mRN
bHLH	AT4G09820.1	maker-VaccDscaff24-augustus-gene-24.28-mRNA
bHLH	AT4G09820.1	maker-VaccDscaff24-augustus-gene-24.28-mRNA
bHLH	AT4G09820.1	maker-VaccDscaff24-augustus-gene-24.28-mRNA
MIKC_MADS	AT4G09960.1	maker-VaccDscaff2-augustus-gene-218.35-mRNA
MIKC_MADS	AT4G09960.1	maker-VaccDscaff14-augustus-gene-172.28-mRN
MIKC_MADS	AT4G09960.1	maker-VaccDscaff3-augustus-gene-224.23-mRNA
MIKC_MADS	AT4G09960.2	maker-VaccDscaff2-augustus-gene-218.36-mRNA
MIKC_MADS	AT4G09960.2	maker-VaccDscaff14-augustus-gene-172.29-mRN
NAC	AT4G10350.1	maker-VaccDscaff25-snap-gene-184.31-mRNA-1
NAC	AT4G10350.1	maker-VaccDscaff2-augustus-gene-215.31-mRNA
NAC	AT4G10350.1	maker-VaccDscaff14-augustus-gene-168.43-mRN
NAC	AT4G10350.1	maker-VaccDscaff3-augustus-gene-228.23-mRNA
C2H2	AT4G12240.1	maker-VaccDscaff43-snap-gene-231.52-mRNA-1
C2H2	AT4G12240.1	maker-VaccDscaff13-augustus-gene-277.36-mRN
C2H2	AT4G12240.1	augustus_masked-VaccDscaff46-processed-gene
C2H2	AT4G12240.1	maker-VaccDscaff32-augustus-gene-89.30-mRNA
C2H2	AT4G12240.1	maker-VaccDscaff30-snap-gene-123.24-mRNA-1
C2H2	AT4G12240.1	snap_masked-VaccDscaff42-processed-gene-18
MYB	AT4G12350.1	maker-VaccDscaff22-augustus-gene-139.29-mRN
MYB	AT4G12350.1	maker-VaccDscaff22-augustus-gene-139.29-mRN
MYB	AT4G12350.1	maker-VaccDscaff43-augustus-gene-229.28-mRN
MYB	AT4G12350.1	maker-VaccDscaff46-augustus-gene-18.51-mRNA
HB-other	AT4G12750.1	maker-VaccDscaff20-snap-gene-89.36-mRNA-1
HB-other	AT4G12750.1	maker-VaccDscaff19-snap-gene-85.42-mRNA-1
HB-other	AT4G12750.1	maker-VaccDscaff28-augustus-gene-272.28-mRN
HB-other	AT4G12750.1	maker-VaccDscaff48-snap-gene-30.34-mRNA-1
MYB	AT4G13480.1	maker-VaccDscaff25-augustus-gene-144.17-mRN
MYB	AT4G13480.1	maker-VaccDscaff14-augustus-gene-116.33-mRN
MYB	AT4G13480.1	augustus_masked-VaccDscaff3-processed-gene-
G2-like	AT4G13640.1	maker-VaccDscaff25-augustus-gene-71.29-mRNA
G2-like	AT4G13640.1	maker-VaccDscaff11-snap-gene-220.34-mRNA-1
G2-like	AT4G13640.1	maker-VaccDscaff2-augustus-gene-73.21-mRNA-
G2-like	AT4G13640.1	maker-VaccDscaff14-augustus-gene-94.22-mRNA
G2-like	AT4G13640.1	maker-VaccDscaff3-augustus-gene-341.18-mRNA
G2-like	AT4G13640.1	maker-VaccDscaff3-snap-gene-391.39-mRNA-1
G2-like	AT4G13640.1	maker-VaccDscaff3-snap-gene-393.41-mRNA-1
HSF	AT4G13980.1	maker-VaccDscaff36-augustus-gene-226.26-mRN
HSF	AT4G13980.1	maker-VaccDscaff9-augustus-gene-78.39-mRNA-

HSF	AT4G13980.1	maker-VaccDscaff4-augustus-gene-122.41-mRN/
NF-YB	AT4G14540.1	maker-VaccDscaff10-augustus-gene-37.38-mRN/
NF-YB	AT4G14540.1	augustus_masked-VaccDscaff8-processed-gene-[illegible]
NF-YB	AT4G14540.1	snap_masked-VaccDscaff38-processed-gene-93.
NF-YB	AT4G14540.1	snap_masked-VaccDscaff38-processed-gene-28[illegible]
NF-YB	AT4G14540.1	augustus_masked-VaccDscaff13-processed-gene
NF-YB	AT4G14540.1	snap_masked-VaccDscaff6-processed-gene-320.
NF-YB	AT4G14540.1	augustus_masked-VaccDscaff5-processed-gene-[illegible]
NF-YB	AT4G14540.1	augustus_masked-VaccDscaff37-processed-gene
NF-YB	AT4G14540.1	snap_masked-VaccDscaff1-processed-gene-405.
NF-YB	AT4G14540.1	augustus_masked-VaccDscaff30-processed-gene
NF-YB	AT4G14540.1	snap_masked-VaccDscaff42-processed-gene-51.
NF-YB	AT4G14540.1	snap_masked-VaccDscaff23-processed-gene-27[illegible]
NF-YB	AT4G14540.1	snap_masked-VaccDscaff39-processed-gene-99.
ARR-B	AT4G16110.1	maker-VaccDscaff10-snap-gene-382.28-mRNA-1
ARR-B	AT4G16110.1	maker-VaccDscaff8-snap-gene-394.21-mRNA-1
ARR-B	AT4G16110.1	maker-VaccDscaff20-augustus-gene-147.18-mRN
ARR-B	AT4G16110.1	maker-VaccDscaff1-augustus-gene-21.22-mRNA-
ARR-B	AT4G16110.1	maker-VaccDscaff44-augustus-gene-177.24-mRN
ARR-B	AT4G16110.1	maker-VaccDscaff4-snap-gene-5.49-mRNA-1
ARR-B	AT4G16110.1	maker-VaccDscaff4-snap-gene-9.54-mRNA-1
ARR-B	AT4G16110.1	maker-VaccDscaff4-snap-gene-30.31-mRNA-1
ARR-B	AT4G16110.1	maker-VaccDscaff28-augustus-gene-175.24-mRN
CAMTA	AT4G16150.1	maker-VaccDscaff10-augustus-gene-384.17-mRN
CAMTA	AT4G16150.1	maker-VaccDscaff8-augustus-gene-396.14-mRN/
CAMTA	AT4G16150.1	maker-VaccDscaff5-augustus-gene-17.20-mRNA-
CAMTA	AT4G16150.1	augustus_masked-VaccDscaff9-processed-gene-[illegible]
CAMTA	AT4G16150.1	maker-VaccDscaff1-augustus-gene-19.24-mRNA-
CAMTA	AT4G16150.1	maker-VaccDscaff4-augustus-gene-5.37-mRNA-1
CAMTA	AT4G16150.1	maker-VaccDscaff4-augustus-gene-10.30-mRNA-
CAMTA	AT4G16150.1	maker-VaccDscaff4-augustus-gene-31.48-mRNA-
bHLH	AT4G16430.1	augustus_masked-VaccDscaff22-processed-gene
bHLH	AT4G16430.1	augustus_masked-VaccDscaff43-processed-gene
bHLH	AT4G16430.1	augustus_masked-VaccDscaff46-processed-gene
bHLH	AT4G16430.1	augustus_masked-VaccDscaff47-processed-gene
bHLH	AT4G16430.1	maker-VaccDscaff47-snap-gene-166.46-mRNA-1
bHLH	AT4G16430.1	maker-VaccDscaff47-snap-gene-166.46-mRNA-1
HD-ZIP	AT4G16780.1	maker-VaccDscaff22-augustus-gene-155.20-mRN
HD-ZIP	AT4G16780.1	maker-VaccDscaff22-augustus-gene-156.19-mRN
HD-ZIP	AT4G16780.1	maker-VaccDscaff43-augustus-gene-212.28-mRN
HD-ZIP	AT4G16780.1	maker-VaccDscaff43-augustus-gene-212.29-mRN
HD-ZIP	AT4G16780.1	maker-VaccDscaff46-augustus-gene-36.13-mRN/
HD-ZIP	AT4G16780.1	maker-VaccDscaff46-augustus-gene-37.21-mRN/
ERF	AT4G17500.1	snap_masked-VaccDscaff22-processed-gene-15[illegible]
ERF	AT4G17500.1	augustus_masked-VaccDscaff43-processed-gene
ERF	AT4G17500.1	augustus_masked-VaccDscaff21-processed-gene

ERF	AT4G17500.1	augustus_masked-VaccDscaff20-processed-gene
ERF	AT4G17500.1	augustus_masked-VaccDscaff25-processed-gene
ERF	AT4G17500.1	augustus_masked-VaccDscaff33-processed-gene
ERF	AT4G17500.1	snap_masked-VaccDscaff26-processed-gene-95.
ERF	AT4G17500.1	augustus_masked-VaccDscaff19-processed-gene
ERF	AT4G17500.1	maker-VaccDscaff9-snap-gene-45.39-mRNA-1
ERF	AT4G17500.1	augustus_masked-VaccDscaff4-processed-gene-
ERF	AT4G17500.1	augustus_masked-VaccDscaff29-processed-gene
ERF	AT4G17500.1	augustus_masked-VaccDscaff28-processed-gene
ERF	AT4G17500.1	augustus_masked-VaccDscaff48-processed-gene
GATA	AT4G17570.3	snap_masked-VaccDscaff25-processed-gene-18.
GATA	AT4G17570.3	maker-VaccDscaff46-augustus-gene-42.40-mRN
GATA	AT4G17570.3	maker-VaccDscaff105-augustus-gene-4.47-mRN
bHLH	AT4G17880.1	maker-VaccDscaff22-augustus-gene-339.33-mRN
bHLH	AT4G17880.1	maker-VaccDscaff43-snap-gene-33.39-mRNA-1
bHLH	AT4G17880.1	maker-VaccDscaff46-augustus-gene-174.44-mRN
MYB	AT4G18770.1	maker-VaccDscaff27-augustus-gene-362.34-mRN
MYB	AT4G18770.1	maker-VaccDscaff16-augustus-gene-33.26-mRN
MYB	AT4G18770.1	maker-VaccDscaff7-snap-gene-391.31-mRNA-1
MYB	AT4G18770.1	maker-VaccDscaff18-augustus-gene-365.20-mRN
MIKC_MADS	AT4G18960.1	augustus_masked-VaccDscaff20-processed-gene
MIKC_MADS	AT4G18960.1	maker-VaccDscaff44-augustus-gene-153.36-mRN
MIKC_MADS	AT4G18960.1	maker-VaccDscaff28-augustus-gene-145.15-mRN
LSD	AT4G20380.7	maker-VaccDscaff20-snap-gene-226.18-mRNA-1
LSD	AT4G20380.7	maker-VaccDscaff44-snap-gene-131.30-mRNA-1
LSD	AT4G20380.7	maker-VaccDscaff28-snap-gene-128.23-mRNA-1
MYB	AT4G21440.1	maker-VaccDscaff38-augustus-gene-245.26-mRN
MYB	AT4G21440.1	maker-VaccDscaff11-augustus-gene-183.34-mRN
MYB	AT4G21440.1	maker-VaccDscaff15-augustus-gene-160.24-mRN
MYB	AT4G21440.1	maker-VaccDscaff9-augustus-gene-136.29-mRN
MYB	AT4G21440.1	maker-VaccDscaff37-augustus-gene-38.17-mRN
MYB	AT4G21440.1	maker-VaccDscaff35-augustus-gene-92.23-mRN
MYB	AT4G21440.1	maker-VaccDscaff24-snap-gene-213.30-mRNA-1
MYB	AT4G21440.1	maker-VaccDscaff4-augustus-gene-182.17-mRN
LSD	AT4G21610.1	maker-VaccDscaff11-snap-gene-161.33-mRNA-1
LSD	AT4G21610.1	maker-VaccDscaff19-snap-gene-227.31-mRNA-1
LSD	AT4G21610.1	maker-VaccDscaff15-augustus-gene-142.23-mRN
WRKY	AT4G23810.1	augustus_masked-VaccDscaff42-processed-gene
ARF	AT4G23980.1	maker-VaccDscaff38-augustus-gene-161.11-mRN
ARF	AT4G23980.1	maker-VaccDscaff6-augustus-gene-262.13-mRN
ARF	AT4G23980.1	maker-VaccDscaff49-snap-gene-19.31-mRNA-1
ARF	AT4G23980.1	maker-VaccDscaff37-augustus-gene-145.14-mRN
ARF	AT4G23980.1	maker-VaccDscaff39-augustus-gene-167.7-mRN
Nin-like	AT4G24020.1	maker-VaccDscaff36-snap-gene-255.37-mRNA-1
Nin-like	AT4G24020.1	snap_masked-VaccDscaff36-processed-gene-25
Nin-like	AT4G24020.1	maker-VaccDscaff44-snap-gene-5.21-mRNA-1

Nin-like	AT4G24020.1	maker-VaccDscaff44-snap-gene-5.21-mRNA-1
Nin-like	AT4G24020.1	snap_masked-VaccDscaff4-processed-gene-75.1
WRKY	AT4G24240.1	maker-VaccDscaff40-augustus-gene-29.27-mRN
WRKY	AT4G24240.1	maker-VaccDscaff41-augustus-gene-40.26-mRN
WRKY	AT4G24240.1	maker-VaccDscaff41-augustus-gene-41.27-mRN
MIKC_MADS	AT4G24540.1	maker-VaccDscaff12-snap-gene-134.39-mRNA-1
MIKC_MADS	AT4G24540.1	snap_masked-VaccDscaff2-processed-gene-380.
MIKC_MADS	AT4G24540.1	augustus_masked-VaccDscaff40-processed-gene
MIKC_MADS	AT4G24540.1	maker-VaccDscaff40-augustus-gene-59.26-mRN
MIKC_MADS	AT4G24540.1	maker-VaccDscaff41-snap-gene-6.34-mRNA-1
MIKC_MADS	AT4G24540.1	snap_masked-VaccDscaff14-processed-gene-32
MIKC_MADS	AT4G24540.1	snap_masked-VaccDscaff23-processed-gene-27
ZF-HD	AT4G24660.1	maker-VaccDscaff12-augustus-gene-144.13-mR
ZF-HD	AT4G24660.1	augustus_masked-VaccDscaff6-processed-gene-
ZF-HD	AT4G24660.1	maker-VaccDscaff5-augustus-gene-72.23-mRNA-
ZF-HD	AT4G24660.1	maker-VaccDscaff40-augustus-gene-67.20-mRN
ZF-HD	AT4G24660.1	maker-VaccDscaff40-augustus-gene-67.20-mRN
ZF-HD	AT4G24660.1	augustus_masked-VaccDscaff37-processed-gene
ZF-HD	AT4G24660.1	augustus_masked-VaccDscaff39-processed-gene
ERF	AT4G25470.1	augustus_masked-VaccDscaff21-processed-gene
ERF	AT4G25470.1	augustus_masked-VaccDscaff21-processed-gene
ERF	AT4G25470.1	augustus_masked-VaccDscaff33-processed-gene
ERF	AT4G25470.1	maker-VaccDscaff33-snap-gene-267.37-mRNA-1
ERF	AT4G25470.1	maker-VaccDscaff33-snap-gene-267.37-mRNA-1
ERF	AT4G25470.1	augustus_masked-VaccDscaff26-processed-gene
ERF	AT4G25470.1	snap_masked-VaccDscaff26-processed-gene-69.
ERF	AT4G25470.1	augustus_masked-VaccDscaff29-processed-gene
ERF	AT4G25470.1	maker-VaccDscaff29-snap-gene-70.37-mRNA-1
ERF	AT4G25480.1	augustus_masked-VaccDscaff21-processed-gene
ERF	AT4G25480.1	maker-VaccDscaff33-snap-gene-267.38-mRNA-1
ERF	AT4G25480.1	augustus_masked-VaccDscaff26-processed-gene
ERF	AT4G25480.1	augustus_masked-VaccDscaff29-processed-gene
ERF	AT4G25490.1	snap_masked-VaccDscaff21-processed-gene-70.
ERF	AT4G25490.1	snap_masked-VaccDscaff26-processed-gene-69.
WRKY	AT4G26640.2	maker-VaccDscaff10-augustus-gene-0.24-mRNA-
WRKY	AT4G26640.2	maker-VaccDscaff8-augustus-gene-9.44-mRNA-1
WRKY	AT4G26640.2	maker-VaccDscaff6-snap-gene-34.52-mRNA-1
WRKY	AT4G26640.2	maker-VaccDscaff6-snap-gene-34.52-mRNA-1
WRKY	AT4G26640.2	maker-VaccDscaff5-augustus-gene-416.39-mRN
WRKY	AT4G26640.2	snap_masked-VaccDscaff103-processed-gene-2.
WRKY	AT4G26640.2	maker-VaccDscaff1-augustus-gene-447.28-mRN
WRKY	AT4G26640.2	maker-VaccDscaff93-augustus-gene-5.49-mRNA-
WRKY	AT4G26640.2	maker-VaccDscaff93-augustus-gene-5.49-mRNA-
MYB	AT4G26930.1	maker-VaccDscaff1-snap-gene-449.33-mRNA-1
NAC	AT4G27410.2	maker-VaccDscaff6-augustus-gene-65.18-mRNA-
NAC	AT4G27410.2	maker-VaccDscaff338-augustus-gene-0.27-mRN

NAC	AT4G28500.1	maker-VaccDscaff38-augustus-gene-220.26-mRN
NAC	AT4G28500.1	maker-VaccDscaff11-augustus-gene-155.22-mRN
NAC	AT4G28500.1	maker-VaccDscaff6-augustus-gene-144.18-mRNA
NAC	AT4G28500.1	maker-VaccDscaff19-augustus-gene-221.18-mRN
NAC	AT4G28500.1	maker-VaccDscaff15-augustus-gene-136.29-mRN
NAC	AT4G28500.1	maker-VaccDscaff37-augustus-gene-60.25-mRNA
NAC	AT4G28500.1	maker-VaccDscaff24-augustus-gene-241.17-mRN
NAC	AT4G28500.1	maker-VaccDscaff39-augustus-gene-246.25-mRN
NAC	AT4G28530.1	maker-VaccDscaff22-augustus-gene-331.26-mRN
NAC	AT4G28530.1	maker-VaccDscaff43-augustus-gene-42.25-mRNA
NAC	AT4G28530.1	maker-VaccDscaff46-augustus-gene-166.16-mRN
NAC	AT4G28530.1	maker-VaccDscaff15-snap-gene-359.30-mRNA-1
NAC	AT4G28530.1	maker-VaccDscaff24-snap-gene-36.34-mRNA-1
NAC	AT4G28530.1	maker-VaccDscaff47-augustus-gene-158.17-mRN
NAC	AT4G28530.2	snap_masked-VaccDscaff25-processed-gene-41.
G2-like	AT4G28610.1	maker-VaccDscaff11-snap-gene-362.58-mRNA-1
G2-like	AT4G28610.1	augustus_masked-VaccDscaff19-processed-gene
G2-like	AT4G28610.1	maker-VaccDscaff755-augustus-gene-0.8-mRNA-
G2-like	AT4G28610.1	maker-VaccDscaff28-augustus-gene-265.16-mRN
G2-like	AT4G28610.1	augustus_masked-VaccDscaff48-processed-gene
CPP	AT4G29000.1	maker-VaccDscaff12-augustus-gene-328.25-mRN
CPP	AT4G29000.1	maker-VaccDscaff33-augustus-gene-320.27-mRN
CPP	AT4G29000.1	maker-VaccDscaff130-snap-gene-1.43-mRNA-1
CPP	AT4G29000.1	maker-VaccDscaff41-snap-gene-208.31-mRNA-1
CPP	AT4G29000.1	maker-VaccDscaff69-augustus-gene-7.58-mRNA-
bHLH	AT4G29100.1	maker-VaccDscaff27-snap-gene-318.57-mRNA-1
bHLH	AT4G29100.1	maker-VaccDscaff27-snap-gene-318.57-mRNA-1
C3H	AT4G29190.1	snap_masked-VaccDscaff258-processed-gene-0.
C3H	AT4G29190.1	augustus_masked-VaccDscaff25-processed-gene
C3H	AT4G29190.1	augustus_masked-VaccDscaff33-processed-gene
C3H	AT4G29190.1	augustus_masked-VaccDscaff2-processed-gene-
C3H	AT4G29190.1	snap_masked-VaccDscaff26-processed-gene-3.1
C3H	AT4G29190.1	augustus_masked-VaccDscaff14-processed-gene
C3H	AT4G29190.1	snap_masked-VaccDscaff29-processed-gene-0.9
C3H	AT4G29190.1	augustus_masked-VaccDscaff3-processed-gene-
HB-PHD	AT4G29940.2	maker-VaccDscaff12-snap-gene-312.36-mRNA-1
HB-PHD	AT4G29940.2	maker-VaccDscaff40-augustus-gene-207.28-mRN
HB-PHD	AT4G29940.2	maker-VaccDscaff41-augustus-gene-193.22-mRN
HB-PHD	AT4G29940.2	maker-VaccDscaff23-augustus-gene-80.27-mRNA
ARF	AT4G30080.1	maker-VaccDscaff10-augustus-gene-224.19-mRN
ARF	AT4G30080.1	maker-VaccDscaff8-augustus-gene-237.20-mRNA
ARF	AT4G30080.1	maker-VaccDscaff21-augustus-gene-177.33-mRN
ARF	AT4G30080.1	maker-VaccDscaff33-augustus-gene-171.13-mRN
ARF	AT4G30080.1	maker-VaccDscaff26-augustus-gene-178.26-mRN
ARF	AT4G30080.1	maker-VaccDscaff5-augustus-gene-173.17-mRNA
ARF	AT4G30080.1	maker-VaccDscaff1-augustus-gene-184.14-mRNA

ARF	AT4G30080.1	maker-VaccDscaff29-augustus-gene-171.22-mRN
MYB	AT4G32730.1	maker-VaccDscaff28-augustus-gene-105.31-mRN
ERF	AT4G32800.1	augustus_masked-VaccDscaff44-processed-gene
GATA	AT4G32890.1	maker-VaccDscaff17-snap-gene-355.52-mRNA-1
GATA	AT4G32890.1	maker-VaccDscaff34-augustus-gene-26.31-mRN
bHLH	AT4G33880.1	augustus_masked-VaccDscaff31-processed-gene
bHLH	AT4G33880.1	maker-VaccDscaff18-snap-gene-40.41-mRNA-1
ERF	AT4G34410.1	augustus_masked-VaccDscaff206-processed-gen
ERF	AT4G34410.1	augustus_masked-VaccDscaff27-processed-gene
ERF	AT4G34410.1	augustus_masked-VaccDscaff2-processed-gene-
ERF	AT4G34410.1	augustus_masked-VaccDscaff17-processed-gene
ERF	AT4G34410.1	augustus_masked-VaccDscaff14-processed-gene
ERF	AT4G34410.1	augustus_masked-VaccDscaff34-processed-gene
ERF	AT4G34410.1	augustus_masked-VaccDscaff377-processed-gen
bHLH	AT4G34530.1	maker-VaccDscaff1412-augustus-gene-0.4-mRN
bHLH	AT4G34530.1	maker-VaccDscaff2-augustus-gene-427.18-mRN
C2H2	AT4G35280.1	augustus_masked-VaccDscaff32-processed-gene
WOX	AT4G35550.1	maker-VaccDscaff12-augustus-gene-377.23-mRN
WOX	AT4G35550.1	maker-VaccDscaff40-augustus-gene-280.25-mRN
M-type_MADS	AT4G36590.1	snap_masked-VaccDscaff19-processed-gene-12
M-type_MADS	AT4G36590.1	snap_masked-VaccDscaff28-processed-gene-231
bZIP	AT4G36730.1	maker-VaccDscaff13-augustus-gene-154.14-mRN
bZIP	AT4G36730.1	maker-VaccDscaff32-augustus-gene-210.40-mRN
bZIP	AT4G36730.1	maker-VaccDscaff30-augustus-gene-218.25-mRN
TALE	AT4G36870.2	maker-VaccDscaff13-augustus-gene-147.19-mRN
TALE	AT4G36870.2	maker-VaccDscaff32-snap-gene-219.24-mRNA-1
AP2	AT4G36920.2	maker-VaccDscaff13-augustus-gene-122.18-mRN
AP2	AT4G36920.2	maker-VaccDscaff13-augustus-gene-122.18-mRN
AP2	AT4G36920.2	maker-VaccDscaff16-augustus-gene-167.15-mRN
AP2	AT4G36920.2	maker-VaccDscaff179-snap-gene-1.59-mRNA-1
AP2	AT4G36920.2	maker-VaccDscaff179-snap-gene-1.59-mRNA-1
AP2	AT4G36920.2	maker-VaccDscaff30-augustus-gene-244.22-mRN
AP2	AT4G36920.2	maker-VaccDscaff31-snap-gene-79.27-mRNA-1
AP2	AT4G36920.2	maker-VaccDscaff31-snap-gene-79.27-mRNA-1
AP2	AT4G36920.2	maker-VaccDscaff32-augustus-gene-250.21-mRN
AP2	AT4G36920.2	maker-VaccDscaff7-snap-gene-266.13-mRNA-1
bHLH	AT4G36930.1	maker-VaccDscaff12-snap-gene-97.30-mRNA-1
bHLH	AT4G36930.1	maker-VaccDscaff40-snap-gene-27.32-mRNA-1
bHLH	AT4G36930.1	maker-VaccDscaff41-snap-gene-44.35-mRNA-1
bHLH	AT4G36930.1	maker-VaccDscaff30-augustus-gene-243.35-mRN
bHLH	AT4G36930.1	maker-VaccDscaff23-augustus-gene-314.44-mRN
HSF	AT4G36990.1	maker-VaccDscaff16-augustus-gene-177.37-mRN
HSF	AT4G36990.1	maker-VaccDscaff7-augustus-gene-257.17-mRN
HSF	AT4G36990.1	maker-VaccDscaff31-augustus-gene-88.13-mRN
HSF	AT4G36990.1	maker-VaccDscaff18-augustus-gene-226.18-mRN
HSF	AT4G36990.1	maker-VaccDscaff32-augustus-gene-245.19-mRN

HSF	AT4G36990.1	maker-VaccDscaff30-augustus-gene-239.30-mRN
MYB	AT4G37260.1	augustus_masked-VaccDscaff12-processed-gene
MYB	AT4G37260.1	augustus_masked-VaccDscaff40-processed-gene
MYB	AT4G37260.1	augustus_masked-VaccDscaff41-processed-gene
MYB	AT4G37260.1	augustus_masked-VaccDscaff23-processed-gene
LBD	AT4G37540.1	maker-VaccDscaff12-augustus-gene-111.26-mRN
LBD	AT4G37540.1	maker-VaccDscaff12-augustus-gene-111.27-mRN
LBD	AT4G37540.1	maker-VaccDscaff40-augustus-gene-39.39-mRNA
LBD	AT4G37540.1	maker-VaccDscaff40-snap-gene-40.23-mRNA-1
LBD	AT4G37540.1	maker-VaccDscaff40-augustus-gene-40.21-mRNA
LBD	AT4G37540.1	maker-VaccDscaff41-augustus-gene-28.21-mRNA
LBD	AT4G37540.1	maker-VaccDscaff41-augustus-gene-28.20-mRNA
LBD	AT4G37540.1	augustus_masked-VaccDscaff23-processed-gene
LBD	AT4G37540.1	maker-VaccDscaff23-augustus-gene-301.28-mRN
LBD	AT4G37540.1	maker-VaccDscaff23-augustus-gene-301.29-mRN
GRAS	AT4G37650.1	augustus_masked-VaccDscaff13-processed-gene
GRAS	AT4G37650.1	augustus_masked-VaccDscaff32-processed-gene
GRAS	AT4G37650.1	augustus_masked-VaccDscaff32-processed-gene
bZIP	AT4G37730.1	augustus_masked-VaccDscaff12-processed-gene
bZIP	AT4G37730.1	snap_masked-VaccDscaff13-processed-gene-111
bZIP	AT4G37730.1	snap_masked-VaccDscaff40-processed-gene-45.
GRF	AT4G37740.1	maker-VaccDscaff22-augustus-gene-132.28-mRN
GRF	AT4G37740.1	maker-VaccDscaff43-snap-gene-235.45-mRNA-1
GRF	AT4G37740.1	maker-VaccDscaff46-augustus-gene-12.27-mRNA
AP2	AT4G37750.1	augustus_masked-VaccDscaff32-processed-gene
AP2	AT4G37750.1	maker-VaccDscaff13-augustus-gene-110.34-mRN
AP2	AT4G37750.1	maker-VaccDscaff18-augustus-gene-45.13-mRNA
AP2	AT4G37750.1	maker-VaccDscaff23-augustus-gene-294.23-mRN
AP2	AT4G37750.1	maker-VaccDscaff40-augustus-gene-46.21-mRNA
AP2	AT4G37750.1	maker-VaccDscaff41-augustus-gene-23.23-mRNA
AP2	AT4G37750.1	maker-VaccDscaff7-augustus-gene-57.21-mRNA-
HD-ZIP	AT4G37790.1	maker-VaccDscaff16-augustus-gene-349.16-mRN
HD-ZIP	AT4G37790.1	maker-VaccDscaff13-augustus-gene-109.19-mRN
HD-ZIP	AT4G37790.1	maker-VaccDscaff7-augustus-gene-58.18-mRNA-
HD-ZIP	AT4G37790.1	maker-VaccDscaff31-snap-gene-296.46-mRNA-1
HD-ZIP	AT4G37790.1	maker-VaccDscaff18-augustus-gene-47.18-mRNA
HD-ZIP	AT4G37790.1	maker-VaccDscaff32-augustus-gene-265.14-mRN
MIKC_MADS	AT4G37940.1	augustus_masked-VaccDscaff27-processed-gene
MIKC_MADS	AT4G37940.1	maker-VaccDscaff27-snap-gene-193.24-mRNA-1
MIKC_MADS	AT4G37940.1	maker-VaccDscaff27-augustus-gene-204.24-mRN
MIKC_MADS	AT4G37940.1	maker-VaccDscaff45-augustus-gene-192.31-mRN
MIKC_MADS	AT4G37940.1	maker-VaccDscaff45-augustus-gene-192.30-mRN
MIKC_MADS	AT4G37940.1	maker-VaccDscaff45-snap-gene-205.29-mRNA-1
MIKC_MADS	AT4G37940.1	snap_masked-VaccDscaff2-processed-gene-391.
MIKC_MADS	AT4G37940.1	maker-VaccDscaff17-snap-gene-212.32-mRNA-1
MIKC_MADS	AT4G37940.1	augustus_masked-VaccDscaff17-processed-gene

MIKC_MADS	AT4G37940.1	maker-VaccDscaff17-augustus-gene-222.31-mRN
MIKC_MADS	AT4G37940.1	maker-VaccDscaff17-snap-gene-223.19-mRNA-1
MIKC_MADS	AT4G37940.1	maker-VaccDscaff14-augustus-gene-337.25-mRN
MIKC_MADS	AT4G37940.1	maker-VaccDscaff34-snap-gene-152.25-mRNA-1
MYB	AT4G38620.1	maker-VaccDscaff38-augustus-gene-184.29-mRN
MYB	AT4G38620.1	maker-VaccDscaff239-augustus-gene-0.53-mRNA
MYB	AT4G38620.1	maker-VaccDscaff13-augustus-gene-302.21-mRN
MYB	AT4G38620.1	maker-VaccDscaff6-augustus-gene-184.14-mRNA
MYB	AT4G38620.1	maker-VaccDscaff2-augustus-gene-439.27-mRNA
MYB	AT4G38620.1	maker-VaccDscaff14-augustus-gene-385.22-mRN
MYB	AT4G38620.1	maker-VaccDscaff37-augustus-gene-96.16-mRNA
MYB	AT4G38620.1	maker-VaccDscaff32-augustus-gene-77.19-mRNA
MYB	AT4G38620.1	maker-VaccDscaff30-augustus-gene-93.26-mRNA
MYB	AT4G38620.1	maker-VaccDscaff34-augustus-gene-10.29-mRNA
MYB	AT4G38620.1	maker-VaccDscaff42-augustus-gene-203.27-mRN
MYB	AT4G38620.1	maker-VaccDscaff28-augustus-gene-197.19-mRN
bZIP	AT4G38900.3	maker-VaccDscaff13-augustus-gene-291.15-mRN
bZIP	AT4G38900.3	maker-VaccDscaff32-augustus-gene-100.29-mRN
bZIP	AT4G38900.3	maker-VaccDscaff30-augustus-gene-107.25-mRN
bZIP	AT4G38900.3	maker-VaccDscaff30-augustus-gene-110.52-mRN
MYB_related	AT4G39250.1	maker-VaccDscaff16-augustus-gene-369.19-mRN
MYB_related	AT4G39250.1	maker-VaccDscaff13-snap-gene-273.47-mRNA-1
MYB_related	AT4G39250.1	augustus_masked-VaccDscaff7-processed-gene-
MYB_related	AT4G39250.1	augustus_masked-VaccDscaff31-processed-gene
MYB_related	AT4G39250.1	maker-VaccDscaff2-snap-gene-426.30-mRNA-1
MYB_related	AT4G39250.1	maker-VaccDscaff2-augustus-gene-426.28-mRNA
MYB_related	AT4G39250.1	maker-VaccDscaff18-snap-gene-27.34-mRNA-1
MYB_related	AT4G39250.1	maker-VaccDscaff14-snap-gene-372.36-mRNA-1
MYB_related	AT4G39250.1	maker-VaccDscaff14-snap-gene-372.36-mRNA-1
MYB_related	AT4G39250.1	snap_masked-VaccDscaff32-processed-gene-84.
MYB_related	AT4G39250.1	augustus_masked-VaccDscaff42-processed-gene
WRKY	AT4G39410.1	maker-VaccDscaff38-augustus-gene-108.24-mRN
WRKY	AT4G39410.1	maker-VaccDscaff6-augustus-gene-310.35-mRNA
WRKY	AT4G39410.1	maker-VaccDscaff37-augustus-gene-207.30-mRN
WRKY	AT4G39410.1	maker-VaccDscaff39-augustus-gene-109.22-mRN
bHLH	AT5G01310.1	augustus_masked-VaccDscaff27-processed-gene
bHLH	AT5G01310.1	maker-VaccDscaff45-snap-gene-51.34-mRNA-1
bHLH	AT5G01310.1	maker-VaccDscaff17-snap-gene-73.29-mRNA-1
bHLH	AT5G01310.1	maker-VaccDscaff34-snap-gene-276.42-mRNA-1
Trihelix	AT5G01380.1	maker-VaccDscaff27-augustus-gene-19.21-mRNA
Trihelix	AT5G01380.1	maker-VaccDscaff45-augustus-gene-49.38-mRNA
Trihelix	AT5G01380.1	maker-VaccDscaff5-augustus-gene-200.33-mRNA
Trihelix	AT5G01380.1	maker-VaccDscaff17-augustus-gene-75.35-mRNA
Trihelix	AT5G01380.1	maker-VaccDscaff1-augustus-gene-228.17-mRNA
Trihelix	AT5G01380.1	maker-VaccDscaff34-augustus-gene-274.13-mRN
Trihelix	AT5G01380.1	maker-VaccDscaff73-augustus-gene-3.30-mRNA-

TALE	AT5G02030.1	maker-VaccDscaff17-snap-gene-59.28-mRNA-1
TALE	AT5G02030.1	maker-VaccDscaff34-augustus-gene-250.32-mRN
MYB	AT5G02320.2	maker-VaccDscaff27-snap-gene-33.37-mRNA-1
MYB	AT5G02320.2	maker-VaccDscaff17-snap-gene-51.28-mRNA-1
MYB	AT5G02320.2	maker-VaccDscaff34-augustus-gene-310.25-mRN
C2H2	AT5G03150.1	maker-VaccDscaff554-augustus-gene-0.17-mRNA
C2H2	AT5G03150.1	maker-VaccDscaff17-augustus-gene-87.19-mRNA
C2H2	AT5G03150.1	maker-VaccDscaff34-augustus-gene-304.23-mRN
E2F/DP	AT5G03415.1	maker-VaccDscaff27-augustus-gene-35.19-mRNA
E2F/DP	AT5G03415.1	maker-VaccDscaff36-snap-gene-218.43-mRNA-1
E2F/DP	AT5G03415.1	maker-VaccDscaff36-snap-gene-219.31-mRNA-1
E2F/DP	AT5G03415.1	maker-VaccDscaff36-snap-gene-220.45-mRNA-1
E2F/DP	AT5G03415.1	maker-VaccDscaff17-snap-gene-49.22-mRNA-1
E2F/DP	AT5G03415.1	maker-VaccDscaff9-snap-gene-87.35-mRNA-1
E2F/DP	AT5G03415.1	maker-VaccDscaff9-augustus-gene-88.23-mRNA-
E2F/DP	AT5G03415.1	maker-VaccDscaff34-snap-gene-312.17-mRNA-1
E2F/DP	AT5G03415.1	augustus_masked-VaccDscaff4-processed-gene-
E2F/DP	AT5G03415.2	maker-VaccDscaff11-snap-gene-266.37-mRNA-1
E2F/DP	AT5G03415.2	maker-VaccDscaff9-snap-gene-87.38-mRNA-1
E2F/DP	AT5G03415.2	maker-VaccDscaff24-augustus-gene-132.35-mRN
E2F/DP	AT5G03415.2	maker-VaccDscaff24-augustus-gene-133.33-mRN
Trihelix	AT5G03680.1	maker-VaccDscaff21-augustus-gene-250.23-mRN
Trihelix	AT5G03680.1	maker-VaccDscaff33-augustus-gene-46.22-mRNA
Trihelix	AT5G03680.1	maker-VaccDscaff26-augustus-gene-242.23-mRN
Trihelix	AT5G03680.1	maker-VaccDscaff29-augustus-gene-236.12-mRN
C2H2	AT5G03740.1	maker-VaccDscaff29-augustus-gene-233.29-mRN
HD-ZIP	AT5G03790.1	maker-VaccDscaff26-snap-gene-239.51-mRNA-1
HD-ZIP	AT5G03790.1	maker-VaccDscaff29-snap-gene-230.35-mRNA-1
C2H2	AT5G04240.1	maker-VaccDscaff21-augustus-gene-179.28-mRN
C2H2	AT5G04240.1	maker-VaccDscaff21-augustus-gene-179.28-mRN
C2H2	AT5G04240.1	maker-VaccDscaff21-augustus-gene-179.28-mRN
C2H2	AT5G04240.1	maker-VaccDscaff33-augustus-gene-169.11-mRN
C2H2	AT5G04240.1	maker-VaccDscaff33-augustus-gene-169.11-mRN
C2H2	AT5G04240.1	maker-VaccDscaff33-augustus-gene-169.11-mRN
C2H2	AT5G04240.1	maker-VaccDscaff26-snap-gene-180.31-mRNA-1
C2H2	AT5G04240.1	maker-VaccDscaff26-snap-gene-180.31-mRNA-1
C2H2	AT5G04240.1	maker-VaccDscaff26-snap-gene-180.31-mRNA-1
C2H2	AT5G04240.1	maker-VaccDscaff29-augustus-gene-174.12-mRN
C2H2	AT5G04240.1	maker-VaccDscaff29-augustus-gene-174.12-mRN
C2H2	AT5G04240.1	maker-VaccDscaff29-augustus-gene-174.12-mRN
NAC	AT5G04410.1	maker-VaccDscaff9-snap-gene-145.27-mRNA-1
MYB	AT5G04760.1	maker-VaccDscaff21-snap-gene-218.29-mRNA-1
MYB	AT5G04760.1	maker-VaccDscaff20-augustus-gene-80.36-mRNA
MYB	AT5G04760.1	maker-VaccDscaff33-augustus-gene-136.21-mRN
MYB	AT5G04760.1	maker-VaccDscaff26-augustus-gene-215.14-mRN
MYB	AT5G04760.1	maker-VaccDscaff19-augustus-gene-76.40-mRNA

MYB	AT5G04760.1	maker-VaccDscaff29-augustus-gene-207.25-mRN
MYB	AT5G04760.1	maker-VaccDscaff28-augustus-gene-278.29-mRN
MYB	AT5G04760.1	maker-VaccDscaff48-augustus-gene-39.41-mRNA
Trihelix	AT5G05550.2	maker-VaccDscaff20-augustus-gene-39.25-mRNA
NF-X1	AT5G05660.1	maker-VaccDscaff21-snap-gene-126.54-mRNA-1
NF-X1	AT5G05660.1	maker-VaccDscaff33-augustus-gene-210.29-mRN
NF-X1	AT5G05660.1	maker-VaccDscaff26-augustus-gene-129.41-mRN
NF-X1	AT5G05660.1	maker-VaccDscaff26-augustus-gene-129.41-mRN
NF-X1	AT5G05660.1	snap_masked-VaccDscaff26-processed-gene-129
NF-X1	AT5G05660.1	maker-VaccDscaff29-augustus-gene-127.27-mRN
LBD	AT5G06080.1	maker-VaccDscaff21-augustus-gene-143.22-mRN
LBD	AT5G06080.1	augustus_masked-VaccDscaff20-processed-gene
LBD	AT5G06080.1	augustus_masked-VaccDscaff20-processed-gene
LBD	AT5G06080.1	augustus_masked-VaccDscaff33-processed-gene
LBD	AT5G06080.1	maker-VaccDscaff26-augustus-gene-147.17-mRN
LBD	AT5G06080.1	augustus_masked-VaccDscaff19-processed-gene
LBD	AT5G06080.1	maker-VaccDscaff29-augustus-gene-144.28-mRN
LBD	AT5G06080.1	maker-VaccDscaff28-augustus-gene-304.27-mRN
HD-ZIP	AT5G06710.1	maker-VaccDscaff10-augustus-gene-126.33-mRN
HD-ZIP	AT5G06710.1	maker-VaccDscaff22-augustus-gene-77.25-mRNA
HD-ZIP	AT5G06710.1	maker-VaccDscaff33-augustus-gene-8.34-mRNA-
HD-ZIP	AT5G06710.1	maker-VaccDscaff703-augustus-gene-0.9-mRNA-
HD-ZIP	AT5G06710.1	maker-VaccDscaff5-augustus-gene-284.26-mRNA
G2-like	AT5G06800.1	snap_masked-VaccDscaff21-processed-gene-364
G2-like	AT5G06800.1	augustus_masked-VaccDscaff33-processed-gene
G2-like	AT5G06800.1	maker-VaccDscaff26-augustus-gene-351.28-mRN
G2-like	AT5G06800.1	maker-VaccDscaff29-augustus-gene-339.15-mRN
bZIP	AT5G06839.3	maker-VaccDscaff21-augustus-gene-363.31-mRN
bZIP	AT5G06839.3	maker-VaccDscaff33-snap-gene-15.29-mRNA-1
bZIP	AT5G06839.3	augustus_masked-VaccDscaff26-processed-gene
bZIP	AT5G06839.3	maker-VaccDscaff29-augustus-gene-338.24-mRN
C3H	AT5G07500.1	augustus_masked-VaccDscaff21-processed-gene
C3H	AT5G07500.1	augustus_masked-VaccDscaff33-processed-gene
C3H	AT5G07500.1	augustus_masked-VaccDscaff26-processed-gene
C3H	AT5G07500.1	augustus_masked-VaccDscaff29-processed-gene
ERF	AT5G07580.1	augustus_masked-VaccDscaff29-processed-gene
TCP	AT5G08330.1	augustus_masked-VaccDscaff11-processed-gene
TCP	AT5G08330.1	augustus_masked-VaccDscaff19-processed-gene
TCP	AT5G08330.1	augustus_masked-VaccDscaff15-processed-gene
TCP	AT5G08330.1	augustus_masked-VaccDscaff24-processed-gene
TCP	AT5G08330.1	augustus_masked-VaccDscaff24-processed-gene
TCP	AT5G08330.1	snap_masked-VaccDscaff24-processed-gene-103
MYB	AT5G08520.1	maker-VaccDscaff12-augustus-gene-367.27-mRN
MYB	AT5G08520.1	maker-VaccDscaff36-augustus-gene-211.31-mRN
MYB	AT5G08520.1	snap_masked-VaccDscaff7-processed-gene-286.
MYB	AT5G08520.1	maker-VaccDscaff31-snap-gene-59.30-mRNA-1

MYB	AT5G08520.1	maker-VaccDscaff40-augustus-gene-267.47-mRN
MYB	AT5G08520.1	maker-VaccDscaff41-augustus-gene-250.26-mRN
MYB	AT5G08520.1	maker-VaccDscaff9-augustus-gene-205.32-mRN
MYB	AT5G08520.1	maker-VaccDscaff35-augustus-gene-146.23-mRN
MYB	AT5G08520.1	maker-VaccDscaff4-augustus-gene-245.25-mRN
MYB	AT5G08520.1	snap_masked-VaccDscaff23-processed-gene-47.
MYB	AT5G08520.1	snap_masked-VaccDscaff23-processed-gene-48.
NAC	AT5G08790.1	maker-VaccDscaff21-snap-gene-347.27-mRNA-1
NAC	AT5G08790.1	augustus_masked-VaccDscaff33-processed-gene
NAC	AT5G08790.1	maker-VaccDscaff26-augustus-gene-339.13-mRN
NAC	AT5G08790.1	maker-VaccDscaff5-snap-gene-270.29-mRNA-1
NAC	AT5G08790.1	maker-VaccDscaff23-snap-gene-268.26-mRNA-1
NAC	AT5G08790.1	maker-VaccDscaff29-snap-gene-324.36-mRNA-1
C2H2	AT5G10970.1	augustus_masked-VaccDscaff32-processed-gene
C2H2	AT5G10970.1	augustus_masked-VaccDscaff30-processed-gene
C2H2	AT5G10970.1	maker-VaccDscaff30-snap-gene-250.26-mRNA-1
C2H2	AT5G10970.1	augustus_masked-VaccDscaff30-processed-gene
bZIP	AT5G11260.1	maker-VaccDscaff20-snap-gene-279.31-mRNA-1
bZIP	AT5G11260.1	maker-VaccDscaff44-snap-gene-84.35-mRNA-1
bZIP	AT5G11260.1	maker-VaccDscaff28-snap-gene-90.32-mRNA-1
HB-other	AT5G11270.1	maker-VaccDscaff20-augustus-gene-277.24-mRN
HB-other	AT5G11270.1	maker-VaccDscaff20-snap-gene-279.32-mRNA-1
HB-other	AT5G11270.1	maker-VaccDscaff44-augustus-gene-85.35-mRN
HB-other	AT5G11270.1	maker-VaccDscaff28-augustus-gene-91.41-mRN
ERF	AT5G11590.1	augustus_masked-VaccDscaff27-processed-gene
ERF	AT5G11590.1	augustus_masked-VaccDscaff20-processed-gene
ERF	AT5G11590.1	augustus_masked-VaccDscaff36-processed-gene
ERF	AT5G11590.1	augustus_masked-VaccDscaff13-processed-gene
ERF	AT5G11590.1	augustus_masked-VaccDscaff139-processed-gen
ERF	AT5G11590.1	augustus_masked-VaccDscaff113-processed-gen
ERF	AT5G11590.1	augustus_masked-VaccDscaff7-processed-gene-
ERF	AT5G11590.1	augustus_masked-VaccDscaff31-processed-gene
ERF	AT5G11590.1	augustus_masked-VaccDscaff9-processed-gene-
ERF	AT5G11590.1	augustus_masked-VaccDscaff35-processed-gene
ERF	AT5G11590.1	augustus_masked-VaccDscaff34-processed-gene
ERF	AT5G11590.1	augustus_masked-VaccDscaff4-processed-gene-
ERF	AT5G11590.1	augustus_masked-VaccDscaff28-processed-gene
SRS	AT5G12330.4	maker-VaccDscaff11-augustus-gene-70.11-mRN
SRS	AT5G12330.4	maker-VaccDscaff19-augustus-gene-171.24-mRN
SRS	AT5G12330.4	snap_masked-VaccDscaff15-processed-gene-77.
SRS	AT5G12330.4	snap_masked-VaccDscaff24-processed-gene-34
WRKY	AT5G13080.1	maker-VaccDscaff36-augustus-gene-194.40-mRN
WRKY	AT5G13080.1	maker-VaccDscaff16-augustus-gene-7.31-mRNA-
WRKY	AT5G13080.1	maker-VaccDscaff9-augustus-gene-223.19-mRN
WRKY	AT5G13080.1	maker-VaccDscaff35-augustus-gene-162.12-mRN
WRKY	AT5G13080.1	maker-VaccDscaff4-augustus-gene-261.18-mRN

NAC	AT5G13180.1	augustus_masked-VaccDscaff27-processed-gene
NAC	AT5G13180.1	maker-VaccDscaff102-augustus-gene-4.41-mRN/
NAC	AT5G13180.1	maker-VaccDscaff36-augustus-gene-198.14-mRN
NAC	AT5G13180.1	maker-VaccDscaff2-augustus-gene-237.26-mRN/
NAC	AT5G13180.1	maker-VaccDscaff17-augustus-gene-312.30-mRN
NAC	AT5G13180.1	maker-VaccDscaff14-augustus-gene-197.27-mRN
NAC	AT5G13180.1	maker-VaccDscaff9-augustus-gene-218.37-mRN/
NAC	AT5G13180.1	maker-VaccDscaff50-augustus-gene-10.32-mRN/
NAC	AT5G13180.1	maker-VaccDscaff35-augustus-gene-158.11-mRN
NAC	AT5G13180.1	snap_masked-VaccDscaff34-processed-gene-68.
NAC	AT5G13180.1	maker-VaccDscaff4-augustus-gene-256.12-mRN/
NAC	AT5G13180.1	maker-VaccDscaff3-augustus-gene-201.21-mRN/
ERF	AT5G13330.1	augustus_masked-VaccDscaff294-processed-gen
ERF	AT5G13330.1	augustus_masked-VaccDscaff32-processed-gene
ERF	AT5G13330.1	snap_masked-VaccDscaff30-processed-gene-23
MIKC_MADS	AT5G13790.1	maker-VaccDscaff1545-snap-gene-0.5-mRNA-1
ERF	AT5G13910.1	augustus_masked-VaccDscaff2-processed-gene-
ERF	AT5G13910.1	augustus_masked-VaccDscaff14-processed-gene
ERF	AT5G13910.1	augustus_masked-VaccDscaff72-processed-gene
ERF	AT5G13910.1	augustus_masked-VaccDscaff3-processed-gene-
C2H2	AT5G14140.1	maker-VaccDscaff13-augustus-gene-52.34-mRN/
C2H2	AT5G14140.1	maker-VaccDscaff13-augustus-gene-52.35-mRN/
C2H2	AT5G14140.1	maker-VaccDscaff32-augustus-gene-311.42-mRN
C2H2	AT5G14140.1	maker-VaccDscaff32-augustus-gene-311.41-mRN
C2H2	AT5G14140.1	maker-VaccDscaff30-augustus-gene-308.39-mRN
C2H2	AT5G14140.1	maker-VaccDscaff42-augustus-gene-9.37-mRNA-
C2H2	AT5G14140.1	maker-VaccDscaff42-snap-gene-9.48-mRNA-1
MYB	AT5G14340.1	maker-VaccDscaff16-augustus-gene-28.43-mRN/
MYB	AT5G14340.1	maker-VaccDscaff16-augustus-gene-29.27-mRN/
MYB	AT5G14340.1	maker-VaccDscaff7-augustus-gene-403.31-mRN/
MYB	AT5G14340.1	snap_masked-VaccDscaff7-processed-gene-403.
MYB	AT5G14340.1	maker-VaccDscaff18-augustus-gene-378.19-mRN
MYB	AT5G14340.1	maker-VaccDscaff18-snap-gene-378.20-mRNA-1
MYB	AT5G14750.1	maker-VaccDscaff27-augustus-gene-251.19-mRN
MYB	AT5G14750.1	maker-VaccDscaff17-augustus-gene-277.14-mRN
MYB	AT5G14750.1	maker-VaccDscaff34-augustus-gene-101.26-mRN
MYB	AT5G14750.1	maker-VaccDscaff4-augustus-gene-367.24-mRN/
MYB	AT5G14750.1	maker-VaccDscaff28-augustus-gene-122.42-mRN
ZF-HD	AT5G15210.1	augustus_masked-VaccDscaff16-processed-gene
ZF-HD	AT5G15210.1	augustus_masked-VaccDscaff7-processed-gene-
ZF-HD	AT5G15210.1	augustus_masked-VaccDscaff18-processed-gene
MYB	AT5G15310.1	maker-VaccDscaff16-snap-gene-66.36-mRNA-1
MYB	AT5G15310.1	maker-VaccDscaff13-augustus-gene-80.14-mRN/
MYB	AT5G15310.1	maker-VaccDscaff7-snap-gene-358.35-mRNA-1
MYB	AT5G15310.1	maker-VaccDscaff18-snap-gene-329.50-mRNA-1
MYB	AT5G15310.1	maker-VaccDscaff30-augustus-gene-325.32-mRN

MYB	AT5G15310.2	maker-VaccDscaff2-snap-gene-283.28-mRNA-1
MYB	AT5G15310.2	maker-VaccDscaff3-snap-gene-141.26-mRNA-1
MIKC_MADS	AT5G15800.1	maker-VaccDscaff36-augustus-gene-95.18-mRN/
MIKC_MADS	AT5G15800.1	maker-VaccDscaff35-augustus-gene-254.29-mRN
bZIP	AT5G15830.1	augustus_masked-VaccDscaff41-processed-gene
G2-like	AT5G16560.1	snap_masked-VaccDscaff41-processed-gene-24(
G2-like	AT5G16560.1	maker-VaccDscaff23-augustus-gene-40.19-mRN/
G2-like	AT5G16560.1	augustus_masked-VaccDscaff23-processed-gene
MYB	AT5G16600.1	snap_masked-VaccDscaff38-processed-gene-55.
MYB	AT5G16600.1	maker-VaccDscaff13-augustus-gene-22.24-mRN/
MYB	AT5G16600.1	maker-VaccDscaff6-augustus-gene-368.19-mRN/
MYB	AT5G16600.1	maker-VaccDscaff37-augustus-gene-258.25-mRN
MYB	AT5G16600.1	maker-VaccDscaff42-augustus-gene-38.17-mRN/
HSF	AT5G16820.2	maker-VaccDscaff20-augustus-gene-1.32-mRNA-
HSF	AT5G16820.2	maker-VaccDscaff19-augustus-gene-1.29-mRNA-
HSF	AT5G16820.2	maker-VaccDscaff28-augustus-gene-349.41-mRN
HSF	AT5G16820.2	maker-VaccDscaff48-augustus-gene-108.44-mRN
MYB_related	AT5G17300.1	maker-VaccDscaff12-snap-gene-362.52-mRNA-1
MYB_related	AT5G17300.1	maker-VaccDscaff40-augustus-gene-261.27-mRN
MYB_related	AT5G17300.1	snap_masked-VaccDscaff41-processed-gene-24(
MYB	AT5G17800.1	maker-VaccDscaff21-augustus-gene-307.25-mRN
MYB	AT5G17800.1	maker-VaccDscaff26-augustus-gene-299.26-mRN
WOX	AT5G17810.2	augustus_masked-VaccDscaff8-processed-gene-:
WOX	AT5G17810.2	maker-VaccDscaff1-augustus-gene-177.18-mRN/
G2-like	AT5G18240.4	maker-VaccDscaff14-snap-gene-37.40-mRNA-1
NAC	AT5G18270.1	snap_masked-VaccDscaff10-processed-gene-11!
NAC	AT5G18270.1	maker-VaccDscaff5-augustus-gene-293.26-mRN/
NAC	AT5G18270.1	snap_masked-VaccDscaff1-processed-gene-315.
ERF	AT5G18450.1	augustus_masked-VaccDscaff27-processed-gene
ERF	AT5G18450.1	augustus_masked-VaccDscaff45-processed-gene
ERF	AT5G18450.1	augustus_masked-VaccDscaff34-processed-gene
NF-YC	AT5G19490.1	maker-VaccDscaff15-augustus-gene-89.28-mRN/
NF-YC	AT5G19490.1	maker-VaccDscaff24-augustus-gene-356.25-mRN
ERF	AT5G19790.1	augustus_masked-VaccDscaff881-processed-gen
ERF	AT5G19790.1	augustus_masked-VaccDscaff30-processed-gene
MIKC_MADS	AT5G20240.1	maker-VaccDscaff25-augustus-gene-207.33-mRN
MIKC_MADS	AT5G20240.1	maker-VaccDscaff2-augustus-gene-203.29-mRN/
MIKC_MADS	AT5G20240.1	maker-VaccDscaff14-augustus-gene-156.26-mRN
MIKC_MADS	AT5G20240.1	maker-VaccDscaff14-augustus-gene-156.26-mRN
MIKC_MADS	AT5G20240.1	maker-VaccDscaff3-augustus-gene-240.31-mRN/
ARF	AT5G20730.1	augustus_masked-VaccDscaff23-processed-gene
ARF	AT5G20730.1	augustus_masked-VaccDscaff23-processed-gene
ARF	AT5G20730.1	augustus_masked-VaccDscaff23-processed-gene
ARF	AT5G20730.2	augustus_masked-VaccDscaff41-processed-gene
ARF	AT5G20730.2	maker-VaccDscaff23-augustus-gene-72.31-mRN/
ARF	AT5G20730.2	maker-VaccDscaff23-augustus-gene-72.31-mRN/

ARF	AT5G20730.3	augustus_masked-VaccDscaff12-processed-gene
ARF	AT5G20730.3	augustus_masked-VaccDscaff12-processed-gene
ARF	AT5G20730.3	augustus_masked-VaccDscaff12-processed-gene
ARF	AT5G20730.3	augustus_masked-VaccDscaff12-processed-gene
ARF	AT5G20730.3	maker-VaccDscaff41-snap-gene-199.27-mRNA-1
ERF	AT5G21960.1	augustus_masked-VaccDscaff21-processed-gene
ERF	AT5G21960.1	augustus_masked-VaccDscaff33-processed-gene
ERF	AT5G21960.1	augustus_masked-VaccDscaff26-processed-gene
ERF	AT5G21960.1	augustus_masked-VaccDscaff29-processed-gene
E2F/DP	AT5G22220.2	augustus_masked-VaccDscaff22-processed-gene
E2F/DP	AT5G22220.2	maker-VaccDscaff46-augustus-gene-110.29-mRN
E2F/DP	AT5G22220.2	maker-VaccDscaff47-snap-gene-44.29-mRNA-1
E2F/DP	AT5G22220.2	maker-VaccDscaff47-snap-gene-49.36-mRNA-1
NAC	AT5G22380.1	maker-VaccDscaff21-augustus-gene-272.23-mRN
NAC	AT5G22380.1	maker-VaccDscaff21-snap-gene-272.31-mRNA-1
NAC	AT5G22380.1	maker-VaccDscaff21-snap-gene-272.31-mRNA-1
NAC	AT5G22380.1	maker-VaccDscaff33-augustus-gene-63.17-mRNA
NAC	AT5G22380.1	maker-VaccDscaff33-snap-gene-64.31-mRNA-1
NAC	AT5G22380.1	maker-VaccDscaff33-snap-gene-64.31-mRNA-1
NAC	AT5G22380.1	augustus_masked-VaccDscaff33-processed-gene
NAC	AT5G22380.1	augustus_masked-VaccDscaff131-processed-gen
NAC	AT5G22380.1	maker-VaccDscaff11-augustus-gene-280.28-mRN
NAC	AT5G22380.1	augustus_masked-VaccDscaff616-processed-gen
NAC	AT5G22380.1	maker-VaccDscaff26-augustus-gene-259.22-mRN
NAC	AT5G22380.1	maker-VaccDscaff26-snap-gene-260.30-mRNA-1
NAC	AT5G22380.1	maker-VaccDscaff26-snap-gene-260.30-mRNA-1
NAC	AT5G22380.1	maker-VaccDscaff19-augustus-gene-312.37-mRN
NAC	AT5G22380.1	maker-VaccDscaff15-augustus-gene-264.23-mRN
NAC	AT5G22380.1	maker-VaccDscaff1-augustus-gene-218.14-mRNA
NAC	AT5G22380.1	maker-VaccDscaff24-augustus-gene-120.21-mRN
NAC	AT5G22380.1	maker-VaccDscaff29-augustus-gene-248.29-mRN
NAC	AT5G22380.1	augustus_masked-VaccDscaff29-processed-gene
NAC	AT5G22380.1	maker-VaccDscaff29-augustus-gene-288.27-mRN
C2H2	AT5G22890.1	maker-VaccDscaff22-augustus-gene-249.38-mRN
C2H2	AT5G22890.1	maker-VaccDscaff43-augustus-gene-136.13-mRN
C2H2	AT5G22890.1	augustus_masked-VaccDscaff11-processed-gene
C2H2	AT5G22890.1	maker-VaccDscaff225-snap-gene-0.35-mRNA-1
C2H2	AT5G22890.1	augustus_masked-VaccDscaff19-processed-gene
C2H2	AT5G22890.1	augustus_masked-VaccDscaff15-processed-gene
C2H2	AT5G22890.1	augustus_masked-VaccDscaff24-processed-gene
MYB	AT5G23000.1	maker-VaccDscaff46-snap-gene-85.18-mRNA-1
MYB	AT5G23000.1	augustus_masked-VaccDscaff32-processed-gene
MYB	AT5G23000.1	maker-VaccDscaff47-augustus-gene-68.22-mRNA
NF-YB	AT5G23090.4	maker-VaccDscaff6-snap-gene-101.28-mRNA-1
NF-YB	AT5G23090.4	snap_masked-VaccDscaff37-processed-gene-23.
MIKC_MADS	AT5G23260.2	maker-VaccDscaff25-augustus-gene-208.27-mRN

MIKC_MADS	AT5G23260.3	maker-VaccDscaff2-augustus-gene-202.29-mRNA
TCP	AT5G23280.1	augustus_masked-VaccDscaff36-processed-gene
TCP	AT5G23280.1	augustus_masked-VaccDscaff9-processed-gene-
TCP	AT5G23280.1	augustus_masked-VaccDscaff35-processed-gene
TCP	AT5G23280.1	augustus_masked-VaccDscaff4-processed-gene-
bZIP	AT5G24800.1	maker-VaccDscaff7-augustus-gene-156.20-mRNA
bZIP	AT5G24800.1	maker-VaccDscaff31-augustus-gene-190.22-mRN
bZIP	AT5G24800.1	maker-VaccDscaff18-augustus-gene-157.17-mRN
ERF	AT5G25190.1	maker-VaccDscaff27-augustus-gene-295.35-mRN
ERF	AT5G25190.1	maker-VaccDscaff20-augustus-gene-302.16-mRN
ERF	AT5G25190.1	augustus_masked-VaccDscaff16-processed-gene
ERF	AT5G25190.1	maker-VaccDscaff7-augustus-gene-191.19-mRNA
ERF	AT5G25190.1	maker-VaccDscaff31-augustus-gene-166.12-mRN
ERF	AT5G25190.1	maker-VaccDscaff17-snap-gene-324.54-mRNA-1
ERF	AT5G25190.1	maker-VaccDscaff18-augustus-gene-177.16-mRN
ERF	AT5G25190.1	augustus_masked-VaccDscaff50-processed-gene
ERF	AT5G25190.1	maker-VaccDscaff44-augustus-gene-65.21-mRNA
ERF	AT5G25190.1	maker-VaccDscaff34-augustus-gene-49.37-mRNA
ERF	AT5G25190.1	maker-VaccDscaff28-augustus-gene-73.20-mRNA
TALE	AT5G25220.1	maker-VaccDscaff38-snap-gene-29.27-mRNA-1
TALE	AT5G25220.1	maker-VaccDscaff28-snap-gene-77.25-mRNA-1
TALE	AT5G25220.2	maker-VaccDscaff20-augustus-gene-297.24-mRN
TALE	AT5G25220.2	maker-VaccDscaff16-snap-gene-224.37-mRNA-1
TALE	AT5G25220.2	maker-VaccDscaff6-augustus-gene-386.22-mRNA
TALE	AT5G25220.2	maker-VaccDscaff7-augustus-gene-193.29-mRNA
TALE	AT5G25220.2	maker-VaccDscaff31-augustus-gene-165.22-mRN
TALE	AT5G25220.2	maker-VaccDscaff18-snap-gene-180.19-mRNA-1
TALE	AT5G25220.2	maker-VaccDscaff81-augustus-gene-3.30-mRNA-
TALE	AT5G25220.2	maker-VaccDscaff15-snap-gene-209.37-mRNA-1
TALE	AT5G25220.2	maker-VaccDscaff44-augustus-gene-69.23-mRNA
TALE	AT5G25220.2	maker-VaccDscaff75-augustus-gene-4.25-mRNA-
TALE	AT5G25220.2	maker-VaccDscaff39-augustus-gene-27.19-mRNA
TALE	AT5G25220.2	maker-VaccDscaff60-augustus-gene-9.42-mRNA-
ERF	AT5G25390.2	maker-VaccDscaff44-augustus-gene-82.17-mRNA
ERF	AT5G25810.1	augustus_masked-VaccDscaff33-processed-gene
ERF	AT5G25810.1	augustus_masked-VaccDscaff32-processed-gene
ERF	AT5G25810.1	maker-VaccDscaff30-snap-gene-72.24-mRNA-1
ERF	AT5G25810.1	maker-VaccDscaff30-snap-gene-72.24-mRNA-1
ERF	AT5G25810.1	augustus_masked-VaccDscaff28-processed-gene
GATA	AT5G25830.1	maker-VaccDscaff10-augustus-gene-117.28-mRN
GATA	AT5G25830.1	maker-VaccDscaff8-augustus-gene-140.33-mRNA
GATA	AT5G25830.1	maker-VaccDscaff20-augustus-gene-245.33-mRN
GATA	AT5G25830.1	maker-VaccDscaff139-augustus-gene-2.21-mRNA
GATA	AT5G25830.1	maker-VaccDscaff113-augustus-gene-3.44-mRNA
GATA	AT5G25830.1	snap_masked-VaccDscaff7-processed-gene-230.
GATA	AT5G25830.1	maker-VaccDscaff31-augustus-gene-116.27-mRN

GATA	AT5G25830.1	maker-VaccDscaff5-augustus-gene-291.25-mRNA
GATA	AT5G25830.1	maker-VaccDscaff1-augustus-gene-312.27-mRNA
GATA	AT5G25830.1	maker-VaccDscaff44-augustus-gene-114.29-mRN
GATA	AT5G25830.1	maker-VaccDscaff28-augustus-gene-112.36-mRN
MYB	AT5G26660.1	maker-VaccDscaff27-augustus-gene-259.25-mRN
MYB	AT5G26660.1	maker-VaccDscaff27-augustus-gene-356.33-mRN
MYB	AT5G26660.1	maker-VaccDscaff17-augustus-gene-286.24-mRN
MYB	AT5G26660.1	maker-VaccDscaff34-augustus-gene-92.35-mRNA
C3H	AT5G26749.1	maker-VaccDscaff27-augustus-gene-120.30-mRN
C3H	AT5G26749.1	maker-VaccDscaff45-augustus-gene-125.20-mRN
C3H	AT5G26749.1	maker-VaccDscaff34-snap-gene-190.38-mRNA-1
M-type_MADS	AT5G26880.1	maker-VaccDscaff12-snap-gene-159.29-mRNA-1
M-type_MADS	AT5G26880.1	maker-VaccDscaff40-snap-gene-123.26-mRNA-1
M-type_MADS	AT5G26880.1	maker-VaccDscaff41-snap-gene-110.26-mRNA-1
bZIP	AT5G28770.1	maker-VaccDscaff583-snap-gene-0.21-mRNA-1
MYB	AT5G35550.1	augustus_masked-VaccDscaff27-processed-gene
MYB	AT5G35550.1	maker-VaccDscaff17-augustus-gene-385.21-mRN
MYB	AT5G35550.1	maker-VaccDscaff32-augustus-gene-54.30-mRNA
MYB	AT5G35550.1	maker-VaccDscaff32-augustus-gene-55.27-mRNA
MYB	AT5G35550.1	maker-VaccDscaff30-snap-gene-17.57-mRNA-1
MYB	AT5G35550.1	maker-VaccDscaff34-augustus-gene-10.31-mRNA
MYB	AT5G35550.1	snap_masked-VaccDscaff42-processed-gene-21
SAP	AT5G35770.1	maker-VaccDscaff10-augustus-gene-34.28-mRNA
SAP	AT5G35770.1	maker-VaccDscaff8-augustus-gene-41.37-mRNA-
SAP	AT5G35770.1	maker-VaccDscaff5-augustus-gene-388.33-mRNA
SAP	AT5G35770.1	maker-VaccDscaff1-augustus-gene-408.36-mRNA
ARF	AT5G37020.1	maker-VaccDscaff13-snap-gene-29.49-mRNA-1
ARF	AT5G37020.1	maker-VaccDscaff11-snap-gene-273.28-mRNA-1
ARF	AT5G37020.1	maker-VaccDscaff40-snap-gene-269.42-mRNA-1
ARF	AT5G37020.1	maker-VaccDscaff41-snap-gene-251.35-mRNA-1
ARF	AT5G37020.1	maker-VaccDscaff19-augustus-gene-305.22-mRN
ARF	AT5G37020.1	maker-VaccDscaff15-snap-gene-258.34-mRNA-1
ARF	AT5G37020.1	maker-VaccDscaff30-augustus-gene-285.30-mRN
ARF	AT5G37020.1	maker-VaccDscaff24-augustus-gene-127.18-mRN
ARF	AT5G37020.1	maker-VaccDscaff42-augustus-gene-31.33-mRNA
ARF	AT5G37020.1	maker-VaccDscaff23-snap-gene-49.45-mRNA-1
NAC	AT5G39820.1	maker-VaccDscaff16-augustus-gene-60.41-mRNA
NAC	AT5G39820.1	maker-VaccDscaff18-augustus-gene-337.37-mRN
bHLH	AT5G39860.1	maker-VaccDscaff16-snap-gene-56.38-mRNA-1
MYB	AT5G40330.1	maker-VaccDscaff27-augustus-gene-338.29-mRN
MYB	AT5G40330.1	maker-VaccDscaff17-snap-gene-386.39-mRNA-1
MYB	AT5G40330.1	maker-VaccDscaff34-snap-gene-9.38-mRNA-1
MYB	AT5G40330.1	maker-VaccDscaff218-snap-gene-1.34-mRNA-1
MYB	AT5G40330.1	maker-VaccDscaff3-augustus-gene-434.14-mRNA
MYB	AT5G40330.1	maker-VaccDscaff91-augustus-gene-0.27-mRNA-
MYB	AT5G40350.1	snap_masked-VaccDscaff7-processed-gene-390.

MYB_related	AT5G41020.1	snap_masked-VaccDscaff132-processed-gene-2.
MYB_related	AT5G41020.1	snap_masked-VaccDscaff19-processed-gene-95.
MYB_related	AT5G41020.1	maker-VaccDscaff28-snap-gene-262.41-mRNA-1
MYB_related	AT5G41020.1	maker-VaccDscaff48-augustus-gene-21.23-mRN/
GRAS	AT5G41920.1	maker-VaccDscaff2-snap-gene-228.30-mRNA-1
GRAS	AT5G41920.1	maker-VaccDscaff14-augustus-gene-187.22-mRN
GRAS	AT5G41920.1	maker-VaccDscaff72-snap-gene-3.38-mRNA-1
GRAS	AT5G41920.1	maker-VaccDscaff3-augustus-gene-207.27-mRN/
BBR-BPC	AT5G42520.1	maker-VaccDscaff12-augustus-gene-393.19-mRN
BBR-BPC	AT5G42520.1	maker-VaccDscaff16-augustus-gene-378.18-mRN
BBR-BPC	AT5G42520.1	maker-VaccDscaff7-augustus-gene-46.25-mRNA-
BBR-BPC	AT5G42520.1	maker-VaccDscaff31-augustus-gene-322.28-mRN
BBR-BPC	AT5G42520.1	maker-VaccDscaff18-augustus-gene-18.14-mRN/
BBR-BPC	AT5G42520.1	augustus_masked-VaccDscaff23-processed-gene
BBR-BPC	AT5G42520.1	augustus_masked-VaccDscaff23-processed-gene
BBR-BPC	AT5G42520.2	augustus_masked-VaccDscaff31-processed-gene
BBR-BPC	AT5G42520.2	augustus_masked-VaccDscaff31-processed-gene
B3	AT5G42700.1	maker-VaccDscaff31-augustus-gene-339.25-mRN
B3	AT5G42700.1	maker-VaccDscaff18-augustus-gene-1.32-mRNA-
B3	AT5G42700.1	maker-VaccDscaff169-augustus-gene-2.44-mRN/
NF-YC	AT5G43250.1	snap_masked-VaccDscaff334-processed-gene-0.
NF-YC	AT5G43250.1	augustus_masked-VaccDscaff14-processed-gene
NF-YC	AT5G43250.1	augustus_masked-VaccDscaff463-processed-gen
NF-YC	AT5G43250.1	augustus_masked-VaccDscaff3-processed-gene-
ERF	AT5G44210.1	maker-VaccDscaff38-snap-gene-224.35-mRNA-1
ERF	AT5G44210.1	augustus_masked-VaccDscaff6-processed-gene-
ERF	AT5G44210.1	augustus_masked-VaccDscaff9-processed-gene-
ERF	AT5G44210.1	augustus_masked-VaccDscaff37-processed-gene
ERF	AT5G44210.1	augustus_masked-VaccDscaff35-processed-gene
ERF	AT5G44210.1	augustus_masked-VaccDscaff30-processed-gene
ERF	AT5G44210.1	augustus_masked-VaccDscaff42-processed-gene
ERF	AT5G44210.1	augustus_masked-VaccDscaff42-processed-gene
ERF	AT5G44210.1	augustus_masked-VaccDscaff4-processed-gene-
ERF	AT5G44210.1	augustus_masked-VaccDscaff39-processed-gene
BES1	AT5G45300.2	maker-VaccDscaff25-augustus-gene-3.30-mRNA-
BES1	AT5G45300.2	maker-VaccDscaff2-augustus-gene-27.28-mRNA-
BES1	AT5G45300.2	maker-VaccDscaff14-augustus-gene-16.20-mRN/
BES1	AT5G45300.2	maker-VaccDscaff3-augustus-gene-412.31-mRN/
ERF	AT5G47220.1	snap_masked-VaccDscaff20-processed-gene-19[illegible]
ERF	AT5G47220.1	augustus_masked-VaccDscaff46-processed-gene
ERF	AT5G47220.1	maker-VaccDscaff147-snap-gene-2.71-mRNA-1
ERF	AT5G47220.1	augustus_masked-VaccDscaff105-processed-gen
ERF	AT5G47220.1	augustus_masked-VaccDscaff3-processed-gene-
ERF	AT5G47220.1	maker-VaccDscaff3-snap-gene-430.39-mRNA-1
HD-ZIP	AT5G47370.1	maker-VaccDscaff25-augustus-gene-24.30-mRN/
HD-ZIP	AT5G47370.1	maker-VaccDscaff247-augustus-gene-0.47-mRN/

HD-ZIP	AT5G47370.1	maker-VaccDscaff3-augustus-gene-432.28-mRN/
HD-ZIP	AT5G47370.1	maker-VaccDscaff197-augustus-gene-0.20-mRN/
MYB_related	AT5G47390.1	maker-VaccDscaff25-augustus-gene-24.29-mRN/
MYB_related	AT5G47390.1	maker-VaccDscaff247-augustus-gene-0.53-mRN/
MYB_related	AT5G47390.1	maker-VaccDscaff9-snap-gene-7.48-mRNA-1
MYB_related	AT5G47390.1	snap_masked-VaccDscaff42-processed-gene-233
MYB_related	AT5G47390.1	maker-VaccDscaff3-augustus-gene-433.36-mRN/
MYB_related	AT5G47390.1	maker-VaccDscaff197-augustus-gene-1.49-mRN/
CO-like	AT5G48250.1	snap_masked-VaccDscaff38-processed-gene-283
M-type_MADS	AT5G48670.1	snap_masked-VaccDscaff22-processed-gene-65.
M-type_MADS	AT5G48670.1	augustus_masked-VaccDscaff22-processed-gene
M-type_MADS	AT5G48670.1	snap_masked-VaccDscaff43-processed-gene-140
M-type_MADS	AT5G48670.1	maker-VaccDscaff43-snap-gene-255.37-mRNA-1
M-type_MADS	AT5G48670.1	maker-VaccDscaff43-snap-gene-255.37-mRNA-1
M-type_MADS	AT5G48670.1	augustus_masked-VaccDscaff21-processed-gene
M-type_MADS	AT5G48670.1	augustus_masked-VaccDscaff25-processed-gene
M-type_MADS	AT5G48670.1	augustus_masked-VaccDscaff33-processed-gene
M-type_MADS	AT5G48670.1	maker-VaccDscaff11-augustus-gene-42.13-mRN/
M-type_MADS	AT5G48670.1	snap_masked-VaccDscaff2-processed-gene-341.
M-type_MADS	AT5G48670.1	augustus_masked-VaccDscaff26-processed-gene
M-type_MADS	AT5G48670.1	augustus_masked-VaccDscaff14-processed-gene
M-type_MADS	AT5G48670.1	augustus_masked-VaccDscaff29-processed-gene
M-type_MADS	AT5G48670.1	augustus_masked-VaccDscaff3-processed-gene-(
M-type_MADS	AT5G48670.1	snap_masked-VaccDscaff47-processed-gene-59.
MYB	AT5G49330.1	maker-VaccDscaff22-augustus-gene-367.35-mRN
MYB	AT5G49330.1	maker-VaccDscaff43-snap-gene-6.43-mRNA-1
MYB	AT5G49330.1	maker-VaccDscaff46-augustus-gene-203.39-mRN
MYB	AT5G49330.1	maker-VaccDscaff30-augustus-gene-18.33-mRN/
MYB	AT5G49330.1	maker-VaccDscaff42-augustus-gene-218.38-mRN
MYB	AT5G49330.1	maker-VaccDscaff47-augustus-gene-192.25-mRN
WRKY	AT5G49520.1	maker-VaccDscaff10-augustus-gene-390.33-mRN
WRKY	AT5G49520.1	maker-VaccDscaff8-augustus-gene-402.23-mRN/
WRKY	AT5G49520.1	maker-VaccDscaff5-augustus-gene-11.18-mRNA-
WRKY	AT5G49520.1	maker-VaccDscaff1-augustus-gene-12.29-mRNA-
MYB	AT5G49620.1	maker-VaccDscaff10-augustus-gene-393.29-mRN
MYB	AT5G49620.1	augustus_masked-VaccDscaff5-processed-gene-8
MYB	AT5G49620.1	augustus_masked-VaccDscaff1-processed-gene-8
ERF	AT5G51190.1	augustus_masked-VaccDscaff22-processed-gene
ERF	AT5G51190.1	maker-VaccDscaff43-snap-gene-192.53-mRNA-1
ERF	AT5G51190.1	augustus_masked-VaccDscaff43-processed-gene
ERF	AT5G51190.1	snap_masked-VaccDscaff25-processed-gene-22.
ERF	AT5G51190.1	augustus_masked-VaccDscaff46-processed-gene
ERF	AT5G51190.1	augustus_masked-VaccDscaff19-processed-gene
ERF	AT5G51190.1	augustus_masked-VaccDscaff147-processed-gen
ERF	AT5G51190.1	augustus_masked-VaccDscaff105-processed-gen
ERF	AT5G51190.1	augustus_masked-VaccDscaff3-processed-gene-4

ERF	AT5G51190.1	maker-VaccDscaff28-snap-gene-328.35-mRNA-1
MIKC_MADS	AT5G51870.2	snap_masked-VaccDscaff539-processed-gene-0.
TCP	AT5G51910.2	maker-VaccDscaff33-snap-gene-264.40-mRNA-1
TCP	AT5G51910.2	augustus_masked-VaccDscaff11-processed-gene
TCP	AT5G51910.2	augustus_masked-VaccDscaff26-processed-gene
TCP	AT5G51910.2	augustus_masked-VaccDscaff19-processed-gene
C3H	AT5G51980.2	maker-VaccDscaff22-augustus-gene-29.21-mRN/
ERF	AT5G51990.1	snap_masked-VaccDscaff22-processed-gene-243
ERF	AT5G51990.1	augustus_masked-VaccDscaff31-processed-gene
C2H2	AT5G52010.1	snap_masked-VaccDscaff40-processed-gene-28.
C2H2	AT5G52010.1	maker-VaccDscaff41-snap-gene-42.38-mRNA-1
C2H2	AT5G52010.1	augustus_masked-VaccDscaff23-processed-gene
ERF	AT5G52020.1	augustus_masked-VaccDscaff22-processed-gene
ERF	AT5G52020.1	augustus_masked-VaccDscaff22-processed-gene
ERF	AT5G52020.1	augustus_masked-VaccDscaff43-processed-gene
ERF	AT5G52020.1	augustus_masked-VaccDscaff43-processed-gene
ERF	AT5G52020.1	augustus_masked-VaccDscaff21-processed-gene
ERF	AT5G52020.1	maker-VaccDscaff20-snap-gene-22.35-mRNA-1
ERF	AT5G52020.1	augustus_masked-VaccDscaff33-processed-gene
ERF	AT5G52020.1	augustus_masked-VaccDscaff26-processed-gene
ERF	AT5G52020.1	augustus_masked-VaccDscaff46-processed-gene
ERF	AT5G52020.1	augustus_masked-VaccDscaff46-processed-gene
ERF	AT5G52020.1	augustus_masked-VaccDscaff19-processed-gene
ERF	AT5G52020.1	augustus_masked-VaccDscaff19-processed-gene
ERF	AT5G52020.1	augustus_masked-VaccDscaff29-processed-gene
ERF	AT5G52020.1	augustus_masked-VaccDscaff28-processed-gene
ERF	AT5G52020.1	augustus_masked-VaccDscaff28-processed-gene
ERF	AT5G52020.1	augustus_masked-VaccDscaff48-processed-gene
ERF	AT5G52020.1	augustus_masked-VaccDscaff48-processed-gene
MYB	AT5G52260.1	maker-VaccDscaff21-augustus-gene-63.29-mRN/
MYB	AT5G52260.1	maker-VaccDscaff33-augustus-gene-273.22-mRN
MYB	AT5G52260.1	maker-VaccDscaff26-augustus-gene-63.27-mRN/
MYB	AT5G52260.1	maker-VaccDscaff29-augustus-gene-64.36-mRN/
MYB	AT5G52600.1	maker-VaccDscaff155-augustus-gene-2.52-mRN/
MYB	AT5G52600.1	maker-VaccDscaff65-augustus-gene-8.53-mRNA-
MYB	AT5G52600.1	maker-VaccDscaff146-snap-gene-0.30-mRNA-1
MYB	AT5G52600.1	maker-VaccDscaff60-augustus-gene-6.21-mRNA-
MYB_related	AT5G52660.2	maker-VaccDscaff22-augustus-gene-16.27-mRN/
MYB_related	AT5G52660.2	maker-VaccDscaff21-snap-gene-51.43-mRNA-1
MYB_related	AT5G52660.2	augustus_masked-VaccDscaff20-processed-gene
MYB_related	AT5G52660.2	maker-VaccDscaff33-augustus-gene-283.30-mRN
MYB_related	AT5G52660.2	maker-VaccDscaff26-augustus-gene-52.27-mRN/
MYB_related	AT5G52660.2	maker-VaccDscaff28-augustus-gene-335.35-mRN
MYB_related	AT5G52660.2	maker-VaccDscaff48-augustus-gene-95.42-mRN/
Nin-like	AT5G53040.1	snap_masked-VaccDscaff21-processed-gene-35.
Nin-like	AT5G53040.1	maker-VaccDscaff26-augustus-gene-35.29-mRN/

Nin-like	AT5G53040.1	maker-VaccDscaff29-augustus-gene-35.39-mRN/
MYB_related	AT5G53200.1	maker-VaccDscaff22-augustus-gene-7.21-mRNA-
MYB_related	AT5G53200.1	maker-VaccDscaff12-snap-gene-301.25-mRNA-1
MYB_related	AT5G53200.1	maker-VaccDscaff40-snap-gene-195.24-mRNA-1
MYB_related	AT5G53200.1	maker-VaccDscaff41-snap-gene-184.30-mRNA-1
MYB_related	AT5G53200.1	maker-VaccDscaff23-snap-gene-90.17-mRNA-1
bHLH	AT5G53210.1	maker-VaccDscaff20-augustus-gene-12.26-mRN/
bHLH	AT5G53210.1	maker-VaccDscaff19-augustus-gene-10.34-mRN/
bHLH	AT5G53210.1	maker-VaccDscaff28-augustus-gene-339.17-mRN
bHLH	AT5G53210.1	maker-VaccDscaff48-augustus-gene-99.24-mRN/
NAC	AT5G53950.1	maker-VaccDscaff10-augustus-gene-26.21-mRN/
NAC	AT5G53950.1	maker-VaccDscaff8-augustus-gene-34.32-mRNA-
NAC	AT5G53950.1	maker-VaccDscaff5-augustus-gene-395.28-mRN/
NAC	AT5G53950.1	maker-VaccDscaff1-augustus-gene-416.30-mRN/
bHLH	AT5G54680.1	maker-VaccDscaff10-augustus-gene-12.36-mRN/
bHLH	AT5G54680.1	maker-VaccDscaff10-snap-gene-55.41-mRNA-1
bHLH	AT5G54680.1	maker-VaccDscaff8-augustus-gene-19.34-mRNA-
bHLH	AT5G54680.1	snap_masked-VaccDscaff38-processed-gene-24(
bHLH	AT5G54680.1	maker-VaccDscaff11-snap-gene-190.21-mRNA-1
bHLH	AT5G54680.1	maker-VaccDscaff6-augustus-gene-50.33-mRNA-
bHLH	AT5G54680.1	maker-VaccDscaff6-augustus-gene-109.27-mRN/
bHLH	AT5G54680.1	maker-VaccDscaff662-augustus-gene-0.10-mRN/
bHLH	AT5G54680.1	maker-VaccDscaff5-augustus-gene-339.34-mRN/
bHLH	AT5G54680.1	maker-VaccDscaff5-augustus-gene-426.39-mRN/
bHLH	AT5G54680.1	maker-VaccDscaff19-snap-gene-257.25-mRNA-1
bHLH	AT5G54680.1	maker-VaccDscaff15-snap-gene-178.26-mRNA-1
bHLH	AT5G54680.1	maker-VaccDscaff37-snap-gene-32.32-mRNA-1
bHLH	AT5G54680.1	maker-VaccDscaff1-augustus-gene-360.23-mRN/
bHLH	AT5G54680.1	maker-VaccDscaff24-snap-gene-200.42-mRNA-1
bHLH	AT5G54680.1	maker-VaccDscaff52-augustus-gene-17.27-mRN/
MYB	AT5G56110.1	augustus_masked-VaccDscaff62-processed-gene
MYB	AT5G56110.1	augustus_masked-VaccDscaff207-processed-gen
MYB	AT5G56110.1	maker-VaccDscaff301-augustus-gene-0.41-mRN/
MYB	AT5G56110.1	maker-VaccDscaff1-augustus-gene-459.31-mRN/
WRKY	AT5G56270.1	maker-VaccDscaff62-augustus-gene-7.31-mRNA-
WRKY	AT5G56270.1	maker-VaccDscaff207-augustus-gene-0.32-mRN/
WRKY	AT5G56270.1	maker-VaccDscaff1-augustus-gene-461.71-mRN/
WRKY	AT5G56270.1	maker-VaccDscaff78-augustus-gene-6.60-mRNA-
MYB_related	AT5G56840.1	maker-VaccDscaff25-augustus-gene-10.27-mRN/
MYB_related	AT5G56840.1	maker-VaccDscaff2-augustus-gene-21.26-mRNA-
MYB_related	AT5G56840.1	maker-VaccDscaff2-snap-gene-21.35-mRNA-1
MYB_related	AT5G56840.1	augustus_masked-VaccDscaff14-processed-gene
MYB_related	AT5G56840.1	maker-VaccDscaff42-augustus-gene-242.37-mRN
MYB_related	AT5G56840.1	maker-VaccDscaff3-augustus-gene-420.18-mRN/
bHLH	AT5G56960.1	maker-VaccDscaff25-augustus-gene-353.25-mRN
bHLH	AT5G56960.1	maker-VaccDscaff25-augustus-gene-353.25-mRN

bHLH	AT5G57150.1	maker-VaccDscaff56-augustus-gene-11.67-mRNA
bHLH	AT5G57150.1	maker-VaccDscaff55-augustus-gene-0.27-mRNA-
bHLH	AT5G57150.1	maker-VaccDscaff99-augustus-gene-4.57-mRNA-
bHLH	AT5G57150.1	maker-VaccDscaff95-snap-gene-2.33-mRNA-1
AP2	AT5G57390.1	maker-VaccDscaff160-augustus-gene-0.19-mRNA
AP2	AT5G57390.1	maker-VaccDscaff37-augustus-gene-303.52-mRN
AP2	AT5G57390.1	maker-VaccDscaff6-augustus-gene-423.24-mRNA
C2H2	AT5G57520.1	augustus_masked-VaccDscaff56-processed-gene
C2H2	AT5G57520.1	augustus_masked-VaccDscaff55-processed-gene
C2H2	AT5G57520.1	augustus_masked-VaccDscaff6-processed-gene-
C2H2	AT5G57520.1	snap_masked-VaccDscaff160-processed-gene-2.
C2H2	AT5G57520.1	augustus_masked-VaccDscaff61-processed-gene
C2H2	AT5G57520.1	augustus_masked-VaccDscaff37-processed-gene
C2H2	AT5G57520.1	augustus_masked-VaccDscaff70-processed-gene
MYB	AT5G57620.1	maker-VaccDscaff22-augustus-gene-252.30-mRN
MYB	AT5G57620.1	maker-VaccDscaff22-augustus-gene-252.29-mRN
MYB	AT5G57620.1	maker-VaccDscaff22-augustus-gene-253.17-mRN
MYB	AT5G57620.1	augustus_masked-VaccDscaff38-processed-gene
MYB	AT5G57620.1	maker-VaccDscaff43-augustus-gene-133.16-mRN
MYB	AT5G57620.1	maker-VaccDscaff43-augustus-gene-133.14-mRN
MYB	AT5G57620.1	maker-VaccDscaff21-augustus-gene-231.19-mRN
MYB	AT5G57620.1	maker-VaccDscaff25-snap-gene-14.33-mRNA-1
MYB	AT5G57620.1	maker-VaccDscaff33-augustus-gene-124.25-mRN
MYB	AT5G57620.1	maker-VaccDscaff56-augustus-gene-8.40-mRNA-
MYB	AT5G57620.1	maker-VaccDscaff11-augustus-gene-296.21-mRN
MYB	AT5G57620.1	maker-VaccDscaff55-augustus-gene-9.33-mRNA-
MYB	AT5G57620.1	maker-VaccDscaff6-augustus-gene-420.37-mRNA
MYB	AT5G57620.1	maker-VaccDscaff26-snap-gene-228.31-mRNA-1
MYB	AT5G57620.1	maker-VaccDscaff46-augustus-gene-85.16-mRNA
MYB	AT5G57620.1	maker-VaccDscaff19-snap-gene-327.44-mRNA-1
MYB	AT5G57620.1	maker-VaccDscaff15-augustus-gene-280.28-mRN
MYB	AT5G57620.1	maker-VaccDscaff61-snap-gene-8.91-mRNA-1
MYB	AT5G57620.1	maker-VaccDscaff37-augustus-gene-301.27-mRN
MYB	AT5G57620.1	maker-VaccDscaff37-augustus-gene-301.28-mRN
MYB	AT5G57620.1	snap_masked-VaccDscaff24-processed-gene-15.
MYB	AT5G57620.1	maker-VaccDscaff24-augustus-gene-104.18-mRN
MYB	AT5G57620.1	maker-VaccDscaff39-augustus-gene-1.24-mRNA-
MYB	AT5G57620.1	maker-VaccDscaff29-augustus-gene-219.25-mRN
MYB	AT5G57620.1	maker-VaccDscaff70-augustus-gene-1.23-mRNA-
MYB	AT5G57620.1	maker-VaccDscaff47-augustus-gene-68.23-mRNA
C3H	AT5G58620.1	augustus_masked-VaccDscaff11-processed-gene
C3H	AT5G58620.1	augustus_masked-VaccDscaff19-processed-gene
C3H	AT5G58620.1	augustus_masked-VaccDscaff15-processed-gene
C3H	AT5G58620.1	maker-VaccDscaff24-snap-gene-69.36-mRNA-1
MYB_related	AT5G58900.1	maker-VaccDscaff22-augustus-gene-291.32-mRN
MYB_related	AT5G58900.1	maker-VaccDscaff43-augustus-gene-98.24-mRNA

MYB_related	AT5G58900.1	maker-VaccDscaff46-augustus-gene-143.24-mRN
MYB_related	AT5G58900.1	maker-VaccDscaff19-augustus-gene-360.19-mRN
MYB_related	AT5G58900.1	maker-VaccDscaff15-augustus-gene-332.31-mRN
MYB_related	AT5G58900.1	maker-VaccDscaff24-augustus-gene-73.38-mRN/
MYB_related	AT5G58900.1	maker-VaccDscaff47-augustus-gene-108.27-mRN
WOX	AT5G59340.1	maker-VaccDscaff22-snap-gene-275.33-mRNA-1
WOX	AT5G59340.1	maker-VaccDscaff22-snap-gene-275.33-mRNA-1
WOX	AT5G59340.1	maker-VaccDscaff47-augustus-gene-92.27-mRN/
C2H2	AT5G59820.1	augustus_masked-VaccDscaff13-processed-gene
Dof	AT5G60200.1	maker-VaccDscaff11-snap-gene-158.12-mRNA-1
Dof	AT5G60200.1	snap_masked-VaccDscaff11-processed-gene-16[illegible]
Dof	AT5G60200.1	snap_masked-VaccDscaff19-processed-gene-23[illegible]
Dof	AT5G60200.1	maker-VaccDscaff19-snap-gene-338.49-mRNA-1
Dof	AT5G60200.1	snap_masked-VaccDscaff15-processed-gene-14[illegible]
Dof	AT5G60200.1	maker-VaccDscaff35-augustus-gene-107.20-mRN
Dof	AT5G60200.1	maker-VaccDscaff24-snap-gene-94.38-mRNA-1
Dof	AT5G60200.1	maker-VaccDscaff24-snap-gene-239.21-mRNA-1
M-type_MADS	AT5G60440.1	maker-VaccDscaff13-augustus-gene-373.17-mRN
M-type_MADS	AT5G60440.1	augustus_masked-VaccDscaff2-processed-gene-[illegible]
M-type_MADS	AT5G60440.1	augustus_masked-VaccDscaff14-processed-gene
M-type_MADS	AT5G60440.1	maker-VaccDscaff14-snap-gene-343.36-mRNA-1
M-type_MADS	AT5G60440.1	maker-VaccDscaff14-snap-gene-343.36-mRNA-1
M-type_MADS	AT5G60440.1	snap_masked-VaccDscaff50-processed-gene-10.
M-type_MADS	AT5G60440.1	maker-VaccDscaff30-augustus-gene-25.28-mRN/
M-type_MADS	AT5G60440.1	maker-VaccDscaff542-snap-gene-0.19-mRNA-1
M-type_MADS	AT5G60440.1	maker-VaccDscaff542-snap-gene-0.19-mRNA-1
M-type_MADS	AT5G60440.1	maker-VaccDscaff42-augustus-gene-224.33-mRN
M-type_MADS	AT5G60440.1	maker-VaccDscaff3-augustus-gene-15.34-mRNA-
M-type_MADS	AT5G60440.1	augustus_masked-VaccDscaff3-processed-gene-[illegible]
HD-ZIP	AT5G60690.1	maker-VaccDscaff22-augustus-gene-261.28-mRN
HD-ZIP	AT5G60690.1	maker-VaccDscaff43-augustus-gene-126.21-mRN
HD-ZIP	AT5G60690.1	maker-VaccDscaff11-snap-gene-302.24-mRNA-1
HD-ZIP	AT5G60690.1	maker-VaccDscaff86-snap-gene-4.44-mRNA-1
HD-ZIP	AT5G60690.1	maker-VaccDscaff46-augustus-gene-93.28-mRN/
HD-ZIP	AT5G60690.1	maker-VaccDscaff18-snap-gene-230.23-mRNA-1
HD-ZIP	AT5G60690.1	snap_masked-VaccDscaff15-processed-gene-28[illegible]
HD-ZIP	AT5G60690.1	maker-VaccDscaff15-snap-gene-286.26-mRNA-1
HD-ZIP	AT5G60690.1	maker-VaccDscaff47-augustus-gene-75.30-mRN/
Dof	AT5G60850.1	augustus_masked-VaccDscaff24-processed-gene
Dof	AT5G60850.1	augustus_masked-VaccDscaff262-processed-gen
MIKC_MADS	AT5G60910.1	maker-VaccDscaff13-snap-gene-88.16-mRNA-1
MIKC_MADS	AT5G60910.1	maker-VaccDscaff13-augustus-gene-229.42-mRN
MIKC_MADS	AT5G60910.1	maker-VaccDscaff32-augustus-gene-148.22-mRN
MIKC_MADS	AT5G60910.1	augustus_masked-VaccDscaff32-processed-gene
MIKC_MADS	AT5G60910.1	maker-VaccDscaff32-augustus-gene-276.18-mRN
MIKC_MADS	AT5G60910.1	maker-VaccDscaff30-augustus-gene-319.17-mRN

MIKC_MADS	AT5G60910.1	maker-VaccDscaff42-snap-gene-145.39-mRNA-1
NAC	AT5G61430.1	maker-VaccDscaff16-augustus-gene-66.30-mRNA
NAC	AT5G61430.1	maker-VaccDscaff13-augustus-gene-80.13-mRNA
NAC	AT5G61430.1	maker-VaccDscaff13-augustus-gene-251.11-mRN
NAC	AT5G61430.1	maker-VaccDscaff7-snap-gene-358.36-mRNA-1
NAC	AT5G61430.1	maker-VaccDscaff18-snap-gene-330.31-mRNA-1
NAC	AT5G61430.1	maker-VaccDscaff32-augustus-gene-141.23-mRN
NAC	AT5G61430.1	maker-VaccDscaff32-augustus-gene-285.31-mRN
NAC	AT5G61430.1	maker-VaccDscaff30-augustus-gene-151.25-mRN
NAC	AT5G61430.1	maker-VaccDscaff30-augustus-gene-326.25-mRN
NAC	AT5G61430.1	maker-VaccDscaff42-augustus-gene-163.37-mRN
LFY	AT5G61850.1	maker-VaccDscaff27-snap-gene-283.34-mRNA-1
LFY	AT5G61850.1	maker-VaccDscaff17-augustus-gene-318.25-mRN
LFY	AT5G61850.1	maker-VaccDscaff50-augustus-gene-16.38-mRNA
LFY	AT5G61850.1	maker-VaccDscaff34-augustus-gene-61.40-mRNA
ERF	AT5G61890.1	maker-VaccDscaff27-augustus-gene-283.20-mRN
ERF	AT5G61890.1	maker-VaccDscaff12-augustus-gene-88.30-mRNA
ERF	AT5G61890.1	maker-VaccDscaff36-augustus-gene-39.22-mRNA
ERF	AT5G61890.1	maker-VaccDscaff36-augustus-gene-201.19-mRN
ERF	AT5G61890.1	maker-VaccDscaff17-augustus-gene-317.37-mRN
ERF	AT5G61890.1	maker-VaccDscaff41-augustus-gene-52.32-mRNA
ERF	AT5G61890.1	maker-VaccDscaff386-augustus-gene-0.29-mRNA
ERF	AT5G61890.1	maker-VaccDscaff9-augustus-gene-214.21-mRNA
ERF	AT5G61890.1	snap_masked-VaccDscaff50-processed-gene-15.
ERF	AT5G61890.1	snap_masked-VaccDscaff35-processed-gene-154
ERF	AT5G61890.1	maker-VaccDscaff34-snap-gene-62.47-mRNA-1
ERF	AT5G61890.1	snap_masked-VaccDscaff4-processed-gene-252.
ERF	AT5G61890.1	maker-VaccDscaff23-augustus-gene-323.27-mRN
ERF	AT5G61890.1	maker-VaccDscaff3-augustus-gene-84.42-mRNA-
HSF	AT5G62020.1	maker-VaccDscaff21-augustus-gene-65.43-mRNA
HSF	AT5G62020.1	maker-VaccDscaff33-augustus-gene-271.38-mRN
HSF	AT5G62020.1	maker-VaccDscaff26-augustus-gene-64.43-mRNA
HSF	AT5G62020.1	maker-VaccDscaff29-augustus-gene-66.45-mRNA
MIKC_MADS	AT5G62165.3	maker-VaccDscaff21-augustus-gene-73.22-mRNA
MIKC_MADS	AT5G62165.3	maker-VaccDscaff33-snap-gene-264.39-mRNA-1
MYB	AT5G62470.2	maker-VaccDscaff25-augustus-gene-250.33-mRN
MYB	AT5G62470.2	maker-VaccDscaff36-augustus-gene-83.18-mRNA
MYB	AT5G62470.2	maker-VaccDscaff13-snap-gene-257.23-mRNA-1
MYB	AT5G62470.2	maker-VaccDscaff9-augustus-gene-334.16-mRNA
MYB	AT5G62470.2	maker-VaccDscaff32-augustus-gene-121.31-mRN
MYB	AT5G62470.2	maker-VaccDscaff35-augustus-gene-264.28-mRN
MYB	AT5G62470.2	maker-VaccDscaff30-augustus-gene-146.39-mRN
MYB	AT5G62470.2	maker-VaccDscaff269-augustus-gene-0.45-mRNA
MYB	AT5G62470.2	maker-VaccDscaff4-augustus-gene-358.29-mRNA
MYB	AT5G62470.2	maker-VaccDscaff3-augustus-gene-137.26-mRNA
MYB	AT5G62470.2	maker-VaccDscaff3-augustus-gene-145.18-mRNA

LBD	AT5G63090.4	maker-VaccDscaff27-snap-gene-259.34-mRNA-1
LBD	AT5G63090.4	augustus_masked-VaccDscaff27-processed-gene
LBD	AT5G63090.4	augustus_masked-VaccDscaff17-processed-gene
LBD	AT5G63090.4	augustus_masked-VaccDscaff34-processed-gene
C2H2	AT5G63280.1	maker-VaccDscaff27-augustus-gene-264.29-mRN
C2H2	AT5G63280.1	maker-VaccDscaff36-augustus-gene-50.34-mRNA
C2H2	AT5G63280.1	maker-VaccDscaff17-augustus-gene-297.31-mRN
C2H2	AT5G63280.1	maker-VaccDscaff9-snap-gene-359.50-mRNA-1
C2H2	AT5G63280.1	maker-VaccDscaff35-augustus-gene-276.38-mRN
C2H2	AT5G63280.1	maker-VaccDscaff34-snap-gene-82.36-mRNA-1
C2H2	AT5G63280.1	maker-VaccDscaff4-snap-gene-379.55-mRNA-1
Trihelix	AT5G63420.1	maker-VaccDscaff27-snap-gene-21.40-mRNA-1
Trihelix	AT5G63420.1	maker-VaccDscaff27-snap-gene-21.43-mRNA-1
Trihelix	AT5G63420.1	maker-VaccDscaff27-snap-gene-268.37-mRNA-1
Trihelix	AT5G63420.1	augustus_masked-VaccDscaff36-processed-gene
Trihelix	AT5G63420.1	maker-VaccDscaff45-snap-gene-47.47-mRNA-1
Trihelix	AT5G63420.1	snap_masked-VaccDscaff45-processed-gene-47.
Trihelix	AT5G63420.1	maker-VaccDscaff45-snap-gene-47.52-mRNA-1
Trihelix	AT5G63420.1	maker-VaccDscaff17-augustus-gene-77.33-mRNA
Trihelix	AT5G63420.1	maker-VaccDscaff17-snap-gene-301.30-mRNA-1
Trihelix	AT5G63420.1	maker-VaccDscaff9-augustus-gene-361.41-mRNA
Trihelix	AT5G63420.1	maker-VaccDscaff50-augustus-gene-0.30-mRNA-
Trihelix	AT5G63420.1	maker-VaccDscaff50-augustus-gene-0.30-mRNA-
Trihelix	AT5G63420.1	augustus_masked-VaccDscaff35-processed-gene
Trihelix	AT5G63420.1	maker-VaccDscaff34-snap-gene-77.41-mRNA-1
Trihelix	AT5G63420.1	maker-VaccDscaff34-snap-gene-272.45-mRNA-1
Trihelix	AT5G63420.1	maker-VaccDscaff34-augustus-gene-272.41-mRN
Trihelix	AT5G63420.1	maker-VaccDscaff4-snap-gene-381.64-mRNA-1
NAC	AT5G64060.1	maker-VaccDscaff33-snap-gene-322.24-mRNA-1
NAC	AT5G64530.1	maker-VaccDscaff27-augustus-gene-85.25-mRNA
NAC	AT5G64530.1	maker-VaccDscaff45-augustus-gene-83.31-mRNA
NAC	AT5G64530.1	maker-VaccDscaff2-augustus-gene-373.34-mRNA
NAC	AT5G64530.1	maker-VaccDscaff17-augustus-gene-123.28-mRN
NAC	AT5G64530.1	maker-VaccDscaff14-augustus-gene-322.40-mRN
NAC	AT5G64530.1	maker-VaccDscaff34-snap-gene-229.47-mRNA-1
NAC	AT5G64530.1	maker-VaccDscaff3-augustus-gene-35.29-mRNA-
WRKY	AT5G64810.1	maker-VaccDscaff38-augustus-gene-99.31-mRNA
WRKY	AT5G64810.1	maker-VaccDscaff6-augustus-gene-314.28-mRNA
WRKY	AT5G64810.1	maker-VaccDscaff37-augustus-gene-211.25-mRN
WRKY	AT5G64810.1	maker-VaccDscaff39-augustus-gene-105.22-mRN
Dof	AT5G65590.1	maker-VaccDscaff13-augustus-gene-135.30-mRN
Dof	AT5G65590.1	maker-VaccDscaff32-augustus-gene-235.20-mRN
Dof	AT5G65590.1	maker-VaccDscaff30-augustus-gene-230.23-mRN
Dof	AT5G65590.1	maker-VaccDscaff42-augustus-gene-68.26-mRNA
bHLH	AT5G65640.1	maker-VaccDscaff12-augustus-gene-130.27-mRN
bHLH	AT5G65640.1	maker-VaccDscaff16-augustus-gene-122.22-mRN

bHLH	AT5G65640.1	maker-VaccDscaff7-snap-gene-310.23-mRNA-1
bHLH	AT5G65640.1	maker-VaccDscaff31-augustus-gene-31.26-mRNA
bHLH	AT5G65640.1	maker-VaccDscaff26-snap-gene-142.22-mRNA-1
bHLH	AT5G65640.1	maker-VaccDscaff18-snap-gene-272.28-mRNA-1
bHLH	AT5G65640.1	maker-VaccDscaff40-augustus-gene-57.14-mRNA
bHLH	AT5G65640.1	maker-VaccDscaff41-augustus-gene-11.27-mRNA
bHLH	AT5G65640.1	maker-VaccDscaff23-augustus-gene-284.18-mRN
bHLH	AT5G65640.1	maker-VaccDscaff29-snap-gene-139.33-mRNA-1
MYB	AT5G65790.1	maker-VaccDscaff12-augustus-gene-121.33-mRN
MYB	AT5G65790.1	maker-VaccDscaff40-augustus-gene-49.31-mRNA
MYB	AT5G65790.1	maker-VaccDscaff41-augustus-gene-20.14-mRNA
MYB	AT5G65790.1	maker-VaccDscaff14-snap-gene-6.26-mRNA-1
MYB	AT5G65790.1	maker-VaccDscaff23-augustus-gene-292.17-mRN
GATA	AT5G66320.2	maker-VaccDscaff32-augustus-gene-321.22-mRN
GATA	AT5G66320.2	maker-VaccDscaff30-augustus-gene-299.45-mRN
GATA	AT5G66320.2	maker-VaccDscaff42-augustus-gene-17.39-mRNA
SRS	AT5G66350.1	maker-VaccDscaff30-snap-gene-116.30-mRNA-1
C2H2	AT5G66730.1	maker-VaccDscaff16-augustus-gene-366.34-mRN
C2H2	AT5G66730.1	maker-VaccDscaff7-augustus-gene-34.24-mRNA-
C2H2	AT5G66730.1	maker-VaccDscaff31-augustus-gene-310.26-mRN
C2H2	AT5G66730.1	maker-VaccDscaff18-augustus-gene-31.33-mRNA
GRAS	AT5G66770.1	augustus_masked-VaccDscaff21-processed-gene
GRAS	AT5G66770.1	augustus_masked-VaccDscaff26-processed-gene
GRAS	AT5G66770.1	augustus_masked-VaccDscaff26-processed-gene
GRAS	AT5G66770.1	augustus_masked-VaccDscaff29-processed-gene
LBD	AT5G66870.1	augustus_masked-VaccDscaff16-processed-gene
LBD	AT5G66870.1	maker-VaccDscaff13-augustus-gene-133.22-mRN
LBD	AT5G66870.1	augustus_masked-VaccDscaff7-processed-gene-
LBD	AT5G66870.1	augustus_masked-VaccDscaff96-processed-gene
LBD	AT5G66870.1	augustus_masked-VaccDscaff31-processed-gene
LBD	AT5G66870.1	maker-VaccDscaff32-snap-gene-236.36-mRNA-1
LBD	AT5G66870.1	augustus_masked-VaccDscaff30-processed-gene
LBD	AT5G66870.1	augustus_masked-VaccDscaff42-processed-gene
bHLH	AT5G67060.1	augustus_masked-VaccDscaff12-processed-gene
bHLH	AT5G67060.1	augustus_masked-VaccDscaff40-processed-gene
bHLH	AT5G67060.1	augustus_masked-VaccDscaff41-processed-gene
bHLH	AT5G67060.1	augustus_masked-VaccDscaff23-processed-gene
MYB	AT5G67300.1	augustus_masked-VaccDscaff12-processed-gene
MYB	AT5G67300.1	augustus_masked-VaccDscaff16-processed-gene
MYB	AT5G67300.1	augustus_masked-VaccDscaff40-processed-gene
MYB	AT5G67300.1	augustus_masked-VaccDscaff41-processed-gene
MYB	AT5G67300.1	augustus_masked-VaccDscaff23-processed-gene
MYB_related	AT5G67580.2	maker-VaccDscaff460-snap-gene-0.19-mRNA-1
MYB_related	AT5G67580.2	maker-VaccDscaff7-snap-gene-292.29-mRNA-1
MYB_related	AT5G67580.2	maker-VaccDscaff31-snap-gene-54.24-mRNA-1
MYB_related	AT5G67580.2	maker-VaccDscaff18-snap-gene-263.26-mRNA-1

-4.5-mRNA-1
-4.3-mRNA-1
-1
NA-1
NA-1

A-1

A-1
NA-1
NA-1
A-1
A-1
NA-1
NA-1
NA-1
A-1
A-1
-94.1-mRNA-1
115.3-mRNA-1
319.6-mRNA-1
341.3-mRNA-1

-1

-7.4-mRNA-1
-253.1-mRNA-1

NA-1
NA-1
A-1
-1
A-1
A-1
NA-1
A-1
A-1
4.11-mRNA-1

A-1
e-282.2-mRNA-1

e-104.5-mRNA-1
e-148.3-mRNA-1
e-189.1-mRNA-1
e-334.6-mRNA-1
e-334.6-mRNA-1
e-334.6-mRNA-1
e-334.5-mRNA-1
e-334.5-mRNA-1
e-151.4-mRNA-1
5.11-mRNA-1
5.11-mRNA-1

e-324.5-mRNA-1

5.15-mRNA-1

e-80.5-mRNA-1
e-149.3-mRNA-1

NA-1
e-352.4-mRNA-1
e-352.4-mRNA-1
e-324.4-mRNA-1
e-324.4-mRNA-1
e-80.10-mRNA-1
NA-1
NA-1
NA-1
16-mRNA-1

NA-1
A-1
24-mRNA-1

A-1
NA-1
NA-1
A-1
A-1
NA-1
NA-1
A-1

NA-1
NA-1
A-1
NA-1
NA-1
e-253.9-mRNA-1
NA-1

0-mRNA-1
9-mRNA-1
118.6-mRNA-1
e-236.0-mRNA-1
e-118.7-mRNA-1
10-mRNA-1
10-mRNA-1
e-31.4-mRNA-1

19-mRNA-1

17-mRNA-1
NA-1
NA-1
e-84.3-mRNA-1
NA-1
A-1

A-1
e-83.9-mRNA-1
NA-1
e-340.8-mRNA-1
e-294.3-mRNA-1
e-295.0-mRNA-1
299.3-mRNA-1
e-236.2-mRNA-1
e-220.4-mRNA-1
e-253.3-mRNA-1
e-123.3-mRNA-1
e-27.4-mRNA-1
339.10-mRNA-1
e-23.0-mRNA-1
113.10-mRNA-1
e-226.7-mRNA-1
3.7-mRNA-1
NA-1
NA-1
NA-1
NA-1
NA-1
A-1
A-1
NA-1

NA-1
A-1
NA-1
A-1
A-1
A-1

ne-0.0-mRNA-1
e-21.8-mRNA-1
e-219.6-mRNA-1
2.17-mRNA-1
e-5.5-mRNA-1
e-38.4-mRNA-1
e-22.0-mRNA-1

NA-1

NA-1

A-1
NA-1
A-1
A-1
A-1

A-1
NA-1
A-1
NA-1
A-1
NA-1
NA-1
NA-1

e-312.8-mRNA-1
43.3-mRNA-1
NA-1
6-mRNA-1
25-mRNA-1

NA-1

e-320.2-mRNA-1
2.12-mRNA-1

4.22-mRNA-1
e-215.1-mRNA-1
9.16-mRNA-1
9.7-mRNA-1
9.7-mRNA-1

0-mRNA-1
A-1
A-1
21-mRNA-1

NA-1

208.3-mRNA-1
e-162.4-mRNA-1

233.6-mRNA-1
1.12-mRNA-1
188.12-mRNA-1
e-144.5-mRNA-1
254.2-mRNA-1
A-1
e-190.3-mRNA-1

A-1
ne-0.10-mRNA-1
A-1
222.8-mRNA-1
222.9-mRNA-1

NA-1
NA-1

NA-1
NA-1

A-1

A-1

A-1
NA-1
A-1
A-1
A-1
NA-1
A-1
A-1
375.10-mRNA-1
NA-1
NA-1
NA-1
NA-1
NA-1
NA-1
NA-1
A-1

NA-1

114.5-mRNA-1
NA-1
NA-1
e-84.8-mRNA-1
e-84.8-mRNA-1
e-281.0-mRNA-1
e-281.0-mRNA-1
2.12-mRNA-1
2.12-mRNA-1

A-1
A-1
A-1

NA-1

A-1
A-1
-1
A-1
e-112.2-mRNA-1
e-355.5-mRNA-1
51.6-mRNA-1
e-303.5-mRNA-1
402.7-mRNA-1
e-40.7-mRNA-1
e-40.5-mRNA-1
e-348.9-mRNA-1
ne-0.7-mRNA-1
e-300.2-mRNA-1
10.6-mRNA-1
1.16-mRNA-1
162.4-mRNA-1
e-109.0-mRNA-1
177.4-mRNA-1
e-123.5-mRNA-1
220.4-mRNA-1

NA-1
NA-1
A-1
NA-1

A-1

A-1
221.4-mRNA-1
-181.0-mRNA-1
A-1
IA-1
IA-1

IA-1

A-1
IA-1
A-1
IA-1
IA-1
A-1
IA-1

IA-1
IA-1
A-1
-1
A-1
IA-1
-1
.
A-1

-1
IA-1
IA-1
A-1
-1
A-1
A-1
A-1
A-1
IA-1
IA-1

\-1

JA-1

e-174.6-mRNA-1
e-7.4-mRNA-1
5.19-mRNA-1
JA-1
e-5.1-mRNA-1
e-44.2-mRNA-1
e-27.6-mRNA-1
e-105.5-mRNA-1
1.7-mRNA-1
e-55.8-mRNA-1
e-20.6-mRNA-1
JA-1

JA-1
\-1
JA-1
\-1
JA-1
\-1
\-1
JA-1

\-1
\-1
JA-1
JA-1
\-1

e-80.6-mRNA-1
e-80.6-mRNA-1
\-1
JA-1
JA-1

5.15-mRNA-1
\-1

A-1

e-337.9-mRNA-1
-1
A-1
A-1

A-1
A-1
-1

A-1
A-1
NA-1
A-1
A-1
-1
NA-1

A-1
A-1
A-1
NA-1
13-mRNA-1
16-mRNA-1
e-347.2-mRNA-1
NA-1
NA-1
-1
-1
NA-1
A-1
NA-1
NA-1

A-1
NA-1
A-1

e-4.5-mRNA-1
5.0-mRNA-1
e-340.2-mRNA-1

e-32.7-mRNA-1
420.6-mRNA-1
442.10-mRNA-1
e-167.2-mRNA-1
e-87.4-mRNA-1
e-368.8-mRNA-1
e-85.0-mRNA-1
e-125.1-mRNA-1
e-268.9-mRNA-1
e-251.0-mRNA-1
e-227.7-mRNA-1
e-49.8-mRNA-1
e-212.5-mRNA-1

A-1
A-1

e-340.6-mRNA-1
e-380.10-mRNA-1
ie-0.4-mRNA-1

13-mRNA-1
13-mRNA-1
13-mRNA-1
A-1
5.17-mRNA-1
0.12-mRNA-1

e-323.3-mRNA-1
e-42.9-mRNA-1
e-43.1-mRNA-1
10.4-mRNA-1
30.4-mRNA-1
e-1.3-mRNA-1

-1
-1
-1
-1

9-mRNA-1
A-1

22-mRNA-1
21-mRNA-1
3.23-mRNA-1
A-1
373.0-mRNA-1
25-mRNA-1
3.23-mRNA-1
6.22-mRNA-1
9-mRNA-1

NA-1

NA-1
A-1
NA-1
A-1

A-1
305.2-mRNA-1
e-352.3-mRNA-1

e-303.0-mRNA-1
51.0-mRNA-1
3.21-mRNA-1

NA-1
NA-1
-1
NA-1
NA-1
A-1
A-1
NA-1
NA-1

NA-1
NA-1
NA-1

A-1
ne-0.2-mRNA-1
e-63.8-mRNA-1
e-63.5-mRNA-1

1.24-mRNA-1
NA-1
e-38.0-mRNA-1

NA-1
NA-1
NA-1
NA-1
NA-1
e-98.5-mRNA-1
17-mRNA-1
286.7-mRNA-1

NA-1
A-1
A-1
NA-1
A-1
A-1
e-193.0-mRNA-1
A-1
A-1

A-1
A-1
NA-1
NA-1

4.17-mRNA-1
9-mRNA-1
A-1
A-1
e-174.0-mRNA-1
A-1

10-mRNA-1
A-1
NA-1
NA-1
NA-1
NA-1
NA-1
NA-1

JA-1
3.24-mRNA-1
JA-1
1.8-mRNA-1
JA-1
e-228.2-mRNA-1
1.13-mRNA-1
JA-1
JA-1
A-1
JA-1
JA-1
A-1
JA-1
JA-1
299.4-mRNA-1
e-253.4-mRNA-1
113.9-mRNA-1

JA-1
A-1
JA-1
A-1
JA-1
JA-1
A-1
JA-1
A-1
A-1
A-1
JA-1
JA-1

JA-1
A-1
A-1
A-1
JA-1
A-1
-1

\-1
\-1
JA-1
6-mRNA-1

JA-1
JA-1

JA-1
266.2-mRNA-1
-238.9-mRNA-1
-238.6-mRNA-1
-239.0-mRNA-1
154.6-mRNA-1
.25-mRNA-1
-120.5-mRNA-1
-120.0-mRNA-1

JA-1
JA-1
\-1

JA-1
JA-1
JA-1
\-1

\-1

-1
\-1
\-1
JA-1
\-1
\-1
JA-1
JA-1
\-1
-1
-1
1-mRNA-1

22-mRNA-1
\-1

JA-1
e-155.7-mRNA-1
JA-1
JA-1
e-149.4-mRNA-1
JA-1
JA-1
JA-1
JA-1
JA-1

JA-1
-1
e-91.8-mRNA-1
e-131.2-mRNA-1
JA-1
JA-1
-1

A-1
JA-1
JA-1
JA-1
e-68.7-mRNA-1
).14-mRNA-1

e-308.1-mRNA-1
e-27.1-mRNA-1
e-0.0-mRNA-1
3.14-mRNA-1

e-343.6-mRNA-1
ne-3.14-mRNA-1
e-62.6-mRNA-1
10-mRNA-1
5.11-mRNA-1
ene-0.0-mRNA-1
JA-1

JA-1
JA-1
4.16-mRNA-1
-1
JA-1
A-1

A-1
ene-0.1-mRNA-1
e-60.8-mRNA-1

-mRNA-1

1.21-mRNA-1
16-mRNA-1
NA-1
-1
NA-1
A-1
15-mRNA-1
NA-1
e-284.0-mRNA-1
A-1
NA-1

NA-1
-1
NA-1
A-1

NA-1
NA-1
A-1
-1

NA-1

e-209.5-mRNA-1
9-mRNA-1
e-79.7-mRNA-1

e-236.2-mRNA-1

A-1
A-1
e-128.6-mRNA-1
A-1
A-1

A-1
e-296.10-mRNA-1
5.36-mRNA-1
22-mRNA-1
NA-1
A-1
3.10-mRNA-1
26-mRNA-1
NA-1
A-1
A-1
NA-1
0.19-mRNA-1
0.19-mRNA-1
e-309.1-mRNA-1
A-1
NA-1
A-1
A-1
NA-1
-1
A-1
NA-1
NA-1
NA-1
7.15-mRNA-1
7.15-mRNA-1

e-277.2-mRNA-1
30-mRNA-1
A-1
377.3-mRNA-1
e-162.6-mRNA-1
NA-1
NA-1
NA-1
ne-0.0-mRNA-1
NA-1
NA-1
A-1
NA-1

-1
NA-1
NA-1
NA-1
NA-1
NA-1

e-129.3-mRNA-1
20-mRNA-1
e-179.6-mRNA-1
NA-1
e-135.7-mRNA-1
12-mRNA-1
6-mRNA-1
NA-1

A-1
A-1
NA-1
NA-1

NA-1
A-1
NA-1
NA-1
A-1
A-1
NA-1
e-92.9-mRNA-1
e-91.10-mRNA-1
e-0.5-mRNA-1
e-221.3-mRNA-1

NA-1
NA-1
NA-1
A-1
NA-1
A-1
A-1
NA-1
NA-1
A-1
NA-1

IA-1

A-1
IA-1

IA-1
A-1
IA-1
IA-1
A-1
A-1
e-230.0-mRNA-1
A-1
A-1
A-1
A-1
IA-1
IA-1
e-28.0-mRNA-1
e-98.1-mRNA-1
e-166.4-mRNA-1
e-121.9-mRNA-1
266.3-mRNA-1
e-78.5-mRNA-1
e-28.5-mRNA-1
e-43.0-mRNA-1
ne-0.5-mRNA-1
e-250.4-mRNA-1
e-245.6-mRNA-1
e-312.8-mRNA-1
IA-1
A-1
A-1
1.13-mRNA-1

24-mRNA-1
411.7-mRNA-1
e-231.3-mRNA-1
A-1

IA-1

IA-1
IA-1

NA-1

NA-1

NA-1
A-1
A-1
e-281.3-mRNA-1
NA-1
NA-1
NA-1
NA-1
NA-1
NA-1
A-1
NA-1

A-1
NA-1
NA-1

175.3-mRNA-1
NA-1

NA-1
NA-1
NA-1
A-1
A-1
-1
5.23-mRNA-1
NA-1
NA-1
e-57.10-mRNA-1
e-54.6-mRNA-1
e-345.5-mRNA-1
9.14-mRNA-1
e-300.10-mRNA-1
e-64.13-mRNA-1
e-361.7-mRNA-1
e-314.6-mRNA-1
e-72.5-mRNA-1
ne-1.1-mRNA-1

IA-1

IA-1
IA-1
A-1
IA-1
IA-1
IA-1
A-1
A-1
IA-1
IA-1
A-1
IA-1
e-340.7-mRNA-1
).16-mRNA-1
IA-1

IA-1
IA-1
e-62.11-mRNA-1

IA-1
IA-1
A-1
IA-1
-1
114.2-mRNA-1
e-168.9-mRNA-1
IA-1
A-1
A-1
A-1
IA-1

IA-1
A-1

-1

A-1
A-1

A-1
14-mRNA-1

NA-1
-1
A-1
A-1
NA-1
NA-1
A-1
NA-1

e-138.5-mRNA-1
A-1
e-112.4-mRNA-1
A-1
e-136.2-mRNA-1
e-218.2-mRNA-1
9.17-mRNA-1
35-mRNA-1
NA-1
-1
NA-1
-1
A-1
A-1
A-1

393.1-mRNA-1
-1
2.14-mRNA-1
55.5-mRNA-1
ne-1.7-mRNA-1
434.0-mRNA-1
e-0.5-mRNA-1
ne-1.11-mRNA-1
NA-1
A-1
A-1
A-1

JA-1
A-1
JA-1
JA-1
JA-1
1.11-mRNA-1
e-47.1-mRNA-1
e-242.5-mRNA-1
e-236.3-mRNA-1

JA-1

JA-1
JA-1
JA-1
JA-1

A-1
A-1

A-1

8-mRNA-1
A-1
JA-1
e-249.2-mRNA-1
265.7-mRNA-1
150.7-mRNA-1
160.8-mRNA-1
e-252.6-mRNA-1
271.6-mRNA-1
e-142.17-mRNA-1
e-194.0-mRNA-1
e-146.10-mRNA-1
144.4-mRNA-1
157.5-mRNA-1

A-1

\-1
IA-1
\-1
IA-1
\-1
:-44.10-mRNA-1
IA-1
IA-1

IA-1
:-72.0-mRNA-1
\-1
\-1
IA-1
\-1
\-1
\-1
\-1
IA-1
:-167.6-mRNA-1
:-167.6-mRNA-1

\-1
IA-1

206.5-mRNA-1
191.6-mRNA-1
239.8-mRNA-1

IA-1
\-1
IA-1

\-1
IA-1

\-1
IA-1
\-1
IA-1
\-1
IA-1

NA-1
A-1

NA-1
A-1
NA-1
NA-1
A-1
NA-1
NA-1
A-1
A-1
e-84.4-mRNA-1

e-351.2-mRNA-1
e-22.5-mRNA-1

e-343.2-mRNA-1

e-328.4-mRNA-1
e-338.8-mRNA-1
e-338.8-mRNA-1
e-82.7-mRNA-1
NA-1
A-1
NA-1
A-1
NA-1
e-370.2-mRNA-1
NA-1
e-362.10-mRNA-1
e-33.4-mRNA-1
7.8-mRNA-1

NA-1
NA-1
NA-1
NA-1

A-1
e-48.9-mRNA-1
A-1
NA-1

A-1

ne-0.0-mRNA-1
-1

A-1

NA-1
A-1
A-1
A-1
A-1
NA-1
1.18-mRNA-1
NA-1
-1
4.14-mRNA-1

NA-1

2.18-mRNA-1
NA-1

e-111.3-mRNA-1
e-30.0-mRNA-1
e-296.3-mRNA-1
e-249.5-mRNA-1
e-136.11-mRNA-1
e-64.8-mRNA-1
A-1

NA-1
15-mRNA-1
A-1
11-mRNA-1
NA-1
A-1
NA-1

ene-0.0-mRNA-1
ne-1.0-mRNA-1

NA-1

JA-1
A-1
4-mRNA-1
4-mRNA-1
e-5.0-mRNA-1

-1
e-377.6-mRNA-1
e-0.0-mRNA-1
e-3.6-mRNA-1
JA-1
JA-1
JA-1
-1
JA-1

-1
-1

e-365.6-mRNA-1
-1
JA-1
JA-1
e-369.5-mRNA-1
384.7-mRNA-1
e-360.3-mRNA-1
18-mRNA-1
).15-mRNA-1
e-385.3-mRNA-1
e-196.4-mRNA-1
49.5-mRNA-1
e-377.2-mRNA-1
37.1-mRNA-1
e-12.11-mRNA-1
e-14.8-mRNA-1
e-184.2-mRNA-1
JA-1
A-1

-1
JA-1
-1
-1
A-1

A-1

-1
A-1
A-1

0.20-mRNA-1

NA-1
4.16-mRNA-1
-1

e-79.3-mRNA-1
e-86.0-mRNA-1
346.11-mRNA-1
e-313.1-mRNA-1
e-322.0-mRNA-1
A-1
NA-1
NA-1
NA-1
NA-1
NA-1

A-1
NA-1
NA-1
A-1
A-1
NA-1
A-1
A-1
NA-1

NA-1
A-1
NA-1
NA-1
NA-1
e-116.2-mRNA-1

A-1
A-1
A-1
27-mRNA-1
e-252.3-mRNA-1

-1
A-1

-1
-1
NA-1

-1

-1
A-1
-1
A-1
A-1
A-1
A-1
ne-0.0-mRNA-1
A-1
NA-1
NA-1

NA-1
NA-1

e-194.8-mRNA-1
NA-1

199.5-mRNA-1
NA-1

NA-1
e-320.3-mRNA-1
e-312.4-mRNA-1
e-300.0-mRNA-1
e-39.0-mRNA-1
e-39.2-mRNA-1
e-317.1-mRNA-1
17-mRNA-1
NA-1
NA-1
NA-1
NA-1
A-1
6-mRNA-1
3-mRNA-1

e-2.5-mRNA-1
e-2.5-mRNA-1
e-2.5-mRNA-1

e-265.1-mRNA-1
14-mRNA-1
ne-0.2-mRNA-1
ne-0.2-mRNA-1
ne-0.2-mRNA-1
e-1.4-mRNA-1
e-1.4-mRNA-1
e-1.4-mRNA-1
NA-1
NA-1
NA-1
-1
-1

-1
A-1

NA-1

-1

IA-1
e-386.6-mRNA-1
2.17-mRNA-1
A-1
ne-0.9-mRNA-1
A-1
IA-1
e-17.4-mRNA-1
A-1
e-14.2-mRNA-1
1.14-mRNA-1
IA-1
IA-1

A-1
A-1
A-1
A-1
IA-1

A-1
e-15.4-mRNA-1
A-1

A-1
A-1

A-1
IA-1
1.27-mRNA-1
IA-1
A-1

A-1
-1

e-26.5-mRNA-1
34.7-mRNA-1
ne-0.3-mRNA-1
395.5-mRNA-1
416.4-mRNA-1
-1

-1
A-1
A-1
A-1
IA-1
IA-1
A-1
147.5-mRNA-1
A-1
A-1
A-1
IA-1
36-mRNA-1
-1

A-1
A-1

e-109.0-mRNA-1
A-1
2.25-mRNA-1
A-1
IA-1
IA-1

IA-1

IA-1
5.11-mRNA-1
-1
5.21-mRNA-1

8-mRNA-1
6-mRNA-1
e-154.6-mRNA-1
ie-0.3-mRNA-1
e-33.1-mRNA-1

IA-1

-1
-1

NA-1

418.4-mRNA-1

NA-1

-1

e-378.10-mRNA-1
e-55.1-mRNA-1
98.3-mRNA-1
338.2-mRNA-1
359.7-mRNA-1

NA-1

NA-1
-1
2-mRNA-1

e-293.0-mRNA-1
302.2-mRNA-1

16-mRNA-1
e-39.5-mRNA-1
e-284.2-mRNA-1
e-285.6-mRNA-1
e-368.5-mRNA-1
e-368.6-mRNA-1
e-169.11-mRNA-1
e-169.13-mRNA-1
78.2-mRNA-1
e-360.6-mRNA-1
e-360.7-mRNA-1
111.4-mRNA-1
e-23.12-mRNA-1
e-34.11-mRNA-1

64.2-mRNA-1

302.5-mRNA-1
302.4-mRNA-1
e-333.6-mRNA-1
e-39.7-mRNA-1
e-90.3-mRNA-1
3.26-mRNA-1
e-169.9-mRNA-1

5.12-mRNA-1
e-85.6-mRNA-1
e-360.12-mRNA-1

A-1
11-mRNA-1
e-271.6-mRNA-1
e-160.6-mRNA-1
e-30.0-mRNA-1
A-1
A-1
A-1
A-1
A-1

A-1

A-1

A-1
20-mRNA-1
-1
-1
e-335.6-mRNA-1
e-214.6-mRNA-1
NA-1
NA-1

A-1
NA-1
-1

A-1

41-mRNA-1
e-12.5-mRNA-1
NA-1
NA-1
A-1
e-64.6-mRNA-1

A-1
A-1
A-1
NA-1
NA-1
NA-1

A-1
A-1
NA-1
NA-1
NA-1
A-1
e-308.4-mRNA-1
8-mRNA-1
4-mRNA-1
e-78.5-mRNA-1
362.6-mRNA-1
e-301.2-mRNA-1
6.9-mRNA-1
4-mRNA-1
e-286.8-mRNA-1
3.28-mRNA-1
e-327.6-mRNA-1
358.0-mRNA-1
e-289.2-mRNA-1

A-1
3.13-mRNA-1
e-106.3-mRNA-1
e-90.8-mRNA-1
335.9-mRNA-1
e-1.0-mRNA-1
e-306.10-mRNA-1
e-110.2-mRNA-1

e-247.6-mRNA-1
e-6.4-mRNA-1

NA-1
NA-1
NA-1
NA-1
NA-1
NA-1
NA-1
A-1
A-1

NA-1
-1
e-283.7-mRNA-1
e-102.10-mRNA-1

A-1
NA-1

A-1
-1
-1
A-1
NA-1

A-1
A-1
A-1

NA-1
NA-1
NA-1
A-1
A-1

NA-1
NA-1
A-1
A-1

A-1
NA-1

.11-mRNA-1

18-mRNA-1
.12-mRNA-1
-1
.14-mRNA-1
12-mRNA-1
7-mRNA-1
.11-mRNA-1
-1
19-mRNA-1
A-1

A-1
A-1
-111.6-mRNA-1
A-1
A-1

-1
-1
-1
A-1
A-1
A-1
A-1

-121.3-mRNA-1
-215.5-mRNA-1
-125.1-mRNA-1
.18-mRNA-1
21.6-mRNA-1
-335.2-mRNA-1
-5.13-mRNA-1
e-0.3-mRNA-1
-172.3-mRNA-1
A-1
A-1
A-1
A-1
A-1
-1

A-1
e-14.0-mRNA-1
e-54.8-mRNA-1
e-15.2-mRNA-1
e-279.8-mRNA-1

e-283.3-mRNA-1
e-73.4-mRNA-1
e-269.4-mRNA-1
e-229.1-mRNA-1
e-258.3-mRNA-1
NA-1
A-1
NA-1
NA-1
NA-1
NA-1
A-1

A-1
A-1

NA-1
NA-1
A-1

e-275.2-mRNA-1

A-1
NA-1

e-370.7-mRNA-1
NA-1

e-309.0-mRNA-1
e-296.3-mRNA-1
NA-1
5.16-mRNA-1
1-mRNA-1
3.17-mRNA-1
19-mRNA-1

e-282.6-mRNA-1
5-mRNA-1
e-185.11-mRNA-1
e-186.0-mRNA-1
e-208.1-mRNA-1
A-1
NA-1
ene-0.0-mRNA-1
A-1
NA-1
ene-0.0-mRNA-1
NA-1
NA-1
NA-1
A-1
e-62.2-mRNA-1
A-1
NA-1
e-337.6-mRNA-1
e-338.1-mRNA-1
A-1
e-35.4-mRNA-1
NA-1
e-173.1-mRNA-1
e-165.0-mRNA-1
e-164.5-mRNA-1

NA-1
NA-1
A-1

5.16-mRNA-1
A-1
NA-1
e-214.0-mRNA-1
e-269.5-mRNA-1

e-153.3-mRNA-1
e-142.1-mRNA-1
NA-1
NA-1
NA-1
NA-1
NA-1

JA-1
A-1

A-1

JA-1
ie-0.2-mRNA-1
A-1

19-mRNA-1

JA-1
A-1
A-1
JA-1
A-1

A-1
A-1
JA-1
e-368.3-mRNA-1
e-4.12-mRNA-1
7.20-mRNA-1
329.4-mRNA-1
e-205.3-mRNA-1
e-284.0-mRNA-1
2-mRNA-1
e-193.3-mRNA-1
A-1
e-4.7-mRNA-1
e-260.7-mRNA-1
e-57.4-mRNA-1
e-43.0-mRNA-1

JA-1
JA-1
JA-1
A-1

A-1

A-1
A-1

NA-1

NA-1
NA-1
A-1
-mRNA-1
-1
A-1
-1
NA-1
58.6-mRNA-1
A-1
A-1
NA-1
A-1
NA-1
A-1
NA-1
A-1
A-1
NA-1
213.0-mRNA-1
e-114.1-mRNA-1
e-122.5-mRNA-1
ne-0.2-mRNA-1
-1
NA-1
NA-1
A-1
A-1
NA-1
NA-1
A-1
A-1
e-379.7-mRNA-1

e-379.7-mRNA-1
e-379.7-mRNA-1
NA-1
NA-1
NA-1
NA-1
NA-1
NA-1
A-1
A-1
A-1
A-1
NA-1
A-1
A-1
NA-1

A-1
NA-1
A-1

NA-1
e-17.2-mRNA-1
A-1

9.27-mRNA-1
NA-1
NA-1
NA-1
A-1

NA-1

NA-1
NA-1
324.0-mRNA-1
A-1

-1
A-1
A-1

NA-1
-1

A-1
A-1
44.3-mRNA-1
11-mRNA-1
0.11-mRNA-1
e-10.6-mRNA-1
17-mRNA-1
383.4-mRNA-1
e-217.7-mRNA-1
22-mRNA-1
e-265.12-mRNA-1
16-mRNA-1
2.20-mRNA-1
29-mRNA-1

NA-1
-1
NA-1

NA-1
NA-1
A-1
-1
5.1-mRNA-1
-1
.
-1
-1
e-339.8-mRNA-1
e-33.1-mRNA-1
e-175.5-mRNA-1
e-167.0-mRNA-1

NA-1
NA-1
NA-1
NA-1
A-1
A-1
3.13-mRNA-1
e-210.7-mRNA-1
e-97.2-mRNA-1

e-29.2-mRNA-1
e-22.1-mRNA-1
e-241.9-mRNA-1
15-mRNA-1
e-28.6-mRNA-1

79.1-mRNA-1
e-96.1-mRNA-1
e-327.13-mRNA-1
e-87.8-mRNA-1
10-mRNA-1
A-1
A-1
NA-1

NA-1
NA-1
A-1

NA-1
e-204.0-mRNA-1
NA-1
NA-1

NA-1
NA-1
NA-1
A-1
A-1
A-1

A-1

NA-1
e-249.6-mRNA-1
NA-1
A-1

NA-1
A-1

6.10-mRNA-1

8-mRNA-1
A-1
A-1
A-1

25-mRNA-1
e-59.9-mRNA-1
A-1

9.23-mRNA-1
7.8-mRNA-1
NA-1
349.4-mRNA-1
-1
A-1
A-1
e-243.7-mRNA-1
e-73.3-mRNA-1
e-70.3-mRNA-1
e-70.2-mRNA-1
e-267.4-mRNA-1

e-69.3-mRNA-1
14-mRNA-1
e-70.6-mRNA-1

e-70.1-mRNA-1

e-69.1-mRNA-1
e-70.5-mRNA-1
10-mRNA-1
10-mRNA-1
-1

A-1
30-mRNA-1
A-1
-1
-1

-1
A-1

A-1
A-1
-1
A-1
A-1
-1
A-1
A-1
A-1
-1
A-1

A-1
17-mRNA-1

-92.8-mRNA-1
1
A-1
-24.6-mRNA-1
A-1
A-1

1

18-mRNA-1
-14.6-mRNA-1
-327.2-mRNA-1
17.4-mRNA-1
0-mRNA-1
-5.4-mRNA-1
-mRNA-1
423.7-mRNA-1

A-1
A-1
-1
A-1
-1
A-1
A-1
A-1
-1
-1

NA-1
NA-1
e-111.1-mRNA-1

A-1
e-303.7-mRNA-1

ne-1.6-mRNA-1
e-222.2-mRNA-1
412.3-mRNA-1
e-238.3-mRNA-1
e-359.1-mRNA-1
e-135.11-mRNA-1
ne-0.4-mRNA-1
A-1
A-1
e-62.9-mRNA-1
NA-1
NA-1
0.16-mRNA-1
1.12-mRNA-1
NA-1
NA-1
NA-1
NA-1

NA-1
NA-1
NA-1

NA-1

NA-1

NA-1
NA-1
NA-1
A-1
A-1
NA-1
NA-1

IA-1
-101.2-mRNA-1
-31.0-mRNA-1
-38.2-mRNA-1
-311.8-mRNA-1
IA-1
IA-1
\-1

\-1
\-1
\-1
-301.5-mRNA-1
IA-1
IA-1
-114.6-mRNA-1
-260.6-mRNA-1
-269.6-mRNA-1
-117.2-mRNA-1
l.15-mRNA-1
8-mRNA-1
IA-1

\-1
-264.0-mRNA-1
IA-1
\-1
IA-1
\-1
\-1
-1
IA-1
IA-1
-1

\-1
IA-1
-192.10-mRNA-1

IA-1
IA-1
IA-1

10-mRNA-1

-213.0-mRNA-1

NA-1

NA-1

NA-1
A-1
NA-1
A-1
A-1
NA-1
A-1
A-1
A-1
A-1
NA-1
NA-1
NA-1
NA-1
NA-1
NA-1
NA-1

37.3-mRNA-1
e-308.7-mRNA-1

A-1

20-mRNA-1
e-185.4-mRNA-1
NA-1
A-1
NA-1
NA-1
e-17.0-mRNA-1

A-1
A-1
A-1
A-1
A-1
NA-1
-1

NA-1

NA-1
A-1
A-1
NA-1
A-1

-1

133.0-mRNA-1

NA-1
NA-1
NA-1
A-1
NA-1
NA-1
NA-1

NA-1
NA-1
NA-1
NA-1
NA-1
NA-1

NA-1
NA-1
NA-1

A-1
NA-1
NA-1
A-1

NA-1
NA-1
A-1
A-1

NA-1
NA-1
NA-1
9.30-mRNA-1
NA-1
NA-1
e-49.5-mRNA-1
e-53.9-mRNA-1
e-192.3-mRNA-1
NA-1
e-49.9-mRNA-1
NA-1
NA-1
NA-1
A-1
-1
-1
A-1
4.14-mRNA-1
e-14.0-mRNA-1
NA-1
NA-1
NA-1

e-350.1-mRNA-1
NA-1
e-97.6-mRNA-1
e-241.1-mRNA-1
e-96.1-mRNA-1
e-96.5-mRNA-1
e-95.6-mRNA-1
e-298.0-mRNA-1
e-329.4-mRNA-1
e-282.2-mRNA-1
e-103.6-mRNA-1
e-103.1-mRNA-1
3.20-mRNA-1
NA-1
NA-1
13-mRNA-1

NA-1
NA-1
A-1
NA-1
A-1
20-mRNA-1
12-mRNA-1

e-27.1-mRNA-1
NA-1

e-253.4-mRNA-1
e-248.4-mRNA-1

e-250.3-mRNA-1

NA-1

A-1
A-1
e-318.8-mRNA-1
e-248.0-mRNA-1
e-0.6-mRNA-1
e-327.2-mRNA-1
ne-3.7-mRNA-1
ne-3.14-mRNA-1
229.4-mRNA-1
e-117.7-mRNA-1
394.3-mRNA-1
e-306.3-mRNA-1
e-26.12-mRNA-1
421.9-mRNA-1
e-109.3-mRNA-1
A-1
NA-1
9-mRNA-1
5.11-mRNA-1
NA-1
-1
A-1
NA-1
A-1

e-276.5-mRNA-1
A-1
NA-1
A-1
NA-1
NA-1
A-1
A-1
NA-1
26-mRNA-1
A-1
A-1
ne-0.10-mRNA-1
e-244.4-mRNA-1
9.13-mRNA-1

231.9-mRNA-1
e-191.1-mRNA-1
e-7.13-mRNA-1
204.3-mRNA-1
A-1
A-1
NA-1
NA-1
NA-1
-1

A-1
A-1
A-1
16-mRNA-1
NA-1

NA-1
NA-1
NA-1
A-1
NA-1
e-62.2-mRNA-1
362.2-mRNA-1
e-335.5-mRNA-1

A-1

NA-1

A-1
NA-1
e-25.5-mRNA-1
0.20-mRNA-1
A-1
e-40.3-mRNA-1
8-mRNA-1
A-1
A-1
NA-1
A-1
-1
-1
NA-1
NA-1

NA-1
5.14-mRNA-1
NA-1
NA-1
247.3-mRNA-1
A-1

5.7-mRNA-1
A-1
12-mRNA-1
e-74.0-mRNA-1
e-71.1-mRNA-1
e-240.8-mRNA-1
A-1
NA-1
ne-0.1-mRNA-1
e-324.5-mRNA-1
NA-1
A-1
NA-1
NA-1
A-1
e-74.1-mRNA-1
e-74.1-mRNA-1
e-74.1-mRNA-1
e-256.4-mRNA-1
A-1
A-1

e-374.6-mRNA-1
e-374.6-mRNA-1
e-374.6-mRNA-1
e-374.6-mRNA-1

e-16.2-mRNA-1
e-304.4-mRNA-1
e-12.2-mRNA-1
e-11.5-mRNA-1
e-234.4-mRNA-1
NA-1

NA-1

A-1

e-64.0-mRNA-1
ne-2.9-mRNA-1
NA-1
ne-0.1-mRNA-1
NA-1

NA-1
NA-1
A-1
NA-1
NA-1
e-248.6-mRNA-1
NA-1
NA-1
NA-1
e-294.4-mRNA-1

e-325.6-mRNA-1
e-278.5-mRNA-1
e-106.6-mRNA-1

e-18.4-mRNA-1
A-1

23-mRNA-1
NA-1

A-1
e-208.0-mRNA-1
209.15-mRNA-1
e-150.6-mRNA-1
248.11-mRNA-1
A-1
NA-1
NA-1
NA-1
NA-1
e-226.4-mRNA-1
A-1
NA-1

NA-1
e-23.3-mRNA-1
A-1
A-1
A-1

NA-1

A-1
A-1
NA-1

-1

A-1
-1
A-1
-1
A-1
e-323.5-mRNA-1
e-6.4-mRNA-1

e-109.4-mRNA-1
NA-1
A-1
NA-1
A-1
A-1
22-mRNA-1
NA-1

A-1
A-1
NA-1
NA-1
NA-1
NA-1
NA-1
A-1
NA-1
NA-1

e-338.9-mRNA-1
NA-1
A-1
A-1

A-1
3.13-mRNA-1
A-1
-1
A-1
A-1

NA-1

NA-1
NA-1
A-1

A-1
NA-1

NA-1

A-1
-1
21-mRNA-1

20-mRNA-1
23-mRNA-1

A-1

NA-1

A-1
NA-1
NA-1
-1
NA-1
A-1
e-351.9-mRNA-1
e-353.7-mRNA-1
e-160.9-mRNA-1
e-160.9-mRNA-1
NA-1
-1
A-1
11-mRNA-1
e-203.3-mRNA-1
ne-0.2-mRNA-1
195.6-mRNA-1

141.5-mRNA-1
163.3-mRNA-1
e-57.8-mRNA-1
e-110.3-mRNA-1
e-27.10-mRNA-1
e-223.8-mRNA-1
e-223.8-mRNA-1
233.3-mRNA-1
e-250.0-mRNA-1
-1
-1
A-1
A-1
3.14-mRNA-1
e-39.2-mRNA-1

ne-1.3-mRNA-1
430.7-mRNA-1

A-1
A-1

A-1
A-1
A-1
A-1

3.31-mRNA-1
A-1
A-1
3.17-mRNA-1
38-mRNA-1
e-245.8-mRNA-1
0.12-mRNA-1

e-152.10-mRNA-1
e-340.5-mRNA-1
e-185.0-mRNA-1
A-1
17-mRNA-1
e-156.10-mRNA-1
e-296.6-mRNA-1
e-153.10-mRNA-1
62.5-mRNA-1
14-mRNA-1
NA-1

NA-1
A-1
NA-1
NA-1
NA-1
A-1
-1
-1
NA-1
8.0-mRNA-1
8.0-mRNA-1
e-158.5-mRNA-1

e-210.5-mRNA-1
18-mRNA-1
e-39.6-mRNA-1
e-28.10-mRNA-1
ne-2.9-mRNA-1
ne-0.8-mRNA-1
431.0-mRNA-1

7-mRNA-1

e-298.4-mRNA-1
e-71.5-mRNA-1
e-329.10-mRNA-1
A-1
3.7-mRNA-1
e-208.5-mRNA-1
18-mRNA-1

e-312.5-mRNA-1
e-128.6-mRNA-1
e-128.7-mRNA-1
e-239.3-mRNA-1
e-239.2-mRNA-1
e-69.2-mRNA-1

e-267.8-mRNA-1
e-68.0-mRNA-1
e-7.6-mRNA-1
e-7.5-mRNA-1
e-21.4-mRNA-1
e-21.3-mRNA-1
e-70.3-mRNA-1
e-350.12-mRNA-1
e-350.13-mRNA-1
e-77.6-mRNA-1
e-77.7-mRNA-1
A-1
NA-1
A-1
A-1
A-1
-1

-1
A-1

e-16.3-mRNA-1
NA-1
A-1
NA-1
A-1
8-mRNA-1
A-1

A-1
-1

A-1
A-1
NA-1
A-1
A-1
-1
A-1
A-1
A-1

-1
0.12-mRNA-1

-1
A-1
A-1
A-1
A-1

A-1

A-1
e-8.18-mRNA-1
ne-1.10-mRNA-1
A-1
A-1
-1
A-1
A-1
-1
A-1
-1

e-9.5-mRNA-1
NA-1
A-1
NA-1
NA-1

A-1
-1
-1

A-1
NA-1
A-1
e-9.5-mRNA-1
e-9.0-mRNA-1
421.9-mRNA-1
19-mRNA-1
e-8.23-mRNA-1
e-302.7-mRNA-1
e-0.2-mRNA-1
NA-1
NA-1
NA-1
e-9.5-mRNA-1
NA-1
NA-1
NA-1

NA-1
-1
NA-1
-1
A-1

A-1

NA-1

NA-1
NA-1
29-mRNA-1
NA-1
-1
NA-1
-1
A-1
e-323.6-mRNA-1
e-363.5-mRNA-1
e-311.6-mRNA-1

NA-1
A-1

NA-1
NA-1
NA-1
A-1
NA-1

A-1
-370.13-mRNA-1

.11-mRNA-1
.11-mRNA-1

.13-mRNA-1
NA-1

NA-1
395.14-mRNA-1
-342.12-mRNA-1

20-mRNA-1
A-1

NA-1
-1
16.5-mRNA-1
NA-1
NA-1

A-1

.17-mRNA-1

A-1
-290.7-mRNA-1
e-0.0-mRNA-1

NA-1
NA-1
-148.8-mRNA-1
NA-1
NA-1

\-1
\-1
IA-1

IA-1
IA-1
IA-1
IA-1
IA-1

IA-1
\-1
\-1
IA-1
\-1
\-1
IA-1
IA-1
\-1
\-1
\-1
17-mRNA-1
4.16-mRNA-1

16-mRNA-1
IA-1
-1
\-1
IA-1
\-1
\-1
\-1

IA-1
\-1

\-1
IA-1
IA-1
IA-1
\-1
\-1
\-1
\-1

e-357.1-mRNA-1
e-286.7-mRNA-1
e-93.1-mRNA-1
NA-1
A-1
NA-1

NA-1

e-48.13-mRNA-1

29-mRNA-1

A-1

A-1
-1
-1
e-278.5-mRNA-1

NA-1

A-1
A-1
A-1
NA-1
NA-1

-1
A-1
A-1
NA-1
NA-1
NA-1
NA-1
NA-1
A-1
NA-1
NA-1

A-1

A-1
A-1
NA-1

NA-1
A-1
A-1

NA-1
NA-1
NA-1
A-1

NA-1
-1
NA-1
A-1
e-319.3-mRNA-1
e-311.4-mRNA-1
e-311.3-mRNA-1
e-299.0-mRNA-1
e-185.0-mRNA-1
NA-1
249.7-mRNA-1
e-0.0-mRNA-1
e-95.5-mRNA-1

e-232.0-mRNA-1
e-66.5-mRNA-1
e-95.7-mRNA-1
e-26.0-mRNA-1
e-45.7-mRNA-1
e-315.7-mRNA-1
e-84.16-mRNA-1
e-163.6-mRNA-1
e-16.4-mRNA-1
e-55.2-mRNA-1
e-326.0-mRNA-1

Click here to download Table Extended_Data_Table5.xlsx

Extended Data Table 5. Anthocyanin and chlorogenic acid biosynthesis genes witl

Phenylalanine ammonia-lyase (PAL)
maker-VaccDscaff10-augustus-gene-222.29-mRNA-1
maker-VaccDscaff10-augustus-gene-222.30-mRNA-1
augustus_masked-VaccDscaff10-processed-gene-225.6-mRNA-1
maker-VaccDscaff1-augustus-gene-186.19-mRNA-1
maker-VaccDscaff1-augustus-gene-187.25-mRNA-1
maker-VaccDscaff1-augustus-gene-185.22-mRNA-1
maker-VaccDscaff5-snap-gene-236.31-mRNA-1
maker-VaccDscaff5-augustus-gene-235.33-mRNA-1
maker-VaccDscaff5-snap-gene-236.34-mRNA-1
maker-VaccDscaff8-augustus-gene-235.22-mRNA-1
maker-VaccDscaff8-augustus-gene-235.20-mRNA-1
maker-VaccDscaff8-augustus-gene-236.15-mRNA-1

4-hydroxycinnamoyl-CoA ligase (CoAl_4CL)
maker-VaccDscaff26-snap-gene-61.70-mRNA-1
maker-VaccDscaff26-snap-gene-61.68-mRNA-1
maker-VaccDscaff26-augustus-gene-61.51-mRNA-1
maker-VaccDscaff26-augustus-gene-61.53-mRNA-1
snap_masked-VaccDscaff29-processed-gene-61.34-mRNA-1
maker-VaccDscaff29-snap-gene-61.44-mRNA-1
maker-VaccDscaff29-augustus-gene-61.39-mRNA-1
snap_masked-VaccDscaff33-processed-gene-276.28-mRNA-1
snap_masked-VaccDscaff33-processed-gene-276.26-mRNA-1
snap_masked-VaccDscaff33-processed-gene-276.24-mRNA-1
snap_masked-VaccDscaff33-processed-gene-276.25-mRNA-1

Cinnamate 4-hydroxylase (C4H)
maker-VaccDscaff11-snap-gene-342.44-mRNA-1
maker-VaccDscaff11-augustus-gene-343.38-mRNA-1
snap_masked-VaccDscaff11-processed-gene-343.15-mRNA-1
maker-VaccDscaff24-snap-gene-59.54-mRNA-1
maker-VaccDscaff24-augustus-gene-58.38-mRNA-1
maker-VaccDscaff47-augustus-gene-123.34-mRNA-1
snap_masked-VaccDscaff47-processed-gene-123.17-mRNA-1

Hydroxycinnamoyl-CoA shikimate/quinate hydroxycinnamoyltransferase (HCT)
maker-VaccDscaff1-augustus-gene-69.29-mRNA-1
maker-VaccDscaff1-augustus-gene-70.25-mRNA-1
maker-VaccDscaff37-snap-gene-282.24-mRNA-1
maker-VaccDscaff37-snap-gene-282.25-mRNA-1
maker-VaccDscaff37-snap-gene-281.44-mRNA-1

maker-VaccDscaff37-augustus-gene-283.26-mRNA-1
maker-VaccDscaff37-snap-gene-285.29-mRNA-1
augustus_masked-VaccDscaff37-processed-gene-285.4-mRNA-1
maker-VaccDscaff37-augustus-gene-285.28-mRNA-1
augustus_masked-VaccDscaff38-processed-gene-2.16-mRNA-1
augustus_masked-VaccDscaff38-processed-gene-5.8-mRNA-1
maker-VaccDscaff38-snap-gene-4.32-mRNA-1
maker-VaccDscaff38-augustus-gene-2.42-mRNA-1
augustus_masked-VaccDscaff38-processed-gene-6.0-mRNA-1
maker-VaccDscaff38-augustus-gene-2.44-mRNA-1
augustus_masked-VaccDscaff38-processed-gene-5.5-mRNA-1
augustus_masked-VaccDscaff38-processed-gene-5.3-mRNA-1
maker-VaccDscaff38-snap-gene-2.54-mRNA-1
maker-VaccDscaff38-augustus-gene-0.18-mRNA-1
maker-VaccDscaff38-augustus-gene-0.16-mRNA-1
maker-VaccDscaff38-snap-gene-1.17-mRNA-1
maker-VaccDscaff39-augustus-gene-42.23-mRNA-1
maker-VaccDscaff39-augustus-gene-43.27-mRNA-1
snap_masked-VaccDscaff39-processed-gene-42.20-mRNA-1
maker-VaccDscaff6-augustus-gene-402.29-mRNA-1
maker-VaccDscaff6-augustus-gene-399.26-mRNA-1
maker-VaccDscaff6-snap-gene-399.34-mRNA-1
augustus_masked-VaccDscaff6-processed-gene-398.6-mRNA-1
augustus_masked-VaccDscaff6-processed-gene-400.9-mRNA-1
augustus_masked-VaccDscaff6-processed-gene-403.6-mRNA-1
snap_masked-VaccDscaff6-processed-gene-405.14-mRNA-1
maker-VaccDscaff6-augustus-gene-404.22-mRNA-1
maker-VaccDscaff6-snap-gene-404.30-mRNA-1
maker-VaccDscaff6-snap-gene-404.28-mRNA-1
maker-VaccDscaff6-augustus-gene-405.28-mRNA-1

4-coumaric acid 3`-hydroxylase (C3H)
maker-VaccDscaff10-snap-gene-65.37-mRNA-1
maker-VaccDscaff10-snap-gene-65.38-mRNA-1
snap_masked-VaccDscaff10-processed-gene-64.27-mRNA-1
snap_masked-VaccDscaff10-processed-gene-64.26-mRNA-1
maker-VaccDscaff1-snap-gene-373.43-mRNA-1
maker-VaccDscaff1-augustus-gene-373.37-mRNA-1
maker-VaccDscaff1-snap-gene-373.42-mRNA-1
snap_masked-VaccDscaff1-processed-gene-373.19-mRNA-1
maker-VaccDscaff5-snap-gene-352.42-mRNA-1
maker-VaccDscaff5-augustus-gene-352.30-mRNA-1
maker-VaccDscaff8-snap-gene-79.46-mRNA-1

maker-VaccDscaff8-augustus-gene-79.42-mRNA-1
maker-VaccDscaff8-augustus-gene-79.43-mRNA-1
maker-VaccDscaff8-augustus-gene-79.41-mRNA-1

Hydroxycinnamoyl-CoA quinate hydroxycinnamoyltransferase (HQT)
augustus_masked-VaccDscaff38-processed-gene-2.16-mRNA-1
augustus_masked-VaccDscaff38-processed-gene-5.8-mRNA-1
maker-VaccDscaff38-snap-gene-4.32-mRNA-1
maker-VaccDscaff38-augustus-gene-2.42-mRNA-1
augustus_masked-VaccDscaff38-processed-gene-6.0-mRNA-1
maker-VaccDscaff38-augustus-gene-2.44-mRNA-1
augustus_masked-VaccDscaff38-processed-gene-5.5-mRNA-1
augustus_masked-VaccDscaff38-processed-gene-5.3-mRNA-1
maker-VaccDscaff38-snap-gene-2.54-mRNA-1
augustus_masked-VaccDscaff39-processed-gene-45.1-mRNA-1
augustus_masked-VaccDscaff39-processed-gene-44.7-mRNA-1
maker-VaccDscaff39-augustus-gene-47.29-mRNA-1
maker-VaccDscaff39-snap-gene-44.38-mRNA-1
snap_masked-VaccDscaff39-processed-gene-47.15-mRNA-1
maker-VaccDscaff39-snap-gene-47.33-mRNA-1
augustus_masked-VaccDscaff39-processed-gene-46.0-mRNA-1
maker-VaccDscaff39-augustus-gene-43.31-mRNA-1
maker-VaccDscaff39-augustus-gene-44.31-mRNA-1
augustus_masked-VaccDscaff39-processed-gene-44.5-mRNA-1

Chalcone synthase (CHS)
maker-VaccDscaff12-snap-gene-318.35-mRNA-1
augustus_masked-VaccDscaff12-processed-gene-318.4-mRNA-1
maker-VaccDscaff12-snap-gene-319.32-mRNA-1
maker-VaccDscaff12-snap-gene-319.33-mRNA-1
snap_masked-VaccDscaff12-processed-gene-318.19-mRNA-1
maker-VaccDscaff13-augustus-gene-46.32-mRNA-1
snap_masked-VaccDscaff13-processed-gene-46.30-mRNA-1
augustus_masked-VaccDscaff23-processed-gene-74.2-mRNA-1
maker-VaccDscaff23-snap-gene-74.34-mRNA-1
maker-VaccDscaff25-augustus-gene-173.26-mRNA-1
maker-VaccDscaff25-augustus-gene-174.22-mRNA-1
snap_masked-VaccDscaff3-processed-gene-334.15-mRNA-1
maker-VaccDscaff3-augustus-gene-334.17-mRNA-1
snap_masked-VaccDscaff40-processed-gene-213.18-mRNA-1
maker-VaccDscaff40-snap-gene-213.40-mRNA-1
snap_masked-VaccDscaff40-processed-gene-213.15-mRNA-1
snap_masked-VaccDscaff40-processed-gene-213.14-mRNA-1

maker-VaccDscaff41-snap-gene-198.38-mRNA-1
maker-VaccDscaff41-snap-gene-198.34-mRNA-1
maker-VaccDscaff41-snap-gene-198.39-mRNA-1

Chalcone--flavonone isomerase (CHI)
maker-VaccDscaff25-snap-gene-101.17-mRNA-1
maker-VaccDscaff25-augustus-gene-101.12-mRNA-1

Flavonoid 3',5'-hydroxylase (F3'5'H)
maker-VaccDscaff22-augustus-gene-138.42-mRNA-1
snap_masked-VaccDscaff22-processed-gene-138.31-mRNA-1
augustus_masked-VaccDscaff22-processed-gene-138.4-mRNA-1
maker-VaccDscaff22-snap-gene-138.49-mRNA-1
maker-VaccDscaff22-augustus-gene-138.38-mRNA-1
maker-VaccDscaff43-snap-gene-230.38-mRNA-1
augustus_masked-VaccDscaff43-processed-gene-231.0-mRNA-1
maker-VaccDscaff43-augustus-gene-230.31-mRNA-1
maker-VaccDscaff43-augustus-gene-231.35-mRNA-1
maker-VaccDscaff46-snap-gene-18.55-mRNA-1
maker-VaccDscaff46-snap-gene-18.56-mRNA-1
maker-VaccDscaff46-augustus-gene-18.50-mRNA-1
maker-VaccDscaff46-augustus-gene-18.48-mRNA-1

Dihydroflavonol reductase (DFR)
maker-VaccDscaff30-augustus-gene-208.35-mRNA-1
maker-VaccDscaff30-augustus-gene-208.37-mRNA-1

Anthocyanidin reductase (ANR)
maker-VaccDscaff15-augustus-gene-178.21-mRNA-1
maker-VaccDscaff19-augustus-gene-256.19-mRNA-1
maker-VaccDscaff24-augustus-gene-201.24-mRNA-1

UDP glucose:flavonoid 3-O-glucosyl transferase (UFGT)
maker-VaccDscaff28-augustus-gene-290.32-mRNA-1
maker-VaccDscaff28-augustus-gene-290.34-mRNA-1
maker-VaccDscaff34-augustus-gene-294.29-mRNA-1
maker-VaccDscaff34-augustus-gene-294.28-mRNA-1
maker-VaccDscaff37-augustus-gene-300.29-mRNA-1
maker-VaccDscaff37-augustus-gene-300.34-mRNA-1
maker-VaccDscaff38-augustus-gene-9.29-mRNA-1
maker-VaccDscaff38-augustus-gene-9.33-mRNA-1
maker-VaccDscaff39-augustus-gene-2.34-mRNA-1
augustus_masked-VaccDscaff39-processed-gene-2.0-mRNA-1

maker-VaccDscaff6-augustus-gene-420.31-mRNA-1
maker-VaccDscaff6-augustus-gene-420.36-mRNA-1

Anthocyanin O-methyltransferase (OMT)
snap_masked-VaccDscaff13-processed-gene-342.21-mRNA-1
maker-VaccDscaff13-snap-gene-341.30-mRNA-1
snap_masked-VaccDscaff13-processed-gene-341.19-mRNA-1
maker-VaccDscaff30-snap-gene-57.21-mRNA-1
augustus_masked-VaccDscaff30-processed-gene-58.9-mRNA-1
maker-VaccDscaff30-augustus-gene-59.19-mRNA-1
maker-VaccDscaff30-augustus-gene-56.12-mRNA-1
snap_masked-VaccDscaff32-processed-gene-23.13-mRNA-1
maker-VaccDscaff32-snap-gene-23.20-mRNA-1
augustus_masked-VaccDscaff32-processed-gene-21.11-mRNA-1
maker-VaccDscaff32-augustus-gene-21.27-mRNA-1
augustus_masked-VaccDscaff42-processed-gene-262.6-mRNA-1
maker-VaccDscaff42-augustus-gene-261.27-mRNA-1

n tandem duplicates.

Manuscript

Click here to access/download
Supplementary Material
Supplement-BB-Manuscript-GigaScience.pdf

Manuscript

Click here to download Personal Cover GigaScience_Cover_Letter_BB_Manuscript.pdf

Patrick P. Edger, Ph.D.
Department of Horticulture
Michigan State University
East Lansing, MI 48824
edgerpat@msu.edu

Dear Dr. Hans Zauner :

Thank you for considering our manuscript titled "**Subgenome dominance and evolution of phytonutrient pathways in allopolyploid blueberry** " for publication in *GigaScience*.

The cultivated blueberry (*Vaccinium corymbosum*), besides having a relatively short domestication history (~100 years), has the distinction of being only one of three commercially valuable fruit crops with wild progenitors native to North America. The others are the cranberry (*V. macrocarpon*) and the garden strawberry (*Fragaria x ananassa*). As a result for the demand for fresh blueberries as a "superfruit", highbush blueberry production has increased 600% during the past three decades and steadily grown to a multi-billion dollar industry. However, in spite of its economic importance and health benefit potential, breeding efforts to improve fruit quality traits in blueberry have been slow due in large part to the lack of genetic information and genomic resources.

Here, we present the first chromosome-scale genome assembly of a tetraploid highbush blueberry. The haplotype-phased assembly consists of 48 pseudomolecules with ~1.68Gb of assembled sequence, ~1.29% gaps, and an average of 32,140 protein coding genes per haplotype (128,559 total). We leveraged this genome to gain insights into the underlying genetics of fruit development and biosynthesis of metabolites contributing to superior fruit quality. Our analyses also revealed that highbush blueberry arose from the hybridization of two distinct species (i.e. allopolyploidy) based on the sequence divergence, unique insertion of transposable elements and gene expression dominance observed between parental subgenomes. The emergence of dominant subgenome is predicted to resolve genetic and epigenetic conflicts that arise following the merger of distinct parental genomes into a single nucleus. Our analyses revealed that the dominantly expressed subgenome exhibits spatial-temporal specificity. For example, thedominant subgenome in most surveyed tissues (e.g. flower buds) becomes the least expressed during fruit development. This has serious implications in guiding crop improvement efforts in highbush blueberry. Thus, we anticipate that this genome, combined with our insights into subgenome dominance, will greatly accelerate molecular breeding efforts in the cultivated highbush blueberry.

Should you agree to consider our manuscript for review in *GigaScience*, we have identified potential reviewers with expertise and scientific interests relevant to our studies and analyses:

• Dr. Ray Ming, Univ. of Illinois (rming@life.uiuc.edu). Dr. Ming is an expert in plant genomics, including polyploid genome assembly, and evolutionary genetics in fruit crops.

• Dr. Jeffrey Doyle, Cornell Univ. (jjd5@cornell.edu). Dr. Doyle is an expert in plant molecular systematics, molecular evolution and comparative genomics of polyploids.
• Dr. J. Chris Pires, Univ. of Missouri (piresjc@missouri.edu). Dr. Pires is an expert in the field of plant genetics and genomics of resynthesized and neopolyploids.

Thank you for your considering this manuscript for publication in *GigaScience*.

Sincerely,

Patrick P. Edger

Patrick P. Edger
