# Supplementary material for: Haplotype-phased genome and evolution of phytonutrient pathways of tetraploid blueberry
Source: Gigascience. 2019 Jan 31;8(3):giz012. doi: 10.1093/gigascience/giz012 (PMC6423372; doi:10.1093/gigascience/giz012)
Supplement: GIGA-D-18-00370_Revision_1.pdf [file giz012_giga-d-18-00370_revision_1.pdf]

GigaScience

Haplotype-phased genome and evolution of phytonutrient pathways of tetraploid blueberry

--Manuscript Draft--

**Manuscript Number:**	GIGA-D-18-00370R1
**Full Title:**	Haplotype-phased genome and evolution of phytonutrient pathways of tetraploid blueberry
**Article Type:**	Research
**Funding Information:**	National Institute of Food and Agriculture (1015241) — Dr Patrick Edger<br>National Institute of Food and Agriculture (1009804) — Dr Patrick Edger<br>National Institute of Food and Agriculture (1016057) — Dr Jennifer Wisecaver<br>National Natural Science Foundation of China (31560302) — Dr Zhiyong Xiong<br>Inner Mongolia University (5163901) — Dr Zhiyong Xiong
**Abstract:**	Highbush blueberry (Vaccinium corymbosum) has long been consumed for its unique flavor and composition of health-promoting phytonutrients. However, breeding efforts to improve fruit quality in blueberry have been greatly hampered by the lack of adequate genomic resources and a limited understanding of the underlying genetics encoding key traits. The genome of highbush blueberry has been particularly challenging to assemble in large part to its polyploid nature and genome size. Here, we present a chromosome-scale and haplotype-phased genome assembly of the cultivar 'Draper', which has the highest antioxidant levels among a diversity panel of seventy-one cultivars and thirteen wild Vaccinium species. We leveraged this genome, combined with gene expression and metabolite data measured across fruit development, to identify candidate genes involved in the biosynthesis of important phytonutrients among other metabolites associated with superior fruit quality. Genome-wide analyses revealed that both polyploidy and tandem gene duplications modified various pathways involved in the biosynthesis of key phytonutrients. Furthermore, gene expression analyses hint at the presence of a spatial-temporal specific dominantly expressed subgenome including during fruit development. These findings and the reference genome will serve as a valuable resource to guide future genome-enabled breeding of important agronomic traits in highbush blueberry.
**Corresponding Author:**	Patrick Edger<br>Michigan State University<br>UNITED STATES
**Corresponding Author Secondary Information:**	
**Corresponding Author's Institution:**	Michigan State University
**Corresponding Author's Secondary Institution:**	
**First Author:**	Marivi Colle
**First Author Secondary Information:**	
**Order of Authors:**	Marivi Colle<br>Courtney Leisner<br>Ching Man Wai<br>Shujun Ou<br>Kevin Bird

Powered by Editorial Manager® and ProduXion Manager® from Aries Systems Corporation

	Jie Wang
	Jennifer Wisecaver
	Alan Yocca
	Gil Ben-Zvi
	Elizabeth Alger
	Haibao Tang
	Zhiyong Xiong
	Pete Callow
	Avital Brodt
	Kobi Baruch
	Kevin Childs
	Lily Shiue
	Guo-qing Song
	Anthony Schilmiller
	Nicholi Vorsa
	Robert VanBuren
	Robin Buell
	Ning Jiang
	Patrick Edger
	Thomas Swale
**Order of Authors Secondary Information:**	
**Response to Reviewers:**	Dear Reviewers and Editors:<br>Thank you for a thorough and very thoughtful review of our manuscript. We have made several changes to our manuscript to address points raised by Reviewer #2, which truly helped improve the overall quality of the manuscript. This includes slightly changing the title of the manuscript based on comment #4 to “Haplotype-phased genome and evolution of phytonutrient pathways of tetraploid blueberry”. We feel that this new title more accurately reflects the highlights of the paper.<br><br>Best,<br><br>Patrick Edger<br><br>Reviewer reports:<br>Reviewer #1: This is a well-prepared manuscript. The results provide an important platform for further research<br>on this and related species. The specific gene groups studied are appropriate to the unique features of this system.<br>The observations on sub genome contributions are also important contributions to understanding of polyploid systems.<br><br>Response: Thank you for the positive feedback on our manuscript.<br><br>Reviewer #2: The manuscript presents a chromosome-scale haplotype phased genome assembly of<br>highbush blueberry which is of high economic importance mainly due to its composition of health<br>promoting phytonutrients. Genes and pathways associated with antioxidant and sugar levels in blueberry<br>fruits were analyzed in more detail. Overall this work provides a valuable new genomic reference for

blueberry and enables future studies on the genome of blueberry for research and breeding purposes.
Furthermore, the findings give insights into expression patterns of genes associated with fruit ripening
and antioxidant biosynthesis as well as the expansion of gene families related to these traits. Finally, it is
shown that blueberry is an allopolyploid species with subgenome dominance. The presented reference
genome sequence is of high quality and both the gene annotation and TE analysis are sound.
Transcriptome analyses were properly carried out, although many of the results are mainly confirming
prior assumptions. The manuscript could further be improved by considering these points:
Major:
1. the authors claim that there is a high average sequence similarity among syntenic homeologous genes
(96.3%) and that there is a divergence between syntenic homeologous genes of ~0.036 per synonymous
site. They thus conclude that blueberry is allopolyploid. It is not obvious how this conclusion was made
and it should be further elaborated on the connection between allopolyploidy and the mentioned numbers.

Response: We thank you for this comment and have revised the manuscript to help clarify this to the
reader. We completely agree that additional details were needed. Furthermore, we added a time estimate
for the polyploid event based on dating unique LTR insertions.

2. in the case of genes involved in anthocyanin and chlorogenic acid biosynthesis, various tandem
duplicates were identified. Are these all functional genes and not pseudogenes? Also, a more detailed
description or analysis of the expression patterns of tandem duplicated genes and the mentioned gene
family expansions would be desirable. This would shed light on possible dosage effects and put the
analysis into a biological context with the other transcriptome analyses.

Response: We have added additional details regarding the expression of tandem duplicated genes to the
manuscript in the 'Expansion of antioxidant-related gene families through tandem duplication' section
and have revised the 'Transcriptome assembly and gene-expression analysis' section in the methods. In
short, 83.4% the tandem duplicates were expressed in at least one transcriptome library with 73.5%
expressed in at least one of the fruit transcriptome libraries. We agree that additional transcriptome
analyses with more diverse libraries are needed to provide further insights into the dosage effects of these
duplicate genes and their possible involvement in fruit development and quality traits. We are currently
working on generating additional datasets and analyses which will be included in a follow-up manuscript
later next year. The goal for this manuscript was to provide a new genomic resource and research findings
to the blueberry community to enable various future research efforts including to investigate the evolution
of duplicate genes.

3. in that context, and especially for an allopolyploid, a more detailed analysis of pseudogenes and gene

Powered by Editorial Manager® and ProduXion Manager® from Aries Systems Corporation

fragments would be very interesting.

Response: We recognize the importance of differentiating pseudogenes from “real” genes as some
pseudogenes are linked to certain biological functions. There are available tools to predict pseudogenes,
however, they have limitations and characterizing a pseudogene remains difficult given its high similarity
to the “real” genes. Also, to verify if a pseudogene is functional or not, we need to set up experiments to
examine the presence or absence of biological function of a pseudogene. At this point, we believe that
performing such extensive experiments is beyond the scope of this paper. However, we agree with you
that this would likely yield very interesting results.

4. the authors found a difference in gene expression levels between the two subgenomes and hypothesize
this might be due to differences in transposon density around homeologous genes. Since transposon
density was also measured, it should be included in the manuscript whether or not TE-density correlates
with subgenome specific gene expression levels.

Response: Transposable element (TE) content differences among homoeologous genes may not play
major role in highbush blueberry. Each of the homoeologous chromosomes have relatively similar total
TE content. For example, there is only a ~1.85% differences in TE content between chromosomes 1, 13,
25 and 37. Chromosomes 1 and 13 having the most and least amount of TEs, respectively. The most
dominantly expressed subgenome typically has the lowest amount of methylated TEs near genes. We are
currently in the process of generating the datasets to look at the methylation status of individual TEs in
different organs and developmental stages and to compare this to gene expression patterns. These
analyses will take us several months (up to a year) to complete and thus will be included as part of followup
manuscript. The observed expression patterns shown in Figure 2B are very interesting. These are very
different from what has been observed in most other allopolyploids exhibiting subgenome expression
dominance - a single dominant subgenome. However, as you point out below, these findings are quite
preliminary (i.e. “hints”). For these reasons, we have decided to change the title of the manuscript and
have revised the manuscript to state that these findings are preliminary and require follow-up studies.

5. The mapping of reads retrieved from RNA-Seq data to the genome was performed uniquely. Since
highly similar genomic regions are in general problematic when performing gene expression studies, it
should be included in the description of the method whether non-unique reads where mapped randomly or
excluded from the mapping.

Response: We excluded the non-unique reads from the analyses. We revised the ‘Transcriptome
assembly and gene-expression analysis’ section in the methods to include this additional information.

Powered by Editorial Manager® and ProduXion Manager® from Aries Systems Corporation

6. gene expression was analyzed across 14 different samples and total gene expression values were used
to compare total gene expression across haplotypes. However, replicates were only available for fruit
samples but not for all other samples. Hence, the findings here should be described as hints. Moreover, it
should be explained how the fruit samples were treated in this analysis (was only one replicate used or the
average count of all three?).

Response: Total gene expression values for subgenome dominance analysis were derived from a single
biological replicate of different tissue types (1=flower bud; 2=flower at anthesis; 3=petal fall; 4=green
fruit; 5=pink fruit; 6=ripe fruit; 7, 8=leaf collected at 12 p.m. and 12 a.m., respectively; 9, 10, 11=methyl
jasmonate treated leaf collected after one hour, eight hours and 24 hours, respectively; 12=shoot; 13=root;
14=salt-treated root). To eliminate potential variability in gene expression due to environmental effects,
we only analyzed the data from tissues collected from 'Draper' grown in the growth chamber (i.e.
identical environmental conditions). We completely agree that these findings are quite preliminary and
require follow-up analyses. Thus, we have added additional text to the manuscript to address this
important point and removed several paragraphs in the Discussion section.

7. The citations of extended table 4 and 5 in the text seem to be wrong! I believe 5 is meant to be 4 and 6
(which doesn't exist) should be 5. This needs to be fixed. In general there seems to be a problem with the
formatting ("error for extended table 2") and content of the extended tables. I would suggest to deposit
them under a public data DOI instead of having them attached to the main manuscript.

Response: We apologize for this mix-up. We have corrected Extended data tables 4, 5, and 6. We
checked Extended data table 2 and did not discover any formatting issues. The file size is a bit large, thus,
it may take a few moments for some computers to load all of its contents.

8. I recommend that the manuscript is proof read in order to improve language, sentence structure,
grammar and typing errors.

Response: We have carefully edited the manuscript to correct these various errors.

Minor:

1. I would recommend not to use the term "expressed chromosomes".

Response: We agree and have removed this term from the manuscript.

2. in order to improve understanding the authors definition of the term 'haplotype' should be included in
the manuscript since various definitions have been used in other publications.

Response: We agree and have added a definition of 'haplotype' to the manuscript.

3. The labels of the heatmaps in figure 3 are not readable. It would be nice to be able link the gene
expression to the pathway.

Powered by Editorial Manager® and ProduXion Manager® from Aries Systems Corporation

	Response: There's quite a number of genes included in the heatmap of Figure 3. Thus, the labels for<br>individual genes are quite small. We will upload a high-resolution image of Figure 3 to the project's data<br>repository in PURR.<br><br>4. The y-axis labels of figure S6-b are not readable<br><br>Response: We revised Fig. S6 and the labels are now readable.<br><br>5. Figure S3 has a very low resolution<br><br>Response: We revised Fig. S3 to improve the resolution.
Additional Information:	
Question	Response
Are you submitting this manuscript to a special series or article collection?	No
**Experimental design and statistics**<br><br>Full details of the experimental design and statistical methods used should be given in the Methods section, as detailed in our Minimum Standards Reporting Checklist. Information essential to interpreting the data presented should be made available in the figure legends.<br><br>Have you included all the information requested in your manuscript?	Yes
**Resources**<br><br>A description of all resources used, including antibodies, cell lines, animals and software tools, with enough information to allow them to be uniquely identified, should be included in the Methods section. Authors are strongly encouraged to cite Research Resource Identifiers (RRIDs) for antibodies, model organisms and tools, where possible.<br><br>Have you included the information requested as detailed in our Minimum Standards Reporting Checklist?	Yes
**Availability of data and materials**	Yes

All datasets and code on which the conclusions of the paper rely must be either included in your submission or deposited in publicly available repositories (where available and ethically appropriate), referencing such data using a unique identifier in the references and in the "Availability of Data and Materials" section of your manuscript.<br><br>Have you have met the above requirement as detailed in our Minimum Standards Reporting Checklist?	

Powered by Editorial Manager® and ProduXion Manager® from Aries Systems Corporation

Manuscript

Click here to access/download;Manuscript;BB_Genome_Manuscript_GigaSci

Click here to view linked References

Haplotype-phased genome and evolution of phytonutrient pathways of tetraploid blueberry

Authors: Marivi Colle[a], Courtney P. Leisner[b], Ching Man Wai[a], Shujun Ou[a,c], Kevin A. Bird[a,c], Jie Wang[b], Jennifer H. Wisecaver[d,e], Alan E. Yocca[a], Elizabeth I. Alger[a], Haibao Tang[f], Zhiyong Xiong[g], Pete Callow[a], Gil Ben-Zvi[h], Avital Brodt[h], Kobi Baruch[h], Thomas Swale[i], Lily Shiue[i], Guo-qing Song[a], Kevin L. Childs[b,j], Anthony Schilmiller[k], Nicholi Vorsa[l,m], C. Robin Buell[b,n], Robert VanBuren[a,n], Ning Jiang[a,c], and Patrick P. Edger[a,c,1]

a. Department of Horticulture, Michigan State University, East Lansing, MI, USA
b. Department of Plant Biology, Michigan State University, East Lansing, MI, USA
c. Ecology, Evolutionary Biology and Behavior, Michigan State University, East Lansing, MI, USA
d. Department of Biochemistry, Purdue University, West Lafayette, IN, USA
e. Purdue Center for Plant Biology, Purdue University, West Lafayette, IN, USA
f. Human Longevity Inc: San Diego, CA
g. Key Laboratory of Herbage and Endemic Crop Biotechnology, School of Life Sciences, Inner Mongolia University, Hohhot, 010070, China
h. NRGene, Ness Ziona, 7403648 Israel
i. Dovetail Genomics, Santa Cruz, CA, USA
j. Center for Genomics Enabled Plant Science, Michigan State University, East Lansing, MI, USA
k. Mass Spectrometry & Metabolomics Core Facility, Michigan State University, East Lansing, MI, USA
l. Department of Plant Biology, Rutgers University, New Brunswick, NJ, USA
m. Philip E. Marucci Center for Blueberry and Cranberry Research and Extension, Rutgers University, Chatsworth, NJ, USA
n. Plant Resilience Institute, Michigan State University, East Lansing, MI, USA

1. Author for correspondence: edgerpat@msu.edu

Abstract:

Background: Highbush blueberry (*Vaccinium corymbosum*) has long been consumed for its unique flavor and composition of health-promoting phytonutrients. However, breeding efforts to improve fruit quality in blueberry have been greatly hampered by the lack of adequate genomic resources and a limited understanding of the underlying genetics encoding key traits. The genome of highbush blueberry has been particularly challenging to assemble in large part to its polyploid nature and genome size.

Findings: Here, we present a chromosome-scale and haplotype-phased genome assembly of the cultivar 'Draper', which has the highest antioxidant levels among a diversity panel of seventy-one cultivars and thirteen wild *Vaccinium* species. We leveraged this genome, combined with gene expression and metabolite data measured across fruit development, to identify candidate genes involved in the biosynthesis of important phytonutrients among other metabolites associated with superior fruit quality. Genome-wide analyses revealed that both polyploidy and tandem gene duplications modified various pathways involved in the biosynthesis of key phytonutrients. Furthermore, gene expression analyses hint at the presence of a spatial-temporal specific dominantly expressed subgenome including during fruit development.

Conclusions: These findings and the reference genome will serve as a valuable resource to guide future genome-enabled breeding of important agronomic traits in highbush blueberry.

Introduction:

Since domestication efforts began in the early 1900s [1], highbush blueberry (*Vaccinium corymbosum* L.) has rapidly become a high value fruit crop worldwide [2–4]. Highbush blueberry, compared to hundreds of closely related blueberry species (e.g. huckleberry, *V. ovatum* Pursh; bilberry, *V. myrtillus* L.; and sparkleberry, *V. arboreum* Marshall) in the Ericaceae [5,6], is widely cultivated due to its adaptation to temperate climates, excellent fruit quality, yield and composition of phytonutrients [7]. As a result for the demand for fresh blueberries as a “superfruit” [8], highbush blueberry production has increased 600% during the past three decades and steadily grown to a multi-billion dollar industry [9]. In addition to its short domestication history, highbush blueberry is unique in being one of only three major commercially valuable fruit crops, accompanied by cranberry (*V. macrocarpon* Ait.) [10] and the garden strawberry (*Fragaria x ananassa*) [11], with wild progenitor species native to North America.

Blueberries have a single epidermal layer that expresses a rich profile of anthocyanins during ripening, which in combination with epicuticular wax, generates its characteristic ‘powdery blue’ color. The cuticular and epidermal layers contain nearly all of the phytonutrients in the fruit such as anthocyanins, proanthocyanidins and flavonols [12–14]. Previous studies on blueberry have reported that these group of compounds may have diverse health-promoting properties including controlling diabetes, improving cognitive function, and inhibiting tumor growth [15–21]. With the growing awareness of the potential health benefits of blueberry and increasing consumer demand, a primary goal of the blueberry research community is to develop cultivars with improved antioxidant levels along with other important fruit quality traits (e.g. aroma, taste, and firmness) [22]. However, despite its economic importance and health benefit potential, breeding efforts to improve fruit quality traits in blueberry have been slow due in large part to the lack of genomic resources. A draft genome for a wild diploid species ($2n=2x=24$) of blueberry was previously assembled [23]. However, this draft genome consists of a large number of scaffolds (13,757 total; N50 of ~145kb), high percentage of gaps (~27.35%) in a ~393.16Mb assembly, and most importantly, does not reflect the genome complexity of the economically important and cultivated tetraploid ($2n=4x=48$) highbush blueberry.

Here, we present the first chromosome-scale genome assembly of tetraploid highbush blueberry. The haplotype-phased assembly consists of 48 pseudomolecules with ~1.68Gb of assembled sequence, ~1.29% gaps, and an average of 32,140 protein coding genes per haplotype (128,559 total). A haplotype is the complete set of DNA within the nucleus of an individual that was inherited from one parent. We leveraged this genome to examine the origin of the polyploid event, gain insights into the underlying genetics of fruit development and to identify candidate genes involved in the biosynthesis of metabolites contributing to superior fruit quality. Furthermore, we examined gene expression patterns among the four haplotypes in highbush blueberry. This analysis uncovered the presence of spatial-temporal specific dominantly expressed subgenomes. These findings and the reference genome will serve as a powerful platform to further investigate ‘subgenome dominance’ [24–26], facilitate the discovery and analysis of genes encoding economically important traits, and ultimately enable molecular breeding efforts in blueberry.

Results

Assembly and annotation of the tetraploid highbush blueberry genome

Our goal was to obtain a high-quality reference genome for the highbush blueberry cultivar 'Draper', which is widely grown around the world due to its excellent fruit quality. We sequenced the genome using a combination of both 10X Genomics (Pleasanton, CA) and Illumina (San Diego, CA), totaling 324X coverage of the genome (Table S1). These data were assembled and scaffolded using the software package DenovoMAGIC3 (NRGene, Nes Ziona, Israel)(Table S2). The genome was further scaffolded to chromosome-scale using Hi-C data (91.4X coverage) with the HiRise pipeline (Dovetail, Santa Cruz, CA)(Figure S1 and S2). The total length of the final assembly is 1,679,081,592 bases distributed across 48 chromosome-level pseudomolecules (**Figure 1**). The final assembly size falls within the estimated genome size of 'Draper' based on flow cytometry (1.63Gb with 95% C.I. +/- 0.06Gb) (Extended Data Table 1).

The genome was annotated using a combination of evidence-based and *ab initio* gene prediction using the MAKER-P pipeline [27] (Table S3). RNAseq data from thirteen different gene expression libraries, representing unique organs, developmental stages, and treatments (Table S4), and publicly available transcriptome and expressed sequence tags (EST) data of *V. corymbosum* in NCBI were used as transcript evidence. Protein sequences from *Arabidopsis thaliana [28,29]*, *Actinidia chinensis* [30] and UniprotKB plant database were also used as evidence for genome annotation. We predicted a total of 128,559 protein-coding genes. Benchmarking Universal Single-Copy Orthologs analysis (BUSCO, RRID:SCR_015008) v.3 [31] was performed to assess the completeness of the assembly and quality of the genome annotation. The annotated gene set contains 1,394 out of 1,440 (97%) BUSCO genes (Table S5). Functional annotation was assigned using BLAST2GO [32] to reference pathways in the KEGG database [33] (Figure S3). Comparative genomic analyses assigned genes to 16,909 orthogroups shared by six phylogenetically diverse plant species including five eudicots (*A. chinensis* [30], *A. thaliana [28,29]*, *Fragaria vesca* [34], *Rubus occidentalis* [35] and *Vitis vinifera* [36]), each with distinct fruit types, and *Zea mays* [37] as the outgroup.

Transposable elements (TEs), both Class I and II, were identified and classified in the genome using the protocol described by Campbell et al. (2014) [27]. Overall, 44.3% of the blueberry genome is composed of TEs (Table S6). Consistent with previous reports [38,39], the most abundant Class I TEs were long terminal repeat retrotransposons (LTR-RTs), specifically the superfamily LTR/*Gypsy* followed by LTR/*Copia*, while for Class II transposons, the miniature inverted repeat (MITE) superfamily *hAT* was the most abundant. The quality of the genome was further assessed by examining the assembly continuity of repeat space using the LTR Assembly Index (LAI) deployed in the LTR_retriever package (v1.8) [40]. The adjusted LAI score of this blueberry genome is 14 and based on the LAI classification, this score is within the range of "reference" quality (**Figure 1**). Estimation of the regional LAI in 3Mb sliding windows also showed that assembly continuity is uniform and of high-quality across the entire genome.

Assessment of the origin of tetraploid highbush blueberry

The origin of highbush blueberry from either a single (i.e. autopolyploid) or multiple diploid progenitor species (i.e. allopolyploid) is a long-standing question [41]. Previous reports have suggested that highbush blueberry may be an autotetraploid based on the segregation ratios of certain traits [42]. However, an analysis of chromosome pairing among different cultivars revealed largely bivalent pairing during metaphase I [43], similar to patterns observed in known allopolyploids [44,45]. To gain further insights into the polyploid history of highbush blueberry, we calculated sequence similarity and synonymous substitution (*Ks;* silent mutation) rates between genes in homoeologous regions across the genome. The average sequence similarity is ~96.3% among syntenic homoeologous genes. The average *Ks* divergence between syntenic homoeologous genes is ~0.036 per synonymous site. The average Ks divergence between homoeologous genes can be used to not only identify polyploid events [46–48], but also to estimate the divergence of the diploid progenitors from their most common recent ancestor (MCRA) [49]. The Ks divergence between homoeologs in highbush blueberry is six times higher than that between orthologs of two *Arabidopsis thaliana* lines (Col and Ler; Ks of ~0.006) that diverged roughly 200,000 years ago [50]. Based on the relatively high Ks rate between homoeologous regions across the genome, this suggests that tetraploid blueberry is unlikely an autopolyploid that was formed from somatic doubling or failure during meiosis involving a single individual (parent).

Furthermore, comparative genomics revealed that homoeologous regions are highly collinear, except a few notable chromosome level translocations (**Figure 1a)**. These translocations were manually inspected and verified with both the raw sequence and Hi-C data. Rapid changes among homoeologous chromosomes is known to occur in newly formed allopolyploids [44,45,51]. We also assessed the level of similarity and content of LTR transposable elements among the four haplotypes. As the most prevalent transposable elements in plants, LTR-RTs undergo continual “bloat and purge” cycles within most plant genomes [52], resulting in a unique signature that may distinguish subgenomes in an allopolyploid. To examine the evolutionary history of LTR-RTs in the highbush blueberry genome, we calculated the mean sequence identity of LTR sequences among each of the four haplotypes (Figure S4). This analysis revealed that the majority of more recent LTRs (>96% similarity) are subgenome specific in highbush blueberry. In other words, the data suggests that LTRs proliferated independently in the genomes of each diploid progenitor (i.e. subgenome), following the divergence from their MCRA, but prior to polyploidy. The pair-wise LTR difference (d) of the two ancestors is 3.7% - 4.5%. With Jukes-Cantor correction ($K= -3/4*\ln(1-4d/3)$) and mutation rate of ($\mu = 1.3e\text{-}8$) [53], the estimated time ($T=K/2\mu$) of divergence for the diploid progenitors from their MCRA is between 1.46 to 1.78 million years ago.

Given these date estimates and the average speciation rate ($\lambda = 0.59$ per million years; [54]) for temperate angiosperms, this suggests that highbush blueberry is either an allopolyploid derived from two closely related species or an autopolyploid derived from the hybridization of two highly divergent populations of a single species. To date the most recent polyploid event in highbush blueberry, we analyzed the unique LTR insertions present in each haplotype. Based on the pair-wise LTR difference between the four haplotypes of 0.18% - 0.20%, the polyploid event occurred approximately 69 to 77 thousand years ago.

After allopolyploidization, one of the parental genomes (i.e. subgenomes) often emerges with significantly greater gene content and a greater number of more highly expressed genes [55–58]. The

emergence of a dominant subgenome in an allopolyploid is hypothesized to resolve genetic and epigenetic conflicts that may arise from the merger of highly divergent subgenomes into a single nucleus [26,59,60]. However, classic autopolyploids, formed by somatic doubling, are not expected to face these challenges or exhibit subgenome dominance since all genomic copies were contributed by a single parent [61]. This was recently supported by genome-wide analyses of a putative ancient autopolyploid (soybean; *Glycine max*) [62]. It's important to note that subgenome expression dominance could still be observed in intraspecific hybrids and autopolyploids formed by parents with highly differentiated genomes [25].

To explore this in highbush blueberry, we compared gene content and expression level patterns between homoeologous chromosomes (**Figure 2)**. While gene content levels were largely similar among homoeologous chromosomes, with a few notable exceptions (**Figure 2a**), gene expression levels were highest for one of the four chromosome copies in the majority (average 9.3 of 14) of gene expression libraries (112 of 168 comparisons, x2 test p-value < 0.001)(**Figure 2b**, Figure S5). Noteworthy, in the three fruit libraries, the most dominantly expressed often became the least expressed among the four homoeologous chromosomes (19 of 36 comparisons; x2 test p-value <0.01) or among the two lowest expressed copies (26 of 36 comparisons; x2 test p-value <0.01). The most dominantly expressed in other tissues remained so in developing fruit for only two of the chromosomes (6 and 10). These homoeologous chromosomes sets have undergone the most structural variation which may have modified gene expression patterns (**Figure 1a**). These analyses are based on a single biological replicate from a plant grown in a growth chamber. Thus, the findings reported here should be considered as preliminary. Future studies should further explore subgenome expression dominance in highbush blueberry, including at the individual homoeolog level [63,64] , with additional biological replicates and across multiple environments.

Changes in transcript abundance during blueberry fruit development

The progression of fruit development in blueberry is marked with visible external and internal morphological changes including in size and color (Figure S6a). We profiled gene expression in fruit across seven developmental stages from the earliest stage (i.e. post-fertilization) through the final stage (i.e. ripe fruit) to identify genes differentially expressed during fruit development. Distinctive transitions in gene expression were observed between early fruit growth to start of color development and complete color change to ripened fruit. We found that the majority of genes upregulated during early fruit development were involved in phenylpropanoid biosynthesis, nitrogen metabolism, as well as cutin, suberin and wax biosynthesis (Table S7a). In contrast, genes involved in starch and sugar metabolism were highly expressed at the onset of and during fruit ripening (Table S7b). Moreover, principal component analysis (PCA) showed the first two components accounted for 84% of the variation and separated the developmental stages into three groups: (early developmental stages) petal fall and small green fruit; (middle developmental stages) expanding green and pink fruit; and (late developmental stages) complete fruit color change, unripe and ripe fruit (Figure S6a & Figure S7).

Genes associated with cell division, cell wall synthesis and transport were found to be expressed the highest during the earliest developmental stages (Extended Data Table 2), which is consistent with previous work on other fruit species [65,66]. In addition to genes regulating cell proliferation, defense response related genes were also highly upregulated during the earliest developmental stages. During the

middle developmental stages, genes regulating cell expansion, seed development and secondary metabolite biosynthesis were highly expressed. During late developmental stages and as the berry transitions to ripening, late embryogenesis, transmembrane transport, defense, secondary metabolite biosynthesis and abscisic acid related genes were highly overrepresented. Blueberry is considered a climacteric fruit but unlike the ethylene-driven fruit ripening in other climacteric species, abscisic acid has been demonstrated to regulate fruit ripening in blueberry [67]. In summary, global gene expression patterns mirror the morphological and physiological changes observed during blueberry development (Figure S6a).

Antioxidant capacity in blueberry

The economic value of blueberry is largely determined by its fruit quality and nutritional value [7,18,68]. We assessed the total antioxidant capacity in mature fruit across a blueberry diversity panel and the abundance of secondary metabolites responsible for its antioxidant activity in developing fruit. A diversity panel, composed of 71 highbush blueberry cultivars and 13 wild *Vaccinium* species, was evaluated for total antioxidant capacity in mature fruit using the oxygen radical absorbance capacity (ORAC) assay [69]. Similar to previous reports [70–72], we observed a wide range in antioxidant capacity (~5 -95 nmol TE/mg FW) across cultivars, with ‘Draper’ having the highest levels of antioxidants (Figure S6b). The observed variation in antioxidants among highbush blueberry, consistent with our results, were previously shown not to correlate with fruit weight or size [73]. However, in another study, a correlation between fruit size and total anthocyanin levels was identified within a few select highbush blueberry cultivars but not across other *Vaccinium* species or blackberry [74]. This inconsistency is likely due to sample size differences between studies.

To further examine the antioxidant capacity in ‘Draper’ during fruit development, fruits from the seven aforementioned fruit developmental stages were assayed for antioxidant levels (Figure S6a). The highest level of antioxidants was observed at the earliest ‘petal fall’ stage (537 nmol TE/mg FW) (Figure S8) after which, the level of antioxidants declined during the middle and late developmental stages. This is consistent with previous reports on the antioxidant activity in blueberry during fruit maturation [75] and similar to observations in blackberry and strawberry, wherein green fruit have the highest ORAC values [76]. The antioxidant capacity in blueberry is influenced by various metabolites including anthocyanins [12,74,77]. Using the same fruit development series, we quantified anthocyanin and flavonol aglycones in ‘Draper’ using liquid chromatography-mass spectrometry (LC-MS). Overall, as the fruit changed its exocarp color from pink to dark blue during ripening, delphinidine-type anthocyanins started to accumulate and were the most abundant compound in ripe fruit (181 peak area/IS/gDW) followed by cyanidin, malvidin and petunidin (Figure S6c). Flavonols were also detected in all developmental stages with quercetin glycoside being the most abundant (88 peak area/IS/gDW), while myricetin glycoside and rutin were present at very low levels.

Blueberry also has high levels of phenolic acids and among phenolics, chlorogenic acid (CGA) was the most abundant. High levels of CGA was observed throughout fruit development with the highest accumulation detected in young fruits (Figure S6d). This correlates with the pattern of antioxidant capacity across different fruit stages suggesting that CGA is one of the major metabolites contributing to high ORAC values in young developing fruit. CGA is derived from caffeic acid and quinic acid and has

vicinal hydroxyl groups that are associated with scavenging reactive oxygen species (ROS) [78–80]. The antioxidant properties of CGA have been associated with preventing various chronic diseases [81–85].

Expression of antioxidant biosynthesis related genes

To better understand the biosynthesis of antioxidants in blueberry fruit, we identified homologs of previously characterized genes in other species involved in ascorbate, flavonols, chlorogenic acid, and anthocyanin biosynthesis (**Figure 3** & Extended Data Table 3) [67,86–88]. The key biosynthetic genes for these compounds exhibited a distinct developmental specific pattern of expression (**Figures 3c-e** & Figure S9). For example, genes involved in the conversion of leucoanthocyanidins to proanthocyanidins (e.g. *LAR* and *ANR*) are highly expressed in the earliest and middle developmental fruit stages but not in ripening fruit (**Figure 3c**; green triangle & Extended Data Table 4). Conversely, genes involved in the conversion of leucoanthocyanidins to anthocyanins (e.g. *ANS*, *UFGT* and *OMT*) were highly expressed in mature and ripe fruit but not during early fruit developmental stages (**Figure 3c**; red circle & Extended Data Table 4). Additionally, paralogs encoding the same anthocyanin pathway enzymes (e.g. FHT, OMT) and genes involved in vacuolar localization of proanthcyanidins (e.g. glutathione S-transferase and MRP-type) exhibited similar developmental stage-specific expression patterns. The expression of these biosynthetic genes is regulated by specific transcription factors [89]. For example, the transcription factor complex, MYB-bHLH-WD (MBW) regulates expression of anthocyanin biosynthetic genes in eudicots [90–93]. Using the Plant Transcription Factor Database v.4.0 [94], we identified homologs of transcription factors (TF) belonging to 55 gene families and members of some of these gene families were predicted to be involved in the developmental regulation of flavonoid biosynthesis during blueberry fruit growth (Extended Data Table 4), including *R2-R3-MYBs*, *R3-MYBs*, *bHLHs*, and *WDRs* (**Figure 3b,e**). These transcription factors also exhibit fruit development specific expression patterns.

In addition, we performed a gene co-expression network analysis to identify metamodules of genes that appear co-regulated during fruit development, specifically genes that are associated with phytonutrient biosynthesis. Our analysis identified 1,988 metamodules of co-expressed genes, of which, 428 metamodules contained at least one of the 57 Pfam domains that have been previously categorized as associated with specialized metabolic pathways in plants [95]. Our analysis revealed that 142 of 428 metamodules were more highly expressed in developing fruit compared to other plant tissues. Some metamodules showed clear trends of being highly expressed during either early or late fruit development. For example, METAMOD00377 is expressed early in fruit development and contains homologs to known anthocyanin genes *OMT*, *HCT*, *PAL*, *HQT* as well as 31 homologs to known transcription factors. In contrast, METAMOD01221 is expressed late in fruit development and contains homologs of *HCT, TT19, UFGT, OMT* and contains 10 homologs to known transcription factors. Moreover, we also examined metamodules for genes associated with other biosynthetic pathways which impart unique blueberry fruit characteristics. We identified two metamodules where genes appear to be co-regulated. Metamodule METAMOD00377 contains Pfam domains associated with terpene, saccharide, and alkaloid specialized metabolism and METAMOD01221 which contains terpene and saccharide metabolism. These metamodules contained genes that are differentially expressed during fruit development. Overall, the developmental-specific expression patterns of key biosynthetic genes and their putative transcriptional regulators emphasizes the tight regulation of production, conversion and

transport of precursor compounds that lead to the accumulation of antioxidant-related metabolites in blueberry.

Fruit aroma and the role of terpenes

The coregulation of genes involved in the biosynthesis of terpenes and saccharides during early and late fruit development described above reflects a coordinated interplay between these metabolites during fruit growth. Both terpenes and sugars contribute to the characteristic flavor of ripened fruit [96]. In blueberry, two components play a central role in flavor perception: taste, which is a balance of sweetness and acidity, and aroma. Blueberry aroma is a complex blend of volatiles which include aldehydes, esters, terpenes, ketones and alcohols [97,98]. Previous reports in blueberry showed that the aroma profile varies greatly across different blueberry ecotypes and cultivars [99–101]. For example, the aroma of highbush blueberry is primarily driven by terpene hydrocarbons (e.g. linalool, geraniol, hydroxycitronellol) and aldehydes (e.g. (E)-2-hexenal, (E)-2-hexenol, (Z)-3-hexenol) [97,102]. Both linalool and geraniol are associated with sweet floral flavor. However, linalool was reported to largely impart the characteristic blueberry flavor when combined with certain aldehydes [97].

Here we also identified and examined the expression of genes involved in the biosynthesis of linalool. Four of the linalool synthase homologs in tetraploid blueberry are highly expressed during late fruit development (Extended Data Table 5). This pattern of expression coincides with previous reports of linalool accumulation in ripened blueberry fruit [98,102,103]. On the other hand, one homolog of linalool synthase, although it was expressed during fruit growth, it did not show a clear fruit development specific pattern. Investigating the underlying factors regulating these enzymes will facilitate genetic manipulations that may lead to further improving blueberry flavor in the future.

Sugar transporters

Superior fruit quality is also associated with sugar levels [104]. During fruit ripening, sugar levels of the endocarp increases by importing hexose symplastically and/or apoplastically. Sugar transporters (i.e. *SWEET*), sucrose transporter (*SUT*) and tonoplast sugar transporter (*TST*) have been demonstrated to regulate intercellular sugar transport in phloem and fruit [105,106]. In *A. thaliana*, all clade III *SWEET* transporters play a role in sucrose transport with *AtSWEET9* primarily functioning in nectary secretion [107] while *AtSWEET15* is required for seed filling by acting with *SWEET11* and *SWEET12* [108]. In blueberry, the Clade III *SWEET transporters 9* and *10* were highly expressed during early fruit growth while clade III *SWEET transporter 15* was mainly expressed in ripe fruit (Extended Data Table 5). Interestingly, one of the blueberry *SWEET15* homologs showed a distinct pattern of expression compared to the other three homologs. To the best of our knowledge, we are the first to report on the potential role of these genes during blueberry fruit development.

In addition, homologs of *A. thaliana TST1* [109] and watermelon *ClTST1* and *ClTST3* (tonoplast sugar transporters) [106] were expressed during fruit ripening in blueberry. Elevated expression of a *ClTST1* homolog was observed throughout fruit development but the *ClTST3* homolog showed very low expression. Another gene that is highly expressed during fruit maturation is vacuolar invertase. As described in other systems [110], its upregulation during fruit ripening coincided with the breakdown of starch to sucrose or a mixture of glucose and fructose suggesting that it may be involved in the

regulation of sugar accumulation in blueberry fruit. It was previously reported that vacuolar invertase modulates hexose to sucrose ratio in ripening fruit [111]. In addition, there are also two sugar transport protein (*STP*) homologs that exhibited developmental specific expression. However, their function remains largely unknown, thus, their potential role in sugar accumulation in the developing berry requires further investigation.

Expansion of antioxidant-related gene families through tandem duplication

Tandemly duplicated genes arise as a result of unequal crossing-over or template slippage during DNA repair [112,113], exhibit high birth-death rates (i.e. predominantly young) [46] and typically are in co-regulated clusters in the genome [114]. Smaller-scale duplications [115], which includes tandem duplicates, are highly biased towards certain gene families [116] including those involved in specialized metabolism [117–119]. Furthermore, tandem duplications often results in the increased dosage of gene products [120] and may improve the metabolic flux of rate-limiting steps in certain biosynthetic pathways [121].

Most genes associated with the biosynthesis of antioxidants (CGA, flavonols, anthocyanins, proanthocyanidins) have at least one tandem duplicate present in the highbush blueberry genome, with tandem array sizes ranging between two to ten gene copies (Extended Data Table 6). The largest tandem arrays were found for *HQT* and *HCT* genes, which are co-regulated and involved in the CGA pathway (**Figure 3a)**. Differences in tandem array sizes was also observed between homoeologous chromosomes for various genes. For example, the *C3H* gene, which is involved in CGA biosynthesis (**Figure 3a**), was present on all four homoeologous chromosomes but with varying tandem array sizes. One of the homoeologous chromosomes had two copies of *C3H*, while the other three homoeologous chromosomes had four copies. This suggests that copy number differences of *C3H* among subgenomes may be due to either selection for gene duplication or loss, or in the case of allopolyploidy, may be due to preexisting gene content differences among the diploid progenitor species.

Genes in the anthocyanin pathway with other unique duplication patterns include *CHS*, *CHI*, *OMT*, and *UFGT*. The gene *CHS*, involved in the conversion of 4-Coumaryl-CoA to naringenin chalcone, has two copies and both have tandem duplicates in at least three of the homoeologous chromosomes. Interestingly, the gene *CHI*, has a single preserved tandem gene duplicate on only one of the homoeologous chromosomes. However, additional copies of *CHI* were also identified more distantly away from the syntenic ortholog on another homoeologous chromosome, likely involving a transposition event following tandem duplication. The *OMT* and *UFGT* genes all have tandem duplicates on all of the homoeologous chromosomes, although with varying array sizes, while the *ANR* gene involved in the conversion of anthocyanidin to proanthocyanidin is single copy on all homoeologous chromosomes. *DFR* gene, which is involved in the conversion of dihydroquercetin/dihyromyricetin to leucoanthocyanidin, has a single tandem duplicate on only one of the homoeologous chromosomes. These findings suggest that there may have been greater selective pressure to retain tandem duplicates for genes encoding enzymes involved in anthocyanin production than conversion to proanthocyanidins.

The vast majority of tandem duplicates are eventually lost (i.e. nonfunctionalization), however, in rare instances some may undergo functional diversification (e.g. sub- and/or neo-functionalization) [46,122]. Gene expression analysis revealed that 83.4% of the tandem duplicates were expressed in at least one

transcriptome library with 73.5% expressed in at least one of the fruit developmental stages. This suggests that a subset of these duplicate genes have either nonfunctionalized, subfunctionalized or neofunctionalized. Future studies are needed to more thoroughly investigate the functions of these genes with more diverse libraries and additional transcriptome analyses.

Discussion:

Despite the economic importance of blueberry, molecular breeding approaches to produce superior cultivars has been greatly hampered by inadequate genomic resources and a limited understanding of the underlying genetics encoding important traits. This has resulted in breeders having to solely rely on traditional approaches to generate new cultivars; each with widely varying fruit quality characteristics. For example, our analysis of a diversity panel consisting of eighty-four cultivars and wild species revealed that 'Draper' has antioxidant levels that are up to 19x higher than other cultivars. Thus, the genome of 'Draper' should serve as a powerful resource to the blueberry community for guiding future breeding efforts aimed at improving antioxidants levels among other important fruit quality traits. Furthermore, to our knowledge, this is not only the first genome assembly of the cultivated highbush blueberry, but is also the first chromosome-scale and haplotype-phased genome for any species in the order Ericales. Ericales includes several other high-value crops (e.g. tea, kiwifruit and cranberry) and wild species with unique life history traits (e.g. carnivorous, American pitcher plants; parasitic, *Sarcodes* 'snow flower' and extremophiles, 'Jacob cactus'). Thus, we anticipate that this reference genome, plus associated datasets, will be useful for a wide variety of evolutionary studies.

Here, we also leveraged the genome to identify candidate genes and pathways that encode superior fruit quality in blueberry, including those associated with pigmentation, sugar and antioxidant levels. Furthermore, we found that genes encoding key biosynthetic steps in various antioxidant pathways are enriched with tandem gene duplicates. For example, tandem gene duplications have expanded gene families that are involved in the biosynthesis of anthocyanins. This suggests that, in addition to a recent whole genome duplication, tandem duplications may have greatly contributed to the metabolic diversity observed in blueberry (as previously described in *Arabidopsis* [123]). These tandem duplicates may have evolved new functions (i.e. neofunctionalized), possibly involved in the biosynthesis of novel compounds, and/or were selected to improve the metabolic flux of specific biosynthetic steps that alter the dosage of certain endpoint metabolites [121]. Future studies are needed to further investigate the possible role of tandem duplications in having modified metabolite levels and composition in wild and cultivated blueberry.

Our analyses also revealed that highbush blueberry, a tetraploid, likely arose from the hybridization of two distinct parents, possibly allopolyploidy, based on the sequence divergence, unique transposable element insertions and subgenome expression patterns. Our analyses revealed that the subgenomes in highbush blueberry may be controlling a distinct set of genetic programs (e.g. fruit development vs mature leaves). The dominantly expressed subgenome in most surveyed tissues becomes the lowest expressed during fruit development. This observation is similar to findings in allopolyploid wheat where developmental and adaptive traits were shown to be controlled by different subgenomes [124–126]. For example, cell type- and stage-dependent subgenome expression dominance was observed in the developing wheat grain [126]. We argue that both highbush blueberry and hexaploid wheat, each now with high quality reference genomes [127], make excellent systems to further investigate these

underlying mechanisms of subgenome dominance [25]. Subgenome dominance has far reaching implications to numerous research areas including breeding efforts [58]. For example, marker assisted breeding needs to target the correct set of dominant homoeologs given the trait in polyploids that exhibit subgenome dominance. Thus, we anticipate that this genome, combined with improved insights into subgenome dominance, will greatly accelerate molecular breeding efforts in the cultivated highbush blueberry.

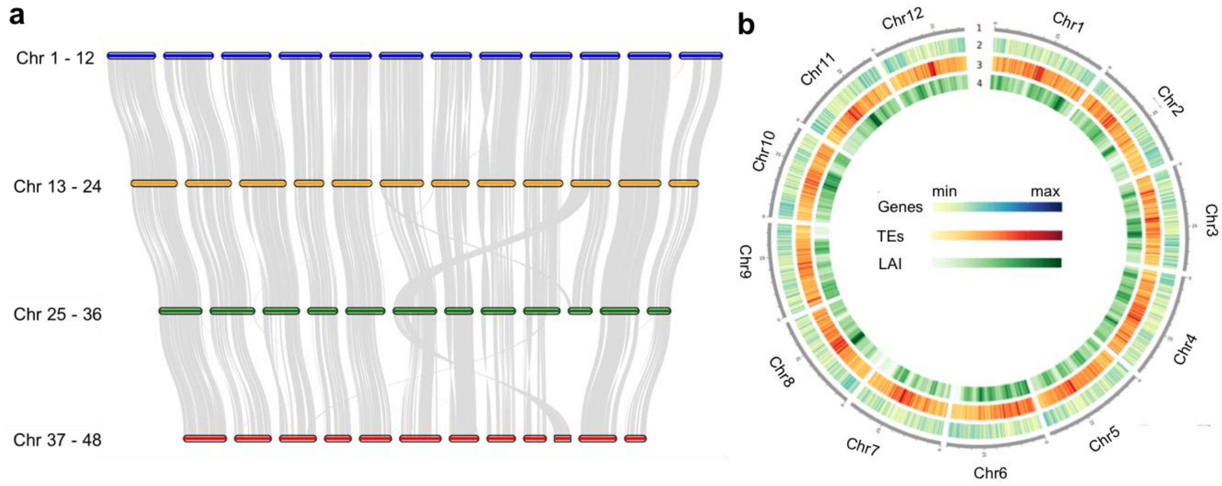

Figure 1. The haplotype-phased chromosome-scale highbush blueberry genome. (a) Collinearity among the homoeologous chromosomes. The gray lines represent conserved gene arrays between chromosomes. Chromosomes were drawn proportionally with respect to the number of genes on each chromosome. (b) Gene and transposable element (TE) density and LTR Assembly Index (LAI) in chromosomes 1-12 plotted in 300 Kb sliding window using Circos. The tracks from outside to inside are: 1= chromosomes, 2=gene density, 3=TE density, and 4= LAI score.

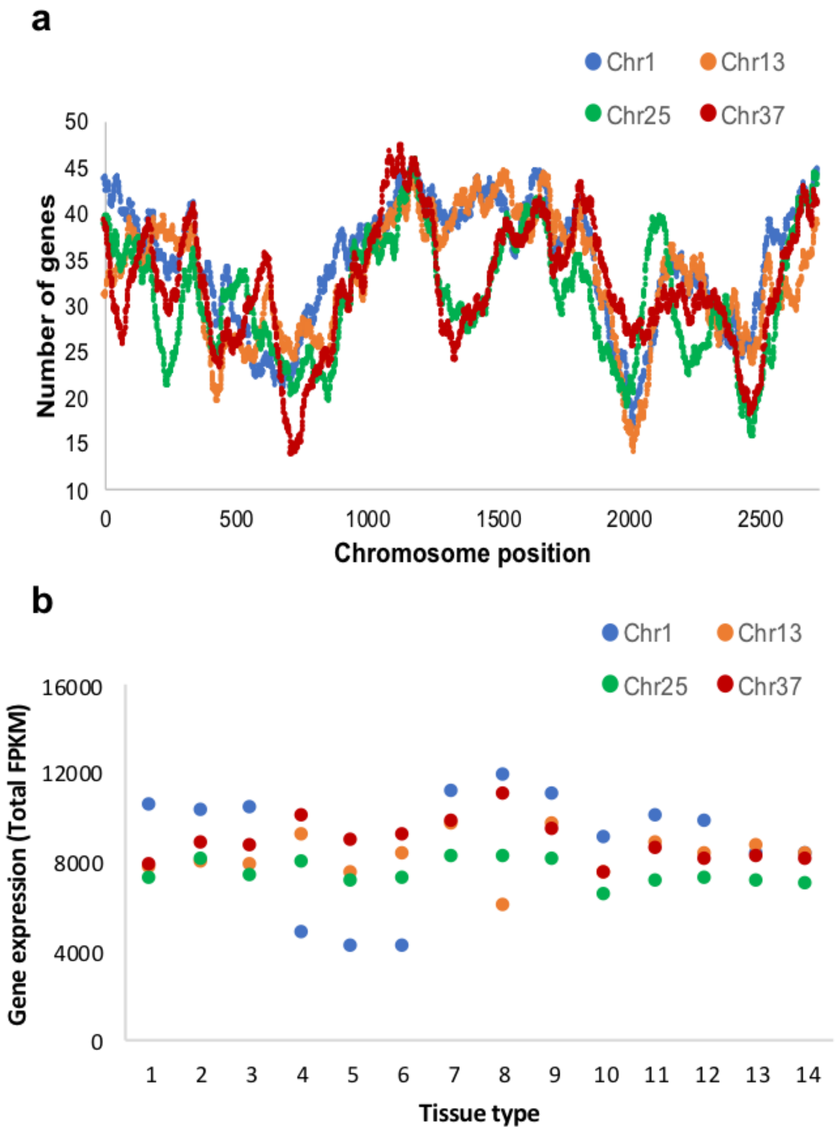

Figure 2. Assessment of the origin of polyploid blueberry. (a) Gene content comparison of homoeologous chromosomes (1, 13, 25, and 37) plotted along 2,725 collinear syntenic regions. This analysis for all 48 chromosomes can be regenerated here: https://genomevolution.org/r/12w9o (b) Gene expression comparison (FPKM; fragments per kilobase per million) among the same four homoeologous chromosomes across different blueberry tissues (1=flower bud; 2=flower at anthesis; 3=petal fall; 4=green fruit; 5=pink fruit; 6=ripe fruit; 7, 8=leaf collected at 12 p.m. and 12 a.m., respectively; 9, 10, 11=methyl jasmonate treated leaf collected after one hour, eight hours and 24 hours, respectively; 12=shoot; 13=root; 14=salt-treated root).

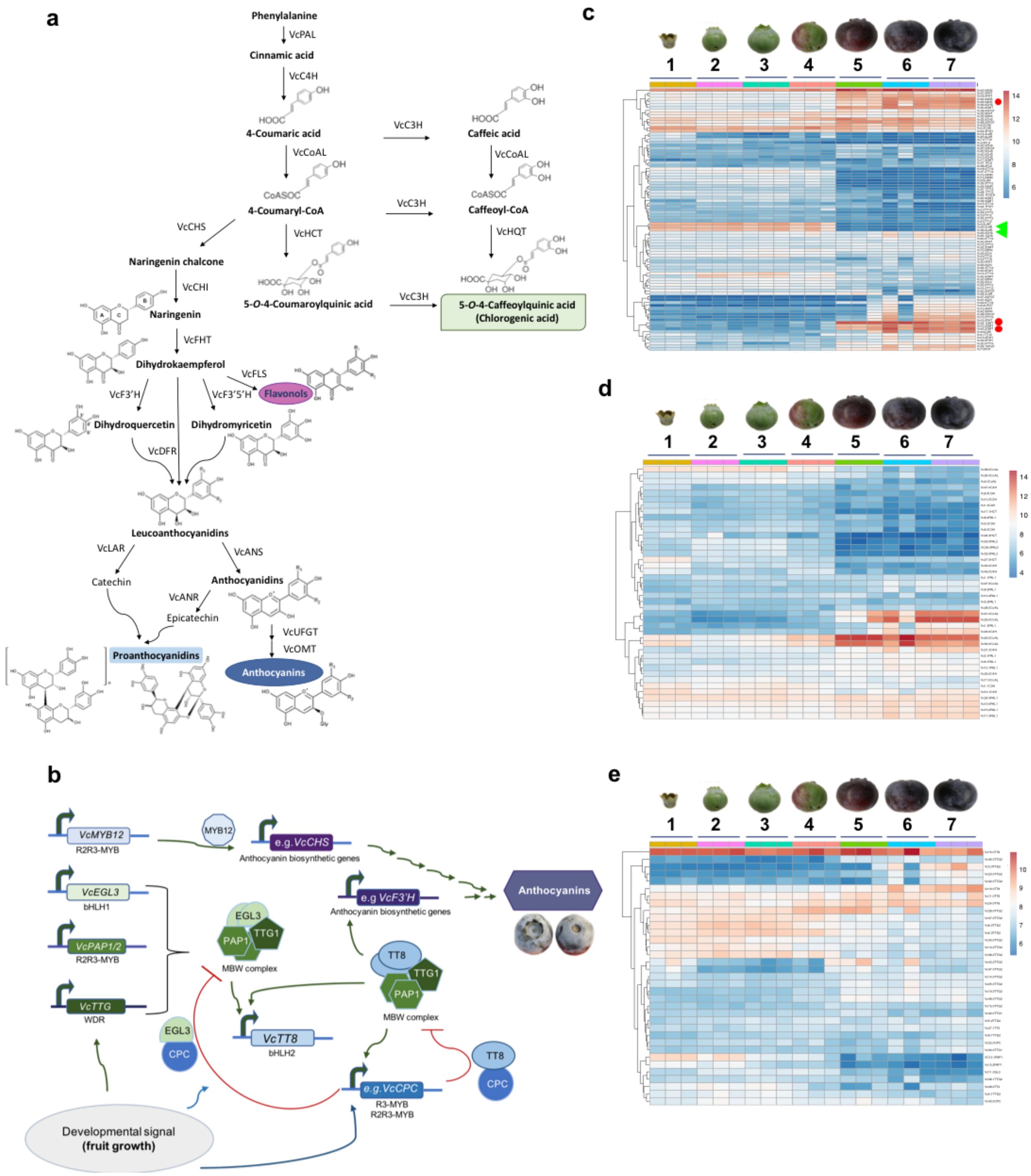

Figure 3. A schematic presentation of flavonoid biosynthesis in blueberry. (a) Predicted flavonoid biosynthetic pathway leading to production of anthocyanin. The proposed pathway is based on previously described flavonoid biosynthetic pathway in plants (Zifkin et al., 2012) and expression of predicted anthocyanin biosynthetic genes in blueberry. The core genes include phenylalanine ammonia-lyase (*PAL*), 4-hydroxycinnamoyl CoA ligase (*4CL*), trans-cinnamate 4-monooxygenase (*C4H*), cytochrome P450 98A3 (*C3H*), chalcone synthase (*CHS*), chalcone flavonone isomerase (*CHI*),

flavanone-3β-hydroxylase (*FHT*), flavanone 3-hydroxylase (*F3H*), flavonoid 3′-hydroxylase (*F3'H*), flavonoid 3′,5′-hydroxylase (*F3'5'H*), dihydroflavonol reductase (*DFR*), leucoanthocyanidin reductase (*LAR*), anthocyanidin reductase (ANR), anthocyanidin synthase (*ANS*), UDP-glucose flavonoid 3-O-glucosyl transferase (*UFGT*), anthocyanin-O-methyltransferase (*OMT*), hydroxycinnamoyl-CoA shikimate/quinate hydroxycinnamoyltransferase (*HCT*) and hydroxycinnamoyl-CoA quinate hydroxycinnamoyltransferase (*HQT*) (b) Hypothetical regulatory pathway of anthocyanin biosynthetic genes based on the proposed model by Albert et al. (2014). (c) Developmental-specific expression pattern of key anthocyanin biosynthetic gene (green triangles = examples of genes upregulated during early fruit growth; red circles = examples of genes upregulated during late fruit development) and (d) chlorogenic acid biosynthetic genes (1=petal fall, 2= small green fruit, 3= expanding green fruit, 4=pink fruit, 5= fruit color completely changed from pink to purple, 6=unripe, 7=ripe) (e) Expression profile of transcription factors predicted to regulate anthocyanin biosynthesis in blueberry. A high-resolution version of the heatmaps is available on PURR (see 'Availability of Supporting Data' section).

Materials and Methods

Plant Material

V. corymbosum cv. Draper was selected based on having the highest antioxidant levels among a diversity panel of leading cultivars and due to its overall importance to the industry (Figure S6). Furthermore, cultivar Draper was selected since germplasm is widely available to the community from blueberry nurseries. The genome size (1.63 +/- 0.06Gb) was estimated using flow cytometry with four technical replicates from Flow Cytometry Core at Benaroya Research Institute at Virginia Mason (Seattle, WA)(Extended Data Table 1).

Genomic Sequencing

High-molecular-weight genomic DNA was isolated from young leaf tissue, following a 72 hour dark treatment, using a modified nuclei preparation method [128,129]. DNA quality was verified by pulsed-field gel electrophoresis. DNA fragments longer than 50Kb were used to construct a 10X Gemcode library using the Chromium instrument (10X Genomics; Pleasanton, CA) and sequenced at HudsonAlpha Institute for Biotechnology (Huntsville, AL) on a HiSeqX system (lllumina; San Diego, CA) with paired-end 150bp reads. A total of ~95Gb (~58 fold coverage, based on an estimated genome size of 1.63Gb) of 10X Chromium library data was sequenced (Table S1). To increase sequence diversity and depth, three separate Mate-Pair (MP) libraries were constructed with 2-5Kb, 5-7Kb and 7-10Kb jumps using the Illumina Nextera Mate-Pair Sample Preparation Kit. In addition, two additional size-selected Illumina genomic libraries, ~470bp and ~800bp, were sequenced. The ~470bp and ~800bp libraries were made using the Illumina TruSeq DNA PCR-free Sample Preparation V2 kit. The ~470bp library was designed to produce 'overlapping libraries' after sequencing with paired-end 265bp reads on a lllumina Hiseq2500 system, producing 'stitched' reads of approximately 265bp to 520bp in length. The 800bp library was sequenced on an Illumina HiSeq2500 system with paired-end 160bp reads while the MP libraries were sequenced on an Illumina HiSeq4000 system with paired end 150bp reads. A total of ~433Gb (~266x fold coverage) of additional Illumina sequencing data was generated (Table S1).

Illumina library construction and sequencing was conducted at Roy J. Carver Biotechnology Center, University of Illinois at Urbana-Champaign.

Genome Assembly

The genome of ‘Draper’ was assembled using the DeNovoMAGIC software platform (NRGene, Nes Ziona, Isreal); a De Bruijn graph-based assembler designed for higher polyploid, heterozygous and/or repetitive genomes [130,131]. The Chromium 10X data was utilized to phase, elongate and validate haplotype scaffolds. Four Dovetail Hi-C libraries were prepared as described previously [132] and sequenced on an Illumina HiSeq X system with paired-end 150bp reads to a total of 90.7X physical coverage of the genome (Figure S1). The *de novo* genome assembly, raw genomic reads, and Dovetail Hi-C library reads were used as input data for HiRise, a software pipeline designed specifically for using proximity ligation data to scaffold genome assemblies [133]. Illumina genomic and Dovetail Hi-C library sequences were aligned to the draft input assembly using a modified SNAP read mapper (http://snap.cs.berkeley.edu). The separations of Dovetail Hi-C read pairs mapped within draft scaffolds were analyzed by HiRise to produce a likelihood model for genomic distance between read pairs, and the model was used to identify and break putative misjoins, and to make joins to close gaps between contigs.

Collection of blueberry tissue samples, RNA library preparation and sequencing

Plant tissue samples (flower bud, flower at anthesis, flower post-anthesis, young shoot, leaves treated with methyl jasmonate, small green fruit, expanding green fruit, pink fruit, ripe fruit and salt-treated and untreated roots) were collected from blueberry cv. Draper grown in the growth chamber (16/8 hours photoperiod; 408mE light intensity; 23/20C day/night temperature). For the fruit developmental series, three biological replicates each of berries at seven developmental stages (petal fall/cup, small green fruit, expanding green fruit, pink fruit, purple reddish fruit, purple unripe fruit and blue ripe fruit) were collected from cv. Draper in a field at the Horticulture Teaching and Research Center, MSU in July 2017. All plant tissues were immediately flash frozen in liquid nitrogen and total RNA isolation was performed using the KingFisher Pure RNA Plant kit (Thermo Fisher Scientific, MA). Isolated total RNA was quantified using a Qubit 3 Fluorometer (Thermo Fisher Scientific, MA). RNA libraries were prepared according to the KAPA mRNA HyperPrep kit protocol (KAPA Biosystems, Roche, USA). All samples were submitted to Michigan State University Research Technology Support Facility (RTSF) Genomics core and sequenced with paired-end 150bp reads on an Illumina HiSeq 4000 system (Illumina, San Diego, CA, USA).

Genome Annotation

The draft genome of *V. corymbosum* cv. Draper was annotated using the MAKER annotation pipeline [27]. Transcript and protein evidence used in the annotation included protein sequences downloaded from *A. thaliana* (Araport11) and UniprotKB plant databases, *V. corymbosum* expressed sequence tags (EST) from NCBI, and transciptome data assembled with StringTie [134] from different blueberry tissues (Table S4). A custom repeat library and Repbase [135] were used to mask repetitive regions in the genome using Repeatmasker [136]. *Ab initio* gene prediction was performed using gene predictors SNAP [137] and Augustus (Augustus: Gene Prediction, RRID:SCR_008417) [138]. The resulting MAKER Max gene set was filtered to select gene models with Pfam domain and annotation edit distance (AED) < 1.0. The filtered gene set (MAKER standard) was further scanned for transposase coding regions. The amino acid sequence of predicted genes was searched (BLASTP, 1e-10) against a

transposase database [27]. The alignment between the genes and the transposases was further filtered for those caused by the presence of sequences with low complexity. The total length of genes matching transposases was calculated based on the output from the search. If more than 30% of gene length aligned to the transposases, the gene is removed from the gene set. Furthermore, to assess the completeness of annotation, the *V. corymbosum* Maker standard gene set was searched against the Benchmarking Universal Single-Copy Orthologs (BUSCO v.3) [31] plant dataset (embryophyta_odb9). Genes were annotated with pfam domains using InterProScan (InterProScan, RRID:SCR_005829) v5.26-65.0 [139].

Annotation of repetitive elements

To identify and classify repetitive elements in the genome, long terminal repeat (LTR) retrotransposon candidates were searched using LTRharvest [140] and LTR_finder [141], and further identified and classified (e.g., Copia and Gypsy) using LTR_retriever [40]. A non-redundant LTR library was also produced by LTR_retriever. Miniature inverted transposable elements (MITEs) were identified using MITE-Hunter [142]. MITEs were manually checked for target site duplications and terminal inverted repeats and classified into superfamilies (e.g. *Mutator*, *hAT*, *Tc1Mariner/Stowaway* and *PIF/Harbinger*). Those with ambiguous TSD and TIR were classified as “unknowns.” Using the MITE and LTR libraries, the *V. corymbosum* genome was masked using Repeatmasker. The masked genome was further mined for repetitive elements using Repeatmodeler [143]. The repeats were then categorized into two groups: sequences with and without identities. Those without identities were searched against the transposase database and if they had a match, they were considered a transposon. The repeats were then filtered to exclude gene fragments using ProtExcluder [27] and summarized using the “fam_coverage.pl” script in the LTR_retriever package. The assembly continuity of repeat space was assessed using the LTR Assembly Index (LAI) [144] deployed in the LTR_retriever package [40]. LAI was calculated based on either 3 Mb sliding windows or the whole assembly using LAI = (Intact LTR-RT length * 100)/Total LTR-RT length. For the sliding window estimation, a step of 300 Kb was used (-step 300000 -window 3000000). To account for dynamics of LTR retrotransposons, LAI was adjusted by the mean identity of LTR sequences in the genome based on all-versus-all blastn search, which was also performed by the LAI program [144].

Transcriptome assembly and gene-expression analysis

Illumina adapters were removed from the raw reads using Trimmomatic/0.33 (Trimmomatic, RRID:SCR_011848) [145] and trimmed reads were filtered using FASTX Toolkit (http://hannonlab.cshl.edu/fastx_toolkit/index.html). After quality assessment using FastQC (FastQC, RRID:SCR_014583; http://www.bioinformatics.bbsrc.ac.uk/projects/fastqc), the filtered reads were then aligned to the *V. corymbosum* genome using STAR [146]. For the samples which were used for annotation, transcript assembly was performed *de novo* using StringTie. Counts of uniquely mapping reads were generated through HTSeq[147] for all 35 RNAseq datasets (plant tissue samples as well as fruit developmental series samples). Multimapping reads were excluded from the analysis except for the tandem gene expression analysis. Differential gene expression analysis was performed using the DESeq2 pipeline [148] across fruit developmental stages with three biological replicates per developmental stage (e.g. stage 1 compared to stage 2)(Figure 3). Gene expression values were derived by calculating the Fragments Per Kilobase per Million reads mapped (FPKM) values using the standard formula for FPKM (=read count/“per million” scaling factor)/gene length in kilobases (Kb)).

To construct the gene co-expression network, genes that were not expressed or very weakly expressed (count < 5) in 30 or more conditions were first excluded from the analysis. The count data was then transformed into variance stabilized values using the variance stabilizing transformation (VST) function in DEseq [147]. Pairwise correlations of gene expression was calculated using Pearson's correlation coefficient (PCC) and mutual rank (MR)[149,150] using scripts available for download from the project's data repository [155]. MR scores were transformed to network edge weights using geometric decay function $e^{-(MR-1/x)}$ [151]; five different co-expression networks were constructed with *x* set to 5, 10, 25, 50, and 100, respectively. Edges with PCC < 0.6 or edge weight < 0.01 was excluded. For each network, modules of co-expressed genes were detected using ClusterONE v1.0 using default parameters [152], and modules with P value > 0.1 or quality score < 0.2 were excluded. The results from all co-expression networks were then combined by collapsing modules into metamodules of nonoverlapping gene sets.

Oxygen Radical Absorbance Capacity (ORAC) Analysis

Total antioxidant capacity of tissues from the fruit developmental panel was analyzed using the oxygen radical absorbance capacity (ORAC) assay [69]. Briefly, ~20-30 mg of frozen ground fruit tissue was measured for tissue samples prior to extraction. Sample extractions were performed on ground tissue using 1.8 mL of ice cold 50% acetone. Samples were vortexed, and then put on a shaker for 5 minutes at room temperature. Samples were then centrifuged at 4 °C for 15 minutes (4,500 *g*). The ORAC assay was performed in a 96-well black microplate (Thermo Fisher Scientific, Waltham, MA) using the FLUOstar OPTIMA microplate reader (BMG LABTECH, Offenburg, Germany). Each reaction well contained 150 µl of 0.08 µM fluorescein and 25 µl of 75 mM phosphate buffer (blank), Trolox standards (6-Hydroxy-2,5,7,8-tetramethylchroman-2-carboxylic acid), or diluted sample extracts. For blueberry tissue samples 1:80-1:20 dilutions were used. Upon loading all appropriate wells, the 96-well microplate was put into the microplate reader and incubated for 10 minutes at 37 °C. Following incubation, 25 µl of 150 mM AAPH (2,2'-azobis-2-methyl-propanimidamide, dihydrochloride) was added to each well and fluorescence measurements began immediately. Fluorescence measurements (excitation: 485 nm, emission: 520 nm) were taken for 90 seconds per cycle for 70 cycles until the fluorescent probe signal was completely quenched. The area under the fluorescence decay curve (AUC) was calculated for each well. The total antioxidant capacity of a sample was calculated by subtracting the AUC from the blank curve from the AUC of the sample curve to obtain the net AUC. Using Trolox (water-soluble analog of Vitamin E) of a known concentration a standard curve was generated (12.5 µM – 100 µM) and the total antioxidant capacity of each sample was calculated as Trolox Equivalents (TE). Each sample was run twice for two technical replicates. The coefficient of variation between technical replicates was required to be less than 0.20. Biological replicates (n= 3) were run for all tissues in the fruit developmental series.

Assay of phenolics and anthocyanin content

Berries from 'Draper' were collected as described above. Approximately 100 mg (~10:1 solvent/tissue ratio) of each frozen ground sample was resuspended in extraction solvent in a 2 ml tube (80% Methanol/20% water + 0.1% formic acid, containing 0.5 M telmisartan (internal standard)). Ground tissue was immediately mixed thoroughly to prevent thawing during extraction and to prevent metabolism of analytes by enzymes in the samples. All tubes were spun down for 10 min at 13,000 x g to pellet protein and other insoluble material. Then, 1 ml of supernatant was transferred to an

autosampler vial. Anthocyanin content was evaluated by liquid chromatography-mass spectrometry (LC-MS) as follows: 5 ml of sample extract were separated using a 10 min gradient on a Waters Acquity HSS-T3 UPLC column (2.1 x 100 mm) on a Waters Acquity UPLC system interfaced with a Waters Xevo G2-XS quadrupole time-of-flight mass spectrometer (Waters Corp, Milford, MA). Column temperature was maintained at 40°C and the flow rate was 0.3 ml/min with starting conditions of 100% solvent A (water + 0.1% formic acid) and 0% solvent B (acetonitrile). The gradient was as follows: hold at 100% A for 0.5 min, ramp to 50% B at 6 min, then ramp to 99% B at 6.5 min, hold at 99% B to 8.5 min, return to 100% A at 8.51 min and hold at 100% A until 10 min. Mass spectra were acquired in positive ion mode electrospray ionization over m/z 50-1500 in continuum mode using a data-independent MS^E method which acquires data under both low and high collision energy conditions with the high collision energy setting using a ramp from 20-80 V. Capillary voltage was 3 kV, desolvation temperature was 350°C, source temperature was 100°C, cone gas flow was 25 L/hr and desolvation gas flow was 600 L/hr. Correction for mass drift was performed using continuous infusion of the lock mass compound leucine encephalin. Anthocyanins and other related flavonoids were identified based on accurate mass and fragmentation pattern. Peak areas were determined using Quanlynx within the Masslynx software package (Waters Corp). Relative anthocyanin content was calculated for each sample using the formula: reported peak area of the compound/peak area of internal standard/weight of extracted tissue (peak area/IS/gdw).

Genomic and Gene Family Analyses
The genome was aligned against itself in CoGe's SynMap program using LAST (LAST, RRID:SCR_006119) and default parameters [153]. Maximum distance between two matches was set to 20 genes, with minimum number of aligned pairs set to 10 genes. Tandemly duplicated genes were identified and filtered from CoGe outputs with a max distance of 10 genes. Fractionation bias was calculated, setting the max query and target chromosomes to 48. These analyses can be regenerated using the following link: https://genomevolution.org/r/12w9o. Protein sequences of blueberry was searched against previously characterized antioxidant related genes in Arabidopsis and other species in UniprotKB and NCBI databases using blastp in the BLAST+ package [154] with a cut-off e-value of 1E-10.

Availability of supporting data:
The genome assembly, annotations, and other supporting data are publicly available on PURR [155] and also via the *Gigascience* database GigaDB [156] and the CyVerse CoGe platform [157]. The raw sequence data were deposited in the Short Read Archive
under NCBI BioProject ID PRJNA494180.

Additional files:
Extended Data Table 1
Extended Data Table 2
Extended Data Table 3
Extended Data Table 4
Extended Data Table 5
Extended Data Table 6

Competing interests:
The authors declare that they have no competing interests.

Abbreviations:
AED: annotation edit distance; ANR: anthocyanidin reductase; ANS: anthocyanidin synthase; BLASTP: Basic Local Alignment Search Tool (Protein); bHLH: basic helix-loop-helix; bp: base pair; BUSCO: Benchmarking Universal Single-Copy Orthologs; CGA: chlorogenic acid; 4CL: 4-hydroxycinnamoyl CoA ligase; C3H: cytochrome P450 98A3; C4H: trans-cinnamate 4-monooxygenase; CHI: chalcone flavonone isomerase; CHS: chalcone synthase; DFR: dihydroflavonol reductase; EST: expressed sequence tags; F3H: flavanone 3-hydroxylase, F3’H: flavonoid 3′-hydroxylase; F3’5’H: flavonoid 3′,5′-hydroxylase; FHT: flavanone-3β-hydroxylase; FPKM: fragments per kilobase per million; kb: kilo base; HCT: hydroxycinnamoyl-CoA shikimate/quinate hydroxycinnamoyltransferase; HQT: hydroxycinnamoyl-CoA quinate hydroxycinnamoyltransferase; LAI: LTR Assembly Index; LAR: leucoanthocyanidin reductase; LC-MS: liquid chromatography-mass spectrometry; LTR: long terminal repeats; LTR-RT: long terminal repeat retrotransposons; Mb: mega base; MBW: MYB-bHLH-WD; MCRA: most common recent ancestor; MITE: miniature inverted repeat; MP: mate-pair; MR: mutual rank; MRP: multidrug resistance-associated protein; MYB: myeloblastosis; OMT: anthocyanin-O-methyltransferase; ORAC: oxygen radical absorbance capacity; PAL: phenylalanine ammonia-lyase; PCC: Pearson’s correlation coefficient; ROS: reactive oxygen species; RTSF: Michigan State University Research Technology Support Facility; STP: sugar transport protein; SUT: sucrose transporter; SWEET: Sugar Will Eventually be Exported Transporter; TE: transposable element; TST: tonoplast sugar transporter; UFGT: UDP-glucose flavonoid 3-O-glucosyl transferase; UPLC: Ultra Performance Liquid Chromatography; VST: variance stabilizing transformation; WDR: WD40 repeats.

Author contributions:
P.P.E. designed the research; M.C., C.P.L., C.M.W., S.O., K.A.B., J.W., J.H.W., A.E.Y., E.I.A., H.T., Z.X., P.C., G.B., A.B., K.B., T.S., L.S., G.S., K.L.C., A.S., N.V., C.R.B., R.V., N.J., and P.P.E. performed research and/or analyzed data; and M.C. and P.P.E. drafted the manuscript. All authors reviewed and edited the manuscript.

Acknowledgements:
We thank the reviewers and Editor for their helpful comments during the review of this manuscript. This work was supported by Michigan State University AgBioResearch, USDA-NIFA HATCH 1009804 to P.P.E., USDA-NIFA AFRI 1015241 to P.P.E and G.S., USDA-NIFA HATCH 1016057 to J.H.W., National Natural Science Foundation of China 31560302 to X.Z., and Inner Mongolia Major and Special Program of Science and Technology 5163901 to X.Z.

References:

1. Coville FV. Experiments in Blueberry Culture. U.S. Government Printing Office; 1910.

2. Ballington JR. Collection, utilization, and preservation of genetic resources in Vaccinium. HortScience. American Society for Horticultural Science; 2001;36:213–20.

3. Lewis NM, Ruud J. Blueberries in the American Diet. Nutr Today. 2005;40:92.

4. Faostat F. Statistical data. Food and Agriculture Organization of the United Nations, Rome. 2017;

5. Kron KA, Judd WS, Stevens PF, Crayn DM, Anderberg AA, Gadek PA, et al. Phylogenetic Classification of Ericaceae: Molecular and Morphological Evidence. Bot Rev. The New York Botanical Garden; 2002;68:335–423.

6. Schwery O, Onstein RE, Bouchenak-Khelladi Y, Xing Y, Carter RJ, Linder HP. As old as the mountains: the radiations of the Ericaceae. New Phytol. 2015;207:355–67.

7. Michalska A, Lysiak G. Bioactive Compounds of Blueberries: Post-Harvest Factors Influencing the Nutritional Value of Products. Int J Mol Sci. 2015;16:18642–63.

8. Davidson KT, Zhu Z, Balabanov D, Zhao L, Wakefield MR, Bai Q, et al. Beyond Conventional Medicine - a Look at Blueberry, a Cancer-Fighting Superfruit. Pathol Oncol Res [Internet]. 2017; Available from: http://dx.doi.org/10.1007/s12253-017-0376-2

9. Faostat F. FAOSTAT statistical database. 2016.

10. Vorsa N, Johnson-Cicalese J. American Cranberry. In: Badenes ML, Byrne DH, editors. Fruit Breeding. Boston, MA: Springer US; 2012. p. 191–223.

11. Darrow GM, Others. The strawberry. History, breeding and physiology. The strawberry History, breeding and physiology [Internet]. Holt, Rinehart & Winston, New York; 1966; Available from: https://www.cabdirect.org/cabdirect/abstract/19681601719

12. Prior RL, Cao G, Martin A, Sofic E, McEwen J, O'Brien C, et al. Antioxidant Capacity As Influenced by Total Phenolic and Anthocyanin Content, Maturity, and Variety of Vaccinium Species. J Agric Food Chem. American Chemical Society; 1998;46:2686–93.

13. Kim H, Bartley GE, Rimando AM, Yokoyama W. Hepatic gene expression related to lower plasma cholesterol in hamsters fed high-fat diets supplemented with blueberry peels and peel extract. J Agric Food Chem. 2010;58:3984–91.

14. Wang SY, Camp MJ, Ehlenfeldt MK. Antioxidant capacity and α-glucosidase inhibitory activity in peel and flesh of blueberry (Vaccinium spp.) cultivars. Food Chem. 2012;132:1759–68.

15. Faria A, Pestana D, Teixeira D, de Freitas V, Mateus N, Calhau C. Blueberry anthocyanins and pyruvic acid adducts: anticancer properties in breast cancer cell lines. Phytother Res. 2010;24:1862–9.

16. Hurst RD, Wells RW, Hurst SM, McGhie TK, Cooney JM, Jensen DJ. Blueberry fruit polyphenolics suppress oxidative stress-induced skeletal muscle cell damage in vitro. Mol Nutr Food Res. Wiley Online Library; 2010;54:353–63.

17. Krikorian R, Shidler MD, Nash TA, Kalt W, Vinqvist-Tymchuk MR, Shukitt-Hale B, et al. Blueberry supplementation improves memory in older adults. J Agric Food Chem. 2010;58:3996–4000.

18. Norberto S, Silva S, Meireles M, Faria A, Pintado M, Calhau C. Blueberry anthocyanins in health promotion: A metabolic overview. J Funct Foods. 2013;5:1518–28.

19. Wang Y, Cheng M, Zhang B, Nie F, Jiang H. Dietary supplementation of blueberry juice enhances hepatic expression of metallothionein and attenuates liver fibrosis in rats. PLoS One. 2013;8:e58659.

20. Stull A, Cash K, Champagne C, Gupta A, Boston R, Beyl R, et al. Blueberry Bioactives Improve Endothelial Function in Adults with Metabolic Syndrome. The FASEB Journal [Internet]. The Federation of American Societies for Experimental Biology; 2015 [cited 2018 Apr 20]; Available from: http://www.fasebj.org/content/29/1_Supplement/923.17.short

21. Bell L, Lamport DJ, Butler LT, Williams CM. A study of glycaemic effects following acute anthocyanin-rich blueberry supplementation in healthy young adults. Food Funct. 2017;8:3104–10.

22. Gallardo RK, Stafne ET, DeVetter LW, Zhang Q, Li C, Takeda F, et al. Blueberry Producers' Attitudes toward Harvest Mechanization for Fresh Market. Horttechnology. 2018;28:10–6.

23. Gupta V, Estrada AD, Blakley I, Reid R, Patel K, Meyer MD, et al. RNA-Seq analysis and annotation of a draft blueberry genome assembly identifies candidate genes involved in fruit ripening, biosynthesis of bioactive compounds, and stage-specific alternative splicing. Gigascience. 2015;4:5.

24. Schnable JC, Springer NM, Freeling M. Differentiation of the maize subgenomes by genome dominance and both ancient and ongoing gene loss. Proc Natl Acad Sci U S A. 2011;108:4069–74.

25. Bird KA, VanBuren R, Puzey JR, Edger PP. The causes and consequences of subgenome dominance in hybrids and recent polyploids. New Phytol [Internet]. 2018; Available from: http://dx.doi.org/10.1111/nph.15256

26. Bottani S, Zabet NR, Wendel JF, Veitia RA. Gene Expression Dominance in Allopolyploids: Hypotheses and Models. Trends Plant Sci [Internet]. 2018; Available from: http://dx.doi.org/10.1016/j.tplants.2018.01.002

27. Campbell MS, Law M, Holt C, Stein JC, Moghe GD, Hufnagel DE, et al. MAKER-P: a tool kit for the rapid creation, management, and quality control of plant genome annotations. Plant Physiol. 2014;164:513–24.

28. Arabidopsis Genome Initiative. Analysis of the genome sequence of the flowering plant Arabidopsis thaliana. Nature. 2000;408:796–815.

29. Berardini TZ, Reiser L, Li D, Mezheritsky Y, Muller R, Strait E, et al. The Arabidopsis information resource: Making and mining the "gold standard" annotated reference plant genome. Genesis. 2015;53:474–85.

30. Huang S, Ding J, Deng D, Tang W, Sun H, Liu D, et al. Draft genome of the kiwifruit Actinidia chinensis. Nat Commun. 2013;4:2640.

31. Simão FA, Waterhouse RM, Ioannidis P, Kriventseva EV, Zdobnov EM. BUSCO: assessing genome assembly and annotation completeness with single-copy orthologs. Bioinformatics. 2015;31:3210–2.

32. Götz S, García-Gómez JM, Terol J, Williams TD, Nagaraj SH, Nueda MJ, et al. High-throughput functional annotation and data mining with the Blast2GO suite. Nucleic Acids Res. 2008;36:3420–35.

33. Kanehisa M, Goto S. KEGG: kyoto encyclopedia of genes and genomes. Nucleic Acids Res. 2000;28:27–30.

34. Edger PP, VanBuren R, Colle M, Poorten TJ, Wai CM, Niederhuth CE, et al. Single-molecule sequencing and optical mapping yields an improved genome of woodland strawberry (Fragaria vesca) with chromosome-scale contiguity. Gigascience. 2018;7:1–7.

35. VanBuren R, Bryant D, Bushakra JM, Vining KJ, Edger PP, Rowley ER, et al. The genome of black raspberry (Rubus occidentalis). Plant J. 2016;87:535–47.

36. Canaguier A, Grimplet J, Di Gaspero G, Scalabrin S, Duchêne E, Choisne N, et al. A new version of the grapevine reference genome assembly (12X.v2) and of its annotation (VCost.v3). Genom Data. 2017;14:56–62.

37. Law M, Childs KL, Campbell MS, Stein JC, Olson AJ, Holt C, et al. Automated update, revision, and quality control of the maize genome annotations using MAKER-P improves the B73 RefGen_v3 gene models and identifies new genes. Plant Physiol. Am Soc Plant Biol; 2015;167:25–39.

38. Lee S-I, Kim N-S. Transposable elements and genome size variations in plants. Genomics Inform. 2014;12:87–97.

39. Vicient CM, Casacuberta JM. Impact of transposable elements on polyploid plant genomes. Ann Bot. 2017;120:195–207.

40. Ou S, Jiang N. LTR_retriever: A Highly Accurate and Sensitive Program for Identification of Long Terminal Repeat Retrotransposons. Plant Physiol. American Society of Plant Biologists; 2018;176:1410–22.

41. Coville FV. BLUEBERRY CHROMOSOMES. Science. 1927;66:565–6.

42. Draper AD, Scott DH. Inheritance of albino seedling in tetraploid highbush blueberry. J Am Soc Hortic Sci [Internet]. 1971; Available from: http://agris.fao.org/agris-search/search.do?recordID=US201302242589

43. Jelenkovic G, Hough LF. CHROMOSOME ASSOCIATIONS IN THE FIRST MEIOTIC DIVISION IN THREE TETRAPLOID CLONES OF VACCINIUM CORYMBOSUM L. Can J Genet Cytol. NRC Research Press; 1970;12:316–24.

44. Xiong Z, Gaeta RT, Pires JC. Homoeologous shuffling and chromosome compensation maintain genome balance in resynthesized allopolyploid Brassica napus. Proc Natl Acad Sci U S A. 2011;108:7908–13.

45. Chester M, Gallagher JP, Symonds VV, Cruz da Silva AV, Mavrodiev EV, Leitch AR, et al. Extensive chromosomal variation in a recently formed natural allopolyploid species, Tragopogon miscellus (Asteraceae). Proc Natl Acad Sci U S A. 2012;109:1176–81.

46. Maere S, De Bodt S, Raes J, Casneuf T, Van Montagu M, Kuiper M, et al. Modeling gene and genome duplications in eukaryotes. Proc Natl Acad Sci U S A. 2005;102:5454–9.

47. Kagale S, Robinson SJ, Nixon J, Xiao R, Huebert T, Condie J, et al. Polyploid evolution of the Brassicaceae during the Cenozoic era. Plant Cell. 2014;26:2777–91.

48. Barker MS, Vogel H, Schranz ME. Paleopolyploidy in the Brassicales: analyses of the Cleome transcriptome elucidate the history of genome duplications in Arabidopsis and other Brassicales. Genome Biol Evol. 2009;1:391–9.

49. Doyle JJ, Egan AN. Dating the origins of polyploidy events. New Phytol. 2010;186:73–85.

50. Ziolkowski PA, Koczyk G, Galganski L, Sadowski J. Genome sequence comparison of Col and Ler lines reveals the dynamic nature of Arabidopsis chromosomes. Nucleic Acids Res. 2009;37:3189–201.

51. Ozkan H, Levy AA, Feldman M. Rapid differentiation of homeologous chromosomes in newly-formed allopolyploid wheat. Isr J Plant Sci. Taylor & Francis; 2002;50:65–76.

52. VanBuren R, Wai CM, Ou S, Pardo J, Bryant D, Jiang N, et al. Extreme haplotype variation in the desiccation-tolerant clubmoss Selaginella lepidophylla. Nat Commun. 2018;9:13.

53. Ma J, Bennetzen JL. Rapid recent growth and divergence of rice nuclear genomes. Proc Natl Acad Sci U S A. 2004;101:12404–10.

54. Lancaster LT. Molecular evolutionary rates predict both extinction and speciation in temperate angiosperm lineages. BMC Evol Biol. 2010;10:162.

55. Thomas B. Light signals and flowering. J Exp Bot. 2006;57:3387–93.

56. Grover CE, Gallagher JP, Szadkowski EP, Yoo MJ, Flagel LE, Wendel JF. Homoeolog expression bias and expression level dominance in allopolyploids. New Phytol. 2012;196:966–71.

57. Woodhouse MR, Cheng F, Pires JC, Lisch D, Freeling M, Wang X. Origin, inheritance, and gene regulatory consequences of genome dominance in polyploids. Proceedings of the National Academy of Sciences. National Acad Sciences; 2014;111:5283–8.

58. Edger PP, McKain MR, Bird KA, VanBuren R. Subgenome assignment in allopolyploids: challenges and future directions. Curr Opin Plant Biol. 2018/4;42:76–80.

59. Freeling M, Woodhouse MR, Subramaniam S, Turco G, Lisch D, Schnable JC. Fractionation mutagenesis and similar consequences of mechanisms removing dispensable or less-expressed DNA in plants. Curr Opin Plant Biol. 2012;15:131–9.

60. Cheng F, Wu J, Cai X, Liang J, Freeling M, Wang X. Gene retention, fractionation and subgenome differences in polyploid plants. Nature Plants. 2018;4:258–68.

61. Garsmeur O, Schnable JC, Almeida A, Jourda C, D’Hont A, Freeling M. Two evolutionarily distinct classes of paleopolyploidy. Mol Biol Evol. Oxford University Press; 2013;31:448–54.

62. Zhao M, Zhang B, Lisch D, Ma J. Patterns and Consequences of Subgenome Differentiation Provide Insights into the Nature of Paleopolyploidy in Plants. Plant Cell. 2017;29:2974–94.

63. Adams KL, Cronn R, Percifield R, Wendel JF. Genes duplicated by polyploidy show unequal contributions to the transcriptome and organ-specific reciprocal silencing. Proc Natl Acad Sci U S A. 2003;100:4649–54.

64. Boatwright JL, McIntyre LM, Morse AM, Chen S, Yoo M-J, Koh J, et al. A Robust Methodology for Assessing Differential Homeolog Contributions to the Transcriptomes of Allopolyploids. Genetics. 2018;210:883–94.

65. Bohner J, Bangerth F. Cell number, cell size and hormone levels in semi-isogenic mutants of Lycopersicon pimpinellifolium differing in fruit size. Physiol Plant. Wiley Online Library; 1988;72:316–20.

66. Gillaspy G, Ben-David H, Gruissem W. Fruits: A Developmental Perspective. Plant Cell. 1993;5:1439–51.

67. Zifkin M, Jin A, Ozga JA, Zaharia LI, Schernthaner JP, Gesell A, et al. Gene expression and

metabolite profiling of developing highbush blueberry fruit indicates transcriptional regulation of flavonoid metabolism and activation of abscisic acid metabolism. Plant Physiol. 2012;158:200–24.

68. Mainland CM, Tucker JW. BLUEBERRY HEALTH INFORMATION - SOME NEW MOSTLY REVIEW. Acta Hortic. 2002;39–43.

69. Gillespie KM, Chae JM, Ainsworth EA. Rapid measurement of total antioxidant capacity in plants. Nat Protoc. 2007;2:867–70.

70. Connor AM, Luby JJ, Tong CBS, Finn CE, Hancock JF. Genotypic and Environmental Variation in Antioxidant Activity, Total Phenolic Content, and Anthocyanin Content among Blueberry Cultivars. J Am Soc Hortic Sci. 2002;127:89–97.

71. Wang H, Guo X, Hu X, Li T, Fu X, Liu RH. Comparison of phytochemical profiles, antioxidant and cellular antioxidant activities of different varieties of blueberry (Vaccinium spp.). Food Chem. 2017;217:773–81.

72. Wu Y, Zhou Q, Chen X-Y, Li X, Wang Y, Zhang J-L. Comparison and screening of bioactive phenolic compounds in different blueberry cultivars: Evaluation of anti-oxidation and α-glucosidase inhibition effect. Food Res Int. 2017;100:312–24.

73. Kalt W, Ryan DAJ, Duy JC, Prior RL, Ehlenfeldt MK, Vander Kloet SP. Interspecific variation in anthocyanins, phenolics, and antioxidant capacity among genotypes of highbush and lowbush blueberries (Vaccinium section cyanococcus spp.). J Agric Food Chem. ACS Publications; 2001;49:4761–7.

74. Moyer RA, Hummer KE, Finn CE, Frei B, Wrolstad RE. Anthocyanins, phenolics, and antioxidant capacity in diverse small fruits: vaccinium, rubus, and ribes. J Agric Food Chem. 2002;50:519–25.

75. Castrejón ADR, Eichholz I, Rohn S, Kroh LW, Huyskens-Keil S. Phenolic profile and antioxidant activity of highbush blueberry (Vaccinium corymbosum L.) during fruit maturation and ripening. Food Chem. 2008;109:564–72.

76. Wang SY, Lin HS. Antioxidant activity in fruits and leaves of blackberry, raspberry, and strawberry varies with cultivar and developmental stage. J Agric Food Chem. 2000;48:140–6.

77. Zheng W, Wang SY. Oxygen radical absorbing capacity of phenolics in blueberries, cranberries, chokeberries, and lingonberries. J Agric Food Chem. 2003;51:502–9.

78. Clifford MN. Chlorogenic Acids. In: Clarke RJ, Macrae R, editors. Coffee: Volume 1: Chemistry. Dordrecht: Springer Netherlands; 1985. p. 153–202.

79. Rice-Evans CA, Miller NJ, Paganga G. Structure-antioxidant activity relationships of flavonoids and phenolic acids. Free Radic Biol Med. 1996;20:933–56.

80. Shi H, Shi A, Dong L, Lu X, Wang Y, Zhao J, et al. Chlorogenic acid protects against liver fibrosis in vivo and in vitro through inhibition of oxidative stress. Clin Nutr. 2016;35:1366–73.

81. Hollman PC. Evidence for health benefits of plant phenols: local or systemic effects? J Sci Food Agric. Wiley Online Library; 2001;81:842–52.

82. Olthof MR, Hollman PC, Zock PL, Katan MB. Consumption of high doses of chlorogenic acid, present in coffee, or of black tea increases plasma total homocysteine concentrations in humans--. Am J

Clin Nutr. Oxford University Press; 2001;73:532–8.

83. Charurin P, Ames JM, del Castillo MD. Antioxidant activity of coffee model systems. J Agric Food Chem. 2002;50:3751–6.

84. Yen W-J, Wang B-S, Chang L-W, Duh P-D. Antioxidant properties of roasted coffee residues. J Agric Food Chem. 2005;53:2658–63.

85. Watanabe T, Arai Y, Mitsui Y, Kusaura T, Okawa W, Kajihara Y, et al. The blood pressure-lowering effect and safety of chlorogenic acid from green coffee bean extract in essential hypertension. Clin Exp Hypertens. 2006;28:439–49.

86. Falcone Ferreyra ML, Rius SP, Casati P. Flavonoids: biosynthesis, biological functions, and biotechnological applications. Front Plant Sci. 2012;3:222.

87. Zhang Y. Regulation of Ascorbate Synthesis in Plants. In: Zhang Y, editor. Ascorbic Acid in Plants: Biosynthesis, Regulation and Enhancement. New York, NY: Springer New York; 2013. p. 87–99.

88. Laing W, Norling C, Brewster D, Wright M, Bulley S. Ascorbate Concentration In Arabidopsis thaliana And Expression Of Ascorbate Related Genes Using RNAseq In Response To Light And The Diurnal Cycle [Internet]. bioRxiv. 2017 [cited 2018 Apr 21]. p. 138008. Available from: https://www.biorxiv.org/content/early/2017/05/15/138008.abstract

89. Liu J, Osbourn A, Ma P. MYB Transcription Factors as Regulators of Phenylpropanoid Metabolism in Plants. Mol Plant. 2015;8:689–708.

90. Petroni K, Tonelli C. Recent advances on the regulation of anthocyanin synthesis in reproductive organs. Plant Sci. 2011;181:219–29.

91. Albert NW, Davies KM, Lewis DH, Zhang H, Montefiori M, Brendolise C, et al. A conserved network of transcriptional activators and repressors regulates anthocyanin pigmentation in eudicots. Plant Cell. 2014;26:962–80.

92. Huang W, Khaldun ABM, Chen J, Zhang C, Lv H, Yuan L, et al. A R2R3-MYB Transcription Factor Regulates the Flavonol Biosynthetic Pathway in a Traditional Chinese Medicinal Plant, Epimedium sagittatum. Front Plant Sci. 2016;7:1089.

93. Nguyen NH, Lee H. MYB-related transcription factors function as regulators of the circadian clock and anthocyanin biosynthesis in Arabidopsis. Plant Signal Behav. 2016;11:e1139278.

94. Jin J, Tian F, Yang D-C, Meng Y-Q, Kong L, Luo J, et al. PlantTFDB 4.0: toward a central hub for transcription factors and regulatory interactions in plants. Nucleic Acids Res. 2017;45:D1040–5.

95. Kautsar SA, Suarez Duran HG, Blin K, Osbourn A, Medema MH. plantiSMASH: automated identification, annotation and expression analysis of plant biosynthetic gene clusters. Nucleic Acids Res. 2017;45:W55–63.

96. Xi W, Zheng H, Zhang Q, Li W. Profiling Taste and Aroma Compound Metabolism during Apricot Fruit Development and Ripening. Int J Mol Sci [Internet]. 2016;17. Available from: http://dx.doi.org/10.3390/ijms17070998

97. Du X, Rouseff R. Aroma Active Volatiles in Four Southern Highbush Blueberry Cultivars Determined by Gas Chromatography–Olfactometry (GC-O) and Gas Chromatography–Mass

Spectrometry (GC-MS). J Agric Food Chem. American Chemical Society; 2014;62:4537–43.

98. Farneti B, Khomenko I, Grisenti M, Ajelli M, Betta E, Algarra AA, et al. Exploring Blueberry Aroma Complexity by Chromatographic and Direct-Injection Spectrometric Techniques. Front Plant Sci. 2017;8:617.

99. Beaulieu JC, Stein-Chisholm RE, Boykin DL. Qualitative Analysis of Volatiles in Rabbiteye Blueberry Cultivars at Various Maturities Using Rapid Solid-phase Microextraction. J Am Soc Hortic Sci. 2014;139:167–77.

100. Du X, Whitaker V, Rouseff R. Changes in strawberry volatile sulfur compounds due to genotype, fruit maturity and sample preparation. Flavour Fragr J. Wiley Online Library; 2012;27:398–404.

101. Du X, Plotto A, Song M, Olmstead J, Rouseff R. Volatile composition of four southern highbush blueberry cultivars and effect of growing location and harvest date. J Agric Food Chem. ACS Publications; 2011;59:8347–57.

102. Hirvi T, Honkanen E. The aroma of blueberries. J Sci Food Agric. 1983;34:992–6.

103. Horvat RJ, Senter SD. Comparison of the volatile constituents from rabbiteye blueberries (Vaccinium ashei) during ripening. J Food Sci. Wiley Online Library; 1985;50:429–31.

104. Gilbert JL, Olmstead JW, Colquhoun TA, Levin LA, Clark DG, Moskowitz HR. Consumer-assisted Selection of Blueberry Fruit Quality Traits. HortScience. 2014;49:864–73.

105. Eom J-S, Chen L-Q, Sosso D, Julius BT, Lin IW, Qu X-Q, et al. SWEETs, transporters for intracellular and intercellular sugar translocation. Curr Opin Plant Biol. 2015;25:53–62.

106. Ren Y, Guo S, Zhang J, He H, Sun H, Tian S, et al. A Tonoplast Sugar Transporter Underlies a Sugar Accumulation QTL in Watermelon. Plant Physiol. 2018;176:836–50.

107. Lin IW, Sosso D, Chen L-Q, Gase K, Kim S-G, Kessler D, et al. Nectar secretion requires sucrose phosphate synthases and the sugar transporter SWEET9. Nature. 2014;508:546–9.

108. Chen H-Y, Huh J-H, Yu Y-C, Ho L-H, Chen L-Q, Tholl D, et al. The Arabidopsis vacuolar sugar transporter SWEET2 limits carbon sequestration from roots and restricts Pythium infection. Plant J. 2015;83:1046–58.

109. Wormit A, Trentmann O, Feifer I, Lohr C, Tjaden J, Meyer S, et al. Molecular identification and physiological characterization of a novel monosaccharide transporter from Arabidopsis involved in vacuolar sugar transport. Plant Cell. 2006;18:3476–90.

110. Sturm A, Tang GQ. The sucrose-cleaving enzymes of plants are crucial for development, growth and carbon partitioning. Trends Plant Sci. Elsevier; 1999;4:401–7.

111. Qin G, Zhu Z, Wang W, Cai J, Chen Y, Li L, et al. A Tomato Vacuolar Invertase Inhibitor Mediates Sucrose Metabolism and Influences Fruit Ripening. Plant Physiol. 2016;172:1596–611.

112. Achaz G, Coissac E, Viari A, Netter P. Analysis of intrachromosomal duplications in yeast Saccharomyces cerevisiae: a possible model for their origin. Mol Biol Evol. 2000;17:1268–75.

113. Leister D. Tandem and segmental gene duplication and recombination in the evolution of plant disease resistance genes. Trends Genet. 2004;20:116–22.

114. Chae L, Kim T, Nilo-Poyanco R, Rhee SY. Genomic signatures of specialized metabolism in plants. Science. 2014;344:510–3.

115. Edger PP, Pires JC. Gene and genome duplications: the impact of dosage-sensitivity on the fate of nuclear genes. Chromosome Res. 2009;17:699–717.

116. Freeling M. Bias in plant gene content following different sorts of duplication: tandem, whole-genome, segmental, or by transposition. Annu Rev Plant Biol. 2009;60:433–53.

117. Kliebenstein DJ, Lambrix VM, Reichelt M, Gershenzon J, Mitchell-Olds T. Gene duplication in the diversification of secondary metabolism: tandem 2-oxoglutarate-dependent dioxygenases control glucosinolate biosynthesis in Arabidopsis. Plant Cell. 2001;13:681–93.

118. Ober D. Seeing double: gene duplication and diversification in plant secondary metabolism. Trends Plant Sci. 2005;10:444–9.

119. Hofberger JA, Lyons E, Edger PP, Pires JC, Schranz ME. Whole Genome and Tandem Duplicate Retention Facilitated Glucosinolate Pathway Diversification in the Mustard Family. Genome Biol Evol. 2013;5:2155–73.

120. Conant GC, Wolfe KH. Turning a hobby into a job: how duplicated genes find new functions. Nat Rev Genet. 2008;9:938–50.

121. Bekaert M, Edger PP, Pires JC, Conant GC. Two-phase resolution of polyploidy in the Arabidopsis metabolic network gives rise to relative and absolute dosage constraints. Plant Cell. 2011;23:1719–28.

122. Rizzon C, Ponger L, Gaut BS. Striking similarities in the genomic distribution of tandemly arrayed genes in Arabidopsis and rice. PLoS Comput Biol. 2006;2:e115.

123. Kliebenstein DJ. A role for gene duplication and natural variation of gene expression in the evolution of metabolism. PLoS One. 2008;3:e1838.

124. Eckardt NA. Genome Dominance and Interaction at the Gene Expression Level in Allohexaploid Wheat. Plant Cell. 2014;26:1834.

125. Li A, Liu D, Wu J, Zhao X, Hao M, Geng S, et al. mRNA and Small RNA Transcriptomes Reveal Insights into Dynamic Homoeolog Regulation of Allopolyploid Heterosis in Nascent Hexaploid Wheat. Plant Cell. 2014;26:1878–900.

126. Pfeifer M, Kugler KG, Sandve SR, Zhan B, Rudi H, Hvidsten TR, et al. Genome interplay in the grain transcriptome of hexaploid bread wheat. Science. 2014;345:1250091.

127. International Wheat Genome Sequencing Consortium (IWGSC), IWGSC RefSeq principal investigators:, Appels R, Eversole K, Feuillet C, Keller B, et al. Shifting the limits in wheat research and breeding using a fully annotated reference genome. Science [Internet]. 2018;361. Available from: http://dx.doi.org/10.1126/science.aar7191

128. Zhang H-B, Zhao X, Ding X, Paterson AH, Wing RA. Preparation of megabase-size DNA from plant nuclei. Plant J. 1995;7:175–84.

129. VanBuren R, Bryant D, Edger PP, Tang H, Burgess D, Challabathula D, et al. Single-molecule sequencing of the desiccation-tolerant grass Oropetium thomaeum. Nature. 2015;527:508–U209.

130. Avni R, Nave M, Barad O, Baruch K, Twardziok SO, Gundlach H, et al. Wild emmer genome architecture and diversity elucidate wheat evolution and domestication. Science. 2017;357:93–7.

131. Luo M-C, Gu YQ, Puiu D, Wang H, Twardziok SO, Deal KR, et al. Genome sequence of the progenitor of the wheat D genome Aegilops tauschii. Nature. 2017;551:498–502.

132. Lieberman-Aiden E, van Berkum NL, Williams L, Imakaev M, Ragoczy T, Telling A, et al. Comprehensive mapping of long-range interactions reveals folding principles of the human genome. Science. 2009;326:289–93.

133. Putnam NH, O'Connell BL, Stites JC, Rice BJ, Blanchette M, Calef R, et al. Chromosome-scale shotgun assembly using an in vitro method for long-range linkage. Genome Res [Internet]. Cold Spring Harbor Laboratory Press; 2016 [cited 2018 Apr 21]; Available from: https://genome.cshlp.org/content/early/2016/02/08/gr.193474.115.long

134. Pertea M, Pertea GM, Antonescu CM, Chang T-C, Mendell JT, Salzberg SL. StringTie enables improved reconstruction of a transcriptome from RNA-seq reads. Nat Biotechnol. 2015;33:290–5.

135. Jurka J, Kapitonov VV, Pavlicek A, Klonowski P, Kohany O, Walichiewicz J. Repbase Update, a database of eukaryotic repetitive elements. Cytogenet Genome Res. 2005;110:462–7.

136. Smit AFA, Hubley R, Green P. RepeatMasker. 1996.

137. Korf I. Gene finding in novel genomes. BMC Bioinformatics. 2004;5:59.

138. Stanke M, Waack S. Gene prediction with a hidden Markov model and a new intron submodel. Bioinformatics. 2003;19 Suppl 2:ii215–25.

139. Jones P, Binns D, Chang H-Y, Fraser M, Li W, McAnulla C, et al. InterProScan 5: genome-scale protein function classification. Bioinformatics. 2014;30:1236–40.

140. Ellinghaus D, Kurtz S, Willhoeft U. LTRharvest, an efficient and flexible software for de novo detection of LTR retrotransposons. BMC Bioinformatics. 2008;9:18.

141. Xu Z, Wang H. LTR_FINDER: an efficient tool for the prediction of full-length LTR retrotransposons. Nucleic Acids Res. 2007;35:W265–8.

142. Han Y, Wessler SR. MITE-Hunter: a program for discovering miniature inverted-repeat transposable elements from genomic sequences. Nucleic Acids Res. 2010;38:e199.

143. Smit A, Hubley R. RepeatModeler Open-1.0. Available fom http://www repeatmasker org. 2008;

144. Ou S. LTR_retriever [Internet]. Github; [cited 2018 Aug 3]. Available from: https://github.com/oushujun/LTR_retriever

145. Bolger AM, Lohse M, Usadel B. Trimmomatic: a flexible trimmer for Illumina sequence data. Bioinformatics. 2014;30:2114–20.

146. Dobin A, Gingeras TR. Mapping RNA-seq Reads with STAR. Curr Protoc Bioinformatics. 2015;51:11.14.1–19.

147. Anders S, Pyl PT, Huber W. HTSeq--a Python framework to work with high-throughput sequencing data. Bioinformatics. 2015;31:166–9.

148. Love MI, Huber W, Anders S. Moderated estimation of fold change and dispersion for RNA-seq data with DESeq2. Genome Biol. 2014;15:550.

149. Obayashi T, Kinoshita K. Rank of correlation coefficient as a comparable measure for biological significance of gene coexpression. DNA Res. 2009;16:249–60.

150. Obayashi T, Aoki Y, Tadaka S, Kagaya Y, Kinoshita K. ATTED-II in 2018: A Plant Coexpression Database Based on Investigation of the Statistical Property of the Mutual Rank Index. Plant Cell Physiol. 2018;59:440.

151. Wisecaver JH, Borowsky AT, Tzin V, Jander G, Kliebenstein DJ, Rokas A. A Global Co-expression Network Approach for Connecting Genes to Specialized Metabolic Pathways in Plants. Plant Cell [Internet]. American Society of Plant Biologists; 2017; Available from: http://www.plantcell.org/content/early/2017/04/13/tpc.17.00009

152. Nepusz T, Yu H, Paccanaro A. Detecting overlapping protein complexes in protein-protein interaction networks. Nat Methods. 2012;9:471–2.

153. Lyons E, Pedersen B, Kane J, Freeling M. The Value of Nonmodel Genomes and an Example Using SynMap Within CoGe to Dissect the Hexaploidy that Predates the Rosids. Trop Plant Biol. Springer-Verlag; 2008;1:181–90.

154. Camacho C, Coulouris G, Avagyan V, Ma N, Papadopoulos J, Bealer K, et al. BLAST+: architecture and applications. BMC Bioinformatics. 2009;10:421.

155. The Purdue University Research Repository (PURR). https://purr.purdue.edu/projects/blueberrygenome.

156. Colle M, Leisner C, Wai CM, Ou S, Bird K, Wang J, et al. Supporting data for "Haplotype-phased genome and evolution of phytonutrient pathways of tetraploid blueberry" GigaScience Database 2019. http://dx.doi.org/10.5524/100537

157. CoGe – a platform for comparative genomics. https://genomevolution.org/coge/GenomeInfo.pl?gid=36464

Personal Cover
Click here to access/download;Manuscript;CoverLetter_BB_December.pdf
Click here to view linked References

December 18th, 2018

Patrick P. Edger, Ph.D.
Department of Horticulture
Michigan State University
East Lansing, MI 48824
edgerpat@msu.edu

Dear Dr. Hans Zauner,

First and foremost, I want thank you on behalf of all the coauthors for your time and effort in handling our manuscript. We have revised the manuscript per your instructions and have included a point-by-point response to the reviewers comments. I completely agree that Reviewer #2 comments were incredibly helpful in improving the quality of our manuscript. We would like to make this genomic resource available to the community as soon as possible. Thus, we have decided to not include an analysis of pseudogenes as recommended by Reviewer #2. We also feel that there is sufficient novel findings in the manuscript to stand alone. Please see the point-by-point response document for additional details. Furthermore, I'm very supportive of having the data also hosted on GigaDB. We've been working with Dr. Mary Ann Tuli to setup a GigaDB database as well as already making all of the raw data publicly available on NCBI-SRA.

Please let me know if there are any additional questions or concerns.

Sincerely,

Patrick P. Edger

Patrick P. Edger

Response to Reviewers

Click here to access/download;Manuscript;Response to Reviewers BB December 2018.pdf

Click here to view linked References

Dear Reviewers and Editors:

Thank you for a thorough and very thoughtful review of our manuscript. We have made several changes to our manuscript to address points raised by Reviewer #2, which truly helped improve the overall quality of the manuscript. This includes slightly changing the title of the manuscript based on comment #4 to “Haplotype-phased genome and evolution of phytonutrient pathways of tetraploid blueberry”. We feel that this new title more accurately reflects the highlights of the paper. Please see below responses in blue.

Best,

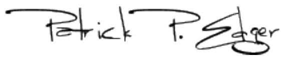

Patrick Edger
Michigan State University

Reviewer reports:

Reviewer #1: This is a well-prepared manuscript. The results provide an important platform for further research on this and related species. The specific gene groups studied are appropriate to the unique features of this system. The observations on sub genome contributions are also important contributions to understanding of polyploid systems.

Response: Thank you for the positive feedback on our manuscript.

Reviewer #2: The manuscript presents a chromosome-scale haplotype phased genome assembly of highbush blueberry which is of high economic importance mainly due to its composition of health promoting phytonutrients. Genes and pathways associated with antioxidant and sugar levels in blueberry fruits were analyzed in more detail. Overall this work provides a valuable new genomic reference for blueberry and enables future studies on the genome of blueberry for research and breeding purposes. Furthermore, the findings give insights into expression patterns of genes associated with fruit ripening and antioxidant biosynthesis as well as the expansion of gene families related to these traits. Finally, it is shown that blueberry is an allopolyploid species with subgenome dominance. The presented reference genome sequence is of high quality and both the gene annotation and TE analysis are sound. Transcriptome analyses were properly carried out, although many of the results are mainly confirming prior assumptions. The manuscript could further be improved by considering these points:

Major:
1. the authors claim that there is a high average sequence similarity among syntenic homeologous genes (96.3%) and that there is a divergence between syntenic homeologous genes of ~0.036 per synonymous site. They thus conclude that blueberry is allopolyploid. It is not obvious how this conclusion was made and it should be further elaborated on the connection between allopolyploidy and the mentioned numbers.

Response: We thank you for this comment and have revised the manuscript to help clarify this to the reader. We completely agree that additional details were needed. Furthermore, we added a time estimate for the polyploid event based on dating unique LTR insertions.

2. in the case of genes involved in anthocyanin and chlorogenic acid biosynthesis, various tandem duplicates were identified. Are these all functional genes and not pseudogenes? Also, a more detailed description or analysis of the expression patterns of tandem duplicated genes and the mentioned gene family expansions would be desirable. This would shed light on possible dosage effects and put the analysis into a biological context with the other transcriptome analyses.

Response: We have added additional details regarding the expression of tandem duplicated genes to the manuscript in the 'Expansion of antioxidant-related gene families through tandem duplication' section and have revised the 'Transcriptome assembly and gene-expression analysis' section in the methods. In short, 83.4% the tandem duplicates were expressed in at least one transcriptome library with 73.5% expressed in at least one of the fruit transcriptome libraries. We agree that additional transcriptome analyses with more diverse libraries are needed to provide further insights into the dosage effects of these duplicate genes and their possible involvement in fruit development and quality traits. We are currently working on generating additional datasets and analyses which will be included in a follow-up manuscript later next year. The goal for this manuscript was to provide a new genomic resource and research findings to the blueberry community to enable various future research efforts including to investigate the evolution of duplicate genes.

3. in that context, and especially for an allopolyploid, a more detailed analysis of pseudogenes and gene fragments would be very interesting.

Response: We recognize the importance of differentiating pseudogenes from "real" genes as some pseudogenes are linked to certain biological functions. There are available tools to predict pseudogenes, however, they have limitations and characterizing a pseudogene remains difficult given its high similarity to the "real" genes. Also, to verify if a pseudogene is functional or not, we need to set up experiments to examine the presence or absence of biological function of a pseudogene. At this point, we believe that performing such extensive experiments is beyond the scope of this paper. However, we agree with you that this would likely yield very interesting results.

4. the authors found a difference in gene expression levels between the two subgenomes and hypothesize this might be due to differences in transposon density around homeologous genes. Since transposon density was also measured, it should be included in the manuscript whether or not TE-density correlates with subgenome specific gene expression levels.

Response: Transposable element (TE) content differences among homoeologous genes may not play major role in highbush blueberry. Each of the homoeologous chromosomes have relatively similar total TE content. For example, there is only a ~1.85% differences in TE content between chromosomes 1, 13, 25 and 37. Chromosomes 1 and 13 having the most and least amount of TEs, respectively. The most dominantly expressed subgenome typically has the lowest amount of methylated TEs near genes. We are currently in the process of generating the datasets to look at the methylation status of individual TEs in

different organs and developmental stages and to compare this to gene expression patterns. These analyses will take us several months (up to a year) to complete and thus will be included as part of follow-up manuscript. The observed expression patterns shown in Figure 2B are very interesting. These are very different from what has been observed in most other allopolyploids exhibiting subgenome expression dominance - a single dominant subgenome. However, as you point out below, these findings are quite preliminary (i.e. "hints"). For these reasons, we have decided to change the title of the manuscript and have revised the manuscript to state that these findings are preliminary and require follow-up studies.

5. The mapping of reads retrieved from RNA-Seq data to the genome was performed uniquely. Since highly similar genomic regions are in general problematic when performing gene expression studies, it should be included in the description of the method whether non-unique reads where mapped randomly or excluded from the mapping.

Response: We excluded the non-unique reads from the analyses. We revised the 'Transcriptome assembly and gene-expression analysis' section in the methods to include this additional information.

6. gene expression was analyzed across 14 different samples and total gene expression values were used to compare total gene expression across haplotypes. However, replicates were only available for fruit samples but not for all other samples. Hence, the findings here should be described as hints. Moreover, it should be explained how the fruit samples were treated in this analysis (was only one replicate used or the average count of all three?).

Response: Total gene expression values for subgenome dominance analysis were derived from a single biological replicate of different tissue types (1=flower bud; 2=flower at anthesis; 3=petal fall; 4=green fruit; 5=pink fruit; 6=ripe fruit; 7, 8=leaf collected at 12 p.m. and 12 a.m., respectively; 9, 10, 11=methyl jasmonate treated leaf collected after one hour, eight hours and 24 hours, respectively; 12=shoot; 13=root; 14=salt-treated root). To eliminate potential variability in gene expression due to environmental effects, we only analyzed the data from tissues collected from 'Draper' grown in the growth chamber (i.e. identical environmental conditions). We completely agree that these findings are quite preliminary and require follow-up analyses. Thus, we have added additional text to the manuscript to address this important point and removed several paragraphs in the Discussion section.

7. The citations of extended table 4 and 5 in the text seem to be wrong! I believe 5 is meant to be 4 and 6 (which doesn't exist) should be 5. This needs to be fixed. In general there seems to be a problem with the formatting ("error for extended table 2") and content of the extended tables. I would suggest to deposit them under a public data DOI instead of having them attached to the main manuscript.

Response: We apologize for this mix-up. We have corrected Extended data tables 4, 5, and 6. We checked Extended data table 2 and did not discover any formatting issues. The file size is a bit large, thus, it may take a few moments for some computers to load all of its contents.

8. I recommend that the manuscript is proof read in order to improve language, sentence structure, grammar and typing errors.

Response: We have carefully edited the manuscript to correct these various errors.

Minor:

1. I would recommend not to use the term "expressed chromosomes".

Response: We agree and have removed this term from the manuscript.

2. in order to improve understanding the authors definition of the term 'haplotype' should be included in the manuscript since various definitions have been used in other publications.

Response: We agree and have added a definition of ‘haplotype’ to the manuscript.

3. The labels of the heatmaps in figure 3 are not readable. It would be nice to be able link the gene expression to the pathway.

Response: There's quite a number of genes included in the heatmap of Figure 3. Thus, the labels for individual genes are quite small. We will upload a high-resolution image of Figure 3 to the project’s data repository in PURR.

4. The y-axis labels of figure S6-b are not readable

Response: We revised Fig. S6 and the labels are now readable.

5. Figure S3 has a very low resolution

Response: We revised Fig. S3 to improve the resolution.

Click here to access/download
Supplementary Material
Supplement-BB_December_2018.pdf

Extended Data Table 1

Click here to access/download
Supplementary Material
Extended_Data_Table1.xlsx

Extended Data Table 2

Click here to access/download
Supplementary Material
Extended_Data_Table2.xlsx

Extended Data Table 3

Click here to access/download
Supplementary Material
Extended_Data_Table3.xlsx

Extended Data Table 4

Click here to access/download
Supplementary Material
Extended_Data_Table4.xlsx

Extended Data Table 5

Click here to access/download
Supplementary Material
Extended_Data_Table5.xlsx

Extended Data Table 6

Click here to access/download
Supplementary Material
Extended_Data_Table6.xlsx
