# Supplementary material for: Haplotype-phased genome and evolution of phytonutrient pathways of tetraploid blueberry
Source: Gigascience. 2019 Jan 31;8(3):giz012. doi: 10.1093/gigascience/giz012 (PMC6423372; doi:10.1093/gigascience/giz012)
Supplement: Supplemental Files [file giz012_supplemental_files.zip › Supplement-BB_December_2018.pdf]

Supplementary Information

1. Genome Assembly

1) Reads pre-processing. PCR duplicates, illumina adaptor AGATCGGAAGAGC and Nextera linkers (for MP libraries) were removed. The PE 470bp 2×265bp libraries (Supplemental Table 1) overlapping reads were merged with minimal required overlap of 10bp to create the stitched reads.
2) Error correction. Following pre-processing, merged PE reads were scanned to detect and filter reads with putative sequencing error (contain a sub-sequence that does not reappear in other reads).
3) Contigs assembly. The first step of the assembly consists of building a *de bruijn* graph (kmer=127 bp) of contigs from the all PE & MP reads (Table S1). Next, PE reads were used to find reliable paths in the graph between contigs for repeat resolving and contigs extension. 10X barcoded reads were mapped to contigs ensure that adjacent contigs were connected only in case there is an evidence that those contigs originate from a single stretch of genomic sequence (reads from the same two or more barcodes were mapped to both contigs).

Table S1: Genomic Sequencing Data

**Library Type**	**Read Length**	**Insert Size**	**NCBI SRA #**
PCR-free	2x265bp	470bp	PRJNA494180
PCR-free	2x160bp	800bp	PRJNA494180
Mate-Pair	2x150bp	4000bp	PRJNA494180
Mate-Pair	2x150bp	7000bp	PRJNA494180
Mate-Pair	2x150bp	10000bp	PRJNA494180
Chromium	2x150bp	-	PRJNA494180

4) Scaffolds assembly. Later, contigs were linked into scaffolds with PE and MP information, estimating gaps between the contigs according to the distance of PE and MP links. In addition, 10X data was used to validate and support correct phasing during scaffolding.
5) Fill Gaps. A final fill gap step used PE and MP links and *de bruijn* graph information to detect a unique path connecting the gap edges.
6) Scaffolds elongation and refinement. 10X barcoded reads were mapped to the assembled scaffolds and clusters of reads with the same barcode mapped to adjacent contigs in the scaffolds were identified to be part of a single long molecule. Next, each scaffold was scanned with a 20kb length window to ensure that the number of distinct clusters that cover the entire window (indicating a support for this 20kb connection by several long molecules) was statistically significant with respect to the number of clusters that span the left and the right edge of the window. In case where a potential scaffold assembly error was detected the scaffold was broken at the two edges of the suspicious 20kb window. Finally, the barcodes that were mapped to the scaffold edges were compared (first and last 20kb sequences) to generate a scaffolds graph with a link connecting two scaffolds with more than two common barcodes. Linear scaffolds paths in the scaffolds graph were composed into the final scaffolds output of the assembly (Table S2).

Table S2: Assembly statistics of the tetraploid blueberry.

	Contigs	Scaffolds
**Total Sequences**	160,465	79,529
**Assembly size (bp)**	1,892,444,017	1,917,911,071
**Gaps%**	36,807	1.28
**N50**	15,420	186
**N90**	8,267	346,108
**N90 #sequences**	54,465	841
**MAX**	328,329	26,056,541

7) HiC Scaffolding. The scaffolds, raw reads, and Dovetail HiC library reads were used as input data (Figure S1) for HiRise, a software pipeline designed specifically for using proximity ligation data to scaffold genome assemblies to chromosome-length pseudomolecules (Figure S2). A total of 508 million 151bp paired-end read pairs were sequenced; equating to ~91.4x sequence depth across the genome.

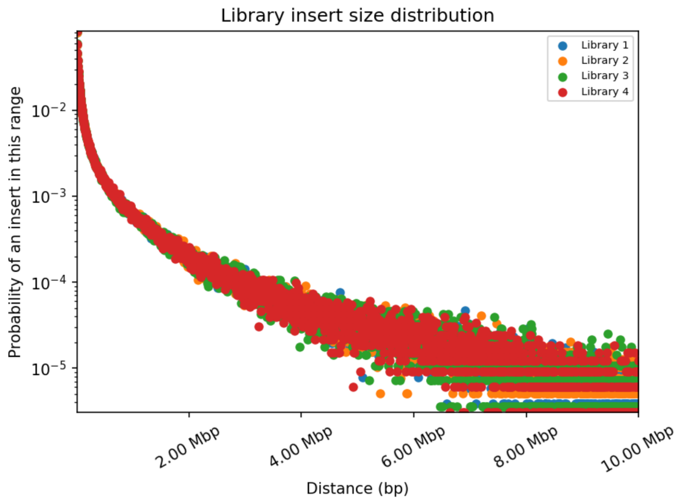

Figure S1: Distribution of insert sizes in the Dovetail library. The distance between the forward and reverse reads is given on the X-axis in basepairs, and the probability of observing a read pair with a given insert size is shown on the Y- axis.

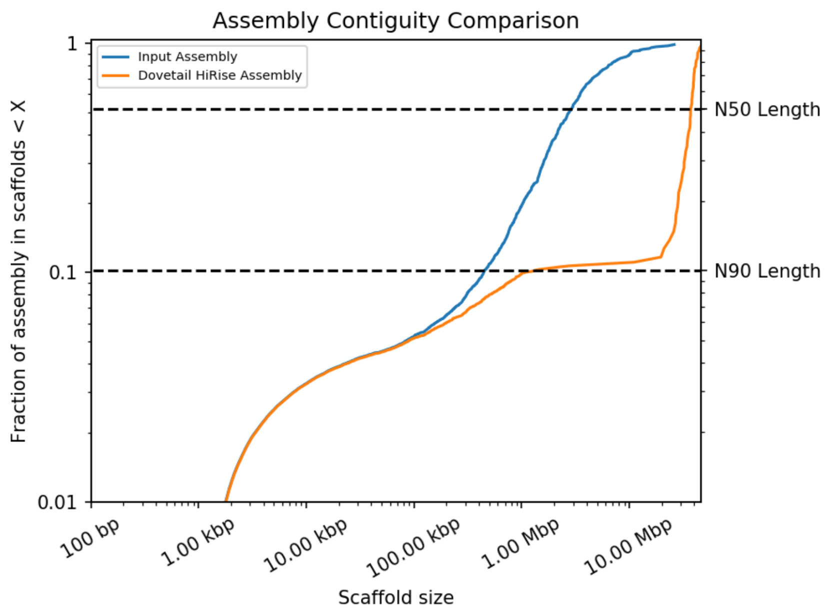

Figure S2: A comparison of the contiguity of the input assembly and the final HiRise scaffolds. Each curve shows the fraction of the total length of the assembly present in scaffolds of a given length or smaller. The fraction of the assembly is indicated on the Y-axis and the scaffold length in basepairs is given on the X-axis. The two dashed lines mark the N50 and N90 lengths of each assembly. Scaffolds less than 1 kb are excluded.

8) Raw illumina data generated in this study has been deposited into the NCBI-SRA under BioProject #PRJNA494180, and final assembly and annotation will be made publicly available on Genome Database for Rosaceae (https://www.rosaceae.org/) and CyVerse CoGe platform (https://genomevolution.org/).

2. Genome Annotation

Table S3: Counts and lengths of annotated features

Features	Protein coding genes
**Number of genes**	128,559
**Number of exons**	671,022
**Number of CDS**	128,559
**Total gene length**	691,164,704
**Total exon length**	193,144,223
**Total CDS length**	151,554,537
**Mean gene length**	5,376
**Mean exon length**	288
**Mean CDS length**	1,179

Table S4: Libraries used for genome annotation

Sample	PF Reads	Yield (Gbp)
Root	24,890,296	7.47
Salt-treated Root	19,835,260	5.95
Leaf (day)	25,651,348	7.70
Leaf (night)	30,707,874	9.21
MJ-treated leaf (8hr)*	25,210,772	7.56
MJ-treated leaf (24hr)*	35,066,276	10.52
Flower bud	28,022,120	8.41
Flower at anthesis	41,926,773	12.58
Flower post-fertilization	35,054,435	10.52
Green fruit	30,615,054	9.18
Pink fruit	35,399,713	10.62
Ripe fruit	32,988,753	9.90
Young shoot	28,669,542	8.60

*MJ = Methyl jasmonate

Table S5: Summary results of BUSCO analysis.

Complete BUSCOs	1,375
Complete and single-copy BUSCOs	151
Complete and duplicated BUSCOs	1,224
Fragmented BUSCOs	19
Missing BUSCOs	46
Total BUSCO groups searched	1,440

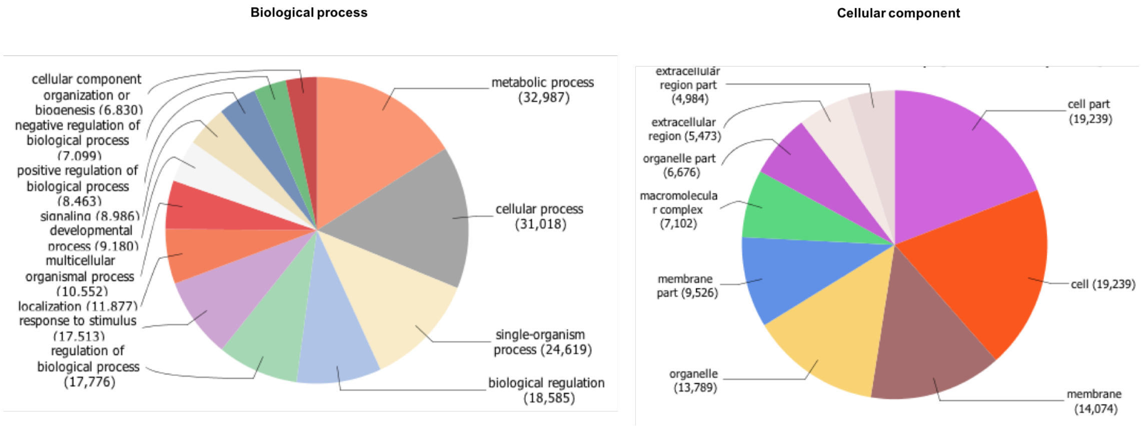

Figure S3: Functional annotation of protein coding genes of blueberry using BLAST2GO.

Table S6: Transposable elements and other repetitive sequences in the genome.

	Super-family	No. of TE[a] (x1000)	Coverage (Mb)	Fraction of genome[b] (%)
**Class I**	LTR/*Copia*	154.5	51.20	2.84
	LTR/*Gypsy*	343.4	213.15	11.83
	LTR/Unknown	449.7	144.06	8.00
	LINE	75.3	32.04	1.78
	SINE	77.9	10.54	0.59
	**Total Class I**	1100.8	450.99	25.04
**Class II**	CACTA	41.2	9.17	0.51
	hAT	383.8	78.51	4.36
	MULE	328.7	60.47	3.36
	PIF-Harbinger/Tourist	278.6	50.43	2.80
	MLE/*Stowaway*	100.5	17.58	0.98
	Unknown	15.8	3.23	0.18
	**Total class II**	1148.6	219.39	12.19
**Total TEs**		2249.4	670.38	37.23
Other repeats[c]		9.3	1.94	0.11
Unknown repeats		704.7	125.52	6.97
**Total Repeats**		2963.4	797.84	44.31

[a]Intact plus fragments; [b]Using non-gap sequence as base; [c]Including rRNA, snRNA, and simple repeats

3. Genome Analyses

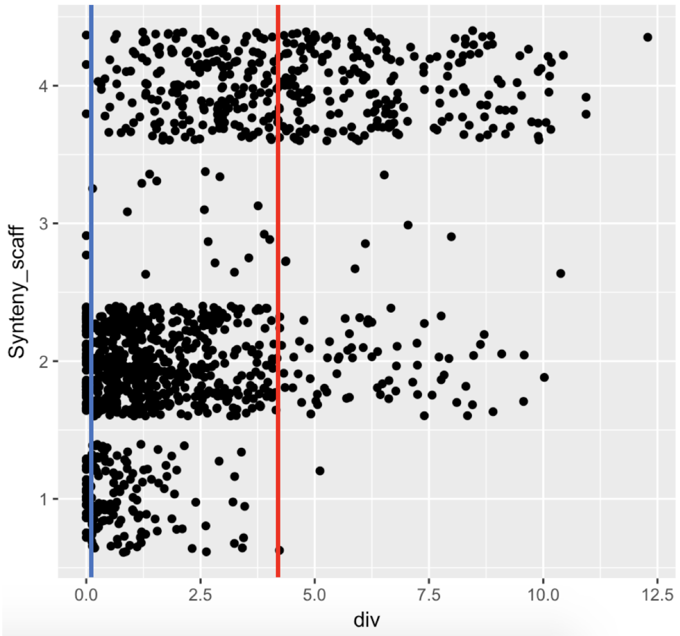

Figure S4: LTR element content and sequence divergence across homoeologous chromosomes. The number of syntenic LTR elements are shown on the y-axis with sequence divergence shown on the x-axis. The red line indicates the putative divergence of the diploid progenitors from the most common recent ancestor based on sequence similarity of both genes and LTRs. The age of the polyploid formation of highbush blueberry occured in North America [1–3], following the last glacial maximum (~20,000 years ago) [4], is shown with the blue line.

To distinguish between the allo- or auto- tetraploid origin of the highbush blueberry genome, we also identified and analyzed LTR elements that have syntenic insertion sites shared among the four chromosome copies. These elements were termed syntenic LTR elements, and their number in syntenic chromosomes were counted (y-axis in Figure S4). For allotetraploid species, formed by joining two different genomes, the majority of syntenic LTR elements should only be shared by two chromosome copies until their most recent common ancestor (red line in Figure S4; reflecting speciation of the diploid progenitors) but before polyploid formation (blue line in Figure S4). In contrast, for autotetraploid species that are formed by duplicating the entire diploid genome, all four syntenic chromosomes would share near identical LTR element content.

We observed the majority of syntenic LTR elements are shared among four syntenic chromosomes prior the LTR divergence of 4.4% (Fig. S4, red line). The syntenic count quickly dropped from four to two within LTR divergence of 4.0-4.4% (Fig. S4), closely matching the estimate for speciation of the diploid progenitors (the most common recent ancestor) based on sequence divergence of homoeologous genes (~96%). After speciation, the majority of syntenic LTR elements are shared by only two chromosomes (red line in Figure S4), supporting allopolyploid origin of the tetraploid highbush blueberry.

LTR-RT annotation

De novo identification of intact LTR retrotransposons (LTR-RTs) were performed using LTR_retriever v1.6 with default parameters [5]. The insertion time of each intact LTR-RT is estimated by LTR_retriever based on $T = K/2\mu$ where K is the divergence between the LTR pair and μ is the mutation rate of 1.3×10^{-8} per bp per year [5]. Whole-genome LTR sequence annotation were achieved using the non-redundant LTR-RT library generated by LTR_retriever and RepeatMasker v4.0.0 (www.repeatmasker.org).

Origin of the tetraploid blueberry

To identify syntenic LTR-RT elements, two 200 bp sequences were extracted from both sides of each intact LTR-RT, with 100 bp overlapping the LTR region and 100 bp overlapping the flanking region. These sequences were searched against the entire genome using BLAST for identification of syntenic regions. A full match is determined if 150 bp or more of the 200-bp sequence is aligned, while a site match is determined if 80 bp or more of the flanking region is aligned. An intact LTR-RT was deemed informative if both flanking sequences of the element were found on all four homoeologous chromosomes, then the number of full match of each informative intact LTR-RT was counted. The number of full match for each informative LTR element was plotted against the divergence of its LTR region using *ggplot()* in R [6].

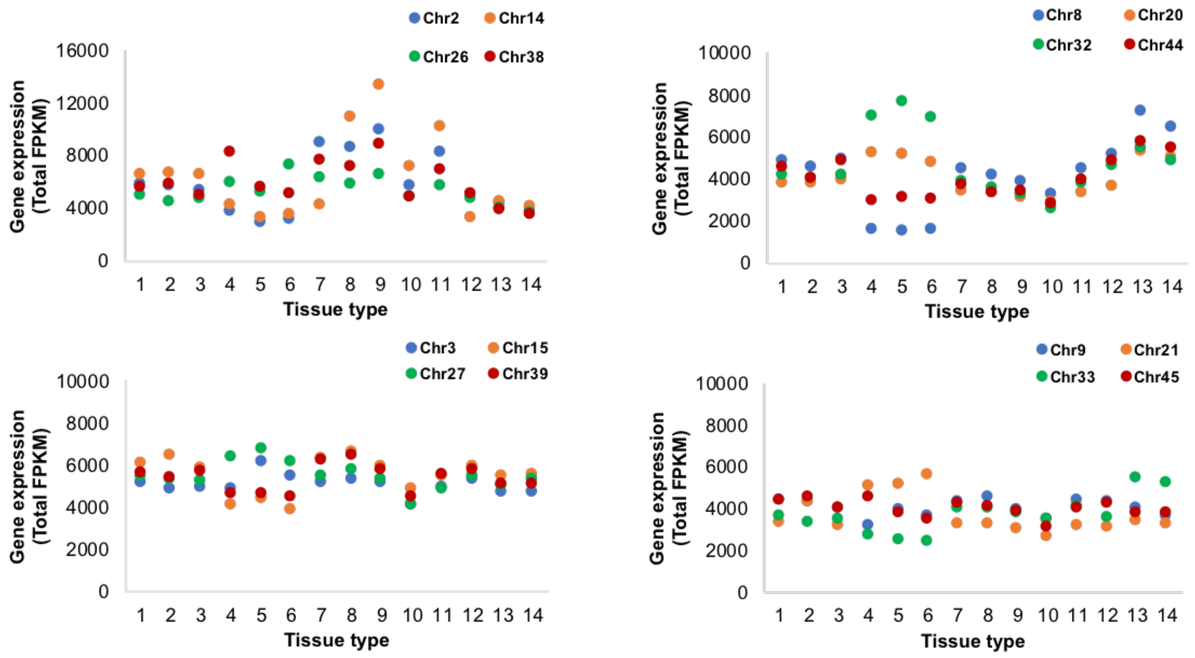
Chr2
Chr14
Chr26
Chr38
Gene expression (Total FPKM)
Tissue type
Chr8
Chr20
Chr32
Chr44
Chr3
Chr15
Chr27
Chr39
Chr9
Chr21
Chr33
Chr45

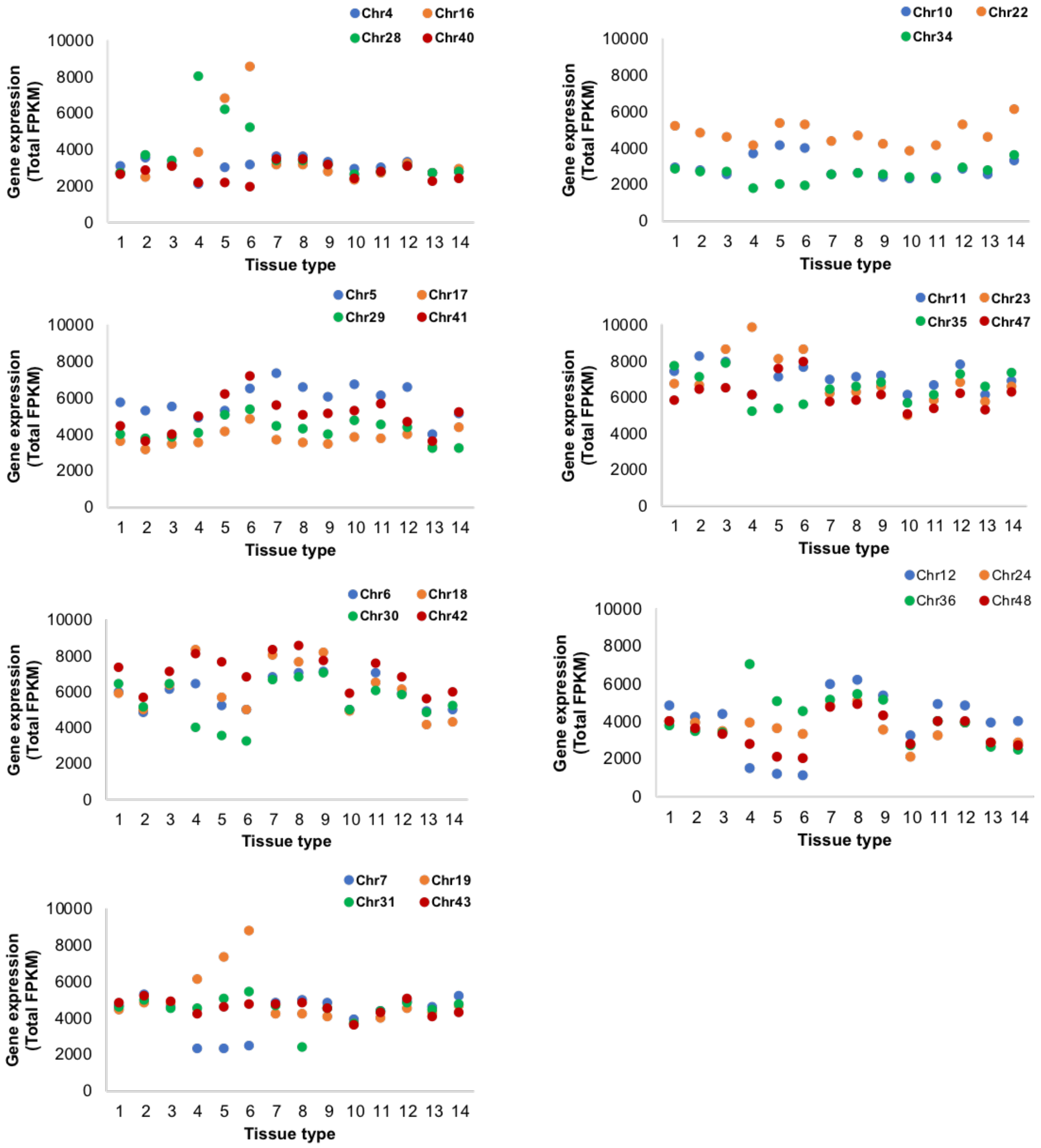

Figure S5: Gene expression (total FPKM) bias among homoeologous chromosomes (1=flower bud; 2=flower at anthesis; 3=petal fall; 4=green fruit; 5=pink fruit; 6=ripe fruit; 7, 8=leaf collected at 12 p.m. and 12 a.m., respectively; 9, 10, 11=methyl jasmonate treated leaf collected after one hour, eight hours and 24 hours, respectively; 12=shoot; 13=root; 14=salt-treated root). See Figure 2A for comparisons among Chromosomes 1, 13, 25, and 37. The most dominant homoeologous chromosome for the above comparisons are 14, 15, 4, 5, 42, 7, 8, 9, 22, 11, 12.

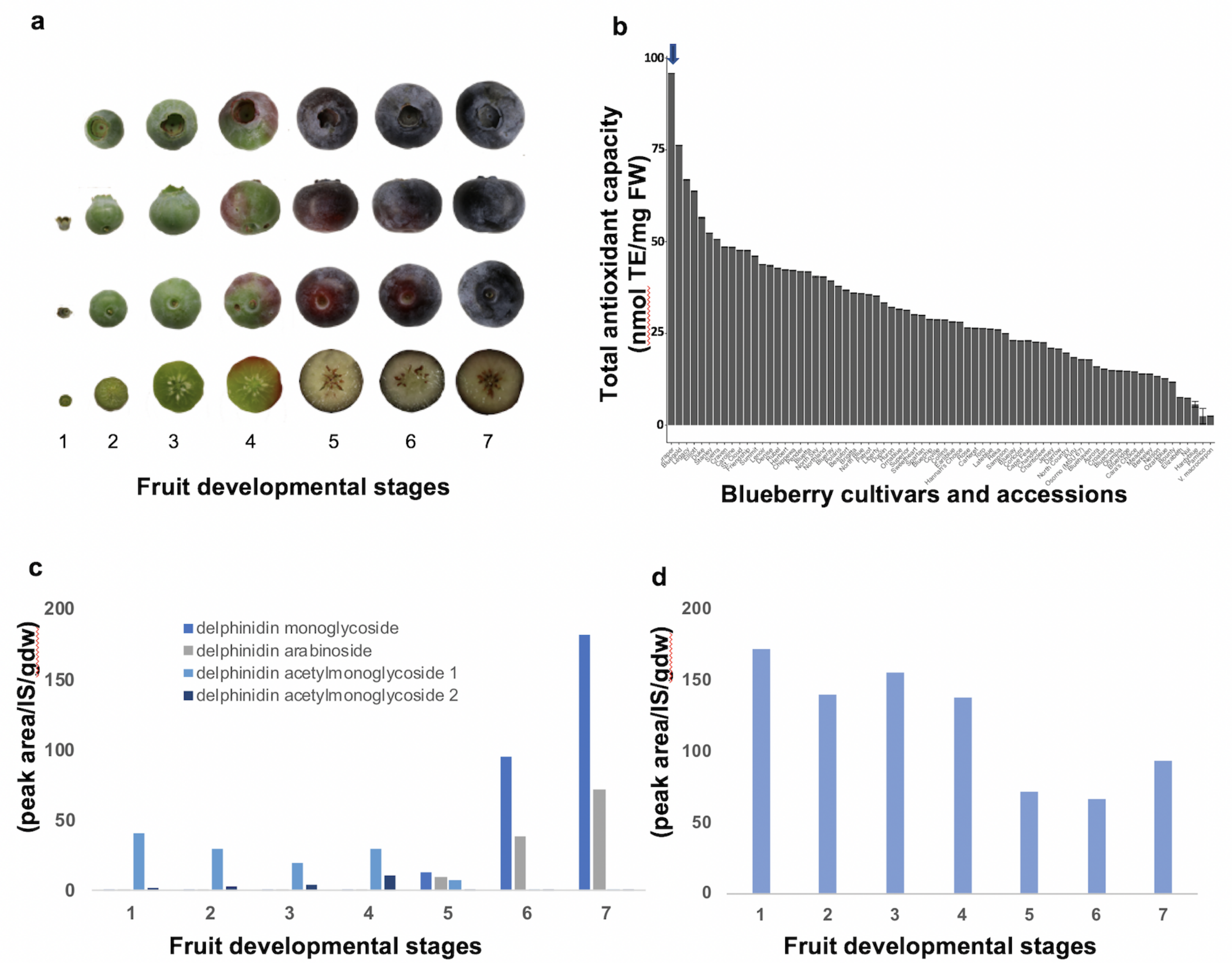

Figure S6: Antioxidant capacity in blueberry. (a) External and internal morphological changes during berry development (1=petal fall, 2= small green fruit, 3= expanding green fruit, 4=pink fruit, 5= fruit color completely changed from pink to purple, 6=unripe, 7=ripe). (b) Oxygen radical absorbance capacity (ORAC) of blueberry cultivars ('Draper' is indicated by the blue arrow). (c) Delphinidin and (d) chlorogenic acid profiles during blueberry fruit development.

Table S7: KEGG enrichment of highly expressed genes in young (a) and ripening (b) blueberry fruit.

**(a) KEGG ID**	**Description**	**p-value**
map00910	Nitrogen metabolism	0.01
map00073	Cutin, suberine and wax biosynthesis	0.01
map00940	Phenylpropanoid biosynthesis	0.04

**(b) KEGG ID**	**Description**	**p-value**
map00052	Galactose metabolism	0.02
map00500	Starch and sucrose metabolism	0.04
map00051	Fructose and mannose metabolism	0.03

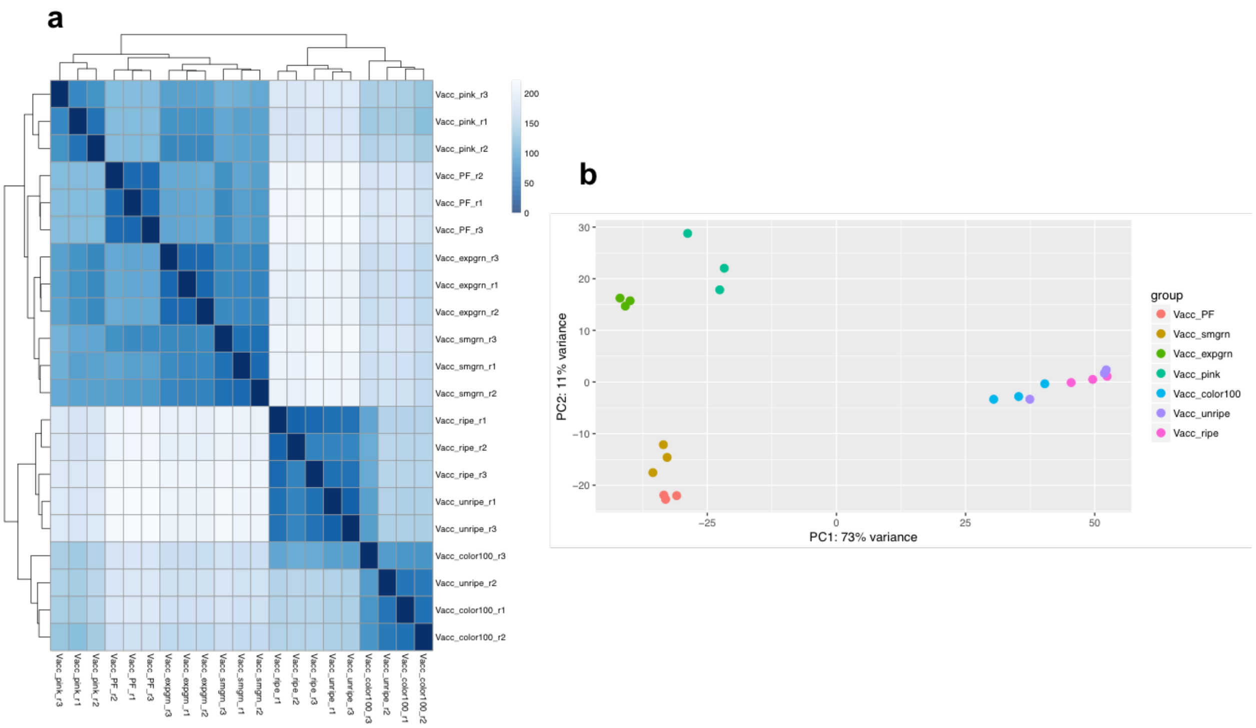

Figure S7: (a) Fruit sample distance based on gene expression. (b) Principal component analysis of transcripts expressed at the seven different fruit ages.

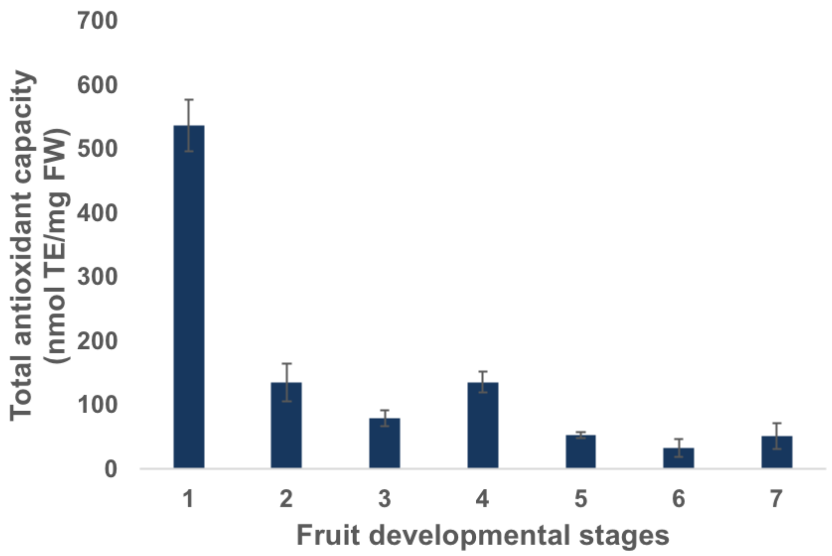

Figure S8: Antioxidant capacity in blueberry fruit at different developmental stages (1=petal fall, 2= small green fruit, 3= expanding green fruit, 4=turning fruit, 5= fruit color completely changed from green to purple, 6=unripe fruit, 7=ripe fruit).

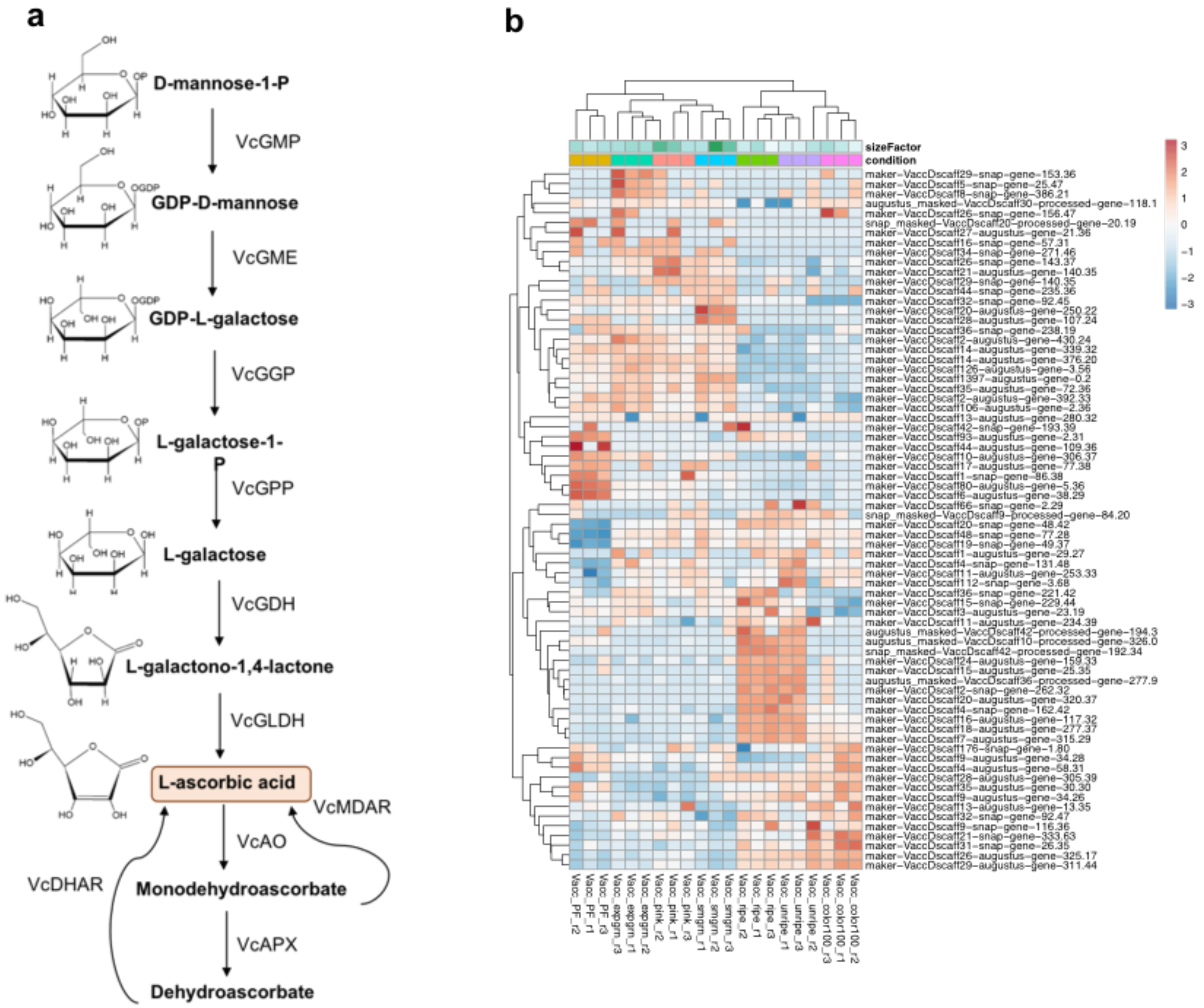

Figure S9: (a) Schematic presentation of predicted ascorbate biosynthesis pathway in blueberry based on previously described ascorbic acid biosynthesis pathway in plants [7] and (b) expression of genes associated with ascorbate biosynthesis.

References:

1. Kloet, S. P. V. The taxonomy of the highbush blueberry, Vaccinium corymbosum. *Can. J. Bot.* **58,** 1187–1201 (1980).
2. Vander Kloet, S. P. & Others. *The genus Vaccinium in North America*. (Agriculture Canada, 1988).
3. Ramsey, J. & Ramsey, T. S. Ecological studies of polyploidy in the 100 years following its discovery. *Philos. Trans. R. Soc. Lond. B Biol. Sci.* **369,** (2014).
4. Clark, P. U. *et al.* The Last Glacial Maximum. *Science* **325,** 710–714 (2009).
5. Ou, S. & Jiang, N. LTR_retriever: A Highly Accurate and Sensitive Program for Identification of Long Terminal Repeat Retrotransposons. *Plant Physiol.* **176,** 1410–1422 (2018).
6. Wickham, H. *ggplot2: Elegant Graphics for Data Analysis*. (Springer, 2016).
7. Liu, F. *et al.* Higher transcription levels in ascorbic acid biosynthetic and recycling genes were associated with higher ascorbic acid accumulation in blueberry. *Food Chem.* **188,** 399–405 (2015).
