# Supplementary material for: Haplotype-phased genome and evolution of phytonutrient pathways of tetraploid blueberry
Source: Gigascience. 2019 Jan 31;8(3):giz012. doi: 10.1093/gigascience/giz012 (PMC6423372; doi:10.1093/gigascience/giz012)
Supplement: Response_to_Reviewer_Comments_Original_Submission.pdf [file giz012_response_to_reviewer_comments_original_submission.pdf]

Author's Response To Reviewer Comments

Close

Dear Reviewers and Editors:
Thank you for a thorough and very thoughtful review of our manuscript. We have made several changes to our manuscript to address points raised by Reviewer #2, which truly helped improve the overall quality of the manuscript. This includes slightly changing the title of the manuscript based on comment #4 to “Haplotype-phased genome and evolution of phytonutrient pathways of tetraploid blueberry”. We feel that this new title more accurately reflects the highlights of the paper.

Best,

Patrick Edger

Reviewer reports:
Reviewer #1: This is a well-prepared manuscript. The results provide an important platform for further research
on this and related species. The specific gene groups studied are appropriate to the unique features of this system.
The observations on sub genome contributions are also important contributions to understanding of polyploid systems.

Response: Thank you for the positive feedback on our manuscript.

Reviewer #2: The manuscript presents a chromosome-scale haplotype phased genome assembly of
highbush blueberry which is of high economic importance mainly due to its composition of health
promoting phytonutrients. Genes and pathways associated with antioxidant and sugar levels in blueberry
fruits were analyzed in more detail. Overall this work provides a valuable new genomic reference for
blueberry and enables future studies on the genome of blueberry for research and breeding purposes.
Furthermore, the findings give insights into expression patterns of genes associated with fruit ripening
and antioxidant biosynthesis as well as the expansion of gene families related to these traits. Finally, it is
shown that blueberry is an allopolyploid species with subgenome dominance. The presented reference
genome sequence is of high quality and both the gene annotation and TE analysis are sound.
Transcriptome analyses were properly carried out, although many of the results are mainly confirming
prior assumptions. The manuscript could further be improved by considering these points:

Major:
1. the authors claim that there is a high average sequence similarity among syntenic homeologous genes
(96.3%) and that there is a divergence between syntenic homeologous genes of ~0.036 per synonymous
site. They thus conclude that blueberry is allopolyploid. It is not obvious how this conclusion was made
and it should be further elaborated on the connection between allopolyploidy and the mentioned numbers.

Response: We thank you for this comment and have revised the manuscript to help clarify this to the
reader. We completely agree that additional details were needed. Furthermore, we added a time estimate
for the polyploid event based on dating unique LTR insertions.

2. in the case of genes involved in anthocyanin and chlorogenic acid biosynthesis, various tandem
duplicates were identified. Are these all functional genes and not pseudogenes? Also, a more detailed
description or analysis of the expression patterns of tandem duplicated genes and the mentioned gene
family expansions would be desirable. This would shed light on possible dosage effects and put the
analysis into a biological context with the other transcriptome analyses.

Response: We have added additional details regarding the expression of tandem duplicated genes to the
manuscript in the 'Expansion of antioxidant-related gene families through tandem duplication' section
and have revised the 'Transcriptome assembly and gene-expression analysis' section in the methods. In
short, 83.4% the tandem duplicates were expressed in at least one transcriptome library with 73.5%
expressed in at least one of the fruit transcriptome libraries. We agree that additional transcriptome
analyses with more diverse libraries are needed to provide further insights into the dosage effects of these
duplicate genes and their possible involvement in fruit development and quality traits. We are currently
working on generating additional datasets and analyses which will be included in a follow-up manuscript
later next year. The goal for this manuscript was to provide a new genomic resource and research findings
to the blueberry community to enable various future research efforts including to investigate the evolution

of duplicate genes.

3. in that context, and especially for an allopolyploid, a more detailed analysis of pseudogenes and gene
fragments would be very interesting.

Response: We recognize the importance of differentiating pseudogenes from “real” genes as some
pseudogenes are linked to certain biological functions. There are available tools to predict pseudogenes,
however, they have limitations and characterizing a pseudogene remains difficult given its high similarity
to the “real” genes. Also, to verify if a pseudogene is functional or not, we need to set up experiments to
examine the presence or absence of biological function of a pseudogene. At this point, we believe that
performing such extensive experiments is beyond the scope of this paper. However, we agree with you
that this would likely yield very interesting results.

4. the authors found a difference in gene expression levels between the two subgenomes and hypothesize
this might be due to differences in transposon density around homeologous genes. Since transposon
density was also measured, it should be included in the manuscript whether or not TE-density correlates
with subgenome specific gene expression levels.

Response: Transposable element (TE) content differences among homoeologous genes may not play
major role in highbush blueberry. Each of the homoeologous chromosomes have relatively similar total
TE content. For example, there is only a ~1.85% differences in TE content between chromosomes 1, 13,
25 and 37. Chromosomes 1 and 13 having the most and least amount of TEs, respectively. The most
dominantly expressed subgenome typically has the lowest amount of methylated TEs near genes. We are
currently in the process of generating the datasets to look at the methylation status of individual TEs in
different organs and developmental stages and to compare this to gene expression patterns. These
analyses will take us several months (up to a year) to complete and thus will be included as part of followup
manuscript. The observed expression patterns shown in Figure 2B are very interesting. These are very

different from what has been observed in most other allopolyploids exhibiting subgenome expression
dominance - a single dominant subgenome. However, as you point out below, these findings are quite
preliminary (i.e. "hints"). For these reasons, we have decided to change the title of the manuscript and
have revised the manuscript to state that these findings are preliminary and require follow-up studies.

5. The mapping of reads retrieved from RNA-Seq data to the genome was performed uniquely. Since
highly similar genomic regions are in general problematic when performing gene expression studies, it
should be included in the description of the method whether non-unique reads where mapped randomly or
excluded from the mapping.

Response: We excluded the non-unique reads from the analyses. We revised the 'Transcriptome
assembly and gene-expression analysis' section in the methods to include this additional information.

6. gene expression was analyzed across 14 different samples and total gene expression values were used
to compare total gene expression across haplotypes. However, replicates were only available for fruit
samples but not for all other samples. Hence, the findings here should be described as hints. Moreover, it
should be explained how the fruit samples were treated in this analysis (was only one replicate used or the
average count of all three?).

Response: Total gene expression values for subgenome dominance analysis were derived from a single
biological replicate of different tissue types (1=flower bud; 2=flower at anthesis; 3=petal fall; 4=green
fruit; 5=pink fruit; 6=ripe fruit; 7, 8=leaf collected at 12 p.m. and 12 a.m., respectively; 9, 10, 11=methyl
jasmonate treated leaf collected after one hour, eight hours and 24 hours, respectively; 12=shoot; 13=root;
14=salt-treated root). To eliminate potential variability in gene expression due to environmental effects,
we only analyzed the data from tissues collected from 'Draper' grown in the growth chamber (i.e.
identical environmental conditions). We completely agree that these findings are quite preliminary and

require follow-up analyses. Thus, we have added additional text to the manuscript to address this
important point and removed several paragraphs in the Discussion section.

7. The citations of extended table 4 and 5 in the text seem to be wrong! I believe 5 is meant to be 4 and 6
(which doesn't exist) should be 5. This needs to be fixed. In general there seems to be a problem with the
formatting ("error for extended table 2") and content of the extended tables. I would suggest to deposit
them under a public data DOI instead of having them attached to the main manuscript.

Response: We apologize for this mix-up. We have corrected Extended data tables 4, 5, and 6. We
checked Extended data table 2 and did not discover any formatting issues. The file size is a bit large, thus,
it may take a few moments for some computers to load all of its contents.

8. I recommend that the manuscript is proof read in order to improve language, sentence structure,
grammar and typing errors.

Response: We have carefully edited the manuscript to correct these various errors.

Minor:

1. I would recommend not to use the term "expressed chromosomes".

Response: We agree and have removed this term from the manuscript.

2. in order to improve understanding the authors definition of the term 'haplotype' should be included in
the manuscript since various definitions have been used in other publications.

Response: We agree and have added a definition of ‘haplotype’ to the manuscript.

3. The labels of the heatmaps in figure 3 are not readable. It would be nice to be able link the gene
expression to the pathway.

Response: There's quite a number of genes included in the heatmap of Figure 3. Thus, the labels for
individual genes are quite small. We will upload a high-resolution image of Figure 3 to the project’s data
repository in PURR.

4. The y-axis labels of figure S6-b are not readable

Response: We revised Fig. S6 and the labels are now readable.

5. Figure S3 has a very low resolution

Response: We revised Fig. S3 to improve the resolution.

Close
