# Supplementary material for: Haplotype-phased genome and evolution of phytonutrient pathways of tetraploid blueberry
Source: Gigascience. 2019 Jan 31;8(3):giz012. doi: 10.1093/gigascience/giz012 (PMC6423372; doi:10.1093/gigascience/giz012)
Supplement: Reviewer_2_report_original_submission_revise -- Manuel Spannagl — 10/18/2018 Reviewed [file giz012_reviewer_2_report_original_submission_revise.pdf]

Reviewer Report

Title: Haplotype-phased genome and evolution of phytonutrient pathways of tetraploid blueberry

Version: Original Submission **Date:** 10/18/2018

Reviewer name: Manuel Spannagl

This manuscript was co-reviewed by myself and Dr. Nadia Kamal.

Reviewer Comments to Author:

The manuscript presents a chromosome-scale haplotype phased genome assembly of highbush blueberry which is of high economic importance mainly due to its composition of health promoting phytonutrients. Genes and pathways associated with antioxidant and sugar levels in blueberry fruits were analyzed in more detail.
Overall this work provides a valuable new genomic reference for blueberry and enables future studies on the genome of blueberry for research and breeding purposes. Furthermore, the findings give insights into expression patterns of genes associated with fruit ripening and antioxidant biosynthesis as well as the expansion of gene families related to these traits. Finally, it is shown that blueberry is an allopolyploid species with subgenome dominance. The presented reference genome sequence is of high quality and both the gene annotation and TE analysis are sound. Transcriptome analyses were properly carried out, although many of the results are mainly confirming prior assumptions. The manuscript could further be improved by considering these points:

Major:

1. the authors claim that there is a high average sequence similarity among syntenic homeologous genes (96.3%) and that there is a divergence between syntenic homeologous genes of ~0.036 per synonymous site. They thus conclude that blueberry is allopolyploid. It is not obvious how this conclusion was made and it should be further elaborated on the connection between allopolyploidy and the mentioned numbers.
2. in the case of genes involved in anthocyanin and chlorogenic acid biosynthesis, various tandem duplicates were identified. Are these all functional genes and not pseudogenes? Also, a more detailed description or analysis of the expression patterns of tandem duplicated genes and the mentioned gene family expansions would be desirable. This would shed light on possible dosage effects and put the analysis into a biological context with the other transcriptome analyses.
3. in that context, and especially for an allopolyploid, a more detailed analysis of pseudogenes and gene fragments would be very interesting.
4. the authors found a difference in gene expression levels between the two subgenomes and hypothesize this might be due to differences in transposon density around homeologous genes. Since transposon density was also measured, it should be included in the manuscript whether or not TE-density correlates with subgenome specific gene expression levels.
5. The mapping of reads retrieved from RNA-Seq data to the genome was performed uniquely. Since highly similar genomic regions are in general problematic when performing gene expression studies, it should be included in the description of the method whether non-unique reads where mapped randomly or excluded from the mapping.

6. gene expression was analyzed across 14 different samples and total gene expression values were used to compare total gene expression across haplotypes. However, replicates were only available for fruit samples but not for all other samples. Hence, the findings here should be described as hints. Moreover, it should be explained how the fruit samples were treated in this analysis (was only one replicate used or the average count of all three?).
7. The citations of extended table 4 and 5 in the text seem to be wrong! I believe 5 is meant to be 4 and 6 (which doesn't exist) should be 5. This needs to be fixed. In general there seems to be a problem with the formatting ("error for extended table 2") and content of the extended tables. I would suggest to deposit them under a public data DOI instead of having them attached to the main manuscript.
8. I recommend that the manuscript is proof read in order to improve language, sentence structure, grammar and typing errors.

Minor:

1. I would recommend not to use the term "expressed chromosomes".
2. in order to improve understanding the authors definition of the term 'haplotype' should be included in the manuscript since various definitions have been used in other publications.
3. The labels of the heatmaps in figure 3 are not readable. It would be nice to be able link the gene expression to the pathway.
4. The y-axis labels of figure S6-b are not readable
5. Figure S3 has a very low resolution

Methods

Are the methods appropriate to the aims of the study, are they well described, and are necessary controls included? Choose an item.

Conclusions

Are the conclusions adequately supported by the data shown? Choose an item.

Reporting Standards

Does the manuscript adhere to the journal's guidelines on minimum standards of reporting? Choose an item.

Choose an item.

Statistics

Are you able to assess all statistics in the manuscript, including the appropriateness of statistical tests used? Choose an item.

Quality of Written English

Please indicate the quality of language in the manuscript: Choose an item.

Declaration of Competing Interests

Please complete a declaration of competing interests, considering the following questions:

- Have you in the past five years received reimbursements, fees, funding, or salary from an organisation that may in any way gain or lose financially from the publication of this manuscript, either now or in the future?
- Do you hold any stocks or shares in an organisation that may in any way gain or lose financially from the publication of this manuscript, either now or in the future?
- Do you hold or are you currently applying for any patents relating to the content of the manuscript?
- Have you received reimbursements, fees, funding, or salary from an organization that holds or has applied for patents relating to the content of the manuscript?
- Do you have any other financial competing interests?
- Do you have any non-financial competing interests in relation to this paper?

If you can answer no to all of the above, write 'I declare that I have no competing interests' below. If your reply is yes to any, please give details below.

I declare that I have no competing interests

I agree to the open peer review policy of the journal. I understand that my name will be included on my report to the authors and, if the manuscript is accepted for publication, my named report including any attachments I upload will be posted on the website along with the authors' responses. I agree for my report to be made available under an Open Access Creative Commons CC-BY license (http://creativecommons.org/licenses/by/4.0/). I understand that any comments which I do not wish to be included in my named report can be included as confidential comments to the editors, which will not be published.

I agree to the open peer review policy of the journal

To further support our reviewers, we have joined with Publons, where you can gain additional credit to further highlight your hard work (see: https://publons.com/journal/530/gigascience). On publication of this paper, your review will be automatically added to Publons, you can then choose whether or not to claim your Publons credit. I understand this statement.

Yes Choose an item.
